# Integrative proteomics, phosphoproteomics and acetylation proteomics analyses of acute pancreatitis in rats

**DOI:** 10.7150/ijms.81658

**Published:** 2023-05-11

**Authors:** Ju Zhang, Zuxing Wei, Xiaoyan Qi, Xuyang Hou, Dekun Liu, Jun He

**Affiliations:** Department of General Surgery, The Second Xiangya Hospital, Central South University, Changsha, Hunan 410011, China.

**Keywords:** Proteomics, Phosphoproteomics, Acetylation, Acute pancreatitis, Severe acute pancreatitis

## Abstract

Acute pancreatitis (AP) is a common acute abdominalgia of the digestive tract. When the disease progresses to severe acute pancreatitis (SAP), the complications and mortality rate greatly increase. Determining the key factors and pathways underlying AP and SAP will help elucidate the pathological processes involved in disease progression and will be beneficial for identifying potential therapeutic targets. We conducted an integrative proteomics, phosphoproteomics and acetylation proteomics analysis of pancreas samples collected from normal, AP and SAP rat models. We identified 9582 proteins, 3130 phosphorylated modified proteins, and 1677 acetylated modified proteins across all samples. The differentiated expression proteins and KEGG pathway analysis suggested the pronounced enrichment of key pathways based on the following group comparisons: AP versus normal, SAP versus normal, and SAP versus AP. Integrative proteomics and phosphoproteomics analyses revealed 985 jointly detected proteins in the comparison of AP and normal samples, 911 proteins in the comparison of SAP and normal samples, and 910 proteins in the comparison of SAP and AP samples. Based on proteomics and acetylation proteomics analyses, we found that 984 proteins were jointly detected in the comparison of AP and normal samples, 990 proteins in SAP and normal samples, and 728 proteins in SAP and AP samples. Thus, our study offers a valuable resource to understand the proteomic and protein modification atlas in AP.

## Introduction

Acute pancreatitis (AP) is a common acute abdominalgia characterized by abdominal pain and upregulation of amylase and lipase in blood^1^. The development of severe AP (SAP) is associated with increased complications, and a 20%-40% mortality rate is noted for SAP^2^. Gallstones and alcohol abuse are the most frequently mentioned causative factors in the initiation of AP, and gallstones and alcohol consumption are often noted in AP patients^3^. The dysfunction of acinar cells, which are responsible for synthesizing, storing, and secreting digestive enzymes, and ductal cells, which are responsible for transporting digestive enzymes to intestines, are the key mechanisms involved in AP^4^. Decades of research using animal models have shed light on the mechanism of initiation and development of AP, which include disorders of Ca^2+^ signaling and endoplasmic reticulum, impaired autophagy and defects in lysosomes, and disorders of acinar cell mitochondrial dynamics^4^. These disorders lead to significant acinar cell death followed by activation of the proinflammatory response, both of which determine the severity and prognosis of AP.

Analysis of protein variations in AP and SAP revealed many key biological events underlying these pathological processes. The expression of lysosomal-associated membrane proteins (LAMPs), which are essential for preserving the structure and function of lysosomes in eukaryotes, was obviously reduced in animal models of AP, and defects in LAMPs spontaneously led to AP in mice^5^. CHOP, a transcription factor essential for endoplasmic reticulum stress, is upregulated in AP, and its inhibition by the antioxidant N-acetylcysteine prevents acinar cell death^6^. Our previous work revealed that the expression of Srxn1, an endogenous antioxidant protein, was dysregulated in AP and SAP. Moreover, inhibition of Srxn1 reduced acinar cell injury and the proinflammatory response, and overexpression of Srxn1 prevented SAP in animal models^7^. On the other hand, variations in the modification of proteins in acinar cells, such as phosphorylated or acetylated modification of proteins, also affect the process of AP. For instance, a high level of phosphorylated TFEB, a master transcription factor of lysosomal biogenesis, leads to its rapid degradation, resulting in impaired lysosomal biogenesis and defective autophagic flux in acinar cells^8^. Regarding protein acetylation, butyrate improves histone acetylation at the H3K9 and H3K14 loci of AP1 and STAT1, leading to suppression of NLRP3 inflammasome activation and decreasing infiltration of inflammatory cells during AP^9^.

The development of “proteomics” and “protein modification-omics” techniques provides a new tool to ascertain the molecular mechanisms and screen the potential therapeutic targets of AP. Proteomic studies have been used to monitor the disease process of AP or SAP in mice or rats^10^. Quantitative simplex tandem mass tag (TMT) analysis has been used to determine the protein variation of AP tissues from early stages, resulting in the identification of 997 unique proteins^11^. Isotope Coded Affinity Tags (ICAT) technology and mass spectrometry (MS)-based proteomics were used to determine the protein variation between normal and chronic pancreatitis^12^-^15^. The low molecular weight proteome of AP tissues was analyzed by liquid chromatography-MS (LC‒MS)^11^, ^12^. Several works have used proteomic methods to explore the therapeutic targets of drugs, such as emodin and Qingyi pellets, in AP. A recent work measured the proteomic and phosphoproteomic profiles of SAP in rats using a data-independent acquisition strategy including a total of 2419 proteins^12^. Nevertheless, a protein profile comparing AP and SAP is needed to expand the understanding of the progression of AP to SAP. Additionally, studies on the protein modification-omics of AP and SAP are sparse, and a systematic profile of modification-omics of AP and SAP would be beneficial to explore the potential therapeutic targets of SAP.

Here, we conducted an integrative proteomic and protein modification-omics (phosphoproteomics and acetylation proteomics) characterization of normal, AP, and SAP pancreases in rats. Our work presents a multiple-omics overview of pancreatic tissues with or without pancreatitis. Compared with other reports, our study reveals 9582 proteins, 3130 phosphorylation-modified proteins, and 1677 acetylation-modified proteins. The multiple-omics overview in rats needs more experiments to confirm the results and explore the underlying functions of the dysfunctional targets in AP and SAP.

## Materials & Methods

### Animal models

Male Sprague‒Dawley rats were used to establish AP and SAP models, and pancreatic tissues were collected for multiple-proteomics analysis. All animals were housed under pathogen-free conditions. The project was approved by The Second Xiangya Hospital, Central South University (Granted number, 2022521) and complied with the animal care and use guidelines.

To establish an AP model in rats, 150 g male rats were intraperitoneally administered 4-hour injections of caerulein (20 μg/kg), a cholecystokinin receptor analog. To establish the SAP model in rats, rats were anesthetized by isoflurane, and a midline abdominal incision was performed to expose the intestine. The rat pancreatic lobes were divided into four segments named the splenic, duodenal, gastric and parabiliary ducts. According to a previous report, we ligated the duodenal duct using 7-0 sutures, closed the abdominal wall, and allowed recovery for three days. Then, caerulein (20 μg/kg) was intraperitoneally injected once. After two days, blood and pancreatic samples were collected for further analysis.

### Histological analysis

Rat tissues (pancreas, lung, and intestine) were collected and immediately fixed in 4% paraformaldehyde for 24 hours. After paraffin embedding, the samples were cut into 4-μM slices. H&E staining of these tissues was performed after deparaffinization and dehydration of the slices. Images were captured by a Zeiss microscope. The histological score was graded in a blinded manner according to the standard described in a previous report^16^.

### Analysis of amylase and lipase activity in plasma

The blood sample supplemented with EDTA was centrifuged at 3000 rpm/min for 10 min to obtain plasma. The amylase and lipase activities in plasma were measured using a clinical analysis system. A kit based on the EPS substrate was used to assess amylase activity, and a kit based on methyl triazine substrate was used to assess lipase activity. The signal was measured using an AU1000 system (Beckman, America).

### Immunohistochemical staining (IHC)

Paraffin-embedded samples were cut into 4-μm slices, heated to 60 °C for 1 hour and dehydrated by rinsing in xylene and various concentrations of ethanol. IHC staining was conducted using an IHC kit (ZSGB-BIO, China) following the manufacturer's instructions. Briefly, antigen retrieval was performed with sodium citrate buffer, and samples were incubated in primary antibodies overnight at high humidity. Then, the slices were rinsed in PBS, and 3% H_2_O_2_ was added to reduce endogenous peroxidase activity for 20 min. After washing with PBS, the response enhancer provided by the kit was added, and the sample was incubated for 20 min. The sample was incubated in secondary antibody for 20 min at 37 °C. The slices were rinsed and incubated with diaminobenzidine working solution (20 x storage solution) for 3-6 min. Then, the slices were washed with ddH_2_O for 10 min, and the nuclei were stained with hematoxylin for 5 min. The slices were dehydrated and sealed with resinene.

### Sample preparation and LC‒MS/MS analysis for proteomics

Pancreatic tissues were ground individually in liquid nitrogen, lysed with lysis buffer, and ultrasonicated on ice for 5 min. The samples were centrifuged at 12000 × g for 15 min to remove the sediment. The supernatant was reduced with 10 mM DTT for 1 h followed by alkylation with IAM for 1 h in the dark. The supernatant was treated with 4 times the volume of precooled acetone and centrifuged at 12000 × g for 15 min to collect the precipitate.

Protein was quantified using the BSA assay, and the integrity was determined by electrophoresis. Then, each sample was mixed with DB dissolution buffer, trypsin and 100 nM TEAB buffer followed by overnight digestion. After centrifugation, the supernatant was filtered using a C18 desalting column, and the eluents were collected and lyophilized. The eluents were labeled with TMT reagent, desalted and lyophilized. Mobile phases A (2% acetonitrile, adjusted pH to 10.0 using ammonium hydroxide) and B (98% acetonitrile) were used to create a fraction solution, and 10 fractions were obtained for each sample. The separated samples were analyzed using an EASY-nLC^TM^ 1200 UHPLC system and a Q Exactive^TM^ HF-X mass spectrometer (Thermo Fisher), and the raw data were obtained and used for data analysis.

### Sample preparation and LC‒MS/MS analysis for phospho/acetylation proteomics

Pancreatic tissues were treated as described for proteomics, quantified using the BSA assay, and assessed by electrophoresis for integrity. The peptide preparation was treated with trypsin at 37 °C for 4 h. The eluents were lyophilized, dissolved in binding buffer, and loaded onto a pretreated IMAC-Fe column for phosphopeptide enrichment or anti-PTM-agarose for acetylated peptide enrichment. After washing several times, the eluate was collected, desalted and lyophilized. LC‒MS/MS analysis was performed using an EASY-nLC^TM^ 1200 UHPLC system and a Q Exactive^TM^ HF-X mass spectrometer (Thermo Fisher).

### Protein identification and quantification

The resulting spectral data were searched using Proteome Discoverer 2.4 (Thermo Fisher). Phosphorylated serine, threonine and tyrosine were specified as dynamic modifications, and acetylation was specified as an N-terminal modification. To identify proteins, peptide spectrum matches with a credibility of greater than 99% and an FDR of less than 1.0% were used as cutoffs. Gene symbols refer to GRCm39. The protein expression difference between the two groups was analyzed by T test.

### Bioinformatics and statistical analysis

Principal component analysis, coefficient of variance and unsupervised hierarchical clustering analysis were performed on the quantitative data. The ratio of the mean value of biological duplications was used for protein difference analysis. A volcano map and hierarchical clustering analysis of differentiated proteins are provided. Gene Ontology (GO) term enrichment analysis was conducted using the interproscan program against nonredundant protein databases (including Pfam, PRINTS, ProDom, SMART, ProSite, and PANTHER). Protein families and pathways were selected by Kyoto Encyclopedia of Genes and Genomes (KEGG) analysis or GSEA of KEGG and Reactome analyses. All data are expressed as the mean ± standard error using GraphPad Prism 8. The significance between 2 groups was analyzed by unpaired Student's t test using a parametric test or nonparametric test. A p value of < 0.05 was considered statistically significant.

## Results

### Construction of AP and SAP models in rats

**Figure 1:**
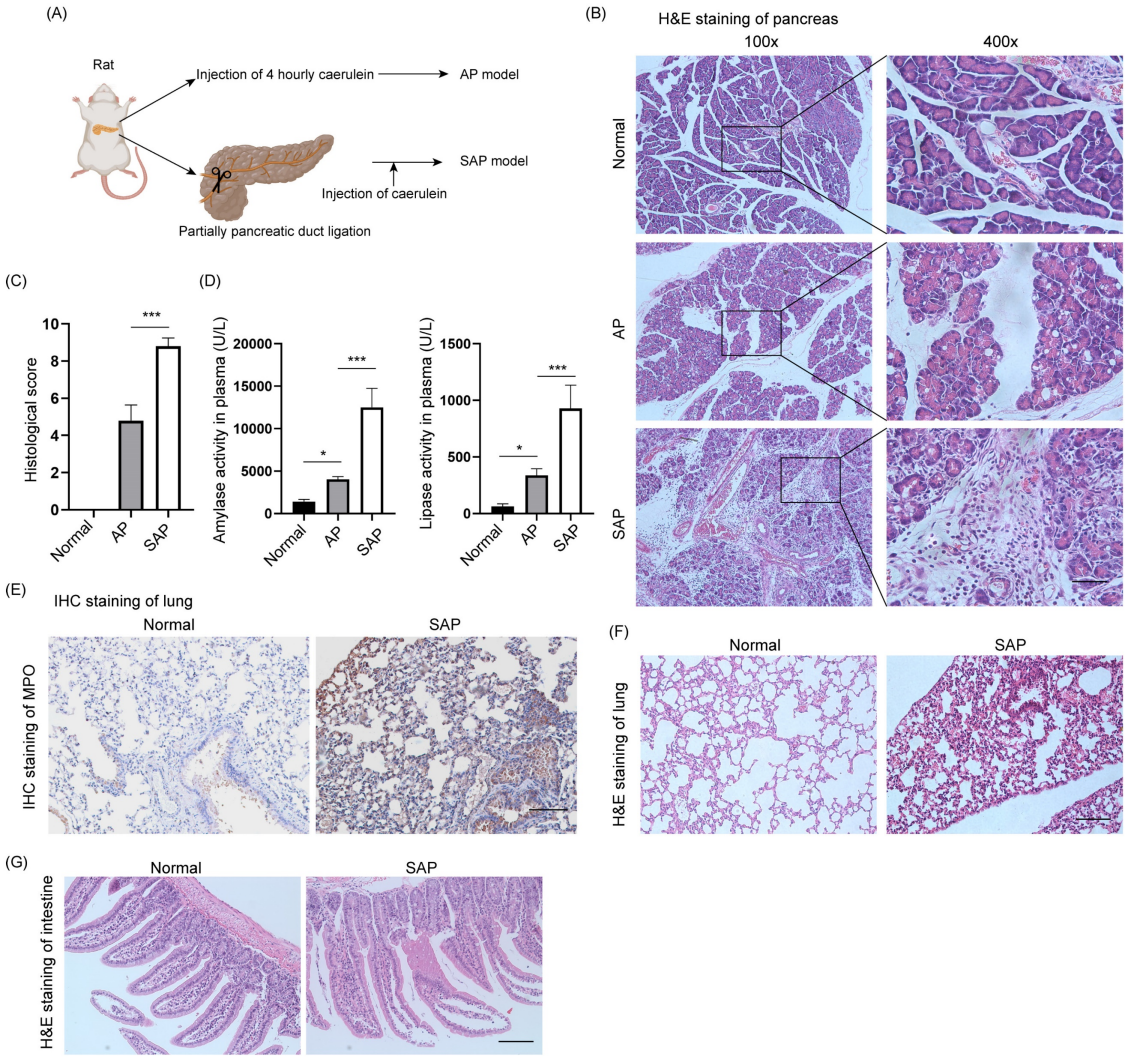
** Establishment of AP and SAP models in rats.** (A) Schematic of the methods used to establish AP and SAP models in rats. Caerulein (20 μg/kg) was intraperitoneally injected 4 times at one-hour intervals. The duodenal duct was ligated using 7-0 sutures. (B) H&E staining of the pancreas in the normal, AP and SAP groups. Scale bar, 100 μm. (C) Histological score of the pancreas in the normal, AP and SAP groups. ***p<0.001. (D) Amylase and lipase activity in plasma among the normal, AP and SAP groups. *p<0.05; ***p<0.001. (E) MPO is a marker of neutrophils. Representative image of IHC staining of MPO in lung tissues in the normal and SAP groups. Scale bar, 100 μm. (F-G) H&E staining of the lung and intestine in the normal and SAP groups. Scale bar, 100 μm. AP, acute pancreatitis; SAP, severe acute pancreatitis.

The AP model was induced by intraperitoneal injection of caerulein at 1-hour intervals 4 times, and pancreatic tissues and plasma were collected 1 hour after the last injection (Fig. 1A). The SAP model was constructed by ligation of the pancreatic duct and stimulated by caerulein after 3 days. H&E staining of the pancreas showed diffuse edema, inflammatory cell infiltration and cell death in AP, whereas diffuse parenchymal necrosis was observed in SAP (Fig. 1B). The histological score of the pancreas in SAP was significantly upregulated compared to that in AP (Fig. 1C). Consistently, the amylase and lipase activity in plasma further demonstrated that AP and SAP were successfully induced in rats (Fig. 1D). SAP was also characterized by secondary complications on remote organs, such as the lung and intestine. As expected, MPO, which is a biomarker of neutrophils, was obviously increased in the lung in the SAP model compared to the normal model (Fig. 1E). H&E staining of lung showed the alveolar septum, alveolar edema and inflammatory cell infiltration in lung tissues in SAP (Fig. 1F). H&E staining of intestine showed epithelial cell exfoliation and villus broadening in SAP (Fig. 1G). Thus, we successfully established AP and SAP models in rats, and pancreatic tissues were collected for multiple-proteomics analysis.

### Global profiling of protein expression changes in AP in rats

**Figure 2:**
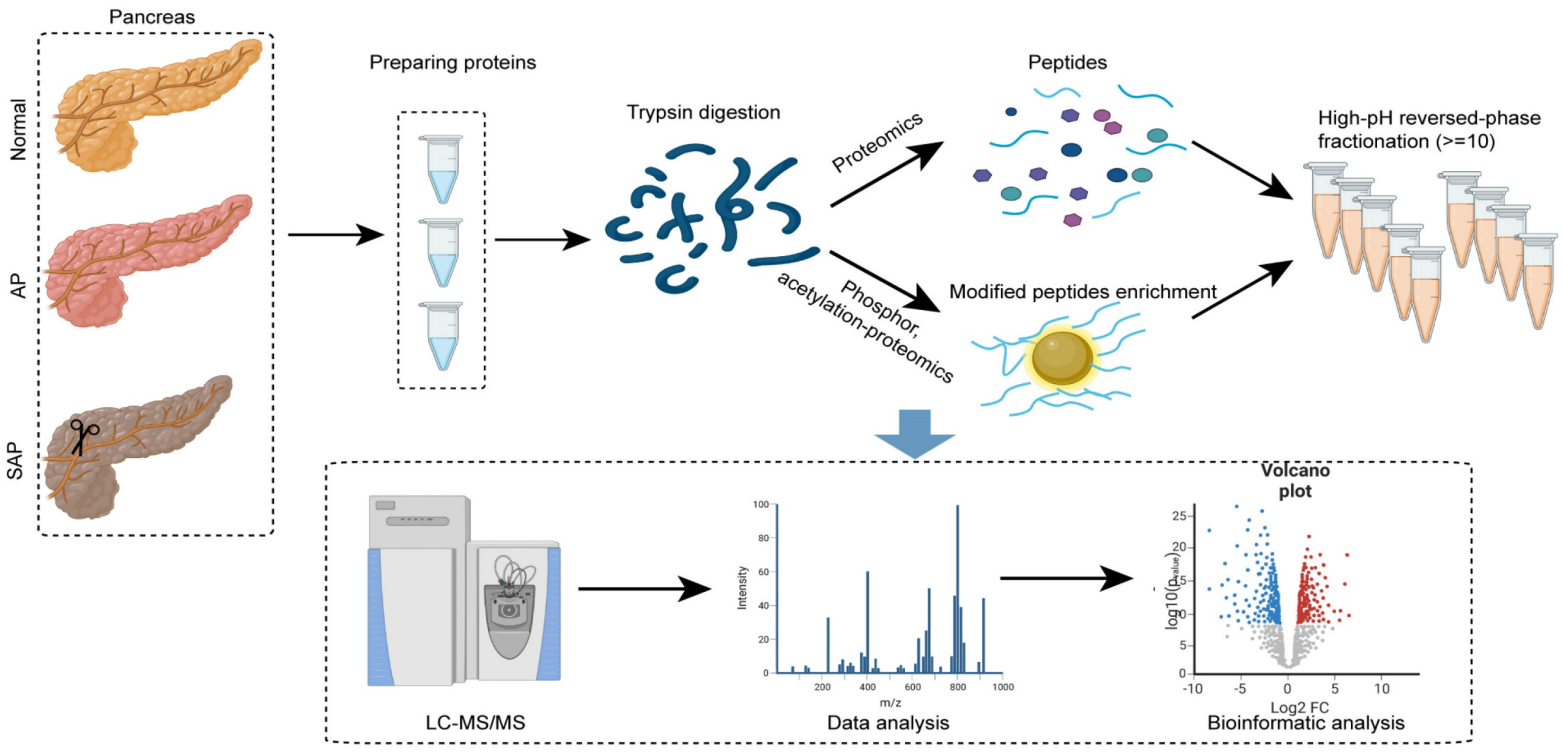
Experimental procedure and data analysis workflow of pancreas proteomics and phospho/acetylation proteomics.

**Figure 3:**
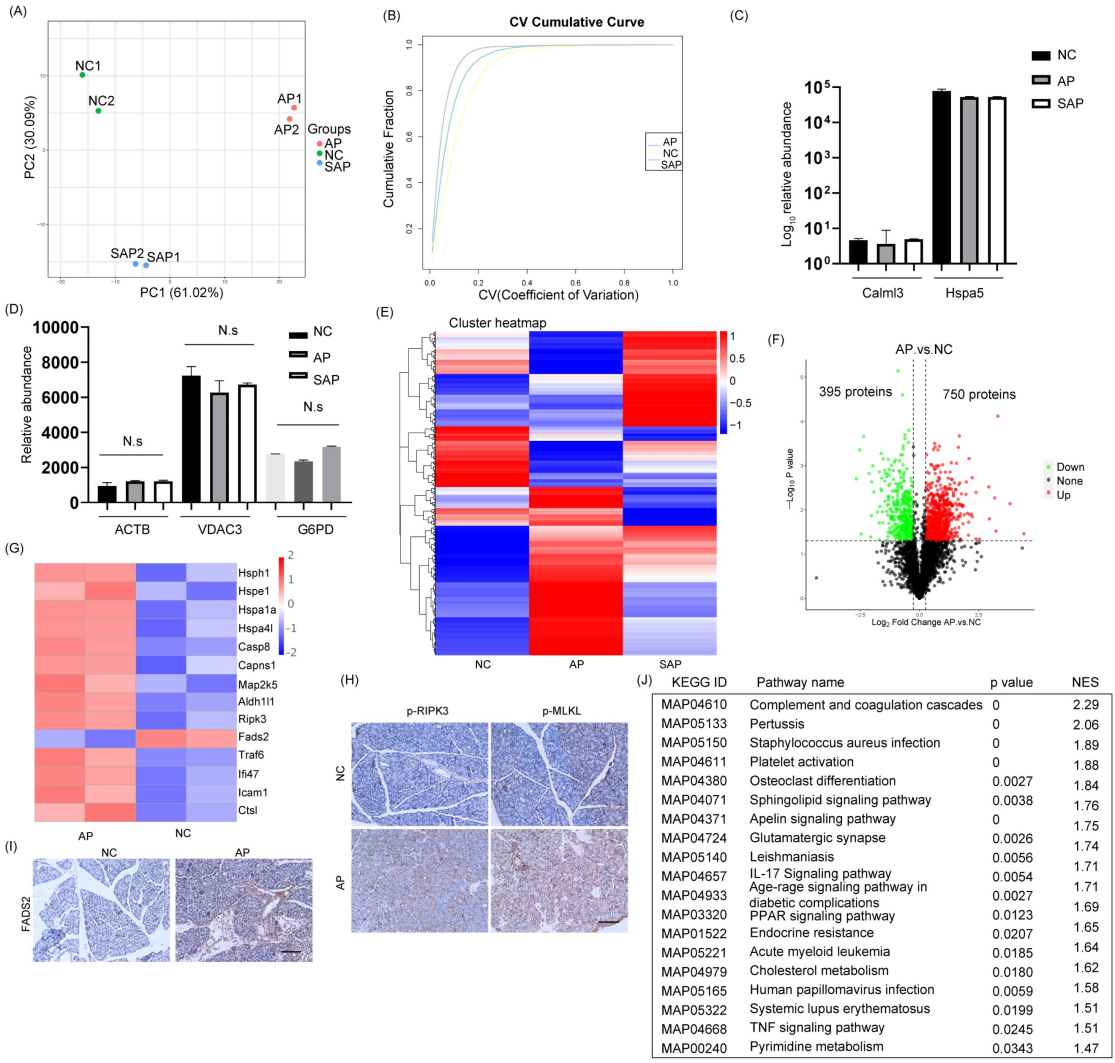
** Global profiling of protein expression changes in AP in rats.** (A) Principal component analysis (PCA) of the global pancreatic proteomics data. (B) Coefficient of variation of the normal, AP and SAP groups based on pancreatic proteomics. (C) Relative abundance of Calml3 and Hspa5 in the normal, AP and SAP groups. (D) Relative abundance of ACTB, VDAC3 and G6PD in the normal, AP and SAP groups. N.s, no significance. (E) Hierarchical clustering map of pancreatic tissues from the normal, AP and SAP groups. (F) Volcanic map of protein expression between AP and normal pancreas. The cutoff value was p<0.05. (G) Heatmap showing the expression of hsph1, hspe1, hspa1a, hspa4l, caspase 8, Capns1, Map2k5, Aldh1l1, Fads2, RIPK3, Caap1, Bclaf1, BIRC6, TRAF6, Ifi47, Ctsl, and Icam1 between the AP and normal groups. (H-I) IHC staining of p-MLKL, p-RIPK3, and FADS2 in pancreas in the AP and normal groups. (J) GSEA of KEGG gene sets between the AP and normal groups. NES, normalized enrichment score. NC, normal control.

To analyze the proteome profiling and protein modification profiling in AP and SAP, we performed TMT proteomics, and the workflow is shown in Fig. 2. We quantified 51327 peptides and 7221 proteins at a 1% peptide-level false discovery rate (FDR) across all samples. Principal component analysis (PCA) of the global data showed tight clustering of replicates from each group (Fig. 3A). The coefficient of variance (CV) showed high reproducibility in the quality control runs (Fig. 3B). The relative expression level among various proteins spanned more than 4 orders of magnitude (Fig. 3C). The abundance of housekeeping proteins, such as ACTB, VDAC3, and G6PD, remained largely unchanged among the groups (Fig. 3D). To achieve higher confidence, proteins identified in both replicates and in all groups were used for further statistical analyses, yielding 3302 proteins that were modified across all samples (Table S1).

The cluster heatmap showed a clear difference with several cluster in protein expression among the normal, AP and SAP groups (Fig. 3E, Table S2). When a cutoff of p < 0.05 was employed, 750 proteins were upregulated in the AP group compared with the normal group, whereas 395 proteins were downregulated (Fig. 3F). Upon initiation of AP, the small heat shock protein family, which functions as molecular chaperones^17^, was frequently increased. Several members of the family, such as hsph1, hspe1, hspa1a and hspa4l, were significantly upregulated in the AP group (Fig. 3G). The programmed cell death of acinar cells and inflammatory cells was frequently observed upon induction of AP. The proteins responsible for the induction of apoptosis, such as caspase 8, Capns1, Map2k5, and Aldh1l1, were significantly upregulated in the AP group compared to the normal group (Fig. 3G). Alternative forms of programmed cell death, including ferroptosis and necroptosis, were also observed in the AP group and exemplified by dysregulation of FADS2 and RIPK3, respectively (Fig. 3G). We further demonstrated the occurrence of ferroptosis and necroptosis by IHC staining (Fig. 3H-I). On the other hand, apoptosis inhibitors, such as Caap1, Bclaf1, and BIRC6, were also upregulated, indicating that an intense balance was necessary to determine the fate of acinar cells (Fig. 3G). In addition, the relative abundance of proteins responsible for the acute inflammatory response, such as TRAF6, Ifi47, Ctsl, and Icam1, was increased in the AP group compared to the normal (Fig. 3G). To gain insight into the high-level functions of the detected proteins among each group, Gene Set Enrichment Analysis (GSEA) was conducted. The signaling pathways involved in complement and coagulation cascades, platelet activation, the IL-17 signaling pathway, the TNF signaling pathway, systemic lupus erythematosus and metabolic regulation pathways (cholesterol metabolism, pyrimidine metabolism) were significantly enriched in the AP group compared to the control (Fig. 3J). Thus, the proteomic data collectively indicated that programmed cell death and the inflammatory response were induced in AP in rats.

### Global profiling of protein expression changes of SAP in rats

**Figure 4:**
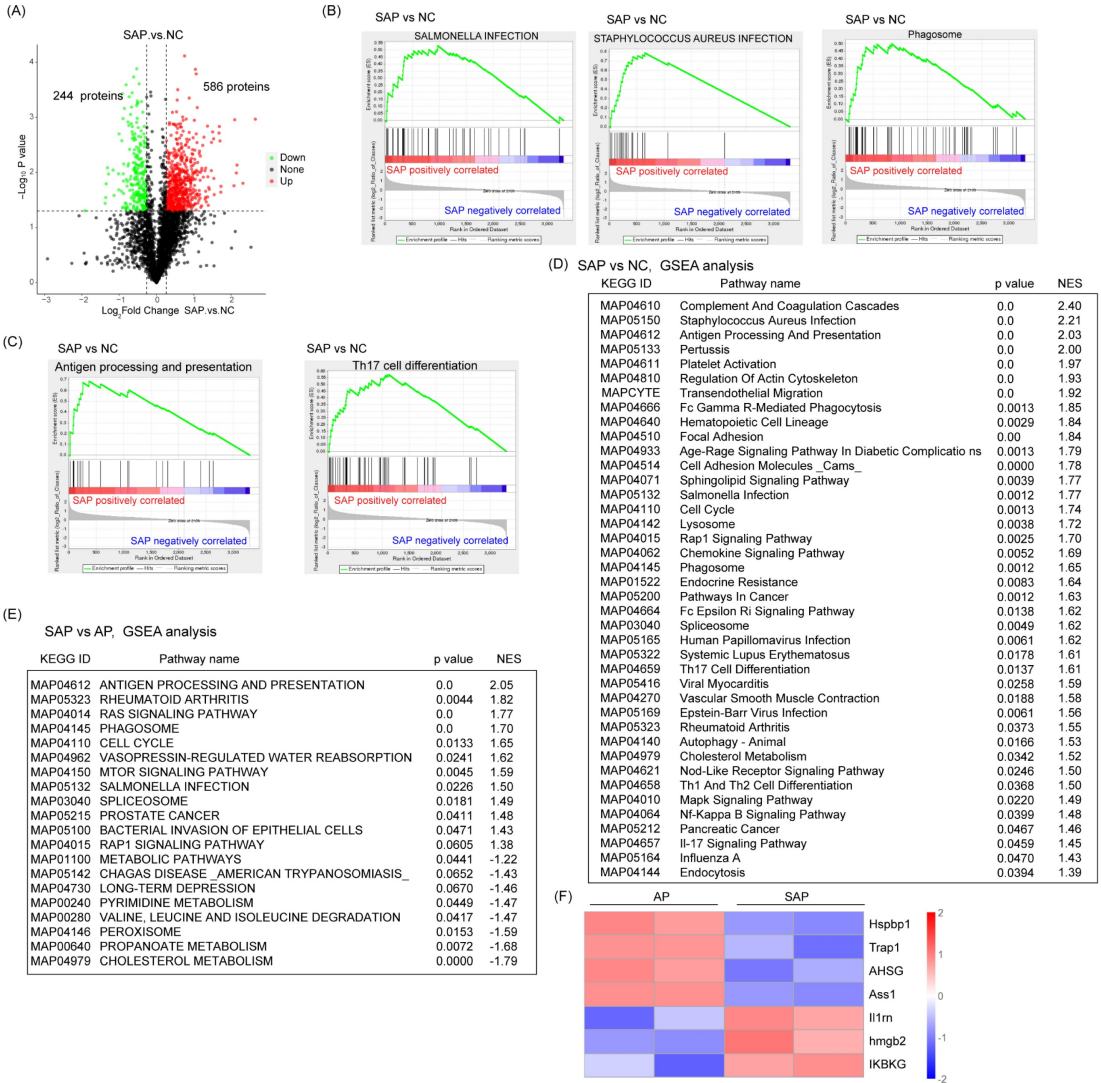
** Global profiling of protein expression changes in SAP in rats.** (A) Volcanic map of protein expression between SAP and normal pancreas. The cutoff value was p < 0.05. (B) GSEA showed enrichment in *Staphylococcus aureus* infection, *Salmonella* infection and phagosomes. (C) GSEA showed enrichment in antigen processing and presentation and Th17 cell differentiation. (D) Top 40 GSEA results comparing protein expression between SAP and normal pancreas. (E) Top 20 GSEA results comparing protein expression between SAP and AP. (F) Heatmap showing the expression of Hspbp1, Trap1, AHSG, Ass1, Il1m, Hmgb2, and IKBKG between SAP and AP. NC, normal control.

Next, we detected the protein variation between SAP and normal pancreas in rats. Compared to the normal, 586 proteins were upregulated and 244 proteins were downregulated in the SAP group by a cutoff of p < 0.05 (Fig. 4A). GSEA showed that in addition to proteins belonging to the innate inflammatory response (such as *Staphylococcus aureus* infection, *Salmonella* infection, and phagosome) and cell death-related pathways (such as autophagy), many signaling pathways belonging to the adaptive immune response (such as antigen processing and presentation, Th17 cell differentiation, Th1 and Th2 cell differentiation) were significantly enriched. These findings suggested an increase in the adaptive immune response in SAP (Fig. 4B-D). The prevalence of coagulation disorders (complement and coagulation cascades and platelet activation) causes disease progression. Several key metabolic pathways were also enriched, such as G-glutamine and D-glutamate metabolism, alpha-linolenic acid metabolism and pyrimidine metabolism, accompanied by DNA replication and the cell cycle pathway (Fig. 4D). These findings suggest pronounced metabolic stress, which might be derived from damaged acinar cells and immune cells.

Compared with the AP group, the SAP group showed negative enrichment in several metabolic pathways, such as cholesterol metabolism, propanoate metabolism, pyrimidine metabolism, and metabolic pathways, which might indicate an impairment of essential metabolism when AP progresses to SAP (Fig. 4E). On the other hand, many acute phase proteins, such as hspbp1, Trap1, and AHSG, were significantly downregulated in the SAP group compared with the AP group (Fig. 4F). These proteins often function as anti-injury factors^18^-^20^, suggesting an exhaustion stage of the defense system in the pancreas during SAP. Furthermore, an anti-inflammatory response occurs in parallel with a proinflammatory response. Considerably enhanced expression of anti-inflammation proteins, such as Il1rn, hmgb2, and IKBKG, was observed in the SAP group compared to the AP group (Fig. 4F). These data revealed a complicated anti-inflammatory response and proinflammatory response in SAP.

### Phospho-proteomics analysis of AP and SAP in rats

**Figure 5:**
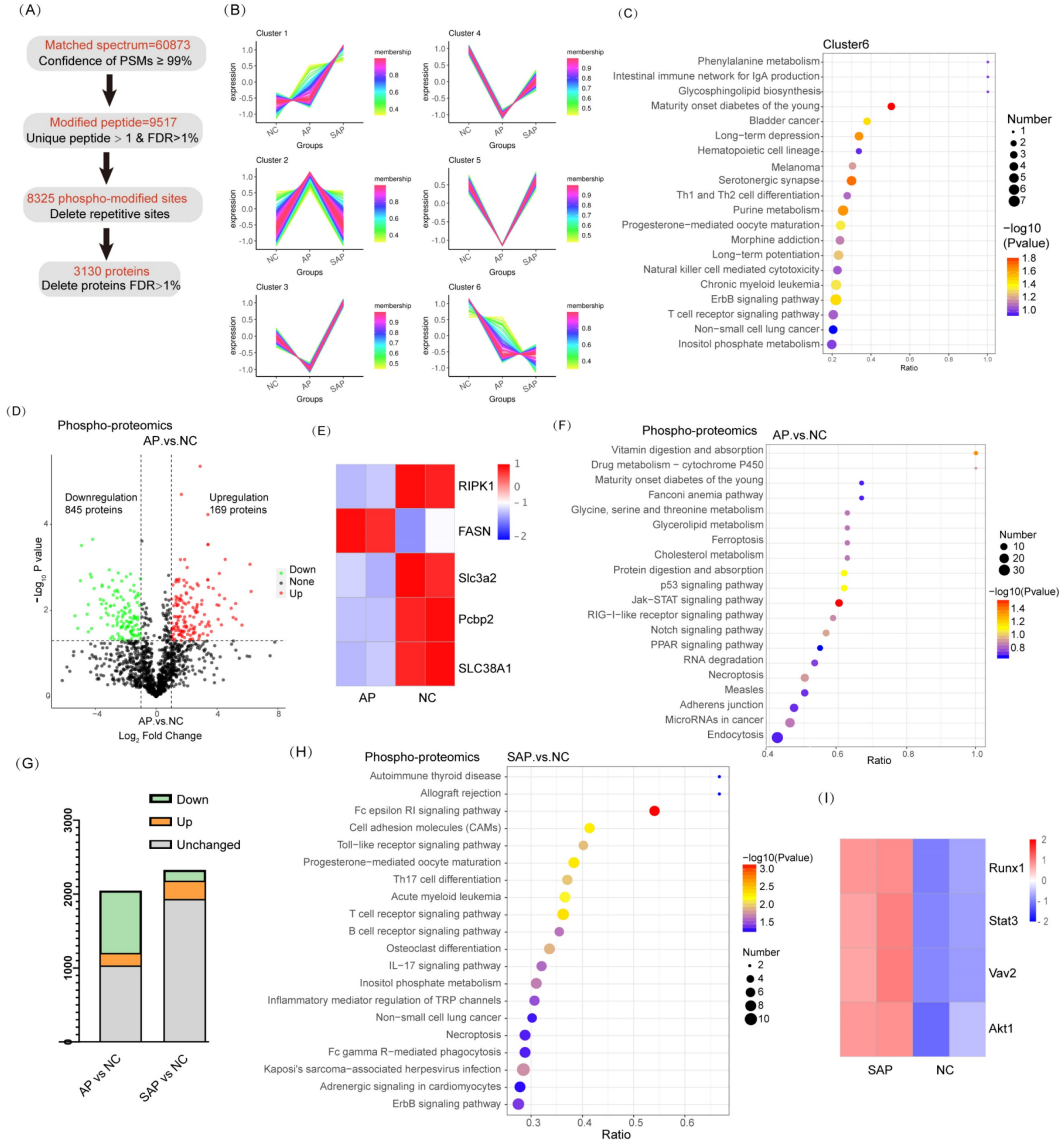
** Phosphoproteomic analysis of AP and SAP in rats.** (A) Data analysis process of phosphoproteomics in normal, AP and SAP pancreatic tissues. (B) Clustering analysis of phosphoproteomics revealing 6 clusters with distinct expression hallmarks. (C) KEGG pathway analysis of Cluster 6 of phosphoproteomics. (D) Phosphoprotein changes between AP and normal pancreas with a cutoff of p<0.05. (E) RIPK1, FASN, SLC3A2, Pcbp2, and SLC38A2 phosphorylation levels in AP and normal tissues. (F) KEGG analysis of differentially phosphorylated proteins between AP and normal tissues. (G) Differentially phosphorylated proteins in the comparison of AP and the control and the comparison of SAP and the control. (H) KEGG analysis of differentially phosphorylated proteins between the SAP and control groups. (I) Heatmap showing Runx1, Stat3, Vav2 and Akt1 expression in SAP and normal pancreas tissues. NC, normal control.

To understand the phosphopeptide profiles of AP and SAP in rats, we used phosphoproteomics analysis of pancreas in normal or pancreatitis. After filtering phosphopeptides for at least two valid values among two biological replicates in at least one condition, we identified 8325 phospho-modified sites in 3130 proteins (Fig. 5A). Clustering analysis revealed 6 clusters with distinct protein hallmarks (Fig. 5B, Table S3). Proteins in Cluster 6 were persistently dephosphorylated in the progression of AP to SAP, and KEGG pathway analysis of Cluster 6 showed enrichment of pathways involved in the development of the pancreas and adaptive immune response (Fig. 5C). Then, we used phosphorylated kinase analysis to reveal the variation in kinases in different samples or groups. The results showed that the AP group showed less kinase activity than the control, and SAP largely recovered (Fig. S1).

A cluster heatmap of phosphoprotein profiles showed a dramatic change between AP and the normal. Compared to the normal, 1014 proteins of the AP group significantly differed. Among these proteins, 169 proteins were upregulated, whereas 845 proteins were downregulated (Fig. 5D). Regarding factors implicated in cell death, Ripk1 and FASN phosphorylation activated necroptosis and were upregulated in AP compared to the control^21^, ^22^; fluctuations in Slc3a2, Pcbp2, and SLC38A1 phosphorylation suggested that ferroptosis was obviously induced in AP^23^-^25^ (Fig. 5E). KEGG pathway analysis revealed significant enrichment of ferroptosis, p53 signaling, and necroptosis in the AP group compared to the normal (Fig. 5F). The physiological function of pancreatic cells was disrupted in AP. In addition, the endocrine and exocrine systems were damaged, as evidenced by enrichment of signaling pathways, such as maturity onset diabetes of the young, protein digestion and absorption, and amino acid metabolism (Fig. 5F). These results were consistent with the data obtained from proteome profiling.

Although SAP tissues exhibited more cell death than AP, the phosphopeptide profiles of SAP did not reveal more proteins associated with cell death or greater upregulation of these proteins (Fig. 5G). Actually, at the beginning of AP, the regulation program of ferroptosis and necroptosis was already induced. KEGG pathway analysis revealed that innate immunity was significantly induced in SAP (enriched in cell adhesion molecules and the Toll-like receptor signaling pathway) (Fig. 5H). A pronounced adaptive immune response was also observed in SAP, as evidenced by enrichment in Th17 cell differentiation, T-cell receptor signaling, and B-cell receptor signaling (Fig. 5H). The phosphorylation levels of Runx1 and Stat3, which are key regulators of Th17 cell differentiation, were significantly increased in SAP compared to those in the control^26^, ^27^. Vav2 and Akt1, which are essential for B-cell proliferation and differentiation, were highly phosphorylated in SAP 28, 29 (Fig. 5I). Thus, SAP caused an extended adaptive immune response, as evidenced by phosphoproteomics analysis.

### Acetylation proteomics analysis of AP and SAP in rats

**Figure 6:**
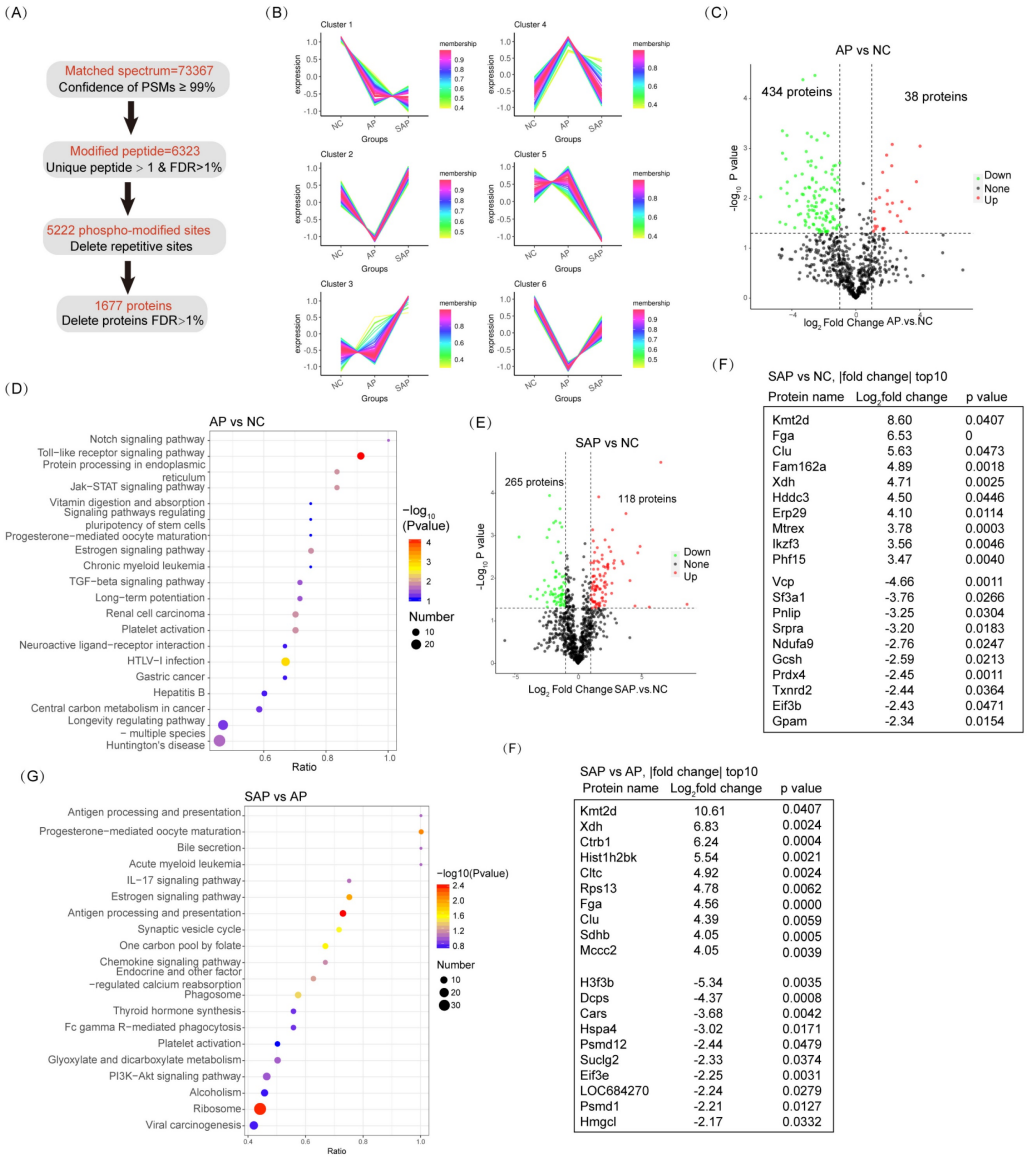
** Acetylation proteomic analysis of AP and SAP in rats.** (A) Data analysis process of acetylation-proteomics in normal, AP and SAP pancreatic tissues. (B) Clustering analysis of acetylation proteomics revealing 6 clusters with distinct expression hallmarks. (C) Acetylation protein changes between AP and normal pancreas with a cutoff of p<0.05 and |log_2_ fold change|>1. (D) KEGG analysis of differentially acetylated proteins between AP and normal tissues. (E) Acetylation protein changes between SAP and normal pancreas with a cutoff of p<0.05 and |log_2_ fold change|>1. (F) A list of the top 10 differentially acetylated proteins between SAP and normal pancreas tissues. (G) KEGG analysis of differentially acetylated proteins between SAP and AP. (H) A list of the top 10 differentially acetylated proteins between SAP and AP tissues. NC, normal control.

For acetylation proteomics, peptide spectrum matches (PSMs) with a confidence level of greater than 99% were identified as credible, and the proteins containing at least one unique peptide segment were selected. A total of 20430 acetylated sites were detected in 1677 proteins (Fig. 6A). Clustering analysis of acetylated proteins revealed 6 clusters with distinct expression hallmarks (Fig. 6B, Table S4). Compared to the normal, the overall acetylation level of the AP group was considerably reduced; only 38 proteins were hyperacetylated, and 434 proteins were hypoacetylated (Fig. 6C). KEGG pathway analysis revealed a significant enrichment of the innate immune response (Toll-like receptor signaling pathway, HTLV-I infection, and hepatitis B) in the AP group compared to the normal (Fig. 6D). Enrichment in the pathway of protein processing in the endoplasmic reticulum (ER) emphasized the essential role of ER stress in the pathological process of AP (Fig. 6D). On the other hand, in SAP, 118 proteins were hyperacetylated, and 265 proteins were hypoacetylated compared to the normal (Fig. 6E). Among the top 10 hypoacetylated and hyperacetylated proteins, Fga and Clu correlated with coagulation cascades; Fam162a and Mtrex were associated with programmed cell death; Xdh, Gcsh, Ndufa9, Gpam and Hddc3 were associated with cell metabolism; Prdx4 and Txnrd2 were associated with redox homoeostasis; Erp29 and Srpra were associated with ER stress; and Ikzf3, Vcp, and Eif3b were associated with inflammatory cell maturation or activity (Fig. 6F). Thus, acetylation-proteomics analysis emphasized the essential innate immune response, programmed cell death, and dysregulated metabolism in the pathology of AP and SAP.

Compared to AP, the SAP group exhibited pronounced enrichment in the adaptive immune response (antigen processing and presentation, Il-17 signaling pathway, antigen processing and presentation) (Fig. 6G). Among the top 10 dysregulated acetylated proteins, Fga and Clu are critical for coagulation cascades. These proteins exhibit sustained hyperacetylated in the progression from AP to SAP, suggesting their role in pathology (Fig. 6H). Several cell proliferation- or metabolism-associated proteins were also ranked in the top 10 dysregulated acetylation proteins, including Kmt2d, Xdh, H3f3b, Hmgcl, Ctrb1, Hist1h2bk, Sdhb, Mccc2, and Suclg2. Thus, these data revealed significant enrichment in the innate immune response and programmed cell death in AP and enrichment in the adaptive immune response, coagulation cascades and dysregulation of cell metabolism in SAP based on acetylation-proteomics analysis.

### Integrative analysis of the proteomics, phosphoproteomics and acetylation proteomics analysis

**Figure 7:**
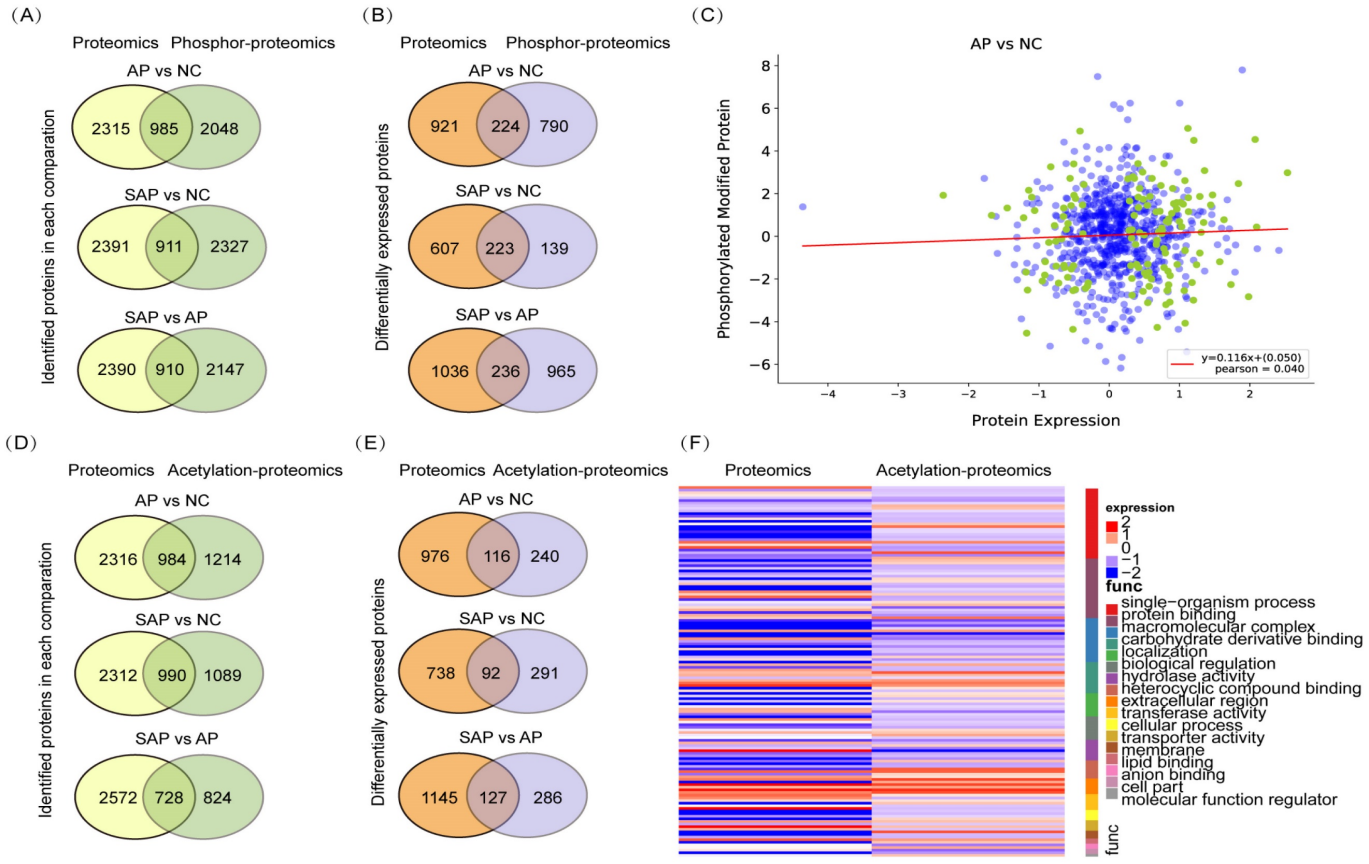
** Integrative analysis of the proteomics, phosphoproteomics and acetylation proteomics analyses.** (A) The coidentified proteins by proteomics and phospho-proteomics for each pair of comparisons. (B) The codifferentially expressed proteins identified by proteomics and phospho-proteomics for each pair of comparisons. (C) The correlation between protein expression and protein phosphorylation level between AP and normal pancreas. (D) The coidentified proteins by proteomics and acetylation proteomics, presented by each pair of comparisons. (E) The codifferentially expressed proteins identified by proteomics and acetylation proteomics for each pair of comparisons. (F) The correlation between protein expression and protein acetylation levels between AP and normal pancreas. NC, normal control.

To perform an integrative analysis of proteomics and phosphoproteomics, the proteins identified in both replicates of all groups and modified proteins identified in at least one group were used for further statistical analyses. A total of 985 proteins were jointly expressed in the proteomics and phosphoproteomics analyses of the comparison between AP and normal tissues, 911 proteins in the comparison between SAP and normal, and 910 proteins in the comparison between SAP and AP (Fig. 7A). Then, we screened the proteins that were jointly differentially expressed in proteomics and phosphoproteomics and identified 224 proteins in the comparison of AP and the normal, 223 proteins in the comparison of SAP and the normal and 236 proteins in the comparison of SAP and AP (Fig. 7B). The level of phosphorylation and the level of protein expression showed no correlation, indicating that phosphorylation did not affect the protein expression (Fig. 7C).

Regarding the integrative analysis of proteomics and acetylation-proteomics, 984 proteins were jointly expressed in proteomics and acetylation proteomics analyses in the comparison of AP and the normal group, 990 proteins in the comparison of SAP and the normal, and 728 proteins in the comparison of SAP and AP (Fig. 7D). Then, we screened the proteins that were jointly differentially expressed in proteomics and acetylation proteomics, revealing in 116 proteins in the comparison of AP and normal, 92 proteins in the comparison of SAP and normal, and 127 proteins in the comparison of SAP and AP (Fig. 7E). The level of acetylation and the level of protein expression also showed no correlation (Fig. 7F). These results suggested that a complicated multiple dimensionality network involving protein expression, phosphorylation modification, and acetylation modification existed in the pathological process of AP and SAP, and more detailed functional research of these data should be conducted to unveil the molecular mechanism underlying AP and SAP.

## Discussion

The proteomic, phospho-proteomics and acetylation proteomics analyses of the pancreatic tissues under normal conditions and in the context of pancreatitis in our work present a multiple dimensional profile that can be characterized and verified by experiments. We assessed 9582 proteins by proteomic analysis, 8325 phospho-modified sites in 3130 proteins, and 5222 acetyl-modified sites in 1677 proteins across all samples. The proteomic data revealed several prominent characteristics of AP. Many proteins responsible for acute phase response, programmed cell death, and acute inflammatory response are dysregulated compared to that noted in normal pancreas, whereas results also suggested a potential stalling of the anti-injury system and an activation of anti-inflammatory response of SAP compared to AP. Results from phosphoproteomics and acetylation proteomics analyses are highly consistent with the proteomic results regarding the comparison between AP and normal pancreas. However, but when SAP is compared to AP, the results emphasized the upregulation of the adaptive immune response (enriched in Th17 cell differentiation, T-cell receptor signaling, and B-cell receptor signaling). KEGG pathway analysis suggested a pronounced enrichment of innate and adaptive immune responses in SAP compared to AP based on acetylation-proteomics analysis. Thus, our integrative proteomic and protein modification-omics (phosphoproteomics and acetylation proteomics) analyses correctly revealed the key characteristics of AP and SAP, and SAP showed a potential exhaustion of the anti-injury response, activation of the anti-inflammatory response, and enhancement of the adaptive immune response compared to AP.

Programmed cell death in acinar cells, including apoptosis, ferroptosis, and necroptosis is essential for the severity of AP^10^, ^30^, ^31^. Many studies have sought to identify potential targets or drugs to alleviate AP using specific inhibitors or genetic knockout of cell death regulators. Our multiomics analysis found several proteins essential for programmed cell death that were dysregulated in AP. Inhibition of apoptosis by the caspase inhibitor z-VAD-fmk vinpocetine alleviated acinar cell death and decreased the proinflammatory response in AP. A similar result was also found in the inhibition of necroptosis and ferroptosis^15^. The interaction among these programmed cell deaths seems complicated during AP. A previous study showed that inhibition of necroptosis by knockout of RIPK3 or MLKL increased pancreatic edema, recruitment of inflammatory cells and apoptosis, which could be explained by the notion that RIP3 also plays an essential role in regulating apoptosis and other cellular functions^32^. Similar to our analysis, these three types of programmed cell death coexisted in AP, and future studies need to further elucidate the interaction among cell death types and provide a more detailed therapeutic strategy based on the inhibition of cell death.

Although several acute phase proteins, such as hsph1, hspe1, haptoglobin, and clusterin, were significantly increased in AP compared to normal samples in our analysis, none of them ranked among the top 50 upregulated proteins. This finding is obviously different from a previous report that showed that 7 out of the top 10 upregulated proteins belong to acute phase proteins^11^. This difference could be explained by the different time points of sample collection (12 hours vs. 2 hours after the first injection of caerulein) as these time points represent fully developed AP and early stage AP, respectively. The top 10 upregulated proteins in our analysis can be clustered into metabolic pathway (Nfs1, Dnah5, Emap2, Apoa2, Supv3l1), inhibition of proteinases (Mug2), inflammatory response (Cd44, Tbc1d2b), programmed cell death (ALDH1L1) and secretory pathway (Unc13a). In contrast, many acute phase proteins, such as hspbp1, Trap1, AHSG, and Ass1, are downregulated in SAP compared to AP. Overexpression of acute phase proteins, such as Hspb1, could suppress pancreatic amylase and lipase activity and acinar cell damage and preserve the actin cytoskeleton in mice^33^. AAT defects might determine the severity of AP in patients^13^. Thus, reactivating the blunt anti-injury response by acute phase proteins represents a beneficial strategy for the treatment of SAP.

A distinct difference in the inflammatory response occurs between AP and SAP. Specifically, the proinflammatory hyperinflammatory phase (SIRS) at the initiation of AP is followed by a hypoinflammatory phase (named CARS) in SAP, and both of these phases determine the disease severity^34^, ^35^. Our proteomics and phosphoproteomics results were consistent with this viewpoint. Concomitantly, AP only produces a sterile inflammatory response. However, when the disease progresses to SAP, secondary infection or damage-associated molecular patterns occur, leading to an adaptive inflammatory response. We observed a significant enrichment of signaling pathways involved in the adaptive inflammatory response, such as Th17 cell differentiation, T-cell receptor signaling and B-cell receptor signaling, in SAP. Th17 cells are increased in AP^36^. Conversely, another study showed that the Th1/Th17 axis is not activated at the early stage of SAP, and patients with SAP exhibit a diminished Th1/Th17 response^37^. A balance between the anti-inflammatory type-2 immune response and proinflammation might start from the early stage of SAP. Thus, time-resolved multiomics involving local and systemic immune cells would provide more details on the dynamics of the immune response during SAP.

## Conclusions

In summary, the multiomics data in this work offer a valuable resource to understand the proteomic and protein modification atlas in AP and SAP. However, further studies need to confirm the multiomics data and explore the underlying functions of the dysfunctional targets in AP and SAP.

## Supplementary Material

Supplementary figure 1.

Supplementary table 1.

Supplementary table 2.

Supplementary table 3.

Supplementary table 4.
