# Supplementary figures and images for: Integrative proteomics, phosphoproteomics and acetylation proteomics analyses of acute pancreatitis in rats
Source: Int J Med Sci. 2023 May 11;20(7):888–900. doi: 10.7150/ijms.81658 (PMC10266050; doi:10.7150/ijms.81658)

# Kinase substrate

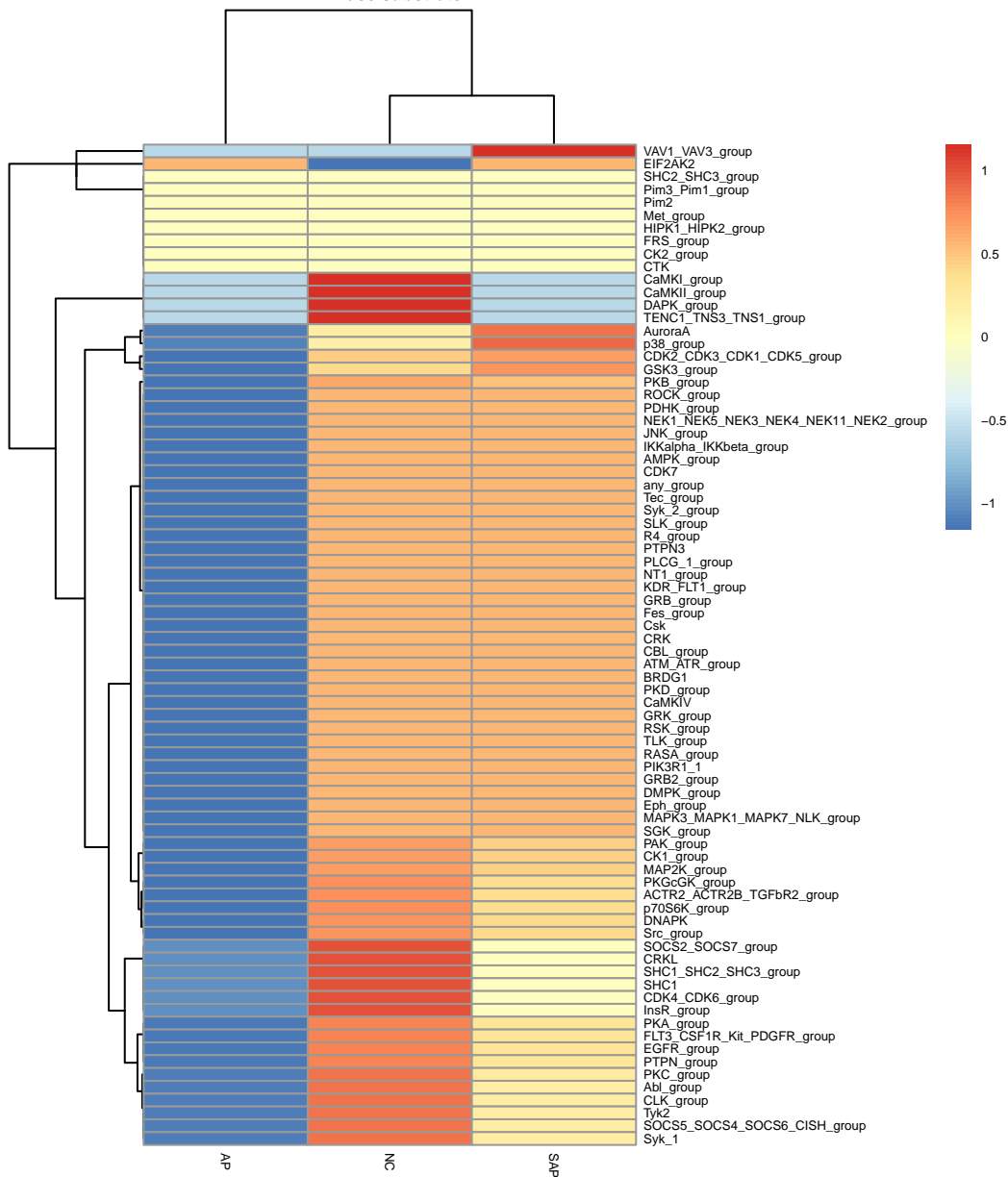

1 **Fig. S1 Phosphorylated kinase analysis**

2

Supplement: Supplementary file 1 — Supplementary figure 1. [file ijmsv20p0888s1.pdf]
