# Supplementary material for: Integrative proteomics, phosphoproteomics and acetylation proteomics analyses of acute pancreatitis in rats
Source: Int J Med Sci. 2023 May 11;20(7):888–900. doi: 10.7150/ijms.81658 (PMC10266050; doi:10.7150/ijms.81658)
Supplement: Supplementary file 2 — Supplementary table 1. [file ijmsv20p0888s2.pdf]

Protein	Gene	NC1	NC2	AP1	AP2	SAP1	SAP2
A0A023IM54	Tapbp	314.9	479.7	219.5	223.9	835.8	805.4
A0A023IMI6	Psmb8	220.2	221.7	374.7	407.1	328	342.2
A0A059NZR0	Jpt1	605.8	431	272.8	265.2	295.5	360.9
A0A068F1Y2	Actb	818.6	1083.4	1643	1700.5	1193.6	1254.4
A0A068FP44	CARM1	113.2	155.5	98.4	98.4	213.4	214.2
A0A096MIV5	Abcf2	1255.8	1275.6	1069.6	1416.9	1280.2	1217.9
A0A096MIX2	Ddx17	3596.9	3658.9	4551.2	4584.8	3812.1	4039.6
A0A096MJ39	Cd36	1932.5	2279.5	2009.2	1906.9	2559.1	2763.8
A0A096MJP9	Ddi2	411	471.1	348.1	374.5	648.3	624.7
A0A096MJZ2	Tbl2	2984.4	3345.6	2845.1	3067.9	2699.1	2758.2
A0A096MK30	Msn	4466.5	3899.3	4533.6	4641.4	6504.1	6755.2
A0A096MKE0	Asph	1220.8	1289.8	1601.3	1685.4	1445.8	1391.8
A0A096MKE5	Zfp326	189.5	218.8	151.3	156.7	266.9	244.9
A0A096MKE9	Lig3	553	606.1	887.4	825	540.7	501.6
A0A096MKG2	Nol6	197.8	247	428.7	445.3	306.5	256
A0A096MKH2	Vta1	459.8	687.5	616.9	681.9	658.7	641.8
A0A096P6L8	Fn1	7528.5	8063.4	12695.8	13581.3	14569.3	16041.1
A0A097BW25	Postn	364.8	342.3	303.7	329	541.7	487.4
A0A0A0MXV4	Ace	134.2	150.3	252.5	242.1	200.7	169.1
A0A0A0MXW1	Bckdhb	1952.1	2032	2021.3	2069.7	1413.2	1173.2
A0A0A0MXY5	Pfkp	366.1	358.6	382	386.1	511.5	595.9
A0A0A0MY25	Rnase1	13796	10834.6	8825.7	7704.4	4830.4	4650.7
A0A0A0MY43	Ascc3	308.6	319.7	210.3	197.2	310.9	310.5
A0A0F7RQJ6	Ddt	3325.4	3591	2315.2	2586.6	3015.8	3091.8
A0A0G2JSG6	Ak2	5782.8	5602.3	5688.6	5196.5	4411.9	4040.7
A0A0G2JSH9	Prdx2	330.4	420.5	235.8	312.5	599.4	532.6
A0A0G2JSI1	Aldh9a1	12594.3	14475.4	9196.3	9452.4	12691.6	13087.7
A0A0G2JSI5	Cela1	4933	4019.5	2769.3	2678.7	1860.5	1628.5
A0A0G2JSK5	Itgb1	3364.7	3264.5	3985.4	5784.5	3404.8	3486
A0A0G2JSL8	Adk	3826.3	4144.7	3312.5	3020.6	3551.2	3401.5
A0A0G2JSM3	Pdlim3	27.2	28.7	24.5	15.1	29.5	27.1
A0A0G2JSM4	Bet1l	296.7	570.9	359.1	532	569.7	584.2
A0A0G2JSM7	Add1	2493.5	2579.3	2458.7	2659.4	2514.7	2541.5
A0A0G2JSQ4	Tpm1	653.5	495.8	390.9	424.2	670.3	761.4
A0A0G2JSQ7	Klk1b3	3084.9	3026.8	5691.6	4180.6	1981	1919
A0A0G2JSR0	Vdac3	6875.9	7605.1	5813.3	6748.5	6664.2	6796.1
A0A0G2JSR7	Matr3	1510.7	1744.1	2141.7	1920.8	2319.5	2315.2
A0A0G2JSR9	Sdf4	291	461.1	417.7	424	358.3	335.2
A0A0G2JSS9	Atl3	1171.7	1557.8	1648.7	1602.3	2301.1	2153.4
A0A0G2JSU3	Set	3340.5	3615.2	3784.4	4138.1	5052	4492.4
A0A0G2JSV2	Cbr1	2380.8	2539.7	2168.3	2186.6	3079.6	2886.3
A0A0G2JSV3	LOC314140	519.1	794.2	906.9	961	1042.4	966.4
A0A0G2JSW3	Hbb	15195.6	17002.9	19096.2	18543.8	18303.4	17377.2
A0A0G2JSY2	Cast	3991.8	4004	4268.5	3986	3194.2	3054.2
A0A0G2JSZ5	Pdia6	7082.3	5253	7298.8	6766.6	4441.5	4188.2

A0A0G2JSZ9	Ccs	188	247.4	322.7	463.4	224.7	196.8
A0A0G2JT25	Dcxr	1037.7	1067.8	997.3	1058.4	943.7	890.6
A0A0G2JT30	Mat2b	755.5	881.1	744.1	749.5	815.3	820.6
A0A0G2JT33	Tep1	141.1	179.1	173.5	187.3	238.3	216.1
A0A0G2JTA0	Faf2	440.2	535.4	599	668	640.5	640.5
A0A0G2JTA1	Ppp2r5e	271.3	211.9	197.5	186.5	181.5	189
A0A0G2JTG7	Hnrnph1	1446.8	1022.7	895	843.7	890.8	947.7
A0A0G2JTI7	Prpf3	501.6	517.5	382	334.2	418.8	430.7
A0A0G2JTK4	Ppp6r1	326.7	441.1	581.6	955.2	487.6	455.4
A0A0G2JTK6	Mta3	55.9	58.2	59.3	68.8	75.7	65.2
A0A0G2JTL5	Pc	6121.3	5092.8	5417.1	5299.5	4019.1	3891.6
A0A0G2JTP6	Plin1	2878.8	2162.8	2552.1	2106.7	1824.1	1878.6
A0A0G2JTQ5	Galk2	288.9	315.3	469	374.7	284.2	283.8
A0A0G2JTV2	Cald1	2859.1	3421.3	3903.5	3785.2	4693.6	4854.7
A0A0G2JTX5	Dpp4	1381.5	1602.1	1168.5	1240.7	1789.1	1876.5
A0A0G2JTX7	Col6a5	267.1	304.9	427.8	377.7	305.1	325.7
A0A0G2JU01	Arhgef1	227.9	274.4	437.9	397.8	417.4	377.5
A0A0G2JU07	Ube2v2	1079.1	416	320.4	320.7	249.4	248.1
A0A0G2JU12	Mgst2	382.7	450.6	569.2	567.8	519.8	531.7
A0A0G2JU15	Ptcd3	503.3	601.7	609.1	593.2	577.4	567.1
A0A0G2JU77	Eif3k	472.1	515.9	480.2	537.8	584.2	561.4
A0A0G2JU79	Rcbtb1	121.5	136.5	236.2	235	141.6	138.8
A0A0G2JU82	Macf1	1611.7	1878.9	2788.1	2914.4	2177.7	2109.7
A0A0G2JU83		274.3	342.7	193.9	160	225.8	216.4
A0A0G2JUA5	Ahnak	33574.7	35302.1	51316.4	49175.4	50497.7	52091.9
A0A0G2JUB0	Fam3c	134.3	143.7	148.8	138	150.8	136.1
A0A0G2JUC7	Dctn2	1765	1911.3	1866.8	2266.3	1722.9	1605.5
A0A0G2JUE4	Golga4	2492.6	3058	4188.7	4220.4	3320.4	3265.2
A0A0G2JUJ9	Sec63	5804.8	6443.7	5409.8	5601.6	5417.3	5228.6
A0A0G2JUM8	Coa6	360.9	394.1	694.9	700.2	365.7	359.1
A0A0G2JUY4		280	235.3	547.7	598.8	275.2	260.6
A0A0G2JV16	Dock11	187.9	273.1	286.8	341.9	471.2	433.6
A0A0G2JV46	Fam107b	118	117.7	74.6	74.6	67.8	66.2
A0A0G2JV54	Ptbp3	25.7	25.3	21.3	26.8	41.5	31.2
A0A0G2JVD0	Rbm28	212.3	235.6	177.6	175.3	185.9	189.1
A0A0G2JVG3	Pkm	540.2	440.8	847.1	594	490	497.1
A0A0G2JVG4	Pecr	4144	3687.9	4012	4152.6	2607.6	2587
A0A0G2JVH4	Immt	4673.6	4944.4	4304.3	4138.7	4033.1	3873.3
A0A0G2JVM0	Aldh4a1	1641.6	1769.9	1415.2	1439.7	1446.8	1556.9
A0A0G2JVM2	Mia3	5606.9	6755.4	5138.6	4487	5305.6	5330.9
A0A0G2JVP1	Vrk2	152.2	157.1	233.7	220.7	190.6	167.9
A0A0G2JVU1	Itgb8	182	233.9	226.8	269.4	374.9	316.8
A0A0G2JVW5	Huwe1	603.5	737.1	980.6	1122	935.4	888.8
A0A0G2JW28	Add3	702.4	588.4	943	985.1	1224.2	1088.2
A0A0G2JW29	Man1a1	1904.1	2114.1	3301	3230.3	2041.7	1862.5
A0A0G2JW52	Prmt3	119.8	146.7	192	236.6	204.7	206.8

A0A0G2JW85	Rasa3	81.9	91.2	76	88.9	153	149
A0A0G2JW94	Clint1	2721.3	2486.4	1603.8	1472.3	2595.2	2506.6
A0A0G2JWF2		143.4	175.7	607.2	448.1	253.5	207.1
A0A0G2JWK2	Mecp2	223.7	221	250.2	244.7	248.5	229.9
A0A0G2JWK7	Tagln	9064.6	7284.9	8042.5	7693.6	10876.1	9927.8
A0A0G2JWM2	Sirt2	470.1	502	594	541.3	498.7	498.4
A0A0G2JWP1	Cstf2	640.1	731.4	990.6	923.8	669.6	727.3
A0A0G2JWQ7	C1galt1	160.4	211.2	288.4	257.6	205.9	205.2
A0A0G2JWR1	Trmt11	148.1	151	85.4	112.5	150.9	151.3
A0A0G2JWR5	LOC103694865	204.1	229.8	166.6	198.2	343.4	283.6
A0A0G2JWU9	Aamp	292.9	374.6	388.9	411.1	393.2	357.1
A0A0G2JWV2	Fam162a	115.9	146.3	101.2	83.5	135	120.2
A0A0G2JX25	Gmpr2	68.6	66.5	107.3	125.8	82.7	74.6
A0A0G2JX30	Atad2b	305	342.8	513.4	574.3	469.7	465.5
A0A0G2JX56	Dnajc5	428.4	513.7	705.9	665.4	471.7	437.1
A0A0G2JX72	Mbnl1	120.9	80.9	30.2	44.3	74.1	82.8
A0A0G2JXC3	Rps21	3677.4	2597.1	2131.2	2193	1848.8	1799.5
A0A0G2JXG7	Carmil1	56.9	71	124.1	111.3	151.7	143.7
A0A0G2JXJ7	Esyt1	1227.7	1303.9	1420.7	1274.5	1261.9	1328.7
A0A0G2JXN8	Osbpl8	562.3	629.8	577	684.3	839	840.5
A0A0G2JXP1	Gyg1	1022.4	916.7	811.6	739.3	1025.9	1094.9
A0A0G2JXT6	Mtmr6	371	454.9	595.3	531	415.5	431.8
A0A0G2JXT8	Flnb	12217	12008.5	14022.4	14407.7	16357.1	16241.2
A0A0G2JXW4	Hnrnpc	4592.1	6034.1	5619.9	5667.2	7955.6	7004.6
A0A0G2JY07	Mcm5	122.9	117.2	164.3	152.9	158.1	164.1
A0A0G2JY08	Myo18a	375.4	450	786.9	685.1	597.3	549.1
A0A0G2JY69	Pex19	1117.7	1241.5	2084.5	2512.5	1481	1416.9
A0A0G2JY73	Eif4g3	74.4	82.2	146.9	132.4	84	84.5
A0A0G2JY82	Scamp1	293.1	317	384.5	396.2	307.6	280
A0A0G2JYC6	Ubap2l	670.1	582.4	503.8	538.2	553.5	574.3
A0A0G2JYE0	Atxn2l	228.7	247.1	121.1	101.2	183.9	172
A0A0G2JYG5	Myo9b	206.4	252.2	224.8	272.6	375.7	397.2
A0A0G2JYI0	Lrba	1094.3	1213	2029.1	2087.4	1229.5	1200.2
A0A0G2JYI7	Ubap2	1091.3	1112.7	956.3	840.9	1130.1	1219
A0A0G2JYJ1	Grb10	178.4	186.5	99.8	92.2	259.1	292.9
A0A0G2JYN0	Dcaf8	135.9	168.1	147.9	188.7	206.7	190
A0A0G2JYN4	Ergic2	1170.7	1371.2	1734.7	1537.1	946.3	881.8
A0A0G2JYR1	Exoc6b	503.6	631.1	794.9	750.9	727.5	709.8
A0A0G2JYT1	Erc1	237.5	242	227.3	276.3	332	308.1
A0A0G2JYU6	Fmnl1	120.4	171.1	240.8	239.4	291.8	312.3
A0A0G2JYW3	Clta	1538.5	1491.6	1535.2	1262.6	1656.8	1717.6
A0A0G2JZ53	Birc6	182.6	237.9	445.4	463.2	316	245.6
A0A0G2JZ73	Serpina1	5919.8	7207.6	8546.5	8466.4	11054.3	12237.3
A0A0G2JZ75	Plvap	723.9	680.7	996.8	956.4	771.1	711.5
A0A0G2JZ79	Sirt1	43	59.2	35.7	36.8	90.6	85.7
A0A0G2JZ83	Agap3	211.3	250.1	518.9	498.6	294	284.8

A0A0G2JZF0	Sec24c	2122.5	2299.4	2711.9	3074.5	2263	2137.6
A0A0G2JZF6	Ndufaf2	299.1	351.3	572.7	513.9	345.3	348.1
A0A0G2JZH0	Cab39	260	391.8	531.3	649.7	1113.2	808.9
A0A0G2JZH8	Pdhx	2150.1	2513.3	2291.2	2373.5	2125.8	2129.5
A0A0G2JZH9	Dhx57	48.8	39.3	22.7	22.1	37.7	43.2
A0A0G2JZI2	Eprs	15335.5	15223.7	14344.5	12895.7	12996.5	12973.9
A0A0G2JZM2	Sec23a	1254.4	1196.1	1550.7	1580.1	1704.8	1639.3
A0A0G2JZM8	Clasp2	366.8	336.6	319.1	356.8	294.4	274.3
A0A0G2JZQ1	Ddx19b	1871.9	1968.3	1560.3	1537.1	1715.6	1772.2
A0A0G2JZY0	Lsm14b	112.7	127.1	98.7	117.7	95	88.6
A0A0G2JZY3	Reps1	79.6	84.9	125.7	164.8	100.7	120.8
A0A0G2K013	Actn4	6241.4	6389.5	8957.8	8662.6	9387	9000.7
A0A0G2K016	Sun1	309.1	365.4	348.8	327.8	390.5	390.5
A0A0G2K022	Rcn3	920.8	915.3	989.3	1064.2	1843.4	1995.1
A0A0G2K038	Rexo2	2236.9	2240.6	1673.9	1766.6	1635	1729.5
A0A0G2K047	Acss3	407.2	434.1	508.8	480.1	434.3	404.4
A0A0G2K051	Eea1	2546.1	2747.2	4063.5	3975.4	3466.9	3295.3
A0A0G2K059	Mcu	204.2	234.3	350.7	332.8	418.2	409.8
A0A0G2K075	Ssr1	3662.9	3731.8	4308.6	4542.4	2983.6	2832.5
A0A0G2K0D8	Aldh1l1	125.5	208.3	4203.3	2913.2	300	213.8
A0A0G2K0I1	Spata5	63.4	75	125.4	134	91.6	80.9
A0A0G2K0I3	Nampt	733.7	854.8	1885.1	1540.1	1267	1225.8
A0A0G2K0L0	Papss1	487.9	510	1236.2	840.3	629.9	680.4
A0A0G2K0Q7	Mylk	1104.9	1209.3	1691.7	1853.8	1511.9	1468.3
A0A0G2K0T6	Sncg	1571	1379.8	1083.8	1035.6	1161.3	1282.2
A0A0G2K0T8	Fam25a	431.2	306.2	145.6	131	101.6	93.7
A0A0G2K0U8	Pus10	73.3	104.4	208.5	174.7	151	131.3
A0A0G2K0V3	Bpnt1	413.8	447.1	272.8	284.9	394.9	402.5
A0A0G2K0V8	LOC100911422	4167	4654.2	6531.8	6242.2	6075.2	5983.8
A0A0G2K0W0	Ggps1	178	201.8	341	346.7	227.3	252.3
A0A0G2K0Y2	Igf2bp3	301	271.8	134.6	117.1	290.9	300.7
A0A0G2K0Z9	Nop14	106.4	102.5	113.8	159.6	122.7	109.8
A0A0G2K101	Luzp1	126.1	150.3	212.4	216.1	220.6	233.4
A0A0G2K110	Eif6	834	688.1	1055.4	1103.5	754	756.6
A0A0G2K121	Mlec	6309	8292.2	5455.1	5778.6	6253.6	6192.1
A0A0G2K125	Acsm1	597.4	481	356.2	381.3	493.1	423.3
A0A0G2K135	Cfi	629.8	789.8	1796.1	1617.6	1017.7	1061.3
A0A0G2K160	Ahctf1	415.6	524.2	1248.4	1000.6	533.9	532.9
A0A0G2K161	Epb41	616.4	650	786.5	745.1	711.5	678.2
A0A0G2K1A1	Zfp706	1501.9	1186.4	1653	1737.5	1216	1061
A0A0G2K1C0	Actr3	8750.5	8922.2	9881.8	10109.9	10863.6	11134.2
A0A0G2K1F2	Acacb	120.1	160.9	156.1	186.9	196.9	169.2
A0A0G2K1F3	Copg1	5046.1	4959.1	5045.9	4485.6	4061.2	4001.7
A0A0G2K1L8	Basp1	167.9	167.4	300.5	289.5	347.8	343.7
A0A0G2K1M5	Dipk2a	85.7	79	49.9	58.8	78.5	78.5
A0A0G2K1M7	Ano6	386.6	509.4	480.2	479.7	452.8	430.9

A0A0G2K1N3	Hmbs	1300.2	1449.7	2199.4	2194.6	1429.3	1355.3
A0A0G2K1N9	Selenoo	663.6	719.2	642.5	722	662.2	604.8
A0A0G2K1Q1	Nlrx1	574.7	741.3	641.8	586.1	696.9	657.4
A0A0G2K1Q7	Ank3	824.4	934.5	1031.6	1004.3	873.9	795.1
A0A0G2K1S6	Me1	1251.2	898.5	830.6	791.4	1047.2	985.4
A0A0G2K1T0	Gls	126.1	141	610	329.8	229	204.4
A0A0G2K1U8	Ctrc	5492.6	6097.9	2373.8	2623.4	2891.2	2837.9
A0A0G2K1W1	Rab11fip5	309.9	379.5	765.6	617.9	532.7	594.8
A0A0G2K1Z9	Hm13	5495.8	5417.1	6879.3	7410.1	5120.7	4512.8
A0A0G2K248	Mat1a	1581.4	1125.2	1824.9	1619.4	1134.9	1022.7
A0A0G2K261	Iars2	1679.2	2004.4	2582.8	2564.7	2080.8	2015.4
A0A0G2K266	Ufl1	4732.3	4064.5	3668.2	3337.9	2924.4	2918.1
A0A0G2K2A5	Uchl5	1380.2	1428.8	1356.6	1294.6	1323.4	1342.6
A0A0G2K2F6		79.6	78	35.9	51.1	51	53.1
A0A0G2K2J9	Mia2	3844.6	4204.4	4087	3979.4	3842.2	3772.7
A0A0G2K2L1	Podxl	836.5	818.7	618.7	654.1	1068	1006.6
A0A0G2K2L8	LOC100910506	739.1	839.7	688	663.8	940.5	1140.6
A0A0G2K2M9	Srrm2	1027.3	888.9	760.8	771.7	804.7	741.9
A0A0G2K2P4	Cyp2t1	1100.2	1269.8	1891.8	1656.8	1265.8	1171
A0A0G2K2P5	Tjp1	2342.7	2228.2	1734.1	1616.7	2616.7	2653.7
A0A0G2K2P6	Hbs1l	2114.6	1856.9	2034.1	2021.6	1771.6	1669.6
A0A0G2K2Q2	Gcat	12315.2	12690.4	5621.1	5393.7	6705.4	6729.5
A0A0G2K2R4	Sytl1	686.3	514.7	453.7	511.9	414.8	434.2
A0A0G2K2T6	Ilf3	1889.6	1862.1	1265.8	1936.3	2283.9	2178.7
A0A0G2K2V7	Yes1	1045.2	1137	1306.3	1768.1	1200.5	1207
A0A0G2K2Y3	Erap1	1103.8	1251	1272.4	1538.1	1793.8	1774.8
A0A0G2K2Z0	Eml4	781.4	849.5	776.6	722.2	935.5	858.6
A0A0G2K320	Siglec1	544	533	626.5	564	688.4	838.7
A0A0G2K350	Napg	938.9	840.5	1206.7	1374.9	858	859
A0A0G2K3C0	Sbno1	126.6	192	208.5	264.4	277.3	267.7
A0A0G2K3G0	Hrg	2903.2	2406.9	3163.9	3333.3	3379.1	3337.2
A0A0G2K3I9	Dusp3	432.5	418.5	209.4	209.3	405.7	473.2
A0A0G2K3K7	Mknk1	599	550.6	529.2	528.4	463	462.6
A0A0G2K3L8	Wdfy1	886.6	1004.9	1498.3	1400.2	1723.7	1545
A0A0G2K3N1	Gbf1	167.5	226.9	331.4	399.6	235.6	239
A0A0G2K3S6	Rbm10	364.5	437	1049.2	845.3	569.7	538.4
A0A0G2K3V4	Ogt	415.6	435	681.2	594.6	586.6	591.4
A0A0G2K3V7	Top1	1163.9	1213	991.3	935.4	1350.8	1337.5
A0A0G2K3W1	Vwa8	90.6	121.1	117.2	137.2	111.4	108.9
A0A0G2K3Z4	Ces1d	3678.2	2522.8	1527.4	1681.3	2003.6	1793.8
A0A0G2K3Z9		11036.5	10574.1	6754.7	7528	10251.4	10616.3
A0A0G2K401	Pcca	10819.3	10153	6781.5	7075	6121.8	6368.4
A0A0G2K415	Trim26	25.8	32.8	33	42.1	34.9	39.6
A0A0G2K4F6	Srrm1	1044.1	1560.2	1953.6	2164.2	2326.1	2378.5
A0A0G2K4H6	Galns	75.6	90.6	88.1	100.2	108.5	102
A0A0G2K4K3	Abcc3	104.3	163.6	244.9	228.6	163.4	163.8

A0A0G2K4R5	Pop1	221.1	273.7	310.8	350.9	313.2	340.4
A0A0G2K4T3	Acap2	117.6	147.8	223.4	233.6	196	177
A0A0G2K4T7	Gtf2i	34.5	70.3	107.1	156.7	126.2	123.4
A0A0G2K4X8	Skp1	2418.9	2734.4	2821.3	2876.3	3185.2	3475.1
A0A0G2K528	Stx16	56.7	100.4	201.7	203.4	220.2	191.7
A0A0G2K531	Gpx3	756.1	227.8	476.1	826.4	195.6	200.4
A0A0G2K542	Ugp2	1517.3	1675.5	2969.4	2485.8	2322	2327.3
A0A0G2K550	B3galnt2	112.6	114.2	131.4	151.7	95.9	104.1
A0A0G2K568	Crym	82.8	95	128.2	144.3	111.6	99
A0A0G2K5A0	Exosc8	357	389	591.4	511.9	503.6	496.5
A0A0G2K5M1	Sp3	246.5	405.7	421.8	268.4	492.7	531.3
A0A0G2K5Y1	Vipas39	177.9	189.4	447.5	334	255.5	230.8
A0A0G2K613		196.5	230.1	531.7	448.5	368.5	361.5
A0A0G2K648	Wdr61	1686.6	1722.2	1286.1	1242.5	1676.2	1738.3
A0A0G2K654	Hist1h1c	6702	7604.4	8169.7	8223.8	9565.3	9215.4
A0A0G2K695	Myof	1060.2	1174.5	2378.4	2222.2	1844	1775
A0A0G2K6I4	Enah	568.1	492.5	168	141.5	431.1	599.2
A0A0G2K6R2		181.1	211.3	219.5	218.9	343.3	366.8
A0A0G2K6S9	Myh11	633.2	649.3	588	576.3	915.4	1049
A0A0G2K6T9	Pcdh1	97.9	131.5	188.8	441.7	151.7	167.5
A0A0G2K6U1	Nsf	5857.5	6570	8534.1	8287	6954.4	6680.7
A0A0G2K714	Sh3glb1	1019.2	948.5	684.1	613.9	985.5	1085
A0A0G2K719	Ddx3x	344.9	304.8	106.4	97.4	177.6	253.6
A0A0G2K737	Txnl1	2532.5	3091	3039	2702.1	3094.2	2982.5
A0A0G2K739	Ube2d3	727.8	797.4	823.3	807.1	769.6	801.8
A0A0G2K751	Dnajc8	778	744.3	542	537.8	944.9	852.8
A0A0G2K776		483.2	551.9	719.2	741.5	992.6	994.8
A0A0G2K781	Ppfibp1	63.6	70.2	53.1	47.5	116.3	111.7
A0A0G2K797	Klk1c6	846.4	1038.7	549.3	450.1	665.9	676.2
A0A0G2K7D7	Nars2	473.8	554	1056.8	855.8	646.5	598
A0A0G2K7H9	Mark1	276.9	335	362.8	381.5	390	377
A0A0G2K7M2	Rad23a	1352.6	1563.2	971.4	907.1	1608.1	1529.3
A0A0G2K7N7		150.8	148	160	159	136.4	132.2
A0A0G2K7N8	Bptf	60.8	67.2	116	109.5	126.5	100.5
A0A0G2K7T6	Nup155	635.2	724.1	1096.1	1064	962.9	976.1
A0A0G2K7W6	RGD1562402	6899.8	5321.9	3253.9	2923.6	3382.1	4534.8
A0A0G2K7W8	Hint3	143.8	108.8	77.6	62	89.4	84.7
A0A0G2K7X3	Nucks1	2204.4	2194.4	1509.8	1355	2276.6	1858.2
A0A0G2K7X7	C7	1071.6	1008	996.6	1109.5	1304.5	1330.9
A0A0G2K845	Adipoq	1072.5	988.1	795.2	922.8	1134.4	1001
A0A0G2K896	RGD1310507	888.9	892.1	1163	1281.9	1279.9	1350.4
A0A0G2K8A9		613	642.2	444	416.1	509.2	527.6
A0A0G2K8B7	Eif4a2	39.4	39.6	125.2	121.9	37.2	27.6
A0A0G2K8H0	Caprin1	3968.4	4019.3	5949.5	6696	4604.7	4823.2
A0A0G2K8K3	Serpind1	291	415.7	802.3	729	619.5	620.5
A0A0G2K8P2	Tnpo3	83.5	101.3	98.4	101.6	111.6	124.6

A0A0G2K8P3	Nedd4	1474.6	1614.2	1363.7	1193.6	1833.2	1744.6
A0A0G2K8Q1	Apoc3	226.2	186.6	313.6	513.1	136.9	155.9
A0A0G2K8S9	Art3	231.9	312.4	373.3	348.1	299.2	250.8
A0A0G2K8T0	Asah1	199.3	216.4	281.8	229.8	304.2	306.5
A0A0G2K8V2	Vcl	7851	7585.8	8650.2	8437.3	8685.1	9435.6
A0A0G2K8Z9	Kif13b	1194.5	1151	1901.6	2198.7	1023.1	1029.5
A0A0G2K905	Arpc2	3603.4	4084.7	4356.4	4613	5585	5777.8
A0A0G2K933	Eif4e2	124.6	122.8	149.2	126	120.6	120.7
A0A0G2K950	Papss2	78.3	111.2	188.6	168.4	151.3	155.8
A0A0G2K9B4	Mrpl15	45.3	56.9	146.7	169.6	72.9	60.1
A0A0G2K9C0	Vasp	677.7	602.4	542.5	687.7	737.5	756
A0A0G2K9D6	Smarcc1	123.6	129.8	228.2	203.6	141.1	137.8
A0A0G2K9F2		130.9	207.1	199.8	315.3	271.7	258.3
A0A0G2K9M5	Tbc1d8b	931.7	964.7	918.1	895.4	825.5	805.4
A0A0G2K9P4	Sh3glb2	224.3	279.2	293	280.7	297.5	283.8
A0A0G2K9P5	Cog5	733.2	598.5	905.3	804.2	471.3	436.3
A0A0G2K9Q1	Golph3l	1130	1450.4	2386.2	2112.5	1518.2	1390.5
A0A0G2K9R9	Psme4	504.3	348.1	375.1	425.4	331.7	326.5
A0A0G2K9S4	Myo5a	192.3	251.1	423.7	368.6	419.8	424.2
A0A0G2K9T1	Itch	197.5	241.1	155.9	140.6	228.1	222
A0A0G2K9V6	Tars	7651.8	7370.4	6452.1	6945.1	5997.8	5819.3
A0A0G2KA71	Nectin2	314.4	366.1	567.6	515.1	308.9	289.1
A0A0G2KAE1	Lims2	383.5	463.4	564.4	562.2	658.3	591.2
A0A0G2KAI8	Drg2	647.8	753.4	1353	1254	960.5	841.4
A0A0G2KAJ7	Col12a1	31.9	44.5	123.8	99	57.5	61.3
A0A0G2KAM4	Fscn1	1495.7	1560.8	2196.2	2340.1	2518.3	2506.1
A0A0G2KAN1	NEWGENE_621351	1722.8	2044.9	2550.7	2584	3304.4	3161.9
A0A0G2KAN2	Vps33a	575.3	190.3	130.9	131.2	174.1	164.2
A0A0G2KAN5	Erh	1600.7	1799.8	4010.6	3402.5	2525.1	2714.1
A0A0G2KAP1	Ero1b	4843.9	4868.5	3192.5	3145.8	3693.9	3536.7
A0A0G2KAW7	Eif4h	1015.9	912.9	629.4	631.4	826	706.7
A0A0G2KAZ7	Hnrnpdl	292.6	349.6	304.4	300.8	531.2	476.6
A0A0G2KB55	LOC102553386	2424.7	2526.8	3006	2915.8	3041.2	2853.3
A0A0G2KB61	Ppp1r13b	213.4	260.9	157.9	213.7	244.7	259.9
A0A0G2KB63	Phb2	3123	3286.5	2478	2737.9	2415.3	2386.2
A0A0G2KBC4	Csf1r	171.5	193.9	503.3	357.6	398.2	416.4
A0A0G2QC02	Skiv2l	1266.9	1369.5	1154.1	1223.6	1200.9	1267.7
A0A0G2QC04	Pls1	1968.1	2537.1	3068.9	2288.8	2988.1	3095.3
A0A0G2QC06	Tf	6785.3	7365.5	4634.8	4562.1	5732.5	5648.5
A0A0G2QC11	Krt86	679.8	701.8	1122.7	1306.1	3325.5	703
A0A0G2QC21	Arhgef7	234.8	262.9	943.3	682.5	342.3	334.7
A0A0G2QC24	Lrrc40	100.7	116	186.5	180.9	180.2	141.2
A0A0G2QC33	Atg4b	349.4	408.7	482	549.9	517.6	483.8
A0A0G2QC53	Eps15l1	191.7	240	286.1	288.3	326.6	318.8
A0A0H2UHA3	Lrrc8d	351.3	366.8	455	488.1	370.8	369.5
A0A0H2UHD9	Ncln	851	198.8	418	381.3	150.4	130.2

A0A0H2UHE1	Suclg1	2880.1	3235.7	2945.1	2774.5	2389.8	2253.7
A0A0H2UHE5	Ppm1a	293.8	371.4	609.3	541.9	346.4	375.2
A0A0H2UHF6	Arl6ip5	359.3	407.2	309.5	366	326.2	383.6
A0A0H2UHG0	Yars	10233.6	12826.9	13529.2	13316.3	11236.9	11158.4
A0A0H2UHG7	Rps20	5000.2	4100.4	3898.9	4227.9	3493.8	3391.6
A0A0H2UHH9	Rps24	2054.4	2590.1	1919.2	1782.1	2175.2	2044.6
A0A0H2UHJ1	S100a9	329.8	377.6	1063	1588.4	1415.7	2685.6
A0A0H2UHL3	Aebp1	370.5	431.9	910.1	763.6	696.7	644.3
A0A0H2UHL6	Ctsh	700.6	661.2	535.4	572.7	750.6	647.5
A0A0H2UHL9	Dbn1	188.8	159.2	124.5	146.5	220.2	286.9
A0A0H2UHM5	Pdia3	24995.7	23675.8	23506.2	22704.1	22351.1	22527.6
A0A0H2UHP0	Ctcf	93.2	94.2	78.1	74	199.1	183
A0A0H2UHP9	Rab6a	972.1	1080.3	2131	1728.2	1240.4	1175.6
A0A0H2UHR1	Nupr1l1	8.1	11.6	11.2	8.7	8.9	4.4
A0A0H2UHT6	Rps18	10499.2	7745.4	5839.4	8494.4	4768.2	4875
A0A0H2UHT9	Cmss1	131.1	170.7	382.5	267.4	246.3	210.5
A0A0H2UHV6	Ppp3r1	1469	1587	2407.9	2428.2	1726.4	1627.9
A0A0H2UHV9	Copg2	1533.6	1842.7	2122.3	1921.8	2014.1	2108.8
A0A0H2UHX0	Trpm4	136.8	147.3	165.5	159.2	227.8	170.5
A0A0H2UHZ2	Nap1l4	300.1	361.7	342.9	345.3	564.4	569.7
A0A0H2UHZ4	Zranb2	446.6	412	288.2	265	325.7	311.4
A0A0H2UHZ6	Puf60	1210.1	1331.1	1615.5	1595.8	1553.6	1554.2
A0A0H2UI10	Oga	38.7	41.5	41.9	54.1	53.3	54.4
A0A0H2UI27	Sept10	144.4	134.3	328	348.9	141.6	130.6
A0A0H2UI38	Rps19l2	28.7	34.8	14.6	12.3	18	23.5
A0A0U1RRP9	Cfb	3679.5	4431.6	7873.8	7056.3	6816.8	7246.8
A0A0U1RRQ7	Sec61a2	39.6	47.4	34.8	41.7	49.7	57.9
A0A0U1RRY3	Eif2b1	332.6	400.2	352	435.6	448	457
A0A140TA89	Gatm	21535.8	27494.1	19441.8	18185	16024.5	15089.5
A0A140TAA4	Pdcd6ip	8897.8	10291.3	9501	9348.2	9948.4	10319
A0A140TAC3	Epn1	264.3	318.5	197.1	202.6	591.1	459.6
A0A140TAD1	Nudt5	955.9	949.4	786.7	722	864.8	831.5
A0A140TAE1	Fads2	52.7	49	30	25.8	81.6	81.4
A0A140TAE6	Mecr	397.5	724.7	883.5	645.1	500.1	577.6
A0A140TAF0	Tpm3	145.5	154.4	187.5	157.8	232.2	242.4
A0A140TAF7	Poglut1	241.2	371.4	842.8	834.6	592.4	578.3
A0A140TAG9	Cdc37	927.8	887.2	1188.6	1059.8	1015	970.9
A0A140TAH1	Hgs	314	232.2	293	274.7	224.3	221.1
A0A140UHX0	Prkcd	430.1	485.3	629.7	591	797.3	746.7
A0A140UHY2	Cdk16	345.4	350.9	323.6	338.6	380.6	349.5
A0A140UHY3	Ewsr1	927.3	1025.5	759.9	614.5	1129.1	1293.5
A0A142BM04		280.3	537.8	10558.4	6050.9	503.7	416.3
A0A1B0GWQ7	Tsn	869.6	878.2	827	871.9	1499.5	1347.7
A0A1B0GWS5	C5	213.1	266	427.3	445.9	420.5	392
A0A1K0FUA6	LOC689064	5824.9	9199.7	9929	9045.7	7482.5	6807.8
A0A1L1WKE8		798.4	847.7	1336.9	1164	989.2	1034.7

A0A1W2Q5Z6	Tax1bp3	544.2	671.2	1282.5	1175.3	1187.3	1112.8
A0A1W2Q680	Gar1	1514.1	1678.1	1435.4	1689.2	1755.6	1765.1
A0A387KC71	Akr1c15	470	476	269.4	233.4	477.4	514.7
A0A3B0J380	Adic	321.4	390.1	793.6	702.8	529.9	603.5
A0A3G2LW22	Fxr1	650.8	679.4	701.8	704.4	764.1	771.1
A0A4X0W8E9	Abr	167.8	185.2	186.3	187.9	253.7	283.4
A0A5H1ZRV3	Tppp	171.8	198.1	205.1	257.8	185.9	165.7
A0A5P8DHK3	Syne2	592.6	744	1131.9	1108.3	986.8	931.9
A0A6F8P9J1	Lamp1	1567	1923.6	2994.6	2895.3	3074.6	2620.7
A0JN17	Kras	110.9	144.3	119	126.6	160.6	159.9
A0JN29	Lnpk	519	587.9	1476.8	1154.4	565.7	526.7
A0JN30	Cnpy2	5457	4634.3	5387.1	5332.3	3413.6	3376.4
A0JPJ7	Ola1	8423.5	9375.9	7022.5	6205.6	7122.2	7091.8
A0JPM9	Eif3j	2605.5	2692.9	3184.5	2718	2381.9	2236
A0JPN6	Med22	79.1	114	182.7	182.5	117.5	115.5
A1A5L1	Blmh	1452.6	1641.1	1062.5	1325.2	2192.1	2167.4
A1A5L2	Pgm1	2596.7	2940.6	2944.4	3049	3937.1	3714.6
A1A5P2	Rrs1	624.4	658	688.7	706.9	702.8	771.5
A1A5Q1	Parp9	127	137.9	143.7	140.4	208.1	207.6
A1A5R3	Krr1	613	680.3	794.2	869.6	753.5	723.3
A1A5S1	Prpf6	266.5	370.9	467.8	541.1	474.7	480.1
A1L108	Arpc5l	86	113	176.9	210.5	314.8	231
A1L134	Aup1	96.6	143.4	151.8	193.2	125.9	116.4
A1L1J8	Rab5b	1392.4	1189	679.1	637.2	1134.6	900.7
A1L1L2	Tmem214	5373.7	7295.2	7839.2	8150.5	7594.6	6778
A1L1L5	Ccnk	27.9	34.9	19.9	16.9	35.2	45.8
A2RUW1	Tollip	622	641.3	659.2	639.3	543.8	528.9
A2VCW2	Cav1	2535.3	2560.3	1665.2	1662	2571.9	2257.1
A2VCW9	Aass	7977.4	7571.7	6072.7	6239.2	5151	5427.5
A4GW50	Stk38l	58.7	62.3	84.2	82.3	60.8	59.3
A4L9P7	Pds5a	832.6	1149.8	754.2	946.1	939.4	1054.9
A7M746	Krt83	274	283.2	433.3	526.6	997.2	269.2
A7VJC2	Hnrnpa2b1	3294	3699.5	3515.3	3039.1	5096.7	4563
A9CMB7	Dars	4534.7	5067.1	4883.6	4458.1	5316.2	4916.6
A9CMB8	Mcm6	522.9	755.5	1020.8	1111.3	1330.6	1327.3
A9CME3	C4bpa	745.4	811.9	1986.8	1964.2	1288.2	1379.2
A9EEP5	Aldh1a3	831.5	949.2	1226.4	1103.5	1129.3	1133.9
A9UMV9	Ndufa7	544.7	608.7	476.5	406.7	428.8	447.9
A9UMW0	Ubl5	796.5	882.9	1845.8	1510.7	1069.8	999.8
B0BMS8	Myl9	944.4	814.2	822.4	854.1	1044.6	1024.7
B0BMT9	Sqor	3370.8	3470.9	3082.2	2928.5	2973	2947.4
B0BMV9	Psmd10	302.1	340.8	396	404	386.9	371.9
B0BMW4	Gnas	644.5	319.3	642.3	596.2	226.5	236
B0BMX3	S100a16	49.9	60.9	445	235	57.5	44.3
B0BMY6	Otulin	82.2	109.6	176.2	119.7	114.5	107.9
B0BMY7	Twf2	71.9	117.6	186.3	169.6	296.3	258.7

B0BMY8	H3f3b	10417.9	7918.8	7172.9	8239.7	9246.3	8949.1
B0BMZ1	Fam241b	69.5	59.1	24	23.9	40.8	40.4
B0BN02	Mtx1	990.9	629.4	416.8	391.4	474.3	457.2
B0BN06	Bcas3	128.8	140.7	132.3	108.9	132.6	125.2
B0BN18	Pfdn2	807.9	715.8	728.3	726.6	731.4	693.1
B0BN31	Fopnl	357.8	542.9	1549.6	1296	687.6	570.2
B0BN46	Grhpr	933.7	735.1	671.3	707.7	569.3	581.3
B0BN52	Mtch2	1105.6	942	754.9	675.9	750.3	792.1
B0BN55	Urod	152	170.1	210.1	154.7	156.3	159.7
B0BN63	Ahsa1	1311.5	1323.6	1060.2	1098.6	1272.1	1349.7
B0BN74	Bag2	813.7	618.1	453.9	534.2	472.9	532.3
B0BN81	Rps5	4162.9	2811.4	2349.3	2241.5	1973	1867.6
B0BN85	Sugt1	2007.5	1426.8	1363.3	1282.5	1227	1273.5
B0BN90	Dimt1	84.1	136.1	277.6	339	259	211.1
B0BN93	Psmd13	4602.5	5374.3	5524.7	5248.4	5323.7	5339.3
B0BN94	Fam136a	579.7	597.2	625.6	607.7	575.4	553.1
B0BN97	Txndc12	622.5	696.6	606.3	669.6	928.3	928.6
B0BNA5	Cotl1	926.1	857.1	860.2	889.8	1249.1	1359.4
B0BNB0	Golt1b	156.2	183.6	378.6	296	208.3	172.6
B0BNB2	Denr	1508.7	1384.2	1022.9	1051.9	1303.1	1280.6
B0BNC4	Phykpl	39.1	62.1	48.3	53.1	56.5	49.2
B0BND5	Rrp9	919.8	922.4	1091.8	1039.9	1146.6	1030.2
B0BNE6	Ndufs8	2262.1	2132.9	1320	1343.3	1491.2	1481.3
B0BNF6	March5	133.9	152.1	164.8	153.9	223	194.2
B0BNG0	Emc2	1780.1	2218.3	1719.9	1595.2	1525.3	1667
B0BNG3	Lman2	5059.2	3863.3	3579.1	3385	3062.4	3151.2
B0BNI6	Slc25a35	329.2	540.1	171.2	202.4	311.4	333
B0BNJ1	Sri	1396.9	635.9	712.8	641.5	682.1	665.9
B0BNK1	Rab5c	1762.7	1938.4	1989.3	1821	2468.2	2289.8
B0BNM9	GLTP	296.7	412.7	478.6	446.3	668.4	676.4
B0BNN0	Tsta3	1081.3	1098.6	960.4	925.4	943.1	969.2
B0BNN3	Ca1	1684.4	2130.1	3556.2	3376.4	2708	2486.3
B0K008	Eif1	1786.8	1678.1	760.1	861.6	1409.8	1453
B0K010	Txndc17	1482.9	1508.2	1814.9	1435.7	1379.9	1494.4
B0K020	Cisd1	254.1	260.7	159.3	115.7	283.7	274.8
B0K025	Ostc	988	1671	675.2	641.7	1040.5	1139.9
B0K030	Dnajb1	400.4	413.4	613.2	585.1	510.3	551.5
B0LT89	Stk24	1677.4	1509.6	1365.6	1543.7	1935.9	1817.2
B1H216	Hba-a1	16199.6	19922.7	13462.3	15398.6	18653.2	18905.2
B1H227	Rcc1	411.7	401.2	358.7	388.1	669.9	707.9
B1H230	Map2k3	701.8	686.4	341.5	327.6	535.1	599.4
B1H241	Ric8a	35.5	43.3	87.7	112.5	66	65.3
B1H248	Vps36	105.8	108.2	121.5	116.3	148.5	117.2
B1H249	Gnpnat1	2817.9	2875.3	4795	4254	2066	2016.4
B1H257	Borcs5	137.4	114.3	163	151.7	144.4	127
B1H267	Snx5	986.4	818.9	440.2	372.8	688.3	615

B1H269	Ddx27	194.2	218.6	286.6	257.2	231	216.8
B1H275	Bud23	136.7	175	649.4	975.7	259.2	240.5
B1H282	Colgalt1	199.9	243.7	657.8	407.9	431.6	486.7
B1PLB1	Cd34	1200.2	1163.5	741.1	729.4	1251.6	1372.2
B1VKB4	Synpo	87.6	102	201.4	151.3	147.3	136
B1WBQ0	Cdc5l	880	1137	1004.6	971.5	1330.4	1355
B1WBS4	Vps26b	423.5	537.2	515	532.8	653.1	652.6
B1WBW0	Mphosph10	177.8	163.3	331	281.3	219.1	203.6
B1WBW4	Armc10	1271.7	1429.7	1610	1681.3	1583.3	1566.6
B1WBY1	Cul1	803.5	1173.1	1348.6	1435.3	1344.1	1375.1
B1WBY2	Flad1	37.8	57.1	126.8	133.4	118.4	101.2
B1WBY5	Dnajc11	399.3	374.5	254.8	665	341.4	380.6
B1WC02	Ctps1	1121.5	1156.2	898.8	1162.4	1225.6	1316.4
B1WC16	Bclaf1	1651.1	1791.3	3814	3286	1918.2	1833.4
B1WC26	Nans	3985	4733.7	4424.2	4715	4773.7	4607
B1WC32	Uba2	1097.7	916.4	782.3	754.7	1033.3	1052.9
B1WC34	Prkcsh	6981.7	5055.2	3598.6	3660.7	4361.5	4516.6
B1WC37	Trmu	257.5	238.7	221.1	230	218.3	208
B1WC49	Api5	3036.5	3670.8	4069	3667.9	4494.6	4270.8
B1WC56	Nhp2	302.4	365.3	321.8	261.2	245.3	300.7
B1WC61	Acad9	2363.7	2738.8	2302.4	2308.5	2276.1	2298.3
B1WC66	Nt5dc1	107	133.4	233.5	186.7	118.1	114.9
B1WC67	Slc25a24	466.9	581.7	997.5	938.7	912.1	816.7
B1WC73	Arl6	93.1	115.3	121.1	123.7	157.1	139.3
B1WC84	Cnpy4	407.9	518.2	699.3	675.9	865.3	803.1
B2GUV2	Vps52	172.9	190.6	215.8	217.9	225.3	227.1
B2GUX5	Nt5c3a	111.2	116.6	135	100.4	105.5	89.6
B2GUZ3	Mthfd1l	1165.2	1176.5	933.6	1034.8	1264.3	1139
B2GUZ5	Capza1	1133.6	1141.4	1439.7	1393.4	1448.2	1339.8
B2GUZ9	Fam49b	405.9	345.1	354.3	357.2	257.6	259.2
B2GV01	Mta2	486.6	496.5	572.7	559.2	640.2	650.3
B2GV06	Oxct1	631.8	691.5	471.5	474.1	597	566.5
B2GV08	Ap1s2	81.7	110.9	196.2	200.8	154.5	158.6
B2GV14	Txlna	1247.7	753.1	741.4	778.7	1051.1	979.6
B2GV15	Dbt	3554.9	3413.4	2605.2	2784.8	2326.2	2247.7
B2GV33	Maoa	5962.6	6344.1	8593.4	7744.1	6448.6	6153.3
B2GV54	Nceh1	464.6	514.5	1036.4	903.8	612.7	546.2
B2GV55	Ube2q1	255.8	292.8	310.1	377.3	413.3	404.5
B2GV57	Cars2	156.3	195.3	372.6	313.5	166.5	159.5
B2GV72	Cbr3	907.5	681.5	501.7	509.7	675	754.6
B2GV73	Arpc3	630.9	1035	1184.7	1318.7	2146.6	2007
B2GV82	Nle1	294.7	368.7	375.4	441.1	370.5	332.4
B2GV92	Ptges3	1863.5	2484.1	2615.7	2342.9	3243.9	3210
B2GV99	Myl6	5255.7	4615.5	4768	4801.7	6548.8	6932.9
B2GVB7	Apeh	2808.2	3195.9	3469.5	2885.2	3248.8	3178.5
B2GVB9	Fermt3	431	420.7	537.9	552.3	685.9	699.1

B2LYI9	Tnc	230.6	242.6	211.3	199.6	282.7	304.6
B2RYB8	Itgb2	911	973.8	1911.9	1797.2	1413.3	1486.9
B2RYC5	RGD1310209	607.8	793	843.2	845.3	806.9	730.8
B2RYC9	Gba	114.2	135.1	148.5	155.7	172.8	161
B2RYD0	Ube2g2	896.8	997.9	1244.5	1269.3	884	896.3
B2RYD2	Esrp1	304.4	260.2	267.3	311.7	235.6	211
B2RYD7	Stt3b	966.1	1000.8	1573.6	1116.3	641.7	681.6
B2RYE6	Gigyf2	27.4	38.1	25.2	29.4	46.9	32
B2RYF8	Cnpy3	229.2	199.7	112.8	139.4	263.7	280.2
B2RYG2	Pck2	12102.3	12287	9976.8	9310.1	8422.6	8444.3
B2RYG6	Otub1	1735.9	1626.2	1551.2	1397	1670.3	1643.3
B2RYI2	Srp68	5574.7	4724.3	3799.3	3545.8	3216	3217.3
B2RYJ7	Actr1b	167.9	171.3	81.5	75.9	107.3	123.6
B2RYK3	Spr	717.5	850.8	1101.2	940.5	945.6	986.2
B2RYM3	Itih1	527.6	517.6	682.3	724.2	659.5	721.5
B2RYN0	Pgm3	4795.5	4750.4	4478.9	4549.4	4362.7	4484.2
B2RYN1	Fn3krp	366.9	497.9	831.8	762.8	512.8	473.5
B2RYN3	Eef1e1	749.3	990.7	1159.6	1152.1	1083.1	940.6
B2RYN6	Ap1g1	1574.9	1846.8	2036.2	2080.5	1799.9	1778.7
B2RYP4	Snx2	1857.7	1861	1962.7	2126.6	2078.8	2044.6
B2RYP6	Luc7l2	1459.3	1523.8	1495.6	1231.8	1611.9	1608.7
B2RYQ2	Ptpa	297.5	446.4	228.7	239.2	537.9	540.7
B2RYQ8	Rpl36a	11604.3	11239.9	4915.2	4504	8143.7	7665.5
B2RYS0	Cox7a2	649.5	541.4	280.2	288.3	493.1	472.6
B2RYS2	Uqcrb	2850.8	2603.3	2420.7	1916.2	2020.4	1919.1
B2RYS8	Ndufb8	1123.4	1489.3	1147.2	1101.8	1143	1251.9
B2RYT4	Mrps14	85.2	115.6	127	130.8	117.6	104.5
B2RYT5	Cox7a2l	1786.2	1556.4	1997.3	1896.8	1164.8	1137
B2RYT7	Hdhd3	788.7	685.9	477.2	362.4	371.7	398.2
B2RYU7	Cbx5	748.2	809.5	487.8	543.5	836.3	851.6
B2RYW3	Ndufb9	2071	1520	1353.9	1327.8	1026.2	1070.4
B2RYW7	Srp14	65.8	102	254.1	234	99.7	100.1
B2RYW8	Micos10	172.9	166	374.5	406.9	205	169.4
B2RYW9	Fahd2	1212.9	1056.5	914.6	863.4	820.4	816.5
B2RZ08	Tmem263	2223.5	2073.2	1380.7	1307.1	872.6	1044
B2RZ24	Sucla2	3719.8	4010.7	3136	2970.1	3380.3	3360.8
B2RZ27	Sh3bgrl3	931.2	703.1	799.7	630.6	774.5	926.8
B2RZ33	Nck1	1139.8	1053.3	427.1	459.6	839.8	913.6
B2RZ37	Reep5	4890.9	6134.1	4123.4	3528.5	4546.3	4244
B2RZ38	Rragd	106.5	122.7	166.2	134.2	169.4	167.3
B2RZ47	Exosc5	39.4	45.7	31.8	37.2	40.7	48.7
B2RZ66	Srp19	943.1	1074.8	1103.9	960	962.4	787.7
B2RZ72	Arpc4	420.8	345.8	657.8	549.1	327.3	342.6
B2RZ74	Snrnp70	1627.1	1894.9	1949.7	2036.7	2437.7	2382.4
B2RZ77	Dpt	937	1013.4	1109.7	1374.7	1184.8	1148.7
B2RZ78	Vps29	1855.2	1924.5	2641.8	2803.9	2269.8	2095.3

B2RZ98	Ncf4	89.2	115.3	253.4	224.8	260.8	250.2
B2RZA4	Ap1m2	1554.3	1771.4	1643.7	1655	1603	1543.5
B2RZA9	Ube2l3	5766.1	5755.9	6241.8	5898.4	6210	6019.8
B2RZB6	Lsm8	437.6	445.6	388.4	395.8	488.8	481.6
B2RZB7	Snrpd1	226.4	180.8	165.9	158.8	225.9	212.8
B2RZC6	Ilf2	448.4	494.9	492.1	565.6	523.8	523
B2RZD1	Sec61b	4132.5	2492.8	2081.7	2029.4	1475.6	1345.4
B2RZD4	Rpl34	3249.6	3146.2	867.3	755.8	1619.9	1737.5
B2RZD5	Rpl22l1	1341.7	1280.4	1233	1217.3	994.9	831.7
B2RZD6	Ndufa4	3125	3368.9	2785.3	3273.1	2300.1	2049.5
B3DM88	Ehbp1l1	57.7	64.2	90.2	101.8	116.4	106.5
B3DM93	Pdcd4	2610	2581.7	2120.9	2290.6	1827.9	1807.4
B3DM95	Ptms	688.6	665.9	194.6	242.9	757.3	770.4
B3DMA0	Tp53i11	128.2	101.1	70.7	85.9	137.3	164.6
B3GNI6	Septin11	1539.5	1106.7	1010.1	996.6	1158.5	1108.5
B3IYD2	ufc1-s	1236.8	1050.4	599.7	682.1	851.8	798.2
B3STU1		121.9	157.5	157	202.6	212.9	201.7
B3SVE6		282.2	315.3	232.6	211.9	297.9	272.4
B4F758	Hmgb1	6644.4	7547.2	11856.7	11763.9	12108.2	10870.2
B4F759	Phf5a	119.1	130.7	97	95	157.6	169.8
B4F765	Ift27	100.5	128.4	261.8	203.2	144.6	129.3
B4F775	Gopc	360.3	338.3	573.8	547.1	321.2	315.2
B4F778	Rfc4	30.5	46.7	24.3	37.6	61.2	59.2
B4F779	Appl2	1746.8	2010.5	1521.9	1775.3	1775.9	1871.1
B4F7A3	Lgalsl	132.5	172	155.6	183.7	213.6	201.1
B4F7A5	Cd99	178.6	178.9	298.7	279.7	244.5	249.5
B4F7C9	Stt3a	3446.6	3493	2184.7	1976.3	2453	2463.6
B4F7E8	Niban2	47.3	58.9	103	100.6	86.5	97.2
B5DEF3	Ggcx	102.3	116	44.4	59.6	109.7	126.4
B5DEH0	Limd1	191.5	183.3	294.4	334.6	184.5	160.3
B5DEH2	Erlin2	4030.9	3824.9	3507	3183.8	3537.5	3512.5
B5DEH4	Uap1l1	197.5	232.8	372.2	373.2	304.7	267.6
B5DEJ5	Eefsec	344.8	304.3	516.4	525.6	321.2	264.8
B5DEJ6	Tpk1	266.5	283.6	217.2	276.1	300.3	252.4
B5DEK0	Rprd1b	583.6	683.1	734.5	1556.4	653.6	668.9
B5DEL8	Ndufs5	727.9	850.5	740.7	745.5	709.1	642.5
B5DEL9	Rps7	6038.5	5756.2	2836.8	3324	4274.3	4283.3
B5DEN5	Eef1b2	9550.7	9740.3	6795	5918.1	8750.4	8532
B5DEN9	Vps28	256.2	193.1	122.2	134.6	165.5	170.8
B5DEP0	Dguok	506.8	297.8	154	156.5	211.2	192.7
B5DEP4	Mrpl42	569.8	734.5	697.9	734.2	480.3	440.5
B5DEP6		2257.4	2548.3	3222.5	3262.9	2731.9	2682.1
B5DEP7	Snrpg	784.8	833.1	596.7	667.8	994.3	862.2
B5DEQ4	Snrpb2	1812.4	1968.6	1153.8	1342.7	1945.5	2020.7
B5DER3	Iah1	267.4	269.3	372.4	361.6	262.4	269.5
B5DER4	Mrpl1	1089.1	1001.2	1865	1863.6	974.5	889

B5DES0	Snrpd2	3941.4	4148	5300.6	5170.4	5033.1	4805.1
B5DEX6	Susd2	33.8	50.3	49.4	83.3	83.1	59.3
B5DEY8	Snx6	1901	1804.2	2004.6	2092.4	2351.3	2251.2
B5DEZ4	Tcerg1	408.9	467.1	499.2	511.7	617.4	653
B5DEZ8	Plxdc2	116.9	142.7	254.5	218.5	193.6	199.7
B5DF45	Traf6	70	74.3	156.1	135.6	66.3	50.9
B5DF46	Pmm2	871.1	744.3	1013.1	1416.3	726.8	699.1
B5DF50	Galnt2	599.3	632.3	543.2	611.5	640.5	655.8
B5DF51	Mmgt1	235	286.6	483.9	422.7	309.3	306.6
B5DF55	Stam	362.6	425.1	691	654.9	503.4	425.3
B5DF65	Blvrb	1942.7	1629.6	1954.3	1902.9	1439.2	1436.5
B5DF80	Pabpc6	338.7	350.6	463.5	433	241.1	253.1
B5DF89	Cul3	471.5	833.4	1103.7	931.8	1234.8	1206
B5DF91	Elavl1	1096.1	1254.3	1046	1232.4	1565.8	1528.5
B5DFA0	Vil1	3810	3742.4	4421.9	4969.2	3181.1	2870.6
B5DFB6	Atxn2	184.9	227.4	371	283.1	394.7	324.7
B5DFC8	Eif3c	8733.7	7092.3	6088.2	6625.7	5933.7	5915.4
B5DFC9	Nid2	2373.1	2705	2513	2600.9	3123.6	3084.7
B5DFD1	Nudcd1	582.9	667.3	644.3	552.5	622.8	616.1
B5DFD8	Sh3bgrl	1496	1121.5	1251.3	1297	1394.5	1275.4
B5DFF4	Vps37c	131.9	142.6	210.8	161.4	128.9	115.2
B5DFI0	RGD1306717	158.3	152.4	130	142.7	191.8	204.5
B5DFI1	Cog1	267.7	348.1	697.9	653.1	458	419.2
B5DFK6	Ap3d1	2704.6	2568.8	2073	2200.9	2212.9	2163.8
B5DFM8	Bcas2	391.2	543.1	494.2	503	597.7	624.5
B5DFN2	Ahcyl1	715.6	568.6	485.5	484.9	571.5	564.3
B5DFN4	Pfdn5	823.3	1079.6	1353.2	1287	1469.7	1353
B6DYQ7	Gstp1	7886	6818.6	4235.8	4591.3	6631	6562.2
B6DYQ9	Gstt2	1581.7	2107.4	2418.4	3183.8	2128.8	2178
B6RK61	Myh7b	16.7	26.7	60	163.4	29.1	32.9
C0JPT7	Flna	20205.2	17978.3	19928.7	20402.8	26457.5	27930.4
C5NTX8	TSN3	736.4	623	882.1	831.8	696.9	692.1
C9WPN6	Eif2s3y	322.6	363.6	770.4	568	373.7	409.1
D2XV59	Gtpbp1	147.4	203.4	121.3	162.2	219	191.9
D3Z7Z5	Ranbp10	69.4	101	107.6	113.3	122.3	118.9
D3Z865	Mpdu1	654.9	868	1055.6	992.6	850.6	717.6
D3Z881	Tbc1d4	381.6	515.7	763.1	749.9	652.4	600.9
D3Z898	Samhd1	607.7	706.7	818.7	866.6	1239.3	1277.2
D3Z8B2	Nup133	523.6	562.1	641.3	689.4	706.1	642
D3Z8E0	Rps6ka3	271.5	282.9	282.9	186.1	310	324.7
D3Z8F1	Vill	109.6	151.1	135	152.9	141.8	147.5
D3Z8L7	Rras	1032.5	980.1	1090.7	960.4	814.5	843.5
D3Z8R4	Rbm25l1	1343.9	1427.7	1556	1253.2	1384.2	1368.8
D3Z8W0	Paox	933	1268.1	1789.7	1791.8	1847.9	1800.1
D3Z8X6	Dtx3l	580.5	650	526	501.2	884.7	900.2
D3Z900	Marc2	4733.3	4520.5	3776.9	3409.8	3016.5	2657.4

D3Z941	Mars	8870.9	7370.8	6848.8	7017.1	7003.3	6529.5
D3Z955	Pgm2l1	238.3	199.1	118.8	107.4	233.7	205.7
D3Z9D2	Fyco1	413.6	391.4	1071	967.8	517	476.3
D3Z9E1	Emilin1	220.6	208.5	189.5	204	403.5	428
D3Z9I1	Coa3	741.4	708.5	477.7	535.8	615.8	568.1
D3Z9J8	Sat2	194.6	247.8	115.8	153.3	214.5	212.4
D3Z9K4	Plekho2	337.4	341.1	419.6	418.7	506.2	565.1
D3Z9L0	Agk	237.8	354.8	493.7	395.6	382.6	315.6
D3Z9L5	Wdr11	599.6	882.8	745.5	699.6	649.6	626.9
D3Z9M1	Mettl16	117.4	141.2	279.7	282.5	180.3	185
D3Z9P1	Kdsr	641.4	652.7	497.4	594.6	763.6	753.9
D3Z9U2	Cd163	774.8	668	663.3	733.2	1216.4	1202.8
D3Z9Z0	Ank1	351.2	431.9	376.8	430.8	443.7	430.2
D3ZAF5	Postn	118	88.1	168.9	154.3	88.7	85.3
D3ZAF6	Atp5mf	1304.6	1010.3	816.7	1297	720.4	941.2
D3ZAF7	Tbc1d2b	346.8	481.7	1964.6	2834.5	396.8	339.6
D3ZAI6	Nt5dc3	43.4	63.1	106.4	109.5	91.6	74.9
D3ZAM3	Cpb1	2723.7	2653.1	2074.4	1814.5	1590.9	1629.9
D3ZAN3	Ganab	3271.3	3031.1	2788.3	2624.4	3100.8	2944.9
D3ZAP7	Chd7	420.2	306.6	1206	1120.8	392.7	291.9
D3ZAP9	Gpd1l	3827.9	3462.4	3291	3394.5	2999	2996.5
D3ZAQ0	Fundc2	491	546.1	351.8	436.8	471.2	465.2
D3ZAR1	Ldlrap1	83.1	84.5	88.1	103	108.2	100.6
D3ZAS8	Sart3	736.6	1013.6	1252.5	1000.4	1210.6	1217.5
D3ZAS9	Ddrgk1	32.6	41.3	90.4	89.9	46.3	42
D3ZAX5	Cherp	832.2	916.1	746.2	831.6	1120.3	1190.1
D3ZAY8	Pnn	228.4	235.5	256.4	389.3	291.4	287.2
D3ZAZ0	Eif3m	1583.9	2039.1	2678.5	2747	2660.9	2247.1
D3ZB48	Hook1	70.7	79.7	60.4	63.2	85.8	64.7
D3ZB65	Bccip	202.6	226.2	408.1	346.1	385.3	424.1
D3ZBE5	Nek7	701.1	743.7	750.3	660.8	733.6	713.1
D3ZBL6	Nup160	340.3	380.5	403.5	434.2	459	433.6
D3ZBM3	Fech	742.7	810.3	1025.9	1054.6	848.8	845.6
D3ZBN0	H1-5	3444.1	3981.4	7808.1	7209.1	6213.8	5981.7
D3ZBR0	Krtap3-3	637.7	780.4	1757	1902.3	1691.5	692.4
D3ZBS2	Itih3	837.9	1024.1	1484.1	1732.2	1515.1	1488.8
D3ZBT9	Ppp6r3	901.3	1352.1	1183.4	1124.6	1750.8	1733.7
D3ZC46	Tcf25	83.1	92.9	102.5	79.7	106.6	103.1
D3ZC63	Cmpk2	68.8	84.2	104.6	106.6	79.3	80.7
D3ZC82	Nufip2	89.4	85.9	243.5	217.9	91	95.5
D3ZC84	Usp9x	1324.3	1636.9	2136.4	2173.5	2090.9	2020.3
D3ZC89		285.3	230.9	103.7	114.1	246.9	280.9
D3ZCA0	Plpbp	67	84.2	44.2	49.3	75.2	77.5
D3ZCF8	Abca8a	1366.1	1584.2	1541.3	1733.9	1865.3	1664.7
D3ZCL3	Snrpc	550.9	719	404.2	435.2	932.9	830.4
D3ZCP9	Gtf2e2	223.9	223.5	154	181.7	222.4	242.5

D3ZCQ2	Alg11	126.6	176.8	201	232.6	153.2	141.4
D3ZCR4	Ppp4r3b	58.9	159.3	228.2	228.2	139.7	121.9
D3ZCS3	Pcbp4	2576	2967.4	1788.5	1903.5	2473.5	2602.8
D3ZCT5	Pald1	148.5	210.6	453	447.5	233.6	205
D3ZCT7	Sec23b	6021.3	4744.8	4647.4	4376.8	3643.8	3569
D3ZCZ9	LOC100912599	1826.1	2007.5	1835.2	1530.4	1339.1	1343.3
D3ZD09	Cox6b1	4403.2	4473.9	4978.8	4502.7	3696.1	3564.6
D3ZD11	Spcs2	7896.1	8385.8	5236.3	5335.8	5393.7	5453.8
D3ZD19	Lyve1	74.4	110.8	162.3	149.5	203.8	210.8
D3ZD23	Abce1	9024.9	8883.3	7294.5	7057.5	6863.6	6496.6
D3ZD31	Mrc1	4736.8	5174.8	7786.6	8375.1	8489.3	8862.5
D3ZD73	Ddx6	846.4	939.7	659.7	577.7	1133.4	1080.9
D3ZD83	Mfsd10	576.5	724.6	515.7	483.9	803	766.9
D3ZD89	Naa15	907.5	1006.2	2100.5	2225.2	1337.1	1182.3
D3ZD97	Dhx15	2165.3	2672.6	3546.9	3449.2	2775	2676.1
D3ZDB9	Nmral1	204.4	241.3	160.2	120.5	242	233.9
D3ZDD7	Strbp	274.4	307.6	337.4	384.9	305.4	325.8
D3ZDI7	Ppp2r5a	344.3	408.7	913.5	714.3	501.4	486.3
D3ZDJ4	Unc93b1	183	209.8	69.6	63	302	164.8
D3ZDK7	Pgp	665.9	454.3	523.9	496.2	458	410.4
D3ZDZ1	Apbb1ip	407.5	476	1246.3	1209.3	1205.2	1254.3
D3ZE09	Tln2	265.5	295	607.9	565.6	338.4	317
D3ZE15	Ndufa13	2346.8	2902.8	1571.8	1707.1	2161.2	2244.5
D3ZE21	Tmed11	1674	1914.4	2241.7	2305.9	1625.2	1396.3
D3ZE59	Tmem115	488.2	508.7	1053.1	937.7	489.5	491.7
D3ZE63		1904.6	1739.4	462.6	497.6	1425.7	1458.4
D3ZE72	Metap1	1032	976.2	1272.9	1378.3	904.9	853.6
D3ZEA0	Fndc3a	376.6	444.9	641.3	763.8	514.7	473.4
D3ZEH2	Foxred1	42.8	44.3	42.3	43.3	32.5	28.5
D3ZEI0		3377.5	3202.3	1618.7	1711.3	2075.3	2264.7
D3ZEW3	Tcaf2	217.2	272.5	358.2	340.9	281.5	290.8
D3ZF12	Spcs3	2388.6	3992.2	3214.3	3528.9	4434.8	3892.9
D3ZF21	Gprin3	159	157.2	447.9	411.5	230.8	250.6
D3ZF26	Tnks1bp1	403.3	503	468.1	443.7	704.1	689.9
D3ZF39	Uap1	357.6	353.8	418	452.9	410.4	435.1
D3ZF45	Larp4b	40.6	37.5	35.2	45.9	50.3	49.5
D3ZF54	Ano10	140.8	129.2	115.4	121.5	155.9	156.2
D3ZF86	Arfgef3	302.2	363.4	242.6	233.4	343.8	315.2
D3ZF97	Erlec1	3264	2760.1	1718.3	1623	1741.3	1631.3
D3ZFA8	LOC100362366	2349.1	3038.6	1675.7	1612.1	3223.5	3108.9
D3ZFB2	Luc7l3	407.2	400.8	304.2	315.9	403.9	416.1
D3ZFF4	Lclat1	2047.6	2392	2282.5	2377.1	1775.9	1787.4
D3ZFG3	Cela3b	12491.2	12998	7796.9	8554.2	10456.8	11289.6
D3ZFJ3	Sh3bp1	133.3	188.6	263	283.1	365.3	347.9
D3ZFJ6	Lactb	1244.3	1323.4	1528.5	1430.2	1228.3	1320.3
D3ZFK5	Spcs1	2223	2941.2	3338.3	3038.9	2308.5	2127.1

D3ZFP4	Mcm3	83.4	98.3	102.1	114.7	216.1	193.5
D3ZFQ8	Cyc1	1870.2	1220.3	1310.6	1370.3	1264.6	1236.9
D3ZFS7	Lyplal1	204.6	432.3	266.2	573.9	389.2	422.9
D3ZFY0	Sephs1	174.1	181.6	102.5	102	175.5	172.6
D3ZFY8	LOC100912618	366.7	362.3	407	330.6	353.5	309.6
D3ZG78	Zzef1	302.3	277.5	419.6	399	303	251.3
D3ZG88	Znrd2	731.3	794.4	830	701.4	740.5	698.4
D3ZGE2	Mpo	174.7	206.1	679.8	920.3	499.9	557.4
D3ZGE6	Cttn	1040.7	1004.6	1299.9	1130.4	1089.7	1049.9
D3ZGF1	Cd44	211.6	282.5	1348.4	1235.6	402.9	438.3
D3ZGL1	Arhgap25	69.6	91.2	192	200.6	161.2	147.9
D3ZGN8	LOC103690015	536.6	447.6	483.2	656.6	309.4	305.9
D3ZGW2	Ap1g2	842.3	925.1	1058.6	1038.1	1088.6	1072.2
D3ZGY1	Pym1	676.8	501.3	547.5	454.7	248.4	326.3
D3ZGY2	Otud6b	80.3	100.4	213.3	172.6	108.5	97.4
D3ZH12	Nosip	91.7	91.9	64.5	68.6	94.9	93.5
D3ZH41	Ckap4	2422.3	2501.2	3757.7	4063.5	3432.4	3659.7
D3ZH44	Syk	109.7	129.2	231.9	210.7	147.5	150.5
D3ZH75	Akt1s1	158	181.9	238.5	289.1	192.9	215.1
D3ZHA0	Flnc	601	661.9	830.2	901.6	979.6	954.5
D3ZHD1	Anxa13	335.9	397.6	456.9	424.2	498.9	464.5
D3ZHG8	Gga3	211.2	241.4	508.8	473.3	317.4	295.7
D3ZHM7	Dnttip2	600.7	726.3	2028	2375.9	811.9	770.8
D3ZHW0	Dhx29	331.1	369.1	361.2	382.3	380.4	365.5
D3ZI07	Kif3b	232.1	235.9	155.2	173.4	271	279.1
D3ZI16	Cops6	1512.5	1557.8	1613.9	1787	1263.4	1160.6
D3ZIE9	Aldh18a1	1569.8	1912.2	1422.1	1500.4	1451.7	1546
D3ZIF0	Zfp512	400.4	392.7	520.3	492.4	537	496
D3ZIF5	Ap3b1	2329.8	2565.9	3859.5	3429.7	2441.4	2415
D3ZIK0	Glce	52.9	59.8	47.2	47.3	66.5	77.5
D3ZIM7	Fam177a1	241.8	235	280.8	303.6	345.7	322.3
D3ZIN7	Mrps23	434.3	428.3	344.2	357.6	398.7	364.6
D3ZIY3	Ythdf3	188.7	254.4	190.4	198.2	241.9	234.2
D3ZJ01	Relch	205.9	268.1	930	763.2	354	305.2
D3ZJ32	Esyt2	977.3	1003.5	1186.8	1362.4	1075.4	1045.8
D3ZJ50	Pkp3	190.4	213	160.2	180.3	204.7	177.2
D3ZJ92	Prpf40a	469.9	528.4	808	744.7	641.3	643.3
D3ZJA9	Slc27a3	27.6	21.7	26.8	33.4	41.2	38.8
D3ZJB1	Mfap5	881.3	352.2	330.1	321.7	364.1	374
D3ZJH9	Me2	283.8	435.3	330.3	381.1	664.4	703.2
D3ZJS3	Tomm6	168.3	191.6	106.2	110.5	201.1	187.1
D3ZJV9	Tmed6	643.6	444.7	232.3	213.5	182.8	171.7
D3ZJX5	Timm50	589.3	400.1	467.6	453.7	351	303.6
D3ZK45	Epb41l5	399.9	479.6	539.3	454.5	465.3	435.7
D3ZK73	Cul4b	34.8	61.5	38	38.2	69.7	90.1
D3ZK96	Papola	441.5	521.5	1071.7	923.2	641.2	661.4

D3ZKG1	Mmut	3332.8	3525.7	2897.3	2965.1	2482	2477.2
D3ZKG9	Cluh	655	637.1	462.6	618.9	603	625.6
D3ZKH2	Mon2	492.5	719	954	964.8	1016.9	995.4
D3ZKQ4	Rabl6	250.1	301.2	385.9	395	294.7	268
D3ZKT8	Hddc2	152.4	170.7	195.5	193.8	173.4	161.3
D3ZL10	Col6a6	1080.3	1039.4	1821.3	1794.6	1392.4	1198.5
D3ZL21	LOC685619	183.8	192.1	294.1	247.3	246.1	262.8
D3ZL23		61.2	122	128.6	122.3	171.2	96.8
D3ZL50	Ttc37	2329.9	2932.4	2921.3	3325.6	3875.6	3488.8
D3ZL86	Heatr1	388.6	469.8	911.7	908.5	609.3	525.3
D3ZLA3	Cpne3	563.3	615.9	584.4	448.7	659.9	691.9
D3ZLC1	Lmnb2	283.1	311.3	276.3	295.4	426.1	386.9
D3ZLD5	Golga3	3698.3	3820.8	3677.8	3853.6	3298.5	3687.4
D3ZLE2	Tmcc3	114.7	179.4	147.4	137.6	191.8	171.5
D3ZLH8	Rtf1	358	524.7	799.5	743.9	574.2	720.1
D3ZLK9	Naxd	437.9	394.7	421.2	444.5	352.5	328.7
D3ZLL8	LOC100909878	1318.3	932.6	633.8	657.4	725.9	711.5
D3ZLM5	Nhlrc2	372.7	278.8	332.3	323.8	244.5	247.6
D3ZLT1	Ndufb7	1574.3	1496.9	824.5	803	999.4	1085.1
D3ZLX3	Mob2	312.6	339.1	383.8	373	272.5	285.9
D3ZM03	LOC100912478	384.1	315.4	488.4	482.7	291.3	264.7
D3ZM09	Sars2	101.6	134.2	86.3	96.4	131.6	116.5
D3ZM21	Comtd1	222.6	227.9	317.7	282.3	213.9	235.3
D3ZM69	Epb41l2	2776.8	2797.4	3535.9	3273.3	3297.7	3306.4
D3ZML4	Trabd	157.6	189.6	131.2	122.1	149.5	144.4
D3ZMS1	Sf3b2	3093.5	3322	4201.7	4338.6	3593.2	3620.1
D3ZMX6	Sntb2	432.3	445.8	749.2	696.2	441.9	466.5
D3ZMY7	Nt5c2	177.7	180.6	201.9	208.3	163.5	189.9
D3ZN27	Dnajc13	761.9	1301.9	1547.5	1499.8	1310	1215.5
D3ZN37	Rock1	562.9	666.6	941.6	890.2	833.8	749
D3ZN38	Stard5	113.4	131.9	157.7	162.2	194.5	168.5
D3ZN76	Sec16a	2612.9	2691.6	3377.7	3828.5	2376.8	2446.3
D3ZN95	Hcfc1	1502.9	1791.5	3110.6	2779	2196.2	2153.3
D3ZNA3		136.1	227.7	921	786.9	202.3	162.8
D3ZNI3	Pdcd11	320.4	318.5	448.4	456.4	315.7	294
D3ZNJ5	Inmt	460.3	662.1	1042.1	679.9	930	942.8
D3ZNQ6	Ube2m	1361.6	1256.4	2110.4	1840.9	1385.9	1330.7
D3ZNS8	Aaas	470.3	488	666.1	591.8	390	386.4
D3ZP13	Qsox2	2103.1	2185.3	1837.7	1858.8	1782.6	1683.4
D3ZP47	Phpt1	304.2	350.4	574.1	571.8	402.7	373.3
D3ZP96	Mcm2	237.3	295	298.9	321.5	499	507.2
D3ZPF0	Fhl3	651.3	428.7	665.4	786.9	499.9	491.8
D3ZPI4	Rap1gap2	370.8	463	639.1	625.2	484.1	443.7
D3ZPI8	C8g	93.4	103.1	81.7	78.5	136.5	143.2
D3ZPL1	Cpsf6	974.9	1124.8	1190.7	1269	1252.1	1164.6
D3ZPN7		5758.5	5009.5	4183.9	4425.5	3608.9	3443

D3ZPP2	Arl8a	794.8	930.1	478.1	363.2	1068	1315.8
D3ZPP9	Card19	87.6	121.6	168.7	226.4	142.7	143.9
D3ZPR0	Cse1l	3264.5	3696.5	3794.1	3939.1	4195.1	4229.2
D3ZPV8	Ggct	2269.4	2459.3	2724.5	2613.4	2036.8	1957.2
D3ZQ25	Fbln1	361.4	388	580.9	640.9	497.2	511
D3ZQ57	Plxnb2	1109.3	1319.6	1288	1543.7	1652.7	1723
D3ZQ59	Nrdc	1015.8	1019.3	1412.7	1288.4	1122.7	1039
D3ZQ74	Plod1	112.8	136.9	190.4	191.8	260.4	225.4
D3ZQB6	Hdhd5	565.6	526.5	310.1	322.9	432.5	455.9
D3ZQI0	Polr2j	245.5	305.3	496	424	417.9	427.5
D3ZQI1	Gpx7	215.1	233.5	351.3	360.6	515.6	545.2
D3ZQL1	Emc7	1205.9	496.8	474.5	485.9	348.8	368.8
D3ZQM0	Sf3a1	2192.2	2431.7	2569.5	2370.9	3059	2791.7
D3ZQM3	Itga3	117.7	119.2	92.2	111.9	129.4	113.5
D3ZQN7	Lamb1	7796.9	7433	8417.4	8975.7	7836	7955.7
D3ZQV0	Prss3	3025.8	4992.4	3784.4	3631.9	2402.2	3081.8
D3ZRE7	Swap70	96.8	107.9	224.3	244.7	171.4	156
D3ZRH1	Oxa1l	290.7	316.3	347.7	316.5	272.7	282.4
D3ZRM5	Rab23	132.2	160.9	226.6	304.4	217.6	180.3
D3ZRN3	Actbl2	20.1	22.6	26.8	21.1	25.4	29.8
D3ZRV0	Dcun1d1	368.6	525.6	635.2	613.9	509.2	496.9
D3ZRX9	Cnn2	726.3	797.4	941	1239.5	1478	1814.9
D3ZS58	Ndufa2	1151	1145.2	962	957.6	857.4	816.6
D3ZSA9	Nomo1	7651.4	6295.3	6463.6	6405	5011.2	4616.1
D3ZSF3		174.7	180.6	206.2	147.5	173.8	182.7
D3ZSP1	Snrpe	137.8	151	147.6	181.3	177.3	190.3
D3ZSV1	Ift20	658.1	691.8	204.9	213.1	648.8	655
D3ZSW5	Txnl4a	435.3	674.6	780.3	806.5	1100.4	869.5
D3ZSY4	Epx	1987.3	2365.6	3228.5	3503.3	5116.3	5606.6
D3ZSY8	Tbc1d10b	112.2	120.3	164.6	143.1	135.3	126.4
D3ZT01	Cog2	257.3	195.8	129.8	141.5	175.1	188.4
D3ZT03	Upf2	561.3	663.6	587.8	570.2	631.5	632.5
D3ZT52	Pbrm1	191.7	200.1	220.4	215.3	220.3	231.9
D3ZT71	Bcl2l13	1140.4	1207.6	1561.3	1361	1247.7	1239
D3ZT90	Gcdh	2670.8	2574.6	1012.4	886.1	1795.2	1858.2
D3ZTB5	S100a13	588.5	664.4	632.9	578.7	989.1	927.6
D3ZTF1	Slc4a1ap	227.8	264.2	272.4	249.3	303.5	294.8
D3ZTF6	Pik3c2a	277.3	354.2	769.3	792.8	431.5	412
D3ZTJ0	Rp2	699.9	692	1019.2	1077.9	751.5	767.8
D3ZTJ7	Kdm2a	54.5	69.9	90.4	75.1	77.1	80.5
D3ZTP0	Aldh1l2	22697.5	23118.7	15924.5	17172.1	15011.6	14399.6
D3ZTR5	Zbed5	1392.8	1143.5	1199.8	1190.6	1045.1	1018.1
D3ZTT7	Sun2	507.2	582.6	840.5	1063.8	776.7	704.7
D3ZTV7	Ash2l	67.9	90.5	151.3	145.7	136.9	124.6
D3ZTW9	Exog	898.6	787.3	442.2	536.6	838.6	860.7
D3ZTX0	Tmed7	1307.6	1237.2	1452.3	1580.7	1137.2	1082.6

D3ZU13	Eif4g1	2964.2	2962.3	2176.3	2156.2	1971.9	2050.1
D3ZU22	LOC108349606	6460.9	10341.8	7678.5	7993.4	11928.7	12238.6
D3ZU48		532.3	593.6	994.3	914.5	676.3	651.6
D3ZU56	Pds5b	901.1	1042	1562.4	1370.5	1372.4	1315.1
D3ZU74	Dync1i2	3031	3155.6	3737.3	3468.5	3475.6	3480.7
D3ZU83	Ergic3	166.5	175.2	119.9	92.2	144.7	131.8
D3ZUB0	Rcn1	552.6	512.1	695.4	754.1	643.2	757.8
D3ZUB7	Anapc4	15.6	18.2	15.1	25.8	30.7	24.8
D3ZUC9	Oxsr1	85	101.4	152	130.6	106.7	107.5
D3ZUD8	Tm9sf3	2803.1	3750.9	1971.4	2005.3	3491	3466.3
D3ZUF9	Pitrm1	365.5	457.2	313.3	354.9	461.5	507.6
D3ZUI1	Apip	336.3	284.5	286.6	276.9	280.8	238.1
D3ZUJ5	Dtymk	18.5	18.2	42.8	47.3	27.5	26.6
D3ZUJ8	Tmtc3	196.1	197.1	184.9	237.8	165.7	168.5
D3ZUL3	Col6a1	8973.7	8980.1	6320.1	6846.1	11225.4	11611.3
D3ZUL4	Bcl7c	179.1	196.7	317.7	335.4	241	211.7
D3ZUL8	Zcchc8	126.2	146.6	140.5	163.6	228.9	216.5
D3ZUM4	Glb1	711.1	899.1	716	794.6	1095.2	1089.3
D3ZUP5	Brk1	93.6	126.7	140.1	142.1	119.1	113.6
D3ZUT9	Ints3	216.5	237	205.3	200	328.9	311.4
D3ZUU6	Clec3b	105.5	117.2	99.1	109.5	184.3	184.1
D3ZUV3	Eif2a	3462	3286.4	3779.4	3526.7	2626.9	2679.2
D3ZUX1	Hykk	411	335	330.5	463	225.4	213.5
D3ZUX5	Chchd3	2510.7	2424.6	2835	2765.7	1958.5	1895.9
D3ZUX7	Acsf3	1447.3	1403.5	954	1071.7	1105.6	1072
D3ZUY8	Ap2a1	150.2	179.9	251.8	272.4	248.4	238.1
D3ZV29		2610.7	3487.2	2845.5	2753.4	2625.7	2704.3
D3ZV33	Pogz	196.1	213.7	225	230.2	201.6	191.8
D3ZV63	Odr4	1878.9	2379.9	1917.2	1578.5	1669.1	1778.6
D3ZV75	Mfsd1	40.5	56.2	85.1	92.8	87.5	84.3
D3ZV82	LOC685067	461	374.8	665.8	600	576.8	614.7
D3ZV96	Lratd2	204.4	248.8	186.3	185.3	223.1	229.4
D3ZVA5	Fbll1	467.7	398.5	315.9	313.9	372	367.4
D3ZVB7	Ogn	3133	2587.7	2285	2729.9	2355.7	2437.8
D3ZVM5	Hspa12b	196.4	186.2	151.8	156.3	198.7	217.9
D3ZVN7	Ppox	278.9	280.2	504	385.7	233.5	261.6
D3ZVQ0	Usp5	2541.5	2133.8	2121.6	2211.1	2036.6	2027.5
D3ZVR7	Prxl2b	68.2	88.6	47.6	40.2	69.4	53.4
D3ZVR9	Pgm5	682.3	760.5	959.5	1010.5	1142	1227.4
D3ZVS2	L2hgdh	1453.9	1459.5	1358	1262.6	1192.4	1166.4
D3ZVU4	Rbks	1070.8	1199.9	1756.7	1650.8	1365.9	1247.9
D3ZVU7	Hdac1l	72.5	106.7	82.2	81.5	168.1	153.5
D3ZVW3	Zc3h4	406.7	387.7	498.1	430.2	474.2	473.4
D3ZW08	Adsl	1252.1	1018.4	821	772.1	919.6	958.8
D3ZW55	Itpa	2554.6	2814.2	2427.6	2593.6	3062.4	3249.9
D3ZWA8	Appl1	789.9	621.9	569	567.8	584.8	668.5

D3ZWC6	Sntb1	3631.9	4048.5	2903.7	2990	3291.5	3245.6
D3ZWD6	C8a	392.2	373.5	717.8	571.2	540.1	577.8
D3ZWF5	Eny2	724	891.3	1834.1	1911.3	757.9	959.4
D3ZWL6	Ahcyl2	451.3	395.1	190.4	173.6	362.1	438.4
D3ZWN1	Zpr1	42.8	51.3	55.4	83.3	60.7	55.9
D3ZWR1	Nt5c	251.3	278.4	177.6	183.5	230.9	241.8
D3ZWS0	Scrib	718.9	461.8	946.9	765.4	406.1	394.9
D3ZX38	Pfdn1	812.1	771.7	767.2	1189.6	858.8	855.2
D3ZX42	Rabgap1	338.6	397.5	584.6	620.3	512.4	485
D3ZXF9	Mrpl12	359.1	512.4	824.9	919.1	639.8	667.2
D3ZXH7	Alyref	486.1	534.7	321.1	356.3	652.7	618.9
D3ZXI0	Pycr1	2989.3	3073.4	2246.5	2143.9	1796.9	1775
D3ZXJ5	Efl1	634.4	437.7	726.5	779.9	462.9	440.2
D3ZXN8	Anxa10	112.5	101.8	100.5	107.4	118.3	109.2
D3ZXP7	Arpc1a	2156.4	2261	1499.9	1433.2	2025.7	1926.2
D3ZXS8	Ube2k	1666.6	1974.2	2456.7	2241.3	2204.6	2055.4
D3ZXT2	Stag2	111.9	134.9	247.4	255.3	288.8	246.6
D3ZXY2	Pdzd8	67.8	95.3	129.6	115.1	80	89.6
D3ZY40	Pcf11	86.6	104.4	204.9	201.2	142.2	136.3
D3ZY44	Mrps2	120.5	122.4	86.5	114.5	198	166.5
D3ZY71	NEWGENE_1586233	1919.9	2109.4	2342.7	2621	1681.3	1716.8
D3ZY96	Ngp	106.4	108.2	244.7	228.4	397.6	531.6
D3ZYL4	Mrpl50	176.3	242.8	424.8	402.8	247.8	234.5
D3ZYM5	Mtss1	35.6	50.4	43	36.8	58.3	54.9
D3ZYM7	Lyrm4	255	348.5	336.5	492.2	422.9	380.8
D3ZYQ9	Rnf20	223.7	300.8	883.5	703.4	408.4	347.4
D3ZYS7	G3bp1	812.7	878.2	764.5	899.6	965.5	972.4
D3ZYT8	Pikfyve	994.6	477.7	329.8	360.6	549.1	412
D3ZZ21	Ndufb6	600.1	800	693.3	583.5	668.1	749.5
D3ZZ38	Snx18	230.3	268.3	669.7	590.2	310.2	325
D3ZZ62	Xpot	258.3	271.8	216.5	226.4	243.8	238.1
D3ZZA8	Sec24a	592.8	699.3	786.5	740.1	754.2	762.2
D3ZZC1	Txndc5	5004.4	4648.5	4377.7	4579.2	4412.7	4753.3
D3ZZC5	Tradd	109.9	131.8	352.5	260.6	165	147.1
D3ZZH6	Map3k5	164.9	184.5	191.1	192	192.1	159.1
D3ZZL9	Gcc2	780.7	815.5	679.6	818.3	752.6	764
D3ZZM3	Cog4	643.9	718	928.1	908.9	695.1	662.7
D3ZZN3	Acss1	1203.6	1356.3	2066.4	2172.9	1309.7	1301.6
D3ZZQ4	Aamdc	312.5	353.8	432.8	417.5	346.5	322.7
D3ZZR3	Ctss	183	184.8	265.1	229.6	296.4	280.9
D3ZZR5	Snrpa1	417.6	438.1	597.2	501.4	526.1	553.5
D3ZZR9	Fkbp2	3757.9	4284.5	2125.9	2136.5	3369.5	3225.2
D3ZZT9	Col14a1	4952.6	4803.7	6553.1	6477.6	5963.1	6260.7
D3ZZW1	Dock1	529.3	553.9	489.6	485.7	601.5	584.1
D3ZZW2	Tma7	1742.5	1432.4	1513.9	1465.8	1292.1	1271.6
D3ZZY2	Utp14a	149.5	179.2	312.4	415.9	209.6	196

D3ZZZ9	Ctnnd1	983.8	1046.8	1196.4	1203.7	1458	1247.9
D4A017	Tmem87a	613.7	699.1	650.3	666.8	744.3	766.3
D4A022	Gapvd1	134.7	154.7	140.5	138.8	158.4	152.3
D4A031	Ddx42	81.7	88.2	124.3	134.6	118.7	125.4
D4A054	Ranbp2	1202.7	1506.4	1601.8	1576.5	1707.9	1683.2
D4A0C3	Hid1	1075.9	813.6	703.4	682.1	706.3	638
D4A0E8	Prmt5	987.9	754.1	763.8	918.9	800.6	771.5
D4A0F5	Sept7	3613.3	3296.3	3852.4	3974.6	3732.3	3905.3
D4A0H4	Cul2	287.9	353.1	523.7	511.7	325.1	351.1
D4A0T0	Ndufb10	4185.3	4587.4	3903.7	4262.9	3576.5	3360.3
D4A0T8	Dhrs7	237.5	225.8	281.1	235.4	257.7	230.9
D4A0W1	Emc4	356.9	333.2	251.1	282.1	292.9	299.7
D4A0Y4	Oxnad1	558	592.9	577.9	604	609.9	600.7
D4A133	Atp6v1a	1813.6	2102	2048.3	2179.1	2924	2773.5
D4A197	Mcee	398.2	470.6	837	652.3	489.9	486.4
D4A1A5	Ppp2r5c	48	57.5	62.7	68.8	63.8	70.8
D4A1B8	Dctn3	794.4	769.6	1137.6	917.7	860.9	741.2
D4A1J3	Palm3	283.6	327.6	593.7	508.7	256.4	221.5
D4A1J4	Bdh2	4469.6	4702.6	6073.1	5613.6	4568.3	3837.2
D4A1J6	Ankfy1	542.1	726.6	874.4	814.5	873.5	975.7
D4A1Q0		2498.6	1616.9	946.7	999.4	1236	1168.9
D4A1Q9	Ttll12	162.5	142.6	116.3	124.8	175.6	161.2
D4A1V7	Mob1b	409.4	545.5	414.7	466.4	907.1	898.3
D4A1Y0	Scyl2	495	489.7	606.6	774.5	544.2	506.5
D4A206	Tcof1	425.2	419.4	455.7	492.4	535.9	491.6
D4A259	Polr2d	43.3	42.9	49.2	50.3	63.8	63
D4A264	Zadh2	420.6	520.3	740.5	717.5	802.2	759.2
D4A2D7	Ipo4	432.7	464	605.6	661.2	510.7	476.7
D4A2G9	Ranbp1	2586.8	2623.6	2474.5	2368.9	2806.9	3031.3
D4A2H2	Sptlc1	75.3	86.1	119.3	116.1	105.7	106.4
D4A2H4	Dennd3	301	368	534.2	488.9	466.6	502.6
D4A2N2	Inpp5b	612.1	806.4	1082.2	887.6	913	890.2
D4A2P1	Ccar1	989.3	1099.1	889.9	1076.5	1160.5	1179.1
D4A3C2	RGD1304884	336.9	333.9	542.2	443.3	303.5	324.5
D4A3E1	Hnrnpll	823.8	900.7	930.9	838.3	944.8	887
D4A3E2	Npepl1	377.9	356.6	398.5	412.9	361.8	359.9
D4A3E8	Mrps27	194.8	226.4	283.8	349.9	213	197.6
D4A3G2	Cdk11b	92.2	119.6	156.8	115.7	148.1	139.1
D4A3I4	Btf3l4	1147.1	1284.4	567.9	479.5	1058.9	1068.7
D4A3I5	Fam160b1	97.6	128.5	87.2	88.7	106.9	108.2
D4A3K5	H1-1	1339.8	1435.6	1832.9	1909.5	2534.1	2565.7
D4A3P1	Ubqln4	281.1	338.4	373.8	373.5	383.5	349.9
D4A3S8	Nsun2	808.2	800.8	2077.2	1571.1	992.1	924
D4A3T3	Cbx1	246.5	239	220.9	191.6	306.2	294.1
D4A3V3	Parp12	281.4	375.8	1097.5	959.8	494.4	430.9
D4A3V5	Cgnl1	1061.1	1195.5	1079.7	1076.9	1196.3	1165.9

D4A416	Clptm1l	125.5	143.6	122.7	102.8	131.3	138
D4A465	Lamtor2	484.9	707.1	678	779.3	1155.2	1134.6
D4A471	Cmc2	77	108.1	95.2	82.7	85.4	82.3
D4A4J0	Supt16h	763.3	929.6	1330.3	1375.9	1289.7	1290.3
D4A4K4	Vps13c	1374.9	1388.6	2196.6	2168.5	1633.3	1532
D4A4S5	Folr2	336.2	406.5	400.6	372.8	736.1	794.9
D4A4T9	Chordc1	2056.9	2215	1838.2	1739.9	2683.3	2648.7
D4A4U3	Mdp1	199	266.4	289.5	233	287.6	272.6
D4A4X4	Cgn	590.6	633.1	424.6	451.5	593.5	603.6
D4A4Y0	Exosc4	33.6	40.5	141.2	125.4	56	53.7
D4A4Z0	Ccdc12	389.2	419.5	773.9	735.6	577.1	523.7
D4A4Z9	Ktn1	2254.7	2557.6	3268.3	2980.8	2881.8	2743.4
D4A510	Smarcc2	210.3	234.2	437.9	422.9	311	315.4
D4A511	Srp9	1093.1	1017.5	267.3	254.5	616.9	626.3
D4A529	She	51.8	72.6	86.3	80.1	95.7	77.6
D4A533	Tapt1	534	595.3	735.9	699	580.3	529.1
D4A565	Ndufb5	3136	3632.2	3217.5	3054	2784.8	2704.3
D4A5A6	Polr2a	838	951.9	1294.8	1254.8	1085.1	1100.8
D4A5I9	Myo6	2784.3	2853.5	3746	3767.7	3020.5	2648.8
D4A5K6	Zmpste24	1508.7	1220.6	1456	1473.9	877.4	907.4
D4A5S6	Alg1	596.4	419.8	185.9	160.4	255.2	237.6
D4A5T8	Ppl	590.2	692.9	699.3	676.7	831	777.4
D4A5X1	Stim2	44.4	47.6	86.1	77.7	57.5	53.6
D4A617	LOC103689954	1085	1213.2	1375.4	1520.8	1637.4	1527.5
D4A631	Arfgef1	500	610.5	1021.3	996.4	714	717.5
D4A648	Stk4	11.4	19.5	33.2	36.2	34	30.1
D4A678	Spta1	394	634.5	681.2	609.5	684.5	650.5
D4A6A2	LOC100911361	678.1	574.5	553.9	557.8	734.6	789.7
D4A6C5	Arhgap1	3396.3	3083.9	3094.8	3217.8	2622.9	2618.7
D4A6D9	Hs1bp3	44.7	46.6	106	96.2	47.1	41.8
D4A6P3	Shtn1	1470.9	1610.7	1967.8	1814.1	1418.4	1371.2
D4A6X4	Acyp1	403.9	474.9	446.8	404.6	556.6	526.1
D4A702	Synpo2	156.2	214.9	225	296.6	260	223.4
D4A746	Gmppb	2112.6	2399.9	1084	979.5	2067.9	2085.7
D4A772	Dtna	714.4	724.9	1312	1159.4	595.4	570.5
D4A777	Fam114a1	1089	1026.5	869.8	940.5	969.1	989.5
D4A781	Ipo5	942	1189	1536.3	1605.7	1412	1383.7
D4A7A8	Rab7b	133.9	157.8	135.3	147.9	279.8	265
D4A7B6	Tmem87b	442.9	430.7	395.1	412.7	399.5	384.9
D4A7F2	Mycbp	845.3	1003.2	2037.8	1653.8	1161.7	1235.5
D4A7G9	RGD1564804	170.2	139	73.9	132.6	147.4	129
D4A7J8	Prpf4	465.5	449.6	490.7	621.9	628.7	587
D4A7L4	Ndufb11	1039.7	1080.7	590.5	525.6	811.1	802.1
D4A7N1	Chchd6	282.3	264.4	269.4	289.9	189.8	224.3
D4A7R0	LOC108348180	837.9	932.1	516.1	888.8	813.3	796.5
D4A7U1	Zyx	425	444.9	766.8	774.1	583.8	617.8

D4A7X1	Mrps16	150.7	174.7	166.2	178.3	247.5	238.7
D4A7X5	Ppm1k	603.5	389.8	580.2	501.4	377.6	376.6
D4A817	Hist2h2be	337.9	334.6	1068.7	981.9	398	357.5
D4A830	Ppa2	1348.9	1420.3	1379.7	1208.1	1172.9	1134.7
D4A857	Ipo9	632.8	634.8	941.9	775.7	631.9	630.9
D4A8A0	Cad	1536.3	1556.2	1149.3	1086.3	1875.9	2005
D4A8E7	Commd6	247.8	224.1	417.5	319.3	263.8	293
D4A8F2	Rsu1	3525.7	4145.9	3680.8	3607.7	4980.3	4835.6
D4A8G5	Tgfbi	283.4	354	635.2	652.3	637.8	618.8
D4A8G7	Snw1	1429.8	1586.6	1894.1	1963	1629.5	1742.6
D4A8H3	Uba6	835.1	1061.3	1113.1	1155	1243	1265.4
D4A8H5	Ppp4r2	138.4	164	579.5	614.3	227	280.3
D4A8H8	Cyfip1	589.7	724.6	1046.9	992.8	1012.2	1083.6
D4A8L5	Mocs3	154.9	224.7	207.1	191.8	214.9	211.9
D4A8N0	Fam111a	481.5	207.1	541.8	331.6	277.3	349.6
D4A8N1	Dpm1	638.4	746.6	597.2	623.6	701.3	584.2
D4A8T3	Copz1	2062.5	2125.1	1354.1	1439.5	1552.8	1599.2
D4A8U7	Dctn1	2255	2107	2493.1	2441.5	2065.5	1975.3
D4A914	Xrn2	924.2	1242.5	1262.3	1156.6	1328.5	1291.8
D4A915	Ano1	101.2	144.7	219.7	214.7	156	167
D4A944	Snrnp40	1524.5	1757.9	1975.3	1845.7	2096.8	2124.7
D4A962	Hnrnpul1	472.9	442.7	803.4	908.5	553.9	572.4
D4A994	Emc1	1182.7	1449.7	1652.3	1602.1	1251.3	1247
D4A997	Htatsf1	753.8	868.3	1124.8	1043.9	1096	1004.6
D4A9A3	Cenpv	1017.1	913.6	1089.3	1022.2	1021.1	1036.8
D4A9B0	Srbd1	213.5	243	245.6	270.8	299.5	325.1
D4A9D6	Dhx9	1328.7	1401.3	1535.2	1494.4	1652.3	1618.1
D4A9D8	Osbp	4949.2	5193.7	3817.6	3513.4	4229.5	4331.4
D4A9K3	Dtd1	321.3	425.9	874.4	757.6	372.8	334.5
D4A9L2	Srsf1	550	513	842.5	943.1	566.4	580.2
D4A9L9	Erp27	11724.1	12026.8	7639.9	6665.4	5857.6	6950.1
D4A9M6	Akap1	87.8	114	239.6	223.9	143.4	153.7
D4A9N5	Trim25	2476.5	3191.8	3364	4237.5	3924.4	3820.1
D4A9P7	Bola2	114.1	123.4	119.7	106	187.8	191.9
D4A9Q3		1552.6	1496.7	1325.7	1386.6	1128.5	992.4
D4A9T6	Aqp12a	2346.6	1792.5	1719.4	1793.4	1372.5	1375.4
D4A9U6	Ubr2	1372.8	1365.2	1575.2	1468.9	1014.3	920.1
D4A9W3	Dglucy	726.1	708.9	371.7	401.8	490.9	447
D4A9Y0	Sdf2l1	845.5	979.5	749.6	746.9	894.6	847.8
D4AA35	Asmtl	498.1	540.9	292.7	402.2	603.2	534.3
D4AA52	LOC297568	9953.5	8598.8	14724.2	14565	8626	8493.7
D4AA54	Plekhh1	89.7	104.4	230.7	235.8	109	102.1
D4AA63	Ubqln2	831.6	1193.9	1691.7	1718.6	1125.8	1113.2
D4AAB5	Pm20d2	613.4	640.4	887.9	943.1	552.5	576.1
D4AAE9	Cisd2	1701.4	1284.5	566	593.2	972.9	1031.9
D4AAH9	Tbc1d23	101.3	167.9	125.7	105.2	118.9	107.6

D4AAI5	Cand2	26.2	39.1	76	75.7	81.8	62.7
D4AAU4	Rprd1a	30.2	38.9	48.5	52.7	43.6	36.8
D4AAV4	Arhgap17	58.4	90	252.5	262.8	110.5	128.4
D4AB01	Hint2	641.3	222.3	274.4	255.7	168.7	177.9
D4AB03	Fam120a	3250.6	3275.3	3373.8	3253.6	3129.4	3162.9
D4AB17	Pfas	1213.2	932.5	1053.6	929.6	829.6	890.7
D4AB70	Edem3	114.9	146.3	179.4	183.3	129.3	105.2
D4ABC4	Ppp4r3a	352.2	502.7	373.8	320.7	662.2	730.2
D4ABD3	Acap1	114	173.1	664.9	994.2	153.4	186.9
D4ABD7	Trip11	1771.1	1978.1	3169.9	2828.9	1986.5	1828.3
D4ABK7	Hnrnph3	307	352.2	583.2	589.8	421.8	397.9
D4ABM5	Mrps34	23.5	12.4	12.1	12.7	13.7	17.5
D4ABP4	Rab3gap2	148.2	194.8	235.3	230.6	221.9	254.2
D4ABT8	Hnrnpul2	2078	2134.9	2755.6	2830.9	2821.8	2598.6
D4ABX6	Mmrn2	161.6	175	110.6	99.8	116.5	104
D4AC23	Cct7	8024.5	8070.9	7399.1	7319.7	8196.6	8069
D4AC36	Eif3f	1662.6	1983	1838.9	1787.2	2009	1829
D4AC45	Agpat2	150.2	137.8	96.4	90.9	155.6	155.5
D4AC65	Coa7	308.3	307.9	364.2	417.3	292.9	282.9
D4ACB8	Cct8	8837.1	7590.2	6504.8	7093.2	7429.7	7972.4
D4ACC2	Kank2	547.2	536	596.7	540.7	702.8	673.4
D4ACG2	Ilvbl	436.5	457.6	505.2	553.5	509.1	479.3
D4ACK1	Nup214	93.4	129.8	175.6	255.9	114.6	102.6
D4ACK7	Cnnm3	31	39.9	31.4	36.2	51	39.8
D4ACL2	Ttc38	456.2	389.6	472.9	495.6	355.2	328.4
D4ACM9	Mfap1a	295.9	309.5	577.5	670.4	370.1	343.7
D4ACN6	Cert1	907	1042.7	975.8	1025.8	1198.5	1173.8
D4ACN8	Plgrkt	300.5	348.4	227.1	285.1	360.7	373.1
D4ACQ9	Gabpa	445.4	492.4	558	446.9	504	521.7
D4ACV3	Hist2h2ac	226.1	175.5	322.5	311.3	160.8	218.2
D4ACW0	Rbm6	233	237.9	243.8	222.3	325.7	307.5
D4ACW1	Nop2	877.7	855.6	1175.1	1313.9	1034.1	988.3
D4AD70		2835.1	2681	2112.2	2077.5	2189.7	1844.7
D4AD75	Dpy19l1	266.8	325.6	531.3	506.9	367.6	308.9
D4ADB4	Cggbp1	202.6	198.3	168.9	194.4	195.5	183.1
D4ADD7	Glrx5	294.7	309	118.3	111.5	292	229.1
D4ADD8	Dctn6	231.7	252.4	227.3	202.4	253.5	228.9
D4ADF5	Pdcd5	1392.4	1443.2	1410.2	1573.9	2068.6	1951.4
D4ADI4	Kif21a	113.5	156.6	73.5	85.9	137.7	159.4
D4ADP9	Copz2	2789.4	3136	2677.8	2913.2	2513.2	2577.5
D4ADS9	Efr3a	332.2	363.1	357.1	369.6	374.4	397.1
D4ADT3	Wapl	100.2	132.6	210.8	189.7	249.6	199.3
D4ADZ9	Pus7	108	140.5	319.8	252.3	180.2	156.9
D4AE02		264.2	353.6	356.2	499.6	437.4	441.2
D4AE06	Fkbp15	248.2	242.6	147.2	113.3	362.5	389.3
D4AE49	Mtrex	416.9	504.6	986.1	1013.1	654.8	605.1

D4AE65	Rrp7a	238.8	241	396.9	365.8	268.5	270.8
D4AEC0	H2afv	2750.4	3614.6	3214.1	3564.1	4931.2	4555.9
D4AED6	Mettl27	191.3	192	268.5	225.2	203	208.8
D4AEG2	Rab32	526.6	601	873	731	666.8	608.5
D4AEG7	Tbc1d13	48.7	69.6	79.9	87.3	154.4	153.4
D4AEH3	Psmd7	364	432.3	329.8	302.2	433.2	428.8
D4AEI5	Myef2	93.8	106.4	70.3	102.4	102.7	89.6
D4AEL0	LOC691083	85.8	104.4	109.2	91.4	101.4	88.3
D4AEP0	Adss	300.4	326.6	518	447.1	342.6	323.2
D7NIW0		92.8	117	101.2	93.6	161.2	158.8
E0A3N4		1312.3	1117.7	1130.3	1040.1	1914.6	1875.7
E9PSJ4	Spag9	880.6	919.4	963.4	990.8	1237.9	1078.6
E9PSP1	Pltp	121.7	120	177.2	225.2	216.1	207.2
E9PSQ1	Amy1a	3798.6	3691.4	1678.2	1370.5	1759.8	1312.7
E9PST0	Sept4	353.8	407.7	623	683.9	485.8	464
E9PST5	Acin1	750.1	667.5	744.8	813.9	880.5	843.8
E9PT04	Vps39	60.5	76.7	140.5	122.7	93	83.4
E9PT23	Slc38a10	140.4	163.2	168	166.8	224.7	199.7
E9PT51	Poldip2	1084	1295.6	1461.9	1463.6	1395.9	1354.4
E9PT66	Sf3b3	2914.8	3215.2	4359.9	4317.8	3485.9	3507.8
E9PT82	Strn3	256.4	287.9	240.8	215.7	218.8	206.2
E9PTA5	Pign	449.7	534.1	697.2	564.2	513.9	536
E9PTE1	Son	767.6	665.6	553.2	570.4	871.4	927.2
E9PTK9	Ankhd1	567.3	624.4	739.1	766.4	764.1	715.2
E9PTN4	Srpk1	330.5	427.2	777.1	682.9	448.1	432.9
E9PTU4	Myh11	2155	1910	1326.6	1269	2555.3	2757.6
E9PTV9		85.7	89	146.9	128.2	102.7	108
E9PTX9	Slc12a2	1421.4	1690.7	1357.8	1343.1	1560.5	1367.2
E9PU01	Chd4	446	524.5	635.2	717.1	623	615.9
E9PU07	Ear1	498.5	537.2	735	767.6	881.3	898.2
E9PU29	Rnf31	193.2	242.7	229.6	237.6	239.5	227.8
E9PU42	Vps26c	288.1	286.6	274.4	334.4	294.9	264.7
F1LM09	Usp7	1351.1	1653.2	2998.4	2897.3	1695.6	1616.2
F1LM33	Lrpprc	1667.9	1765.7	3169.7	2906.3	2650.8	2340.6
F1LM37	Sf1	1573.2	1453.5	1976.9	1756.2	1726.2	1608.9
F1LM55	Ccar2	814.9	886.7	986.1	1018.9	1057.7	1011.1
F1LM66	Eftud2	1730.8	2756.8	5807.4	5220.1	2781	2545.1
F1LM84	Nid1	3069.8	2451.6	2396.2	2369.7	2297.3	2193.5
F1LMA7	Mrc2	232.1	256.5	510	494.8	348.9	366.5
F1LMH5	LOC108348175	93.1	100	181.3	270.4	202.4	191.8
F1LML7	Hip1r	1781.8	1802.9	1973	1657.4	1685.8	1644.5
F1LMM5	Alox5	93.9	115.6	151.1	162.4	134.5	129.6
F1LMP9	Dab2	218.9	239.3	524.4	432.8	339.1	319.2
F1LMQ3	Psmd8	677.4	664.4	1097.5	969	693.9	675.8
F1LMV6	Dsp	1968	2134.2	1709.8	1832.6	2168	2312.9
F1LMV9	Coro2b	71.3	97.3	152.2	158.2	126.2	120.5

F1LMW7	Marcks	3359.2	3099.5	1790.1	1644.1	2857.5	3600.6
F1LN42	Tns1	409.1	414.1	756.5	727.6	584.5	534.4
F1LN59	Eif4g2	3039.6	3263.5	4632	3892.7	3344.4	3154.3
F1LN92	Afg3l2	1730.9	2068.5	1952	1925.2	2032.1	1953.4
F1LNA9	Clps	2505.5	1788.7	843.2	612.5	1204.8	857.8
F1LND7	Fdps	5868	6282.5	5148.2	5252.9	4501.5	4892.6
F1LNF0	Myh14	694.5	759.5	1278.8	1172.9	1080.1	955
F1LNH3	Col6a2	4692.1	4855.7	3486	3516.8	7178.4	7683.5
F1LNI5	Ppm1g	408.7	484.4	698.1	693.8	985.2	768.6
F1LNJ2	Snrnp200	4088.4	5045.7	5283.4	5764.8	6459	5965.5
F1LNL2	Smarca5	1050.4	1353.3	1905.3	2229.2	1798.5	1951.6
F1LNM0	Dlg1	542.9	566.4	992.9	906.3	553.4	585.6
F1LP05	Atp5f1a	27359.1	25413.8	34244.1	35043.2	20297.3	20435.7
F1LP30	Mccc1	9472.1	6980.9	8070.2	8635.5	4955.1	4747.1
F1LP46	Supv3l1	313.6	429.5	1752.8	1726.6	464.5	412.4
F1LP57	Map2k4	90.6	107.6	170.1	129.4	222.4	156
F1LPB4	Akap9	1058.3	1291.2	1735.7	1655.2	1317.1	1326.5
F1LPC6	Cpd	495.9	617.1	769.5	912.7	527.6	551.7
F1LPC7	Hdgf	1830.1	2051.1	3404.5	3403.5	2150.7	2354.2
F1LPD0	LOC108348074	1794.9	888	1342	1265	964.9	889
F1LPD6	Acaa1a	139.8	176.2	280.4	422.9	195.9	199.5
F1LPG9	Washc2c	284.1	318	222.7	248.7	395.5	384.3
F1LPM3	Sorbs2	2400.7	2391.7	2778	2589.4	2916.1	2865.8
F1LPQ3	Man2c1	110.4	144.3	198.2	253.1	162	130
F1LPV0	Nars	7970.4	10431.8	5828.7	5256.7	8132.5	9091.6
F1LQ00	Col5a2	176.7	224.4	283.1	280.3	340.2	332
F1LQ22	Use1	180.2	202.2	333.5	264	211.7	192.2
F1LQ48	Hnrnpl	6910.1	6976.4	6200.8	6037	7924	7860.5
F1LQH2	Nfkb1	486.9	543.9	483.9	448.5	615.5	614.8
F1LQI1	Hagh	1114.9	607.1	618	589.4	609.2	599.4
F1LQM9	Xpo7	625.8	716.8	915.6	890.6	619.7	586.4
F1LQP9	Tnpo1	706.8	764.6	350.7	462.4	1018.4	1084.2
F1LQS1	Mta1	65.4	66	75.3	81.3	120.4	101.9
F1LQS6	Xdh	2811.6	2824.7	3160.7	3491.6	3905.2	4068.4
F1LQT0	Dnajc25	1044.2	1265.1	1534.2	1437.3	1191.6	1173.7
F1LQX8	Itpr1	264.1	389.4	850.3	806.5	476.6	460.3
F1LQZ4	Casp1	432.8	498.2	501.3	448.3	529.3	613.8
F1LR10	Lima1	1125.6	1355.1	1995	2081.7	1645.4	1594.5
F1LR36	Supt6h	465.8	426.6	458.9	367.6	511.9	532
F1LR42	Rufy1	46.3	47	39.6	42.5	63.1	53.7
F1LR87	Hexb	325.6	216.9	266.4	366.6	173	173.5
F1LRB8	Mat2a	220.1	188.9	229.3	277.9	68.5	122
F1LRC2	Aif1	124.2	196	220.9	271.4	416.4	430.3
F1LRI5	Gcn1	9415	10287.4	12314.9	12909.2	10156	9481.2
F1LRI7	Aak1	846	855.1	632.2	563.2	806.3	831.2
F1LRJ9	Selenbp1	8551.9	7637.1	6810.8	7864.6	6930.1	6901.2

F1LRM0	Scaper	66.4	96.6	165	142.5	103.6	104.8
F1LRP7	Ago2	158.8	179.1	155.2	145.9	188.2	200.8
F1LRS8	Cd2ap	4555.6	4846	5866	5637.6	4693	4598.5
F1LRV4	Hspa4	8833.1	8998	10096.1	10052.6	9590.4	9503.6
F1LS29	Capn1	1082.5	1086.7	998.2	1012.1	1200.5	1182.1
F1LS86	Iars	6644.2	7644.3	6484	6432.4	7162.3	6804.9
F1LS93	Sh3bp2	501.5	521.6	719.4	643.9	539.7	492.8
F1LSD3	Itgb4	782.7	830.9	1588	1593.6	949.9	907.5
F1LSK6	Cyp20a1	68.8	60.4	72.8	74.9	72.8	66.5
F1LSP2	Acad10	38.7	49.3	41.4	40.8	39.2	40.9
F1LSQ0	Faf1	246.7	325.4	355.9	364.2	369.7	389.1
F1LSS1	Smc1a	2813.2	3176.5	3500.2	3346.2	3743.4	3535.8
F1LST0	Heatr5b	327.6	261.2	296.6	333.6	207.1	206.7
F1LT09	Wdr33	490.3	623.6	851	877.1	600.9	568.1
F1LT10	Afdn	180.8	225.9	324.3	320.7	313.1	314.9
F1LT35	RGD1564606	17591.7	17166.8	13761.7	13376.4	13133.1	12880.2
F1LT49	Lrrc47	1142.4	1000.1	1125.7	1188.4	1027.5	987.3
F1LTD0	Gpr89b	709.1	808.5	778.5	935.4	672.7	675.3
F1LTD7	Dennd4c	206.6	248	416.8	392	282.3	286.2
F1LTF8	Lama4	2002	2132.1	1809.1	1997.8	2399.5	2293.7
F1LTJ5		5549.4	5136.2	5717.4	6037	5657.1	5635.9
F1LTU4	Mrto4	1016	738.8	754.2	893.2	577.5	585.5
F1LU48	Ergic1	1460.8	1612.3	1260.3	1438.1	1328.3	1401.3
F1LU71	Auh	2141.4	1277.3	1602.7	2145.7	931.4	973.7
F1LV13	Hnrnpm	4936.5	5485.5	5497	5861.3	5603.9	5773.4
F1LV89	Rap1gap	466.7	523.9	715.3	607.5	433.5	418.8
F1LVF5	Ccdc149	292.2	395.2	598.5	580.9	425.4	410.6
F1LVK5		246	226.7	169.8	162.6	303.4	282.7
F1LVT5	Rundc1	322.3	378	495.5	452.1	345.5	350.9
F1LVV3	Ranbp9	41.4	55.7	65.2	74.9	75.6	61.6
F1LVV4	Rcc2	1003.6	1228.4	1201.9	1192.2	1902.5	1754.7
F1LVX1	Dnajc1	1248.5	1249.9	1111.3	1257.2	1148.5	1083.6
F1LW74	Iqgap2	2100.1	2722.7	5471.1	4692.3	3384.4	3422.8
F1LW91	Numa1	2462.9	2822	6550.6	4651.4	3471.6	3325.7
F1LWE6	Msi2	503.5	563.9	371.3	385.5	613.6	529.4
F1LWG4	Ndufaf1	303.8	289.3	286.3	273.6	261.8	241.6
F1LWK7		580.7	585	589.8	618.3	627.7	652.7
F1LX07	Slc25a12	2549.1	2994.7	3199.6	3239.1	2749.5	2728
F1LXA0	Ndufa12	757.4	735.5	609.1	586.7	659.4	646.1
F1LXF5	Get4	72	80.1	86.1	90.1	103.4	95.4
F1LXP8	Arhgap18	1173.4	1381.4	1767	1645.3	1388.9	1313.5
F1LXS5	Rcor1	44.8	51.6	68.4	57.3	67.1	61.9
F1LXV0	Rps6ka1	166.4	201.1	315.6	246.9	243.4	238
F1LY19	Upf1	757.6	827.1	1537.2	1804.9	931.3	887.6
F1LYQ8	Farp1	90.2	147.3	147.2	155.3	314.7	318.7
F1LYX9	Dsg2	107.4	148.4	160.5	124.1	327.6	128.6

F1LZ05	Parp14	289.7	319	365.1	400	594.8	651.8
F1LZF2	Fnbp1	581.4	561.4	763.1	727.2	1052.3	1049.2
F1LZJ4	Hyi	159	210.2	388.7	430.4	235.2	209.2
F1LZW6	Slc25a13	1623.8	1778.2	1310.2	1490.8	1571.7	1440.5
F1M062	Larp1	6701.1	6086.1	4326.2	4170.1	3624.6	3835.9
F1M0G5	LOC683674	198.8	208.8	217.7	256.3	230.4	239.1
F1M0U5	Nbas	1679.2	1665.4	2001.2	2126	1493.9	1523.3
F1M0X6	Magohb	1165.3	1441.7	2948.8	2632.7	1796.5	1779.3
F1M111	Myo5c	2181.4	2222.1	2995.5	2443.9	1816.7	1833.8
F1M124	Cobll1	569.6	634.1	607.9	467.2	584.1	561.4
F1M1A6	LOC681355	82	104	153.1	137.4	151.2	124.4
F1M1B3	Washc5	244.9	254.9	306.3	279.1	274.5	271.7
F1M1D5	Tbcd	598.1	691.3	1274.2	1515.9	705.1	678.3
F1M1H0	Dera	265.6	304.6	443.8	440.7	341.5	335.2
F1M1R8		485.3	645.9	715.3	673.1	582.5	573.4
F1M265		472.5	508.1	846.9	902.2	788.7	842
F1M277	Lrrc25	44.8	68	61.6	57.1	87.1	85
F1M2M6		250.9	274.4	391.2	451.1	263.7	289.8
F1M3H8	LOC681410	1087.2	1065.8	985.1	886.5	1138.5	1039.9
F1M3X3		54.7	65.3	251.5	299	109.3	115.7
F1M403	Ube2o	216.3	314.7	263.2	246.1	334.8	334.6
F1M446	Ecpas	416.1	494.2	629.9	702	576.3	525.7
F1M471	Epm2aip1	49.7	97.8	173.7	235.8	254.5	233.7
F1M4W7	Cstf3	325.8	354.3	620.1	535.6	446.5	416
F1M5R3	Abhd14b	1286.6	1488.5	1205.6	1412.1	1439.8	1294.3
F1M5V2	Glipr2	233.7	273.1	526.2	483.5	447.2	432
F1M609	Acox1	347.5	361	805.7	725	417.5	406
F1M614	Lama2	5869	5814.9	7252.6	7480.9	5806.9	5701.3
F1M635	Lbh	672.5	727.5	858.3	851.7	663.3	600.3
F1M6C4	Arl15	93.9	122.3	135	123.9	124.2	105.6
F1M6F4		3077.2	4908.6	3540.9	4197.9	5146.9	5114.5
F1M6Q3	Col4a2	2198.4	2318.6	3715.8	3957.1	3338.1	3344.2
F1M6V1	Hp1bp3	3898.5	3816.4	2505.9	2440.9	4243.2	4154.8
F1M775	Diaph1	2098.3	2764.8	2804.8	2996.3	3518.5	3052.2
F1M790	Ptgfrn	47.9	54.7	170.8	194	34.1	37.9
F1M7L9		188.6	261.5	234.8	240.7	318.4	303.6
F1M7P1	Dcaf13	27.4	38.2	72.1	64.4	48.5	40.6
F1M7S0	Magi3	288.9	338.4	176.7	155.7	332.1	350.2
F1M7S4	Cpa3	3195.3	4417.4	1874.1	1931.2	2694.4	2817.5
F1M7Y3	Rap1gds1	817.7	1095.2	1766.8	1472.5	1318.4	1269.6
F1M820	Sorbs1	356.9	363	847.6	687.1	385	365.7
F1M842	Tp53bp1	445.2	445.5	554.8	693.4	398.2	399.9
F1M853	Rrbp1	53439.5	49680	41715	38462.9	36244.6	36917
F1M8K0	Dag1	1429.8	1645.4	1794.7	1672.1	1783.6	1711.3
F1M8K7	Alg3	351.2	405.9	707.7	675.9	485.2	420.9
F1M8V2	Ube4b	379.4	447.4	1030.9	637.4	535.9	599.4

F1M8Y4	Deptor	200.1	238.4	211.3	214.7	242.3	228.2
F1M949	Ckap5	1474.2	2003	1866.1	1873.5	2272.6	2212.1
F1M951	Ptpn23	225.3	228.8	259.1	328.6	291.2	303.3
F1M957	Vwf	447.7	582.6	539.5	605	788.8	798
F1M983	Cfh	5159.4	5795.4	9952.8	9402.3	8163.8	8565.4
F1M9C7	Ctdspl2	149.6	205.4	118.6	127	246.9	265
F1M9C9	Hars2	467.2	539	1094.5	901.6	492.7	509
F1M9V7	Npepps	1325	1512.8	1784.6	1518	1655.5	1620.4
F1M9W9	Trappc8	145.9	188.6	238.3	306.8	211.8	210.2
F1M9X2	Gp2	4211.9	2494.5	3534.5	3509.4	1956.9	1988.7
F1M9X5	Prkar1b	448.7	595.9	529.6	444.7	510	493.7
F1MA18	Ybx3	484	469.2	268.3	264.6	677.5	463.6
F1MA56	Ctrb1	63345	54264	29412.5	29158.6	48409.6	51691.5
F1MA59	Col4a1	246	255.3	387.5	326	349.8	312.4
F1MA98	Tpr	1966	2160.1	2516.9	2495.6	2752.1	2748.9
F1MAA1	Usp47	501.9	522.8	414.7	501.8	533.4	478.3
F1MAA3	Ppp2r5d	57	79.5	78.3	73.8	119.7	113.4
F1MAA7	Lamc1	12471.9	10957.7	10592.1	10717.4	11669.7	11423.7
F1MAB9	Tpd52	833.9	1299.6	963.2	866.8	1259.2	1153.2
F1MAF5	Pcid2	854.5	542.6	660.6	738.5	629.1	542.2
F1MAN8	Lama5	1567.6	1633.2	2556.5	2561.2	1808.9	1713.9
F1MAQ4	Trappc10	70.7	85.5	78.5	100	92.8	82.6
F1SW39	Psip1	2553.2	2720.6	3381.1	2911.8	3340.7	3277.5
F2Z3Q8	Kpnb1	2163.8	2864	3941.2	3679.8	4063.2	3987.8
F2Z3T0	Cdc73	54.9	73.4	104.8	117.7	77.7	83.7
F2Z3T4	Mbnl2	183.7	133.6	58.4	50.1	92.5	106.3
F2Z3T7	LOC103694869	1029.6	1157	1212.4	1582.7	1071.4	927.2
F2Z3T9	U2af2	224.1	451.3	339.7	1007.5	875.9	736.5
F5CC78	Dmd	590.2	639.5	819.2	788.4	615.5	638.1
F7EL36	Anp32a	2250.2	4139.1	2722.4	3247.8	5337.3	5215.2
F7EMB2	Rtraf	945.5	1393.9	1661.3	2187	1829.7	1817.5
F7ENH8	Hdac2	29.7	36.9	71.9	69.6	42.4	40
F7EPE0	Psap	1599.5	1753.2	2609.1	2690.2	3519.7	3304.4
F7EPH4	Ppa1	4701.1	5409.4	4878.5	4627.5	4863.3	5010.2
F7ES73	Nub1	226.1	269.3	230.3	206.8	323.1	337.1
F7EV94	Bckdha	3602.1	4389.7	2635.7	2630.7	2529.3	2610.6
F7EVX2	Eif2b2	1303.9	872.4	950.8	1068.2	743.4	675.2
F7EZ89	Tbc1d15	771.2	849.5	1425.5	1588	856.1	830.2
F7F0B1	Zc3hav1	235	324.9	201.2	221.9	350.5	368.5
F7F2F3	Hspa4l	351	461	728.6	717.7	451.7	397.8
F7F350	Syap1	1361	1573.8	1188.6	1094.2	1554.4	1312
F7F3Z1	Lman2l	460.9	311.7	641.1	642.5	259.9	211.4
F7F5J1	Tfeb	16.4	17.2	8	7.2	19.3	22.9
F7F707	Edc4	1358.8	1430.7	1587.6	1640.3	1554	1556
F7FG68	Syvn1	429.3	563.8	330.7	342.3	341.9	282.8
F7FJQ3	Npc2	1001.8	1038.7	1587.6	1470.1	1759.1	1792.1

F7FLB2	Pgm2	3053	3006.1	3693.1	3650	3786.1	3469.9
F7FM32	RGD1311345	144.3	185	139.6	135	189	167.2
F8QYX0	Chga	202.9	228.2	404	527.2	209.6	200.4
F8WFF9	Lrrc57	129.9	202.4	274.9	283.5	243.3	232.9
F8WFK6	Gpx4	1606.1	954.1	1939.6	2254.2	1176.1	1030.3
F8WFT7	Slc4a1	336.6	497.6	329.8	303.4	500.1	476
F8WG67	Acot7	648	699.7	755.1	708.1	1060.1	1003.7
G3V617	Mapk14	971.1	1193.4	2421.4	2148.6	1818.6	1656.9
G3V618	Mapk13	40.4	52.4	51.3	48.1	68.7	51.4
G3V624	Coro1c	3669.7	3902.5	4698.6	4815.4	4415.3	4484.1
G3V631	Rabgef1	441.6	577.2	883.1	811.3	526.2	646.5
G3V643	Tff2	460.5	214	278.3	264	91.3	84.3
G3V644	Ndufv3	1804.6	1792.2	1155.7	1045.3	1502.7	1400.9
G3V647	Pdxk	1435.1	909.6	695.6	778.3	833.9	758
G3V648	Agpat3	588.7	615.9	496.9	525.6	730.2	711.5
G3V662	Nup153	31.5	40.8	60.7	51.9	57.1	45.9
G3V667	Itga6	1348.3	1452.5	1788.8	1851.4	1559	1455.3
G3V678	LOC100912534	110.6	163.3	318.8	259.4	177.9	175.4
G3V681	Mcm4	694.6	762.8	989.5	1044.1	1201.8	1247.5
G3V684	Med15	25.8	50.3	171	156.3	65.8	64.5
G3V6B0	Pdxdc1	3676.6	3712.9	3137.2	3816	2928.5	2633.9
G3V6C4	Ugdh	555.1	565.7	839.8	855.2	1179.6	1168.1
G3V6C9	Cdc42bpa	127.5	182.6	447.5	473.9	250.6	216.3
G3V6D9	Slc9a3r2	357.2	395.5	412.7	371	419.9	386.1
G3V6F5	Elac2	638	769.6	877.8	790.8	781.2	778.5
G3V6H0	LOC100363782	738.1	596.3	993.4	1005.9	605	534.3
G3V6H2	Prpf8	2401.4	2658.3	5232.4	5867	3254.1	3023.5
G3V6H4	Chrne	721.3	667.2	343.1	399.6	575.6	485.7
G3V6H5	Slc25a11	353.2	749.1	448.2	344.3	342.5	364.2
G3V6J2	Soat1	461.2	599.9	585.5	639.3	840.9	765.2
G3V6K1	Tcn2	598.3	649.9	1461.5	1183.1	668.1	641
G3V6K3	Exosc7	170.7	110.9	150.8	156.9	114	93.2
G3V6L8	Strn	203.5	246.1	191.8	202.4	253.7	244.9
G3V6L9	Fkbp3	4227.8	3917.1	3280.7	2980	3229.9	3258.2
G3V6M4	Capn6	198.2	273	288.9	398.4	305.9	292.3
G3V6M8	Nup37	121.7	141.7	196.6	220.3	196.9	194.4
G3V6N2	Tmed4	456.3	369.4	855.4	954	349.7	313.8
G3V6P5	Gcg	1657.8	1294.8	1759.2	2337.3	1510	1444.9
G3V6P7	Myh9	40793.7	40353.6	46567.9	50782.3	57684.5	60236.8
G3V6P8	Gng12	497.6	605.7	535.1	575.3	974.1	871.1
G3V6R7	Oxsm	187.9	259.9	296.4	278.9	301.8	284.7
G3V6S0	Sptbn1	13916.1	14501.8	11917.8	11893.3	16437.4	16966.7
G3V6S1	Pawr	760.9	806.7	1076.7	929.2	777.5	767.2
G3V6S2	Aco1	7816.9	7329	7994.4	7778.3	7064.3	7189.4
G3V6S3	Calu	2598.4	2854.7	2234.6	2256.2	3661.5	3514.3
G3V6S5	Mthfd1	5089.8	5466.5	5467.7	5316.9	5124.2	5076.7

G3V6S8	Srsf6	561.2	519.8	611.8	591	667.9	622.8
G3V6T1	Copa	14047	14055.8	11564.4	10912.8	11868.4	11691.3
G3V6T7	Pdia4	24157.2	23179.2	16463.3	17139.5	20462.4	21168.7
G3V6U3	Alg2	2056.6	2352.6	2412.5	2383.6	2040.9	2041.4
G3V6U9	Setd3	291.3	319.1	742.3	617.3	381.8	337
G3V6W6	Psmc6	1594	1739	2250.7	2251.4	1735.1	1694.3
G3V6Y6	Pygb	1695	2153.2	3313.4	2920.6	2468.9	2401.3
G3V6Y7	Caap1	76.1	87.8	67.3	65.2	98.9	99
G3V6Y9	Ppig	299.8	333.5	385.4	441.1	440.2	455.9
G3V709	Naprt	666.8	877	1807.3	1952.8	1067.2	1083.9
G3V712	Krt7	638	739.1	1354.3	1171.7	1270.9	1537
G3V727	Ddx47	41	53.3	75.3	136.2	65.8	63.1
G3V757	Fut4	58.1	91.6	186.1	151.5	89.7	98.6
G3V784	Adpgk	781.4	882.3	999.8	783.5	758.7	750.6
G3V7A3	Serpini2	13210.8	11159.2	9365.2	9886	8792.9	8837.3
G3V7C6	Tubb4b	2810.9	3218.2	1275.8	1384.7	3672.8	3431.7
G3V7F6	RGD1561590	629.9	561.2	437.9	506.9	568.1	591.1
G3V7G0	Dync1li1	922.4	866.2	653	574.7	1042.3	1035.3
G3V7G9	Eif3l	2304.9	2730.1	2207.4	2376.3	2706.8	2377.6
G3V7I0	Prdx3	5158.5	5330.3	2571.3	2958.8	3854.2	3959.9
G3V7I3	Atp13a1	3407	3610.5	3226	3945.2	3235.3	3209.4
G3V7I8	Slk	735.4	670.9	959	850.9	659.3	683.8
G3V7J0	Aldh6a1	12950.3	12754.9	7949.1	8351.3	8013.6	8158.3
G3V7J2	Prkra	772.4	797.8	945.5	1021.8	1029.7	951.9
G3V7K5	Npc1	739	742.5	1050.8	959.2	1194	1160.3
G3V7L6	Psmc2	3214.9	3418.6	4062.1	3982.4	3518.7	3547.1
G3V7L8	Atp6v1e1	394.2	449.5	412.2	390.4	562.5	533.9
G3V7M0	Cnot1	390.2	497.8	624.4	656.8	744.3	607.3
G3V7P1	Stx12	678.4	531.9	452.5	459.6	704.9	615.9
G3V7P6	Nudt16	94.6	109.1	139.2	145.1	127.1	138.6
G3V7Q4	Ptpn12	395.9	438.7	708	655.6	627.6	599.1
G3V7Q6	Psmb5	3011.2	2944.7	3529.5	4054.4	2855.5	2691.4
G3V7Q7	Iqgap1	10936.8	12627.9	19013.8	19339.6	18870.7	18338.2
G3V7R1	Nup50	130.5	129.2	220.4	243.9	150.9	142.4
G3V7T3	Pank4	276.7	357.9	342.4	353.9	382.9	345.5
G3V7T5	Xylb	60.1	82.8	102.1	98.6	103	95.6
G3V7T6	Sf3b1	3914	4457.7	5913.1	5232.9	5039.5	4994.2
G3V7U4	Lmnb1	9374.6	8351	9566	9405.9	9618.3	9822.5
G3V7U9		437	402.9	451.4	480.5	428.4	379
G3V7V5	Fkbp11	6342.1	6384.6	3989.3	3900.1	4719.1	4986.4
G3V7V6	Retsat	982.7	973.7	933.2	1001.6	1002.5	1146.8
G3V7Y3	Atp5f1d	4491.3	3789.4	2989.1	2741.1	2686.4	2756.9
G3V7Z8	Pabpn1	1041.2	1113.5	648.2	653.3	1279.9	1287.8
G3V811	F13a1	1729.7	1925	2166.4	2223.4	2706	2810.8
G3V816	Nme3	1292	1367.9	1994.3	1891	1098	1055.6
G3V818	Parva	809	860.5	947.8	1126	1067.8	993.9

G3V824	Igf2r	1649	1719.7	2323.9	2219.8	1744.4	1702.4
G3V826	Tkt	18984.4	19222.6	14561.9	17864.7	22696	24406.4
G3V827	Kyat1	809	996.8	1608.9	1717.8	1004.5	990
G3V829	Fubp3	227.4	297.8	622.8	540.9	348.1	310.2
G3V836	Clu	5051.1	5484	9834.4	10758.9	23634.9	28947.4
G3V837	Cd1d1	523.6	539.5	559.2	525.8	534.3	540.8
G3V843	F2	1854	2183.8	1962.3	2010.3	3344.3	3399.4
G3V844	Amy2a3	54785	36094.6	26351.5	26433.1	21228.6	22321.5
G3V852	Tln1	10454.1	10859.1	17709.8	15754.8	15464.9	15231.7
G3V886	LOC108348078	360.5	338.9	391.2	458	377	336
G3V8A4	Mtr	179.9	199.2	207.6	251.3	199.8	192.3
G3V8A5	Vps35	1852.3	1737.4	1481.8	1349.1	2175	2023.8
G3V8A7	Pnlip	121.4	2258.2	1671.3	975.1	895	166.9
G3V8B4	Brix1	209.1	231.5	230.7	236	264.7	249.3
G3V8B6	Psmd1	1775.8	1751.8	2249.3	2200.3	1863	1801.7
G3V8C0	Dctn5	79.5	85.9	97.7	79.3	94	94.5
G3V8C4	Clic4	457.5	546.8	509.3	538.4	749.8	710.1
G3V8C6	Sra1	136.9	191.2	111.7	98.4	244.6	216.7
G3V8D4	Apoc2	428.1	583.5	1159.6	916.5	557.8	631.5
G3V8D5	Pgls	1587.3	1571.6	1022.9	944.5	1347.1	1426.8
G3V8E2	Strip1	197.8	210.5	193.9	202.2	269.3	261.8
G3V8E4	Ubfd1	562.1	621.3	1071	934.8	664	753.9
G3V8F5	Tomm40	617.8	849.8	867.3	828	1005.9	909
G3V8F7	Gga2	419.8	315.1	582.1	542.7	370.4	340.9
G3V8G2	Psmd5	959.6	1021.7	1295.1	1292.4	851.8	919.2
G3V8G5	Glg1	3790.2	3840.7	3475	4024.1	4662.8	4901
G3V8H5	Ikbkb	69.2	69.4	83.8	88.5	79.9	82
G3V8I4	Stx4	583.7	652.7	650.7	570.4	730.3	745.8
G3V8J3	Ctrl	14458.7	10941.2	7104.5	7112.1	5970.1	5584.9
G3V8L1	Pycard	918.4	1051.8	1512.3	1396.4	1713.6	1626.1
G3V8L3	Lmna	29855.7	26718.2	26075.5	26519.5	31864.9	32506.9
G3V8L7	Itgam	823.6	1007.2	1451.8	1517.3	1624.4	1768.4
G3V8M1	Pold1	43.3	58.5	75.5	81.5	79.5	67.5
G3V8N0	Sfxn2	242.5	228.6	152.2	182.9	134	122.4
G3V8P4	Ptprf	384.6	432.6	1327.3	1205.1	515	464
G3V8P5	RGD1310127	649.3	817.6	1168.9	1019.5	720.6	687.6
G3V8Q1	Cope	2231.1	1387.2	1797.5	1819.4	1235.2	1179.6
G3V8Q8	Sec23ip	3855.1	3783.3	4561.1	4192.1	3291.8	3296.9
G3V8R0	RGD1311703	797.2	918	664	715.9	742.6	880
G3V8R5	Lipe	77.1	44.3	43	62.6	50.6	43.9
G3V8S0	Spint2	94	107.9	200	203.8	180.7	174.5
G3V8T4	Ddb1	2057.9	2295.1	2568.6	2371.1	2786.6	2772.6
G3V8U3	Dpf2	631.6	625.3	1000.5	902.2	748.1	806.5
G3V8U9	Psmb4	1383.2	1158.4	1262.1	1272.7	1107.8	1054.5
G3V8V1	Grn	909.3	945.4	2016.5	1695.2	1733	1649.8
G3V8V3	Pygm	108	133.9	143.3	156.3	136.4	134.8

G3V8Y5	Polr2b	52.1	54.4	127.7	133	47.4	52.8
G3V8Z9	Cops7a	732.1	699.1	998.2	1547.7	709.5	722.1
G3V918	Gart	597	639.8	924.5	926.8	790.8	775.5
G3V920	Wdr43	289.6	441.4	288.6	287.5	464.9	451.9
G3V928	Lrp1	1245.2	1269.1	1951.5	1958.2	1977	2062.8
G3V969	Tfcp2	51.5	74.3	120.9	144.9	79.5	81.2
G3V976	Cpa2	32118.9	32680.2	20922	22223.2	16822.7	15480.2
G3V982	Elmo2	202	242.3	240.3	244.7	289	293.6
G3V985	Sco1	871.2	589.7	1307.2	1371.5	326.3	303
G3V9C9	Zmiz2	299.9	303.6	450	373	402.2	383.2
G3V9D1	Plcd1	88.1	98.8	76.7	75.3	102	127.2
G3V9D3	Sel1l	2181.5	2171.9	2972.6	2522	1999.6	1715.9
G3V9E5	Sts	334.9	376.3	344	364.4	375.5	343.4
G3V9H0	Rasa1	85.8	96.9	92.2	97.6	151.4	130.3
G3V9J1		158.9	158.2	191.4	241.9	101.6	107.1
G3V9K0	Cars	7581	7767.7	7432.9	7128.4	5622.8	5445.1
G3V9K8	Rbm45	216.9	222.8	391.9	480.9	319.4	304.7
G3V9M1	Ddx23	615.9	635.2	437.2	415.3	615.6	705
G3V9M6	Fbn1	27488.3	25543.7	24294	24839.9	30079.4	32004.1
G3V9N0	Pabpc4	2297	2220.6	1296.9	1427.2	1513.5	1627.6
G3V9N8	Ap1b1	3069.8	3179.2	3768.7	3909.4	3296.9	3330.6
G3V9P0	Psmd9	416.6	212.3	314.3	243.3	229.6	228.9
G3V9P7	Htt	113.2	142.6	202.1	194	184.7	138.3
G3V9Q4	Stk38	181.3	254.4	181.1	200.4	288.1	265.3
G3V9S9	Sec24d	4187.7	4690.1	5017.2	4734.3	3673.2	3681.8
G3V9U2	Acaa2	4164	4552.9	3846.9	3862.3	5008.1	4540.4
G3V9W0	Cetn2	378.2	357.2	409.7	424.6	352	401.4
G3V9W6	Aldh3a2	2654	2627.6	3449.8	3905.3	2259.2	2449.6
G3V9Y1	Myh10	4219.7	4386.7	4290.1	4614.2	4402.9	5134.2
G3V9Y9	Ap3s1	61.3	65.3	74.4	48.9	66.2	58
G3V9Z6	Sept8	1206	1198.6	970.7	948.7	1409.8	1433.9
H6X320	Apcs	717.1	700	1456	1338.9	717.1	686
H9KVF6	Stk10	309.7	346.4	549.3	568	529.1	504.1
I6L9G6	Tardbp	1181.3	1259.3	826.1	882.3	1196.3	1270.6
J7P1Z1	Irgm2	78.1	100	106.7	175.9	422.2	364.2
M0R3M8	Rrp12	211.2	258.3	475.4	459.8	301.6	315.8
M0R3S1	Mrps21l	129.1	136.9	107.6	100.2	145.9	135.5
M0R3V4	Mydgf	1921.1	1414.2	573.8	447.9	781.3	838.7
M0R3Z8	Rbm15	220.1	222.1	497.1	452.3	289.8	281.4
M0R4F8	Dnmbp	355.7	484.8	370.3	311.3	396.2	375.4
M0R547	LOC100910181	728.9	1021.5	4280.2	2038.7	1697.9	1374.8
M0R565	Wdr82	576.1	598.7	1379.7	1219.4	852.3	762.6
M0R5B6	Exosc1	117.6	130.2	337.6	374.7	178	163.2
M0R5F8	Hmgn5	3256.1	3651.4	1717.4	1543.3	3665.7	3754.8
M0R5H1	Etl4	234.6	253.8	416.8	410.1	360	343.2
M0R5J4		10986.9	10563.4	12180.3	12803.2	13101.8	12851.7

M0R5M5	Jrkl	483.8	465	377.4	619.5	281.4	232.2
M0R5N4	Pfdn4	569.5	588.2	782.6	824.8	663	636.6
M0R5Q3	Ranbp3	436.8	573.7	854	765.4	602.8	617.9
M0R5W4	Mvk	420.7	433.1	699.9	712.5	456.7	458.3
M0R623	Gtpbp4	411	517.5	1083.6	1085.7	637.8	606.5
M0R660		427.9	501	496.9	493.4	615.1	676.4
M0R6E0	Atp9a	834.1	929.6	1271.2	1124.6	1029.8	942.2
M0R6H1	LOC688286	576.7	515	733.4	642.3	421.9	450.2
M0R6L9		633.1	800.5	854.2	939.5	1641.9	1694.7
M0R6T1	Tatdn1	599.1	672.3	600.8	611.7	615.5	586.1
M0R735	Syncrip	3396.7	3640.6	2535.2	2420	3137.4	3154.7
M0R757	LOC100360413	23334.8	23861.8	16632.7	15427.8	17604.7	17801.5
M0R776	Mrps36	241.3	244.6	148.3	145.1	223.8	230.3
M0R7A6	Itsn2	702.2	740	1356.9	1271.7	805.3	931.1
M0R7E6		492.5	486.1	508.1	499.4	656.5	609.4
M0R7G4	Apoo	468.9	284.7	405.6	403.4	203.6	197
M0R7I0	LOC100359600	135.8	155.6	247.9	260.6	230.4	221.3
M0R7W3	Xaf1	172.8	238.6	139.6	145.9	393.2	307.8
M0R7Z0		4390.7	4869	4797.5	4569.3	4240.4	4191.6
M0R851	Usp4	56.3	67	75.8	118.7	81.8	86
M0R8A4		3449.4	2393.1	1868	1713.7	1683.7	1700.4
M0R8U1	Dnah5	203.9	285	2356	2077.7	186.7	160.1
M0R907	Snrpd3	1243.9	877.7	978.5	1110.1	900.2	879.1
M0R959	Mmab	141.3	183.1	355.2	336.4	192.1	160.5
M0R961	Khsrp	1027.2	843.2	1319.3	1287.4	900.2	958.3
M0R9B9	rCG_52671	133	150.3	114.7	136.2	174.9	197
M0R9L0	Naca	3213.5	3787.7	2684.9	2931.7	3872.1	3871.1
M0R9L3	Snx9	1559.4	1576.2	1885.8	2036.3	1906	1814.8
M0R9Q1	Rbm14	441.5	516.1	876.4	1180.3	829.5	646.7
M0R9X8	Dync1h1	25124.2	26379.2	31079.2	31591.4	27713.9	26961
M0R9Y3	Nup43	119.3	112.6	198.2	145.3	191.8	183.4
M0R9Z5	Irf2bp2	105	133.1	119.9	120.9	139.2	119.9
M0RA08	Plin3	823.4	690.2	1316.6	1170.9	854.7	834.3
M0RA15		46.2	62.1	120.9	135.6	69.8	68.3
M0RA79		91.9	113.3	294.4	303.8	199.1	200.5
M0RAD5	Clpp	1422.3	1517	1568.6	1643.3	1328.2	1263.9
M0RAI4	Ears2	261.4	309	608.6	607.1	302.5	291.3
M0RAK2	LOC684270	2496.7	3317.6	2329.4	2027	2572.8	2364
M0RAP5	Sbf1	78.9	83.8	83.8	85.1	86.7	81.5
M0RAQ6	Hk1	46.4	85.9	68	76.1	113.6	125.9
M0RAV6	Arhgef10l	34	68.7	73.5	87.1	117.6	109.7
M0RAY7	Wdr36	171	225.7	307.4	340.3	259.8	247
M0RB46	Phf11	188.6	234	461.9	346.7	240.6	239
M0RBF1	C3	12456.6	14214.5	24506.2	25823.9	18069.3	19940.8
M0RBF8	Exoc4	319.1	253.6	196.4	234	193.3	199.3
M0RBI3	LOC102553099	142.5	184.5	189.1	203.8	220.2	207.6

M0RBT5	Suds3	101.1	146	264.4	259.6	172.5	143.3
M0RBV9	Nup214	364.1	617.2	833.2	625.8	577.4	579.5
M0RC47	Pik3r1	121.6	152	298	254.7	202.2	180.2
M0RC57	Smap1	161.2	130.8	225.2	127.4	156.3	153.3
M0RC73	Naa16	77.5	106.7	217.7	172.4	111.2	107.3
M0RC77	Afmid	63	79.4	73.9	97.8	110.3	91.5
M0RC99	Rab5a	221.1	233.9	305.6	327.2	259.1	258.7
M0RCH0	EIF3I	5258.2	5680.1	3245	3496.3	4072.2	4354.3
M0RCH5	Gnpda1	225.6	255.6	156.8	154.1	320.3	370.8
M0RCH6	Chmp4b	873.6	788	640.4	637.4	643.2	755.9
M0RCH8	Rsl1d1l1	941	866	533.5	571.2	908.3	922.1
M0RCX0	Pcbd2	169.3	235.2	206.7	221.9	238.3	174.4
M0RD20	Capns1	77	105.1	158.8	154.7	314.9	212.7
M0RDJ4	Gmfb	1214.3	861	794.7	678.1	899.2	938.6
M0RDK9	Acad8	5184.4	4252	3621.7	5140.4	3043.9	2939
M0RDM7	LOC100910143	96.8	114.6	142.8	142.3	113.4	104.2
M0RDW3	LOC102556148	1102.1	1045	1892.9	1702.3	821	725.6
O08557	Ddah1	10993.9	12271.2	12268.4	11330.3	9737.8	8969.6
O08629	Trim28	2306.8	1789	1916.9	1960.4	1531.3	1595.2
O08651	Phgdh	4524.1	4315.2	4438.2	4135.7	3455.8	3365.5
O08658	Nup88	316.4	324.2	227.5	216.3	433.9	436.9
O08700	Vps45	350.7	344	820.8	713.5	515.6	471.9
O08701	Arg2	108.4	117.9	97.5	78.5	93.8	45.2
O08719	Evl	256.9	283.3	127.3	158.8	499.8	538.5
O08769	Cdkn1b	365	427.8	555.5	563	536.6	568.8
O08816	Wasl	794	784.6	805	740.3	742	761.6
O08836	Igbp1	270.1	277.2	453.7	602	344.7	307.1
O08839	Bin1	687.1	746.3	596.5	640.3	1013	841.3
O08984	Lbr	87.8	89.6	538.8	425.6	171.3	179.5
O09175	Rnpep	5574.9	6386.2	6464.1	5621.7	5500.5	5313.8
O35077	Gpd1	2051.3	1741.7	1347.5	1242.9	1940.1	2024.7
O35094	Timm44	65.5	95.3	117	109.1	84.6	89.5
O35112	Alcam	2490.8	2681	3358	3167.7	2520.6	2560.3
O35162	Hspa13	2015.9	2008	1474.5	1392	1453.2	1408.3
O35244	Prdx6	3133.4	2326.7	1878.3	1862.4	3021.8	2559.9
O35263	Pafah1b3	183	231.1	357.8	323.6	270.2	267
O35274	Ppp1r9b	66.4	74.8	40.7	49.5	113	112.4
O35303	Dnm1l	880.5	975.9	2928.9	2176.5	1069.1	997.2
O35346	Ptk2	59.7	88.5	127.7	137.6	118.9	114.7
O35358		176.2	212.5	140.5	117.5	224.9	243.2
O35394	Rabac1	383.9	415.7	1429.9	808.5	376.7	371.4
O35397	Casp6	1941.4	1940.8	1755.8	1640.5	1484.7	1426
O35532	Msmo1	499.8	695	889.2	775.7	546.5	602.7
O35547	Acsl4	151.6	195.1	250.9	213.3	222.4	226.8
O35567	Atic	2919.7	3088.3	3084.3	3679.4	3178.8	3013.4
O35775	Sycn	8545.7	5452.7	2876.2	2766.3	2705.4	2844

O35796	C1qbp	2280.9	2051	1904.6	1916.8	1979.2	1893.6
O35814	Stip1	9518.7	9918.3	9986	10854.6	10216.9	10265.6
O35815	Atxn3	66.7	95.2	100.3	132.2	114.5	104.9
O35820	Dnph1	452.1	612.8	510.7	646.1	437	445.4
O35821	Mybbp1a	2667.1	2631.4	3204.7	2651	2632.1	2761.3
O35824	Dnaja2	1628.9	2075.7	1452.3	1317.7	1875.7	1867.3
O35826	Gne	137.8	132.2	189.7	167	146.2	123.7
O35828	Coro7	982.1	1050.2	927.2	1074.5	1303.2	1365.6
O35854	Bcat2	14073.4	13157.8	14772.5	14294.2	9445	9525.9
O35964	Sh3gl1	255.1	264.2	315.6	379.3	387	382.9
O35987	Nsfl1c	2768.4	2321.5	1908	1853.6	2581.8	2347.7
O54748	Stk3	74.1	86.6	166.4	158.2	103.4	100.2
O54772	Smarcd2	216.1	242.3	330.5	320.1	276.8	254.6
O54861	Sort1	304.4	342	471.1	510.5	356.8	337.4
O54921	Exoc2	498.1	678.7	610.7	643.9	969.8	985.8
O54922	Exoc7	794.2	621.9	431.2	507.1	605.6	696.3
O54924	Exoc8	359.5	489.5	463.7	440.7	541	463
O54975	Xpnpep1	1598.5	1657.5	1325	1422.2	1708.5	1735.4
O55004	Rnase4	128.4	135.3	196.2	203	217.6	215.7
O55096	Dpp3	3075.8	3342.6	4860.2	5167.6	4364.4	4222.8
O55147	Utrn	1286.1	1449.8	1570.2	1796.8	1700.9	1617.9
O55158	Tspan8	447.1	522.1	1064.8	881.9	657.5	558.3
O55159	Epcam	555.1	625	540.2	526.4	754	741.3
O55171	Acot2	106.5	167.3	163.2	134.4	256.9	235.5
O55211		7526.5	6794.1	4251.2	4712.4	5136.9	4683
O70190	Nf1x	241.2	332.6	646.8	439.4	674.8	527
O70196	Prep	1333.9	1583.5	1789.5	1848.1	1772	1743.7
O70257	Stx7	1143.8	1062.3	2128.4	1981.9	1236.7	1105.4
O70351	Hsd17b10	2510.8	2438.7	2525.6	2257	1657.1	1664.5
O70377	Snap23	432.6	446.6	432.6	421.5	548	568.5
O70513	Lgals3bp	151.4	215	533.5	410.3	228.1	210.2
O70593	Sgta	1099.1	1149.9	1628.3	1524.6	1207	1187.9
O70595	Abcb6	65	87.9	87.7	102.8	90.5	84.9
O88321	Psmd4	187.1	229.5	154	198	213.9	181.2
O88453	Safb	1964.6	2050.9	2344.5	2332.5	2149.4	2024.9
O88637	Pcyt2	531.9	639.1	801.6	656.2	526.6	485.7
O88656	Arpc1b	1410.8	1540.7	2293.5	1851.2	2372.4	2406.9
O88764	Dapk3	147.8	195.7	206.5	194.6	285.7	290.6
O88767	Park7	10875.8	7514.2	6154.6	6091.1	6367.1	6015.9
O88794	Pnpo	121.6	118.4	67.1	69.4	70.9	83.1
O88801	Homer2	147.6	196	121.8	151.5	160.8	165.4
O88941	Mogs	3544.4	3346	3003.9	3175.5	2740.1	2648.9
O88989	Mdh1	5080.2	5377.9	4645.1	4296.3	5259.4	4500.6
O89035	Slc25a10	1856.6	1934.4	2133	1933.7	1814.4	1823.2
O89046	Coro1b	2523.6	2616.6	1837.5	1911.5	2558.2	2403
P00173	Cyb5a	2545.4	2617.5	956.1	1055.6	2118.8	2316.7

P00388	Por	224	261.3	163.4	154.1	229.3	223.2
P00406	Mtco2	1490.1	678.1	380.2	539.5	422.2	418
P00507	Got2	4033.4	3820.7	3821.1	3857.3	4056.9	3701.2
P00697	Lyz1	185.1	244.1	430.8	489.3	300.4	639.3
P00731	Cpa1	45636.5	38298.4	37367.3	43281.9	26399.4	24933.7
P00762	Prss1	15329.9	16487.7	13473.3	14987.5	11592.1	10725.6
P00763	Prss2	5910.1	7231.9	22769.9	11209	5543.6	5799.3
P00774	Cela2a	14756.9	12187.8	8348	7211.3	7718.9	7519.3
P01015	Agt	121.5	158.3	368.5	352.7	339.5	290.6
P01041	Cstb	1616.7	1604.8	2179	2511.9	1848.3	1894.3
P01322	Ins1	496.7	471.8	103	118.1	586.6	498.6
P01681		78.9	107.8	228	235.6	172.9	163.1
P02401	Rplp2	3844.6	3872.1	3584.4	3620.2	2557.9	3662.4
P02454	Col1a1	4059.9	4264.5	6135.4	5809.8	5838.6	5534.4
P02625	Pvalb	293.8	169.3	143.5	152.5	401.1	385.2
P02651	Apoa4	3739.4	4746.6	7807.4	9243.5	6943.8	7434.3
P02680	Fgg	7301.3	8768.9	13213.5	18366.7	16128.5	16972.9
P02764	Orm1	790.5	799.8	1532.2	1332	2641	2990.9
P02767	Ttr	3545.1	4911.1	3835.5	4652.6	4600.6	6155.1
P02770	Alb	4149.1	904.8	851	978.3	715	641.3
P04041	Gpx1	1354	1172.9	1813.5	2209.5	2411.6	2383.3
P04055	Pla2g1b	3051.3	3048.9	2444.1	2548.6	2292.4	2326
P04157	Ptprc	1758	1897.5	2252.7	2236.1	4636.9	4806.9
P04166	Cyb5b	1623.2	747.3	804.1	880.1	621.1	631.2
P04182	Oat	841.9	1049.4	1059.5	1138.5	1247.9	1370.5
P04218	Cd200	176.1	160.6	413.8	473.5	189.3	167.6
P04256	Hnrnpa1	3870.8	4147.6	4082.9	3578.2	4932.3	5191.2
P04636	Mdh2	18372.9	16202.6	16581.6	16732.1	12608.5	12577.1
P04638	Apoa2	143.8	185.5	709.3	1012.7	251.6	290.6
P04639	Apoa1	6067	9062.6	26539	25311.4	11629.6	13486.9
P04642	Ldha	4555.6	4734.5	5240	5240.6	6394.3	6670.6
P04646	Rpl35a	4999.5	3723.4	1579.3	1848.5	2142.4	2393.7
P04762	Cat	6354.2	6499.4	5873.3	6337	6773.7	6716.4
P04785	P4hb	77662.3	65337	47238.8	48956.1	39933.6	42739.6
P04797	Gapdh	97.6	108.9	124.7	119.1	166.7	163.9
P04916	Rbp4	154.3	159.6	241.5	244.3	176.9	191.9
P04961	Pcna	1025.1	1177	1767.3	1581.7	1611.1	1835.6
P05065	Aldoa	6237.4	6367.7	5985.4	5631.7	9717	9956.8
P05197	Eef2	31764.1	32023.2	35505.5	32645.6	27953.8	27228.2
P05370	G6pdx	2408.8	2300.7	2775.5	2743.1	3206.4	3134.9
P05426	Rpl7	9415.5	8982.8	7446.2	7352.5	8270	7505.7
P05545	Serpina3k	2251.7	2550.9	2114.2	2202.3	3968.4	4400.1
P05708	Hk1	518	581.8	726.7	870.4	696.9	594.1
P05712	Rab2a	7614.7	7415.5	6191.9	5896.7	6092.1	6334.1
P05942	S100a4	135.2	141.7	173.5	194	316.3	284.8
P05964	S100a6	206	144.7	144.4	173.6	175.6	210.8

P05982	Nqo1	302.5	281.1	602.2	509.7	348.1	400.6
P06214	Alad	710.7	679.3	1428.5	1142.3	697.3	670.1
P06238	A2m	155.4	207.5	457.5	402	237.2	225.4
P06302	Ptma	3021.5	3310.1	1944.4	1793.4	4515.3	4125.6
P06685	Atp1a1	14139.8	12880.7	13685.7	13922.7	11528.6	11225.7
P06761	Hspa5	86013.5	71155	51301.8	54314.6	50802.9	53711.4
P06765	Pf4	966	1232.6	923.1	1396.6	1316.4	1528.9
P06766	Polb	190	202.1	328.7	254.7	190.2	251.6
P06866	Hp	6739.1	7297.1	14301.4	15190.6	21105.6	25477.7
P07150	Anxa1	17120.6	13860.6	13736.3	15466.6	23483.7	26022
P07151	B2m	900.2	769	869.1	851.3	1007.4	1090.3
P07154	Ctsl	1504.4	1588.6	1931.8	2078.3	2714.4	2620.3
P07314	Ggt1	8184.1	6752.7	7107.7	7408.3	4863	4694.5
P07323	Eno2	240.7	257.5	380.9	353.7	302.3	291.7
P07335	Ckb	2206.2	2165.6	2602.5	2296.2	4329.4	3610
P07340	Atp1b1	469	627	501.3	434	621.3	660.8
P07687	Ephx1	2633.2	3009.2	3691.3	3475.6	3626.5	3449.3
P07882	Cel	9767	9173.3	1683.5	1288	3387.3	4231.2
P07895	Sod2	4053.6	4753.1	10944.6	10147.2	5966.1	5698.8
P07896	Ehhadh	494.5	546.1	541.1	519.7	627.2	586.8
P07943	Akr1b1	9086.5	9488.7	5899.2	6043	9207.5	8746.1
P08009	Gstm7	1002.8	1321.2	1087.7	1320.2	1129.3	1073
P08010	Gstm2	2650.6	3762.7	3653.8	3329.7	2989.4	3105.3
P08082	Cltb	2464.1	2708.1	2313.4	2238.1	2958.3	3200.4
P08426	Try3	15815.8	13869.4	6798.2	6471.4	6230.4	6170.8
P08461	Dlat	1139.7	1119.8	1232.1	1355.8	1028.7	983.8
P08503	Acadm	4438.9	4707.8	5709.9	5253.1	4194.1	4030.8
P08592	App	89.2	118.7	195	198.6	123.9	126.1
P08753	Gnai3	45.1	55.2	97.5	93.8	84.6	81.5
P08932		1097.9	1343.8	3079.9	2739.5	3904.5	4050
P09034	Ass1	45.9	56.5	111.5	111.3	86.5	85.1
P09456	Prkar1a	2891.1	3311.7	3806.9	3798.9	4390.3	4496.7
P09495	Tpm4	6523.5	6235.6	4974	4640.2	10023.3	11357.4
P09527	Rab7a	6179.5	5960.6	4441.6	4645.8	6348.3	6205
P09606	Glul	374.2	240.1	136.2	356.3	369.6	482.4
P09650	Mcpt1	4260.4	2950	3452.1	3706.5	3068	2747.1
P09655	Spink1	48.9	48.6	181.7	155.1	44.8	48.3
P09656	Spink1l	1421.4	1568.6	563.8	522.3	566.4	791.7
P09895	Rpl5	21624.1	19081.6	26582	28877.9	15670.1	15642.7
P0C0R5	Pik3r4	101.5	114.5	110.6	101.8	138.3	125.4
P0C169		1750.5	464.5	503.3	785.9	295.9	306
P0C5H9	Manf	9228.2	10063.6	10639.7	10125.6	6672.1	7043.5
P0DMW0	Hspa1a	1872.4	2175.5	3085.9	3065.1	3941.5	3552.3
P0DN35	Ndufb1	537.5	439.5	351.1	336.2	342.2	298.3
P0DP31	Calm3	631.2	604.4	599	496.4	775.7	820.2
P10111	Ppia	19109.4	20388.7	15165.3	16602.3	22481.8	23404.4

P10252	Cd48	343.9	358.9	127.7	153.5	402.5	436.2
P10686	Plcg1	249.1	262.9	250.9	241.3	316.5	350.4
P10687	Plcb1	619.6	738.6	1223.6	1196.4	563.8	593.4
P10719	Atp5f1b	23679.1	18290.4	18757.2	21390.7	13923.1	14142.5
P10758	Reg1	11412.1	13073.6	18612.8	19916.3	34276.5	35260.9
P10760	Ahcy	18771.7	16912.7	14564.2	16358.2	14485.4	14499.3
P10818	Cox6a1	438.7	470.2	281.5	243.9	341	421
P10860	Glud1	28512.2	27537.1	20080.4	17798.1	19199.9	19505
P10868	Gamt	1923.7	1610.2	1987.7	1681.5	890	1030.3
P10888	Cox4i1	7943	7805.2	5593.1	5879	5396.1	5714.7
P10959	Ces1c	4332	4454.3	4281.4	5630.3	5466.8	6528.2
P11030	Dbi	7045.4	9406.2	12944.6	10623.6	9447.8	10372.8
P11232	Txn	5209	5292.1	3748.3	3989.9	5242.6	5037.3
P11240	Cox5a	3115.1	1761.1	2697.2	2918.4	1761.2	1800.9
P11275	Camk2a	1997.4	2019.9	754.6	879.9	1554.1	1566.1
P11348	Qdpr	3895.5	4187.8	3483	3316.2	3967.7	3666.2
P11442	Cltc	13929.4	13448.8	15187	14720.1	16080.6	15771.4
P11497	Acaca	1428.9	1254.6	1464.7	1419.6	1105.1	1144.3
P11505	Atp2b1	975.6	1002.2	1703.6	1690.4	1102.4	1282.4
P11507	Atp2a2	9632.5	10532.2	10476.2	9523.4	10679.8	9859
P11654	Nup210	646.8	748.3	884.7	917.1	880.6	871.4
P11762	Lgals1	4012	3786.3	2884.5	2885.4	5433.1	5111
P11884	Aldh2	11778.5	10614	12736.8	12375	8799.2	9021.8
P11915	Scp2	2975.8	2878.8	2970.3	3001.9	3645	3587.6
P11951	Cox6c2	2291	2381.5	865	808.5	1635.2	1669.3
P11980	Pkm	795.6	527.9	343.3	386.1	684.9	737.7
P12001	Rpl18	6228.6	5158.7	3882.9	3732.7	3956.6	3977.2
P12007	Ivd	6708	6991	5677.1	5127.9	4582.3	4813.8
P12075	Cox5b	5666.9	5343.9	4518.3	4512.2	4399.5	4313.9
P12346	Tf	2280.8	2022	1282.2	1370.9	2271.7	2869
P12368	Prkar2a	3836	4110.1	4527.7	4485.8	4570.3	4430.4
P12749	Rpl26	4613.7	4399.4	1677.5	1620.4	3126.5	2932.3
P12785	Fasn	22840.6	17876.9	17798.4	17807.7	16412.7	16554.2
P12788	Try4	1475.1	1980	2636.6	2861	7276	8178.3
P13084	Npm1	2967.1	2603.2	1978.5	2137.3	2653.1	2520.7
P13221	Got1	2742.2	2591.7	2914.9	2980.6	2273.2	2293.2
P13233	Cnp	2825.1	3450.3	3898.2	3403.3	3707.4	3784.1
P13255	Gnmt	2811.2	2417.8	1405.8	1386	1646.9	1564.9
P13599	Fcgrt	628.7	632.4	958.1	945.3	931.5	941.7
P13601	Aldh1a7	55.7	148.4	214.5	179.1	104.3	72.7
P13635	Cp	7017.4	7342	13408.5	19843	10999.3	12599
P13668	Stmn1	1213.1	1294.1	1522.8	1491.4	2128	2312.4
P13803	Etfa	8388.4	9839.3	9563.2	8770.3	7565.7	7457.4
P13832	Rlc-a	152.5	176.8	138.9	372.4	446.2	376
P13852	Prnp	182.6	156.6	156.6	134.8	173.8	196.3
P13941	Col3a1	926.9	1086.3	1211.5	1157	1992.3	2655

P14141	Ca3	9367.6	6487.6	4097.8	4386.8	6129.9	6311.4
P14272	Klkb1	353.2	506.9	1644.8	1709.5	699.5	691.4
P14408	Fh	6283.8	7321.9	7377.1	7244.3	5930.5	5821.1
P14480	Fgb	4385.3	4843.5	15040.5	16439.7	9641	9957.5
P14604	Echs1	4579	5061.5	3511.6	4098.1	3792.4	3622.9
P14668	Anxa5	4550.6	5780.9	10693.7	10390.6	7405	8211.1
P14669	Anxa3	3305.7	2790.1	4707.6	4371.8	4208.7	3913.6
P14942	Gsta4	3910.8	3889.4	5418.9	4647	4928.8	3759.1
P15205	Map1b	316.6	365.3	525.5	528.4	552.8	505.7
P15429	Eno3	582.8	496.9	617.3	595	496.7	467.7
P15650	Acadl	2488	2594.8	2637.7	2853.2	3168.7	2947.8
P15651	Acads	1895.4	1553.2	1330.5	1364.8	1739.3	1720.5
P15684	Anpep	659.7	671.6	1323.9	1006.7	840.9	906.8
P15800	Lamb2	3482	2798.4	3444.5	3301.5	3089.1	2971.6
P16036	Slc25a3	7482.1	5496.6	3251.6	2783	3590.2	3808.9
P16391		1350.7	1382.8	2012.2	1937.5	2122.8	2478.7
P16446	Pitpna	4112.9	4495.8	3416.6	3007.7	4845.8	4912.7
P16617	Pgk1	16944.2	15744.8	13311.5	12934.4	19179.3	18445.5
P16638	Acly	9826.6	10400.8	9147.8	8939.1	10487.8	10070.8
P16970	Abcd3	291.8	369.1	517.5	561.6	527.7	486.9
P16975	Sparc	591	608.7	805	875.1	926	963.8
P17046	Lamp2	1844.5	1642.4	1026.1	981.3	2127.9	1883.3
P17078	Rpl35	3241.6	2104.5	1170.1	1699	2973.1	1986.4
P17220	Psma2	269.5	380.3	303.5	249.3	386.6	400.3
P17764	Acat1	23132	26027.6	17852	16785.6	14026.2	14120.9
P17955	Nup62	580.2	707	794.5	831.8	972.7	944.1
P18163	Acsl1	8657.6	7085.3	6757.5	6809	6663.3	6561.3
P18395	Csde1	4695.8	5142.3	5674.8	5856.5	5198.6	5056.8
P18418	Calr	21652.9	21039.3	20940.8	19127.9	18286.3	19141.9
P18420	Psma1	2990.2	3455.1	9011.6	8182.9	3943.8	4080
P18421	Psmb1	668.9	581.7	381.1	399	642.8	643
P18437	Hmgn2	1427.2	1434.2	1222.3	961.2	1080.7	947.5
P18886	Cpt2	3656.9	3136.3	3764.1	3882.2	2816.7	2750.4
P19112	Fbp1	2280	2202.7	2784.7	2755.2	2166.7	1844.9
P19132	Fth1	206.5	189	398.7	427.6	264.5	242
P19139	Csnk2a1	684.2	875.1	1423.5	1744.9	1220.1	1133.1
P19223	Cpb1	873	636.8	150.8	122.3	295.6	345.3
P19234	Ndufv2	3676	2298.1	3019	3229.5	1837	1705.6
P19468	Gclc	144.6	206.8	261.6	240.2	268.9	231.2
P19511	Atp5pb	1796.9	2518.7	2629	2521.6	2561.1	2289.5
P19643	Maob	794.3	850.1	778.7	685.9	573	615
P19804	Nme2	891	1280.1	3562	3424.2	1276	1133.7
P19814	Ttgn1	1277.7	1427.4	794	1109.7	1306.6	1305
P19836	Pcyt1a	114.6	142.9	198.7	203.4	161.2	141.5
P19944	Rplp1	5360.2	5053.1	1881	2325	3387.1	3503
P19945	Rplp0	14708.7	20500.3	12381.5	13722.5	16104.7	16543.8

P20059	Hpx	10665	10990.4	19383	19421.3	18771.9	19596.4
P20069	Pmpca	1126.4	1288.4	1680.7	1702.7	1251.8	1160.1
P20070	Cyb5r3	1397.7	1547.3	1733.1	1696.2	1775.2	1460.9
P20611	Acp2	293.5	488.4	622.6	466.4	983.2	756.6
P20695	Ifrd1	319.8	401.1	358.2	377.5	386.6	383.4
P20761	Igh-1a	4004.1	4231.1	5116.6	5421.1	6144.2	6626.9
P20762		2172.4	2523.6	6122.5	5878	3036.3	3208.3
P20767		687.5	633.8	1398.5	1414.1	1069.3	1135.1
P20788	Uqcrfs1	3957.2	4032.7	2960.9	2742.7	2744.1	2786.2
P21531	Rpl3	15317.2	15815.5	11183.3	10049.8	10283.9	10336.4
P21571	Atp5pf	554.7	649.7	959.5	915.7	697.5	544.5
P21575	Dnm1	108.1	69	103.5	85.1	152.1	127.5
P21670	Psma4	1191.7	1788.1	2730.2	2898.7	2194.8	2242.4
P21708	Mapk3	686.8	715.1	1011.7	970.4	805.2	745.1
P21913	Sdhb	2735.5	2428.1	1908	1952.4	2196.1	2139.6
P22062	Pcmt1	2272.8	2285.3	1347.5	1303.5	2689.3	2561.1
P22509	Fbl	920	1431.2	1496.2	1392.6	1482.2	1420.6
P22734	Comt	1998	2195	2961.6	3093.4	3349.1	3390.2
P22791	Hmgcs2	781.4	931.5	1484.6	1411.7	2506.2	2448.7
P23347	Slc4a2	151.3	144.4	220.6	254.9	169.4	154.8
P23358	Rpl12	9684.1	10574	6759.3	7041.4	9278	8621
P23457	Akr1c9	515	558.5	573.6	564.4	569.1	513.5
P23514	Copb1	7049.1	7784.8	6485.6	6611.7	7417.5	7360.6
P23606	Tgm1	146.8	151	266	216.9	212.8	201.1
P23640	Rab27a	344.8	456.9	258.9	248.1	497.9	494.8
P23711	Hmox2	732.5	841.2	818.7	835.4	1048.3	1067.4
P24049	Rpl17	10332.9	10764.4	9924.9	8541.5	7684.9	7561.7
P24051	Rps27l	464.1	544.1	404	429.8	575.9	498.7
P24090	Ahsg	7568.3	8372.6	18910.6	12028.1	10493.7	11882.6
P24155	Thop1	3341.3	3426.6	4718.1	4832.5	3841.5	3585.4
P24329	Tst	3708.2	2377.9	2576.6	3020	1413.6	1480.3
P24368	Ppib	34921	32080.9	16347.2	15762.2	25195.2	25286.9
P24473	Gstk1	1260.6	1803.7	1863.8	2072.7	2007.1	1881.5
P24528	Mgmt	118.6	102.1	247	186.7	119.5	116.1
P25030	Krt20	147.7	149.5	307.2	209.1	173.2	168.6
P25086	Il1rn	258.9	250.5	495.3	595.8	838.9	790.4
P25093	Fah	3341.9	3072.7	4268.8	4648	2659.9	2600.1
P25113	Pgam1	6177.9	5965	3199.4	3962.1	6948.5	7094.8
P25235	Rpn2	5191.3	4482.3	3328	3150.8	3333	3395.1
P25286	Atp6v0a1	418.9	417.2	583.9	627	494	467.9
P25304	Agrn	1129.1	1293.8	1723.3	1766.9	1490.5	1489.4
P25409	Gpt	565.2	329.5	398	406	202.4	201.8
P25886	Rpl29	780.1	739.3	778.2	764.6	519.8	640.3
P25977	Ubtf	436.6	501	488.4	692.6	645.3	629.5
P26284	Pdha1	5187.3	3751.1	2952.4	3101.1	2638.4	2721
P26644	Apoh	2577.1	1449.4	2641.1	3049.4	1632.3	1643.8

P26772	Hspe1	8788.1	7945.9	10925.3	11879.2	6588.2	6327.2
P27008	Parp1	5028.3	5328.2	6200.6	5735	6130	6095
P27139	Ca2	755.6	1008	513.2	507.9	1017.8	994.5
P27274	Cd59	435.5	128.7	295.3	296.6	151.7	144.6
P27605	Hprt1	1448.3	1263.5	931.6	926	1170.3	1273.2
P27657	Pnlip	9643.7	8751	4529.3	4726.5	5071.9	4054
P27791	Prkaca	968.3	971.5	1559.9	1832	1048.2	1037.9
P27867	Sord	1683.4	1800.6	1723.8	1454	1290	1243.8
P27881	Hk2	589.4	612.7	974.6	917.5	755.9	794.9
P28037	Aldh1l1	657.5	693.6	471.3	435.6	424.9	357.2
P28042	Ssbp1	2680.4	2563.4	2724.5	2788.4	1880	1924.3
P28073	Psmb6	1736.9	2156.4	2356.6	2356.6	2000.1	2028.4
P28480	Tcp1	7299.3	7281.9	7609.9	7454.1	8110.6	8177.6
P28492	Gls2	933.2	1158.4	2809.8	2828.2	973	1012.3
P28494	Man2a1	913.8	997.9	1055.2	1067	1075.8	1096.8
P28648	Cd63	593.5	571.9	1960.4	1831.8	846.7	855.8
P29117	Ppif	976.7	1009.3	894.3	1149.9	1250.8	1361.4
P29266	Hibadh	1541.7	1687.7	2069.6	1952.8	1159.5	1166.9
P29314	Rps9	8289.8	7366.1	5336.1	6125.9	6456.1	6203.3
P29315	Rnh1	12605.7	10228.7	7848.4	7019.9	7533	7608.5
P29418	Atp5f1e	746.5	763.5	620.7	654.7	413.5	418.7
P29419	Atp5me	2228	1401.6	479.1	562.6	1148.9	1175.6
P30121	Timp2	224.2	227.8	358.4	321.5	308.5	296
P30349	Lta4h	4882.5	5174.5	4787.7	4598.7	5600.4	5753.9
P30835	Pfkl	419.5	372.4	442.2	460.4	425.7	418.1
P31000	Vim	14574.8	9485.3	9290.4	11265.5	15155.1	17504.8
P31044	Pebp1	3574.7	3717.6	2069.6	2527.4	3759	3737.9
P31211	Serpina6	582.7	713.8	2046.3	1628.4	1013.9	1046.9
P31399	Atp5pd	4459.2	4518.2	3409.5	2983.8	3581.9	3522.2
P31430	Dpep1	788.6	516.1	632	742.5	335.7	354.1
P31722	C1qc	158.6	330.9	627.4	679.7	555	649.5
P31977	Ezr	3747.3	2904.1	2352.1	2457.6	3884.5	3720.8
P32089	Slc25a1	835.7	906.5	683.5	628.6	871	861.5
P32198	Cpt1a	355	427	493.5	466	579	584.3
P32232	Cbs	7065.7	5969.8	5085.9	4908.2	3640.3	3625.5
P32551	Uqcrc2	7211.5	7114.2	5466.6	5251.3	5726.7	5605.4
P32577	Csk	366.1	288.2	331.7	291.4	298.2	262.3
P32821		193.8	254.1	237.1	239.2	418.6	408
P34058	Hsp90ab1	13151.9	12908.6	8145	7955.4	14122.2	15052.1
P34064	Psma5	1720.8	1926.6	2284.8	2336.3	2313.3	2392.2
P35213	Ywhab	2489.6	3035.9	2134.2	2194.6	3132.6	3202.1
P35231	Reg3a	521.4	694.3	1275.4	1499.4	3884.8	3763.4
P35433	Ppat	983	1052.9	722.8	698.6	901.3	844.8
P35435	Atp5f1c	4719.6	5340.2	4975.1	4986.1	5002.9	5278.8
P35559	Ide	3386.1	4326.9	4666.1	5170.2	4205.1	4099.2
P35565	Canx	14345.2	14294.6	10626.6	11626.3	12536.5	11946.6

P35571	Gpd2	1040.4	1086.6	1209.4	1181.3	1220.8	1126.8
P35815	Ppm1b	81.3	104.5	84.2	93.2	100.8	100.6
P36201	Crip2	888.5	825	1578	1637.3	1043.3	982.8
P36506	Map2k2	768.9	805.4	612.5	542.9	878.8	837.5
P36860	Ralb	491.3	601.2	244.2	315.3	821	878.4
P36876	Ppp2r2a	234.6	207.9	313.6	291.4	206.9	210.5
P36953	Afm	6273.2	6769.9	11645.4	10432.1	6632.5	7681.9
P36972	Aprt	3774.2	4475.6	3526.9	3520.2	6697	6574
P37285	Klc1	1231	1361.2	1039.6	933.6	1816.5	1968.3
P37397	Cnn3	135.9	245.7	207.1	211.7	488.8	474.9
P37996	Arl3	686.7	733.8	352.9	381.1	839.6	786.4
P38656	Ssb	4821.7	4741.1	7790.7	6575	5464	5391.7
P38983	Rpsa	6767.4	7648.6	4594.9	5959.1	6464.3	6382.5
P39052	Dnm2	931.4	1096.1	1663.3	1602.3	1460.7	1328.3
P39069	Ak1	839.4	368	428.9	487.1	278	278.9
P40112	Psmb3	377.7	453	532.9	700	578.1	543.9
P40307	Psmb2	2261.6	1717.5	1454.1	1742.3	1460.2	1497.8
P40329	Rars1	9860.9	10854.2	10620.2	10515.6	9266.6	9893.7
P40615	Dkc1	722.3	690.9	639.1	756.8	668.8	652.1
P41123	Rpl13	9950.3	5923.6	4789	5074.8	3293.8	3845.2
P41499	Ptpn11	1010.9	952.1	1067.1	1028.8	933.9	913.4
P41542	Uso1	7703.8	7293.7	8251.2	7419.3	6658.4	6266.3
P41562	Idh1	5214	5033.9	4499.7	4221.6	5766.3	5299.9
P41777	Nolc1	1434.8	1628.4	1827.4	1741.1	1619	1481.5
P42123	Ldhb	4008.3	3303.6	5399.3	5006.2	3865	3877.5
P42676	Nln	1269.9	1408.2	1656.9	1609.1	1175.3	1128.9
P42854	Reg3g	120.7	122.6	95	100.4	93	106.7
P42930	Hspb1	743.1	695.5	669.3	698.8	1347	1229.6
P43138	Apex1	494.5	519.9	412.5	401.8	676.6	559.2
P43278	H1-0	2693	3158.4	1381.1	1545.3	3275.8	3176.7
P45479	Ppt1	329.9	353.2	445	466.4	436	440.1
P45592	Cfl1	4286.9	5074.8	4996	4883.6	9114.6	9126
P46413	Gss	852	888.6	762.4	1318.5	914.1	791.5
P46462	Vcp	17048.3	15307.8	12567.1	13056.3	13519.5	13120
P47853	Bgn	2569.1	2089.3	2666.8	2680.2	2457.1	2710.5
P47875	Csrp1	10061.1	7803.8	4353.5	4369.7	9705.9	10708.1
P47942	Dpysl2	5357.7	5657.6	7743.8	8178.7	7470.8	7897.5
P47967	Lgals5	506.7	285	409.5	502.8	353.1	354.2
P48004	Psma7	3186.5	4160.9	4519.4	3958.9	5375	5624.6
P48450	Lss	84.6	114.3	63.6	79.5	86.7	101
P48500	Tpi1	7760.4	7084.3	5745.1	6507	7739.9	7599.8
P48721	Hspa9	9890.4	9862.2	9322	9989.4	8445.2	8276.3
P49088	Asns	7291.7	7738	6101	4945.8	4995.5	5211.4
P49185	Mapk8	101	134.2	193.2	175.3	203.2	342.5
P49186	Mapk9	661.9	740.2	1413.2	1345.7	801	773.9
P49242	Rps3a	25198.3	24084.8	30078.1	33303.4	15376.1	17261.4

P49301	Clec10a	237.4	332.3	607.7	651.1	559	585.9
P49432	Pdhb	2503	2457.7	2587.4	2583.4	1813.9	1866.6
P49793	Nup98	205.2	263.4	357.1	369.4	332.9	302.8
P50115	S100a8	336.9	331.3	778	1030.6	955.1	1743.9
P50339	Cma1	2923.5	3388.9	3307.9	3245.6	4001	3354.7
P50398	Gdi1	1029.9	1197.5	1107.6	1047.7	1233.7	1163.5
P50399	Gdi2	7073.2	7897.1	6093	6448.7	8901.2	8291.2
P50475	Aars1	9062.4	8447.6	7447.8	7432.2	6880.4	6912.8
P50503	St13	5878.9	6114.4	8927.1	8203.5	6129.6	6123
P51158	Rab28	119.6	155.9	149.5	189.5	161.6	148.5
P51583	Paics	1274.4	1259.4	1434.9	1605.9	1469.7	1377.5
P51635	Akr1a1	3628.6	3950.7	2176.5	2141.9	3572.9	3729.8
P51646	Arl5a	48.8	69.9	64.8	80.9	80.5	76.3
P51647	Aldh1a1	6478.8	7407.6	4285.7	3811.4	3959	3579.5
P51650	Aldh5a1	120.6	120.1	114.7	109.1	127.4	156.5
P51886	Lum	5490.1	6434.8	6812.6	7677.7	7489.4	7816.9
P52555	Erp29	2933.5	2309.6	2206.5	2390.4	1949.2	2002.2
P52590	Nup107	127.3	144.1	210.1	203.6	175.9	192.7
P52631	Stat3	1146	1305.6	1185	1329.6	2066.2	2034.1
P52759	Rida	662.3	1074.6	366.9	389.5	731.2	776.5
P52925	Hmgb2	2228.7	1967.8	2126.8	2088.2	3104.7	2834.1
P52944	Pdlim1	1326.5	1555.6	2174.4	2266.3	2121.2	2071.3
P53610	Pggt1b	141.2	179.9	278.1	249.9	163.5	137.1
P53676	Ap3m1	1541.6	1942.8	2123.4	2055.8	2386.6	2240.1
P53812	Pitpnb	1310.5	1748.2	1913.1	1763.4	1860.9	1879.2
P53987	Slc16a1	239.7	240	228.9	181.7	208.6	201
P54001	P4ha1	1356.6	1531.1	2647.1	2401.9	2482.3	2667.6
P54275	Msh2	332.1	499.6	824.9	823.6	643.7	604.7
P54311	Gnb1	522.6	612.5	870.5	1014.7	862.1	879.4
P54313	Gnb2	653	748.9	940	922.4	860.6	870.5
P54316	Pnliprp1	28755.5	33273.8	18654.9	18113.8	20194.4	15421.6
P54318	Pnliprp2	9390.4	9804.9	6853.6	6911.1	6776.1	6325.7
P54319	Plaa	892.6	1005.8	1129.1	1079.3	1065.2	1068.7
P54645	Prkaa1	282.2	319.8	491.7	434	398.7	414.7
P54921	Napa	2759.5	2597.9	1697.9	1975.7	2322.7	2201.7
P55053	Fabp5	3517.9	3088.1	2384.1	2797.1	3851.5	4783.7
P55062	Tmbim6	78.1	79.7	38.7	45.1	48.7	50.1
P55213	Casp3	181.6	189.7	280.4	629.8	284.7	223.8
P55770	Snu13	2510	2420.7	2307.2	2557	2943	3240.4
P56571		2559.8	1624.9	1213.6	1157.2	1467	1311
P56574	Idh2	11807.5	10535.8	7664.8	7950.3	6698.1	6908.5
P57113	Gstz1	1680.1	2088.9	2056.1	2594.4	2032.1	1937.5
P58200	Vti1b	415.5	350.8	681.2	645.9	352.8	346.1
P59798	Selenok	48.5	59.8	57.2	58.4	56.1	53.1
P59924	Thoc1	198	249.1	227.5	289.1	331	319.3
P60123	Ruvbl1	2779.1	2797.3	4386.2	4882.8	3134.2	2982.9

P60522	Gabarapl2	329.6	347.1	1009.2	936.8	476.4	478.3
P60901	Psma6	5381.6	4895.2	4042.4	4395.9	4715.9	4675.9
P61107	Rab14	3520.1	3959.8	4884.5	4394.1	4123.7	3682.3
P61203	Cops2	739.9	689.1	548.4	464	566.5	629.3
P61206	Arf3	1064.8	1107	1216.3	1248.3	1247.3	1070.2
P61212	Arl1	424.2	583.8	386.4	374.5	594.2	527.8
P61227	Rap2b	389	490.2	590.3	579.9	612.7	633.3
P61314	Rpl15	9689.4	10231.4	3201.5	3416	6012.4	7153.7
P61354	Rpl27	2994.2	3650	2539.1	2455.2	2799.9	2748.4
P61480	Wdr12	330	337	535.1	521.7	380.7	378.9
P61515	Rpl37a-ps1	2757.6	2719.8	928.1	851.9	1466.5	1434.2
P61589	Rhoa	4169.3	4963.3	5179.1	4867.7	5779.9	5703
P61621	Sec61a1	2449.2	2960.5	1166.6	1092	2549	2086.1
P61751	Arf4	2642.5	2644.1	1661.5	1917.2	2246.6	2161
P61765	Stxbp1	409.7	452.6	613	612.7	608.8	507.3
P61959	Sumo2	892.6	784.1	323.6	316.5	728.9	835.9
P61972	Nutf2	1300.7	1441.9	1168	1074.9	1362	1481.8
P61983	Ywhag	2803.2	2673.9	2102.1	2124.6	3104.8	3197
P62074	Timm10	381.7	447.4	493.7	446.1	299.6	352.7
P62076	Timm13	1118.3	1226.9	1435.4	1288.4	993.8	932.3
P62078	Timm8b	173.4	198.4	291.8	335.4	149	135.9
P62138	Ppp1ca	897.9	975.2	1621.9	1578.7	1309.8	1291.4
P62142	Ppp1cb	144.7	164.9	159.1	130.6	116.5	137
P62193	Psmc1	2895	3413.6	3656.7	3113.6	4067.5	4097.1
P62198	Psmc5	1112.7	965.7	742.7	756.4	983.1	962
P62243	Rps8	9429.5	8144.1	10544	10579.8	6437.7	6330.3
P62255	Ube2g1	467.8	635	752.1	622.8	873.7	725.9
P62260	Ywhae	16995.7	17704.2	14319.5	13900.4	16304.6	17858.6
P62278	Rps13	12273.7	15054.6	6137	7206.4	11802.4	13040.1
P62282	Rps11	19662.5	20902.8	9753.9	10037.1	14539.7	15067.5
P62329	Tmsb4x	6077.1	6435.7	5299.5	5582	9287.8	11740.3
P62332	Arf6	725.3	1055.7	659.4	815.7	1303	1265
P62494	Rab11a	3477.8	3818	3292.6	3206.9	4602.7	4791.9
P62501	Tsc22d1	274.3	287.6	315.9	369.6	380.9	382.5
P62634	Cnbp	198.6	177.5	553.9	560	165.7	154.5
P62718	Rpl18a	7066.8	7778.1	3782.6	4294.3	4724.2	4614.3
P62749	Hpcal1	1489.3	1683.5	1607.7	1573.1	1615.5	1712.4
P62755	Rps6	9089.5	9781.2	11769.7	10863.3	8450.5	8200.7
P62775	Mtpn	475.2	526.5	471.3	472.2	827.5	730.2
P62804	H4c2	20920.1	10285.1	7928.5	9448.4	8628.5	9277
P62815	Atp6v1b2	220.7	275.2	166.9	173	322.3	286.6
P62828	Ran	5202.3	5661.5	3850.6	3728.9	6883.8	7430.5
P62832	Rpl23	7250.9	8304.3	8097.4	7233.6	7412.8	6956.8
P62836	Rap1a	1150.3	1270.8	478.8	543.7	1209.7	1360.8
P62845	Rps15	8027.2	8175.9	5389.2	5005.8	5640.5	5692.4
P62856	Rps26	3005.3	2783.2	911.4	995.4	1845.9	1757.3

P62859	Rps28	3881.8	4058.4	2102.8	2162	3666.6	3416.8
P62870	Elob	2474.9	2708.5	2022	2253.4	3319.3	3144.7
P62890	Rpl30	2227.4	2600.3	1212.7	1499.6	1744.5	1776.5
P62893	Rpl39	2143.6	643.8	1434.9	1116.3	501.3	501.5
P62898	Cycs	5504.8	4886.5	2822.7	3951	3135.8	3644.9
P62902	Rpl31	5224.6	4187.6	3083.4	2874.5	2742.6	2903
P62907	Rpl10a	12822.4	13891	8567.1	10352.2	10266.6	10754
P62909	Rps3	15400.1	16294.1	15544.8	16943.9	12661.1	12184.9
P62912	Rpl32	5942.6	6717.1	5381.2	4715.6	5372.9	5495.4
P62916	Gtf2b	133.3	224	292.1	303.4	290.2	300.5
P62919	Rpl8	3774.5	3793	3188.2	3428.9	2629.8	2610.6
P62959	Hint1	1673.9	1844.8	923.3	672.5	1206.5	1344.8
P62963	Pfn1	4460	4231.9	4662.9	4801.3	6882.4	6943.6
P62982	Rps27a	11575.4	12814.7	11428.4	11429.1	8702	9641.5
P62994	Grb2	439.7	356.9	393.2	425	485.2	528.6
P63004	Pafah1b1	3762.9	4091.1	4543.4	4344.8	4030.7	4044.4
P63018	Hspa8	25802	24888.2	18414.3	20711.5	32126.8	31929
P63029	Tpt1	8858.5	16088.7	11273.2	12583.3	15276.2	16211.9
P63036	Dnaja1	981.1	1035.6	1171.2	1046.7	1280.4	1289.4
P63039	Hspd1	24019.2	21462.8	17673.7	17177.3	16021.6	16162
P63074	Eif4e	624.6	579.1	480	458.8	556.8	484.7
P63086	Mapk1	954.6	1191.7	1720.6	1728	1739.7	1700.5
P63088	Ppp1cc	560.1	602.4	661.3	706.7	796.7	788.6
P63102	Ywhaz	6736.4	6946.6	3372	2878.1	8087.5	8476
P63155	Crnkl1	505.7	516.1	621	763	579.8	571.6
P63170	Dynll1	652.1	668.2	323.4	331.8	951.4	1074.8
P63245	Rack1	27267.4	27849.2	16539.1	17925.9	25126.1	25695.2
P63255	Crip1	320.6	297	122.5	122.7	376.7	426.6
P63259	Actg1	2802	3486.5	2905.7	2999.5	5850.6	6051.5
P63269	Actg2	8729	9651	6787.9	8252.7	16841.7	17683.6
P63312	Tmsb10	850.9	1114.8	1588	1629.4	1679.4	2642
P63322	Rala	1570.8	1808.3	323	505.8	2071.7	2129.4
P63326	Rps10	9196	7135.6	7200.6	6602.4	4850.8	4708.9
P63329	Ppp3ca	4331.7	4020.9	3363.8	3733.5	2845.8	2960.4
P63331	Ppp2ca	1078.3	1279	1725.8	1625	1583.7	1555.1
P67779	Phb	4928	6047.6	5201	4920.9	5220.2	5073.9
P67999	Rps6kb1	82.7	111.5	496.5	251.7	188.7	159.3
P68101	Eif2s1	2185.8	2314.9	2221.6	2078.5	2009.9	1938.6
P68255	Ywhaq	5358.4	5284.3	4757.9	4505	6660.5	6509.7
P68511	Ywhah	5524.8	5263	5853.6	5013.6	6296.9	6539.8
P69682	Necap1	750	863.9	1989.7	1950.6	790.2	815.3
P69736	Edf1	2944.3	2951.2	2236.5	2091.4	2571.4	2549.6
P69897	Tubb5	4222.1	4477.3	2033.4	2366.1	5826.2	5740.2
P70500	Cdipt	455.5	468.9	289.5	292	362.1	344.6
P70541	Eif2b3	1466.2	1342.9	2240.8	2050.4	1416.1	1249.7
P70562	Bhlha15	324.3	355.6	754.2	682.5	401.4	363.9

P70565		1114.7	1073.2	1458.9	1336.1	1127.9	1097.9
P70569	Myo5b	96.4	103.2	173	174.7	135.2	117.4
P70580	Pgrmc1	677.1	543.1	397.1	281.5	421.1	488.2
P70582	Nup54	356.3	449.5	897	1095.8	700.5	662.5
P70584	Acadsb	5037.1	5350.5	3687.9	3661.9	3700	3644.2
P80067	Ctsc	3041.4	2760.2	1439.5	1787	2390.1	2299.9
P80385	Prkag1	978.3	1074.9	1393.7	1425.8	1197.3	1145.4
P80432	Cox7c	71.8	88.9	50.6	53.3	82.3	59.8
P81155	Vdac2	6503.3	6760.8	4463.8	4025.7	5573	5984.4
P81718	Ptpn6	2123	1911.8	2459.9	2711.1	2229.4	2220.8
P81795	Eif2s3	754.4	777.1	2182.7	1730.4	708.5	692.9
P82471	Gnaq	636.8	686.9	510.2	577.9	659.7	710.1
P82808	Gfpt1	488.8	837.9	1045.6	1185.3	1042.1	1078.3
P82995	Hsp90aa1	10522.7	10181.4	8567.8	8765.6	10598.2	12016.2
P83732	Rpl24	8054.4	6246.6	4344.1	4166.5	4956.8	5152.6
P83941	Eloc	634.2	512.3	655.3	775.7	486.9	476.9
P84083	Arf5	1034.4	1004.8	993.8	954.8	1033.7	948.3
P84087	Cplx2	23.2	19.5	19.9	11.1	30.9	26.6
P84092	Ap2m1	577.8	657.7	567	730.2	871.6	787.5
P84100	Rpl19	1258.5	1647.2	619.8	671.4	1487.2	1249.9
P85108	Tubb2a	848.8	908.3	670.4	764.2	1201.4	1209.7
P85125	Cavin1	5606	5488.6	5164	5162.7	6189.9	6211.3
P85515	Actr1a	397.6	439.8	236.2	236.4	360	436.6
P85834	Tufm	6615.2	6163.6	4838.9	4376.8	5111.6	5069.6
P85845	Fscn1	814.2	1384.1	2068.2	2299.9	1562.9	1324.6
P85968	Pgd	2160.5	2369.3	3026.1	3289.8	3170	3125.9
P85973	Pnp	4414.6	4244.9	4836.2	4615.8	5451.5	5483.5
P86182	Ccdc22	93.2	141.4	186.3	178.3	154.2	153
P86252	Pura	3482.1	2319	1368.8	1468.7	1513.7	1625.8
P97519	Hmgcl	1801.1	1418.2	1948.3	2045.1	1274.8	1248.2
P97521	Slc25a20	1336.9	2151.1	2569	2483	2568.1	2692.1
P97532	Mpst	3699.6	4066.2	4205.4	4722.7	3287.8	2941.2
P97536	Cand1	5628.8	6763.4	7240.4	7821.1	8843.5	8242.7
P97560	tap1	172.5	256.8	239.4	170	278.4	278.3
P97564	Gpam	110.4	125.7	145.1	155.7	146.2	130.9
P97569		701.8	859.6	966.4	1125.2	975.6	1024
P97576	Grpel1	1019.7	982.9	1005.1	1301	1067.9	982.1
P97584	Ptgr1	133.7	125.4	172.1	161.2	176.8	156.5
P97608	Oplah	1005.8	1035.6	1086.8	1246.7	893.8	835.7
P97612	Faah	224.7	241.3	299.6	297.6	270.2	274.5
P97615	Txn2	428.3	436.7	192	211.1	526.4	470.9
P97621		3575.8	6530.2	13348.8	12382.4	4796.4	5209.6
P97633	Csnk1a1	499.8	515.7	535.1	483.7	624.2	563.6
P97675	Enpp3	4928.9	5754.1	8231.8	8169.8	7445.7	7247.5
P97690	Smc3	3413.6	3668.4	4483.9	4518	4166.5	4271
P97697	Impa1	1069.6	1024.5	1183.8	1191.2	1053.3	1066.5

P97829	Cd47	901.6	805.2	526	552.9	1006.2	1111.2
P97839	Dlgap4	769.7	853.9	825.6	760.8	766	729
P97852	Hsd17b4	2455.4	2454.2	2914.4	3131.9	3597.3	3545.2
P97874	Gak	532.9	644.8	824.7	743.9	815.9	693
P97878	Exoc5	736.4	747.7	1556.9	1308.7	966.2	917.5
P97886	UGT1	122.8	104.8	240.6	193.6	113.8	107
Q00238	Icam1	270.2	308	476.8	426.6	502.9	523.4
Q00438	Ptbp1	2555.2	2210.5	2464.2	3025.2	2842.4	3017.5
Q00981	Uchl1	814.9	712.1	560.3	493	823.6	957.6
Q01129	Dcn	5569.8	3979.2	5273.4	6155.5	4047.1	4438.1
Q01177	Plg	5368.3	5891.4	9461.6	9140.5	7862	8016.5
Q01205	Dlst	2095.9	1192.7	1183.1	1152.7	1057.7	999.7
Q01579	Gstt1	405.2	434.7	775.9	738.5	353.5	331.8
Q02293	Fntb	222.3	267.1	344	352.7	278.8	251.8
Q02353	Ndst1	520.7	540.8	426.6	714.5	825.1	862.5
Q02356	Ampd2	679.7	697.9	815.5	586.1	573.1	570.9
Q02589	Adprh	709.4	756.7	1555.1	1757.6	1009.8	936.7
Q02874	Macroh2a1	2902.6	3132.5	3654.4	3859.9	4256.2	4034.6
Q02974	Khk	224.1	272.7	146.5	147.5	243.9	231.1
Q03344	Atp5if1	118.4	127.5	96.6	123.1	185.7	154.6
Q03346	Pmpcb	468.7	447.8	659.9	645.5	436.2	409.8
Q03555	Gphn	259.6	269	261.2	326.6	309	333
Q03626	Mug1	1235.4	1280	1843.9	1650.8	1360	1477.1
Q04462	Vars1	7045.1	7500	6993.7	6964.4	7976.8	7474.3
Q04931	Ssrp1	1077.1	925.1	918.1	970.7	1044.3	1114.2
Q04970	Nras	709.6	755.1	584.4	614.9	768.7	742.8
Q05096	Myo1b	1718	1866.1	1831.6	1825.2	1911	1865.3
Q05982	Nme1	385	452.7	962	1399.2	732.2	534.5
Q06000	Lpl	431.5	478.5	419.3	398.4	380.4	384.2
Q06647	Atp5po	2552.7	2733.5	2463.1	2481	2685.7	2241.5
Q06C60	Bola1	209.5	253.2	344	360.2	287.7	309.4
Q07009	Capn2	3455.8	3162.4	9364.8	12822.5	4717.2	4854.6
Q07014	Lyn	404.8	429	790.4	745.9	447.3	448
Q07116	Suox	444.4	555.6	746.2	703.8	642.8	591
Q07205	Eif5	3727.1	4274.1	3199.9	3405.5	3576.8	3476.9
Q07803	Gfm1	179.6	231.5	284.5	444.9	262.6	242.1
Q07936	Anxa2	11675.9	8693.2	8170.9	8574.7	14611	15837.6
Q07984	Ssr4	4167.6	3811.1	2381.1	2647.4	2943.4	2945.3
Q08013	Ssr3	884.4	242.8	26.6	28.4	73.6	75.1
Q08163	Cap1	4045.6	4494.7	5484.9	5429.5	7615.4	7445.1
Q08290	Cnn1	1045.9	1150.6	1631.5	1461.4	1194.5	1251.5
Q08602	Rabggta	404.1	446.6	244.9	240.2	366.6	370.5
Q08849	Stx3	152.5	151.4	406.7	384.5	266.3	296.4
Q08851	Stx5	603.6	681.1	911	825.2	598.9	602
Q09030	Tff2	533.1	357.5	467.4	591	113.2	98
Q09073	Slc25a5	6508.8	5667	4390.5	5601.4	4892	4215.1

Q09167	Srsf5	1604.6	1608.3	857	858.6	1866.8	2031.5
Q09325	Mgat1	158.9	234.9	518.2	375.3	176.9	169.8
Q09326	Mgat2	1426.7	1484.5	1733.4	3607.9	1218.4	1179.8
Q0D2L2	Mrps22	220.6	237.7	240.3	252.5	224.7	217.8
Q0ZFS6	Ifrd2	183.8	206.6	187.5	211.3	228.2	207.7
Q10728	Ppp1r12a	1217.7	1185.1	1125.9	1161.6	1214.5	1260.1
Q10743	Adam10	957.5	1021.5	1321.1	1254.8	1119.2	1010.1
Q10758	Krt8	29805.8	28180.7	32326.7	31535.3	36401.3	38159.6
Q14TE9	Top2b	2804.1	2678	2855.6	2932.7	2908.7	2855.7
Q156J1		33.5	33.1	60.7	62.8	43	34.5
Q19LA7		224.9	243.1	166.4	177.9	303.5	264.4
Q1EG89	Pxn	426.4	463.3	549.6	563.2	644.8	719.7
Q1JU68	Eif3a	13630.5	14096.2	13975.3	13258.9	11614.1	11117.7
Q1KQ07	Stat6	76	128.1	106	113.1	189.7	154.5
Q1PBJ1	Mfge8	212.7	209	384.5	427.2	308.2	304.3
Q1PS21	Mcm7	115.2	137.3	197.5	206.2	268	236.3
Q1RP77	Nop16	244.7	269.4	264.8	239.4	186.6	240.7
Q27W01	Rbm8a	408.1	455.4	365.8	454.9	600.9	521.5
Q2A121	Fto	243.1	267.6	374	342.5	313.5	266.4
Q2KJ09	Usp16	464.2	668.5	1000.7	1180.9	763.6	759.9
Q2LAP6	Tes	1037.5	440	465.1	290.4	369.3	360.9
Q2PQA9	Kif5b	5162.6	4591.8	4475	4120.6	4707.8	4581.4
Q2TA68	Opa1	2705.1	3057.9	2514.1	2332.5	2721.3	2598.4
Q2TL32	Ubr4	707.8	926.8	1260	1477.3	1199.6	1204
Q2VC85	Gcln	93.9	123.4	60.4	62.4	124.7	112
Q32KJ5	Gns	174.7	217.9	460.3	446.9	376.6	366.2
Q32PX2	Aimp2	694.1	733.9	764.7	879.5	674.6	610.8
Q32PX6	Rhog	870.7	1094.2	1800.7	1886.8	1662.6	1682.6
Q32PX7	Fubp1	3064.5	3378.4	4792.9	4254.6	3964.6	3962.6
Q32PY7	Nudcd3	113.2	134.3	153.6	144.9	162.2	146.2
Q32PZ3	Unc45a	215.2	224.5	225.2	202.4	299.1	306.2
Q32PZ7	Srp72	2101	2158.4	951.7	839.9	1589.3	1565.9
Q32Q06	Ap1m1	493.7	532	789.2	704	571.3	563.3
Q38PG1		440.3	369.4	419.8	410.5	488.2	498.4
Q3B7D0	Cpox	677.4	718	1473.4	1268.2	787.1	779.1
Q3B7D1	Ube2z	315.5	429.9	670.9	549.5	391.1	364.9
Q3B7D6	Spon1	204	222.8	793.3	935.4	408.8	429.9
Q3B7U4	Ctu2	30.9	36.8	51	67.6	33.2	33.4
Q3B7U9	Fkbp8	435.5	483.7	402.8	546.9	662.9	579.9
Q3B8N9	Bphl	1175.4	1247.5	1083.3	1145.1	818.6	727.6
Q3B8Q1	Ddx21	2496	2749	3778.5	3568.1	3326.4	3321.5
Q3B8Q2	Eif4a3	713	858.2	514.5	524.4	1016.6	1023.9
Q3B8R4	Igh-6	1700.1	1911.3	2631.1	2454.8	3536.8	3405.1
Q3B8R6	Azgp1	473.2	544.3	757.2	767	711.9	625.9
Q3B8R8	Nudt16l1	74.4	87.6	76.2	97.6	108.1	97.1
Q3KR55	U2af1	916.1	474.8	221.3	193.8	292.5	346.1

Q3KR79	Atf1	265.6	230.8	233.2	289.9	281.2	292.3
Q3KR94	Vtn	389.6	527.9	958.1	651.1	617.1	691.2
Q3KR97	Baiap2l1	504.1	597.6	635.2	656.6	786.1	734
Q3KRC3	Srpra	9288.2	9162.7	7632.3	6889.7	5611.7	5780.3
Q3KRC5	Dus3l	144.9	162.2	53.8	66.8	178.8	153.8
Q3KRD5	Tomm34	355.5	361.6	321.1	354.5	435.6	409.1
Q3KRE0	Atad3	877.9	749	694	634.8	671.8	598
Q3KRE2	Mettl7a	914.2	915.3	574.7	561.6	1337.5	1211
Q3KRF2	Hdlbp	48884	50420.4	46495.1	40993.1	31641.8	31518.8
Q3MHS7	Gmds	5003.8	4429.1	3770.5	4062.9	3225.6	3385.9
Q3MHS9	Cct6a	5135.4	4700.5	4451	4948.8	4731	4890.2
Q3MID3	Arfgap2	2308.8	2150.1	2195.3	2404.9	1869.4	1928
Q3MIE0	Echdc3	331.5	300.5	283.4	277.9	266.5	250.6
Q3MIE4	Vat1	6089.1	5369.1	5392.6	5605.6	6426.1	6422.1
Q3S4A4	Arfgap1	255.9	242.4	376.8	396.4	267.4	243.5
Q3SWS8	Rae1	36.7	33.8	24.7	23.1	44.2	55.2
Q3SWT0	Pecam1	120.8	143.3	138.7	171.6	185.4	186.1
Q3SWT4	Iws1	82	106.9	217.4	301.4	224.5	191.2
Q3T1G7	Cog7	626.4	641.3	367.4	510.5	484.5	513.7
Q3T1I4	Prrc1	1060.1	1423.4	1850.8	1462	1222.8	1177.2
Q3T1J1	Eif5a	9353.6	10883.8	7226.9	6234.4	10432.5	9096.8
Q3T1K5	Capza2	1735.6	1795.1	2294.2	2321.6	2037	1866.6
Q3T1L0	Aldh16a1	981.2	1104.3	1352.5	1223.2	1334.7	1256
Q3ZAV2	Ybx1	1593.6	1434.8	1072.1	1010.3	1639.7	1545.5
Q3ZB97	Ap2b1	2130.7	2187.8	1491.9	1548.5	2248.7	2316.6
Q3ZB99	Tjp2	767.5	601.7	773.6	716.5	726	711.6
Q3ZU82	Golga5	1138.6	1066.8	1222.7	1132.6	981.6	1000.8
Q45G71	Nat1	638.3	635.2	735.4	826	652.6	617.2
Q45QJ4	Plcb3	136.1	160.5	175.8	169.2	200.8	177
Q45QN0	Gnai2	845.6	1046.7	1486.9	1579.3	1429.8	1309.9
Q496Z0	Elp2	404.3	423.5	666.8	622.2	462.3	462.9
Q496Z5	Prph	2552	2254.5	2418.2	2534.9	2669.4	2941.7
Q496Z9	Trmt1l	165.1	164	273.3	430.8	257.7	249.1
Q497B0	Nit2	2397.1	2296.7	2585.1	2435.3	2513.5	2296
Q497C3	Mettl26	277.4	311	678.2	682.3	426.5	375.6
Q498C8	Rer1	1152.1	1199	1432.4	1260.8	977.3	984.7
Q498C9	Zfp207	46.1	31.2	41.9	55.5	41.5	45.7
Q498M4	Wdr5	550.9	516.5	690.6	807.5	531.5	528.9
Q498N3	Dctn4	321.9	412.1	199.8	205	466.4	480.2
Q498R7	Czib	969.8	976.2	719.4	771.5	726.8	798.7
Q498U4	Sarnp	3315	3641.4	3769.8	3848.8	3716.4	3622.8
Q499N5	Acsf2	4538.8	5993.1	5858.9	5905.4	5117.3	5142.4
Q499T2	Ifi30	1125.1	1283.5	804.5	1138.3	3751.7	3251.2
Q499T3	Sirpa	295.8	277.5	551.6	501.4	580.3	647.2
Q499V7	Suclg2	10946	8762.7	8484.7	9656.6	6467.9	6183.6
Q4FZS2	Bub3	1095	1322.7	1289.1	1288.8	1486.4	1417.6

Q4FZT0	Stoml2	237.7	287.6	320.2	316.9	325.3	316.8
Q4FZT2	Ppme1	555.8	400.1	392.8	359	258.8	266
Q4FZT9	Psmd2	2409.6	2342.7	3018.4	2927	2785.4	2527.5
Q4FZU0	Acp6	896.8	997.9	379.7	361.6	865.9	935.7
Q4FZU3	Nsrp1	156.4	167.3	182.7	157.3	193.8	184.1
Q4FZV0	Manba	154.1	123.3	164.1	125.8	123.8	102.7
Q4FZY0	Efhd2	1785.8	1596.4	1493.3	1338.9	1719.3	1805.1
Q4G005	Ercc3	352.9	1110	418.2	447.1	1151.9	1050.3
Q4G009	Mcts1	1397.1	1735.3	2486.2	2118.2	1776.6	1724.2
Q4G022	Get3	1201.8	1173.3	1192.5	1138.1	1592.9	1411.1
Q4G037	Cdc23	706.7	857.9	1047.4	1013.3	946.4	969.9
Q4G061	Eif3b	10871.6	10256.6	9841.1	8598.4	8978.4	8578.4
Q4G063	Creld2	4104.2	2904.7	4488.3	4990.3	2163.6	2166.3
Q4G064	Coq5	179.3	167.9	186.1	175.5	158.3	175.5
Q4G067	Mrpl44	116.5	159.1	196.6	189.9	120.8	133.8
Q4G069	Rmdn1	62	78.7	44.4	56.1	96.3	91.5
Q4G075	Serpinb1a	9059.4	7889.6	4094.8	3801.5	3016.3	3633.7
Q4G079	Aimp1	3197	3317.9	3613.5	3159.8	2945.1	2860.8
Q4KLF8	Arpc5	2039.5	2331.8	1528.3	1692.8	2753.5	3323.4
Q4KLG9	Zfand2b	249.1	231.3	296	248.7	235.6	266.6
Q4KLH4	Pspc1	50.9	69.9	92.5	89.3	108.6	112.1
Q4KLH7	Rad21	465.9	622.2	983.3	866.4	812.6	785.6
Q4KLI0	Smarcb1	253.9	341.3	483.9	465.4	488.1	501
Q4KLI4	Ppil1	2077.1	2003.8	2681.2	2416.4	2444.1	2433.8
Q4KLI7	Sf3a3	2406.3	2571.9	2519.2	2292.4	3438.3	3586.8
Q4KLJ1	Sfrs7	1654.5	1773.8	1311.8	1296.8	2023.5	2024.4
Q4KLK7	Nop56	3424.7	3347.9	3063.4	3485	3584.7	3678.5
Q4KLK9	Ssu72	58.4	95.4	96.4	74.9	78.3	70.1
Q4KLL0	Tcea1	492.9	555.1	657.1	758.2	557.5	540.5
Q4KLL4	Tm9sf4	561.7	692.5	1231.4	1156	831	743.7
Q4KLL7	Vps4b	2512.5	2856.5	2455.3	2348.2	2715	2965.7
Q4KLN7	Arfgap3	2828.6	3467.6	2555.3	2434.5	2626.2	2363.7
Q4KLP0	Dhtkd1	1672.5	1623.5	668.4	647.7	1032.6	969.2
Q4KLZ0	Vnn1	184.9	243.1	314.5	460.6	312.1	278.7
Q4KLZ3	Dazap1	591.8	585.2	525.3	516.5	569.3	546.4
Q4KLZ6	Tkfc	927.6	999.5	1350.7	1266.6	1128.1	969
Q4KM35	Psmb10	327.6	351.1	362.6	395	739.8	796.1
Q4KM38	Srsf10	178.6	214.6	214.5	260.6	398.7	341
Q4KM64	Jagn1	953	1125.1	311.7	401.2	1161.5	1014.6
Q4KM66	LOC500183	1258.3	893.7	1133.5	1069.6	1292.6	1098.5
Q4KM69	Cops5	171.6	219.6	187.5	202.4	272.9	256.2
Q4KM71	Sfpq	3589.4	4057.7	3559.2	3658.9	5215.4	5006.3
Q4KM73	Cmpk1	5626.2	5510.8	6034.2	5557.1	4185.6	4195.2
Q4KM74	Sec22b	6268.8	6393.6	4132.6	4361.1	5076	5122.8
Q4KM77	Ei24	620.7	408.2	214	242.9	330.5	314.2
Q4KM87	Actl6a	1026.6	1379.7	2291.2	2030.2	1809.2	1753.7

Q4KMA2	Rad23b	2441	2645.3	2200.5	2217	2612.3	2706.1
Q4QQS4	Ruvbl2	2933.9	3297.6	4846.7	4851.6	4447.8	4622.7
Q4QQS6	Alg5	1381.7	1366.5	499	453.9	959.8	1168.8
Q4QQS7	Umps	869.6	1038.3	995	1123.6	1055.7	1013
Q4QQS8	Nup85	105.5	117.4	212	232.6	184	161.1
Q4QQU5	Yipf6	302	254.5	159.8	134.2	160.3	156.6
Q4QQU6	Smndc1	626.8	604.1	1110.8	903.4	545.4	588.2
Q4QQV4	Hars	4103.1	4178.1	4982	4740	3881.7	3790.2
Q4QQV6	Lsp1	1307.5	1353	2680.3	2870.1	2738.9	2984.2
Q4QQV8	Chmp5	1341.4	1214.1	1930.9	2019.4	1387.7	1352
Q4QQW1	Reep4	133.3	178.7	663.5	997	187.9	181.2
Q4QQW3	Adhfe1	1706.3	1692	2636.1	3126.6	1232.5	1229.2
Q4QR73	Dnaja4	80.3	115.7	228.9	207.5	84.7	93.2
Q4QR75	Exosc9	412.3	515.9	898.6	904.9	628.7	597.6
Q4QRB8	Asl	1512.1	1691.3	1333.3	1607.5	2060.6	2204.7
Q4R1A4	Tfg	1255.6	1448	1452.3	1010.1	1310.1	1317.2
Q4V7C6	Gmps	1603.6	1566.7	1895.4	1746.5	1610	1623
Q4V7D9	Smpdl3b	519.7	420.9	593.5	649.9	505.3	509.1
Q4V7E8	Lrrfip2	255.7	266.6	686	488.3	323.1	333.5
Q4V7F2	Creld1	198.6	200.5	260.7	235	195.3	191.6
Q4V885	Colec12	199	224.5	460.8	477.5	346.5	346.9
Q4V886	Paf1	338.5	423.9	631.5	471.8	580.6	536.3
Q4V892	Sigirr	243.2	272	233.9	204.6	248.8	235.3
Q4V897	Ccdc90b	360.7	473.2	478.8	438.4	593.2	624.6
Q4V898	Rbmx	1372	1344.3	1429.6	1436.5	1568	1581.6
Q4V899	Tmem165	320.8	415.8	386.1	320.1	485.4	458.6
Q4V8B0	Oxr1	1307.9	1306	795.8	791	1140.1	1077.8
Q4V8C2	Zw10	604.1	829.4	1700.4	1423.4	720.6	737.6
Q4V8E2	Psmd14	817.5	901.2	1253.6	1129	974.7	943.1
Q4V8F5	Tada3	37.9	52	75.1	104.6	64	59.6
Q4V8H2	Exoc1	318.7	357.9	523	459	664.2	607.8
Q4V8H5	Dnpep	3272.6	2917.4	3313.9	2959.4	2613.1	2631.8
Q4V8H8	Ehd2	5613.3	5454	3010.1	3094.9	6108.8	5945.1
Q4V8I6	Rpl11	8955.7	9767.3	12141	14118.1	6499	6805.8
Q4V8K2	Ctnnbl1	35.8	81.7	57	64.4	259.2	237.5
Q4VBH2	Trnt1	555.3	530.3	320.9	314.5	527	572.6
Q505J8	Farsa	2250.3	1877.9	1717.1	1518	1106	1233.5
Q505J9	Atad1	238.7	258.6	239	234	302.6	304.6
Q52KJ9	Tmx1	359.3	573.5	604	944.5	1134.4	994.5
Q52KK3	Slc25a51	142.8	164	131.2	146.9	195.1	161.6
Q53UA7	Taok3	515.3	495.9	455.5	486.7	593.8	531.6
Q561R9	Lactb2	628.2	702	552.1	589.6	758.2	798.5
Q561S0	Ndufa10	1549.6	1694.7	1588.7	1497	1331.3	1273.6
Q562A2	Zfr	280.1	337	584.4	522.1	367.3	381
Q562B3	Nckap1	954.8	989.9	747.6	797.4	833.8	913.8
Q562B5	Pgam5	475.9	471.6	751.4	562.4	499.5	485.4

Q562B9	Pnpt1	401.4	483	737.5	729.6	565	530.1
Q562C6	Lztfl1	428.9	477.9	769.1	716.1	449.3	481.1
Q562C9	Adi1	2585.8	3156.2	4803.5	3884.4	3135.1	3221.4
Q566Q8	Bles03	451.2	443.1	556.4	470.4	466.6	453.4
Q568Z5	RGD1307526	193.5	217.7	215.4	213.7	211.4	247.6
Q568Z6	Ist1	710.9	672.6	1196.9	1018.1	708.5	704
Q569B4	Ighg	839.5	920.5	2270.6	2180.7	1315.6	1447.1
Q569C9	Golph3	363.6	555.1	670.6	656	443.3	474.1
Q56A18	Smarce1	173.8	236.9	412.9	364.4	269.8	276.2
Q56A27	Ncbp1	306.2	328.1	206.7	221.1	455	432.9
Q56B11	Pelp1	37.8	40.6	35.2	22.7	50.6	46.7
Q56R17	Kpna4	471.1	520.8	599.2	516.9	732.6	742.8
Q58FK9	Kyat3	2759	2792.6	2600.4	2808.9	2546.6	2448.1
Q5BJK8	Golim4	363.2	400.1	933.4	573.9	502.2	435.2
Q5BJN1	Stard10	553.5	421.5	459.8	426.6	320.4	289.3
Q5BJN3	Tial1	1511.6	1563.2	2096.4	2024.8	1935	1952
Q5BJN7	LOC100360647	64.2	73.3	50.8	58.2	51.8	48.3
Q5BJP3	Ufm1	1216.7	1177.5	512	482.5	809.5	778
Q5BJP4	Rbm39	2057.7	1931.4	1822.6	1935.5	2189.9	2171
Q5BJQ0	Coq8a	120.5	171.1	163	212.1	131.4	158.4
Q5BJQ2	Mindy1	98.6	109.9	165	219.5	157.2	140.9
Q5BJQ6	Cstf1	352.7	471.8	406.7	417.7	515	486.7
Q5BJT0	Arglu1	293.6	328.1	241	261.2	473.2	478.7
Q5BJT6	Lsg1	158.2	187.9	188.4	248.9	227	203.6
Q5BJU0	Rras2	182.4	75.4	124.5	125.8	72.5	79.4
Q5BJX1	Mrpl41	220.9	260.6	196.8	179.3	292.1	257.6
Q5BJY9	Krt18	14996.2	13011	16145.6	17056.2	19693.6	19756.8
Q5BJZ3	Nnt	18360.9	19563.2	15763.4	14745.7	15188.3	14585.8
Q5BJZ6	Carnmt1	293.1	320.8	398.5	438.8	452.3	405.6
Q5BK08	Clec4m	330.5	406.5	718	788.2	667.1	630.3
Q5BK18	Ciao3	61.9	77	87.4	77.3	91.7	97.2
Q5BK33	LOC652956	495	620.6	731.8	716.5	768	749.5
Q5BK81	Ptgr2	557.2	667.1	613.2	646.5	693.6	675.2
Q5BKA1	Metap2	4098.4	4337.6	3305.8	2908.9	4095.3	4268
Q5BMA6	Arhgef12	353.9	387.4	374.9	361.4	498.6	497.3
Q5D023	Dync1li2	1566.8	1692.3	1686.7	1364.2	1683.6	1568.7
Q5D059	Hnrnpk	7651.9	8872.2	11400	11050.2	10908.7	10806.1
Q5EB77	Rab18	2696.6	2739.5	2335.4	2821.6	2387.4	2360.4
Q5EBA7	Hgfac	217.9	268.8	518.2	504	274.4	282.4
Q5EBC0	Itih4	2880.4	3331.7	6172	6206	5801.4	5500.9
Q5EBC7	Rabep2	941.1	1594	615.7	624.4	1730.8	590.6
Q5EBD4	Dcps	125.7	157.6	123.6	139.2	190.1	171.7
Q5EGY4	Ykt6	305.5	317	128.9	201.6	313.2	356.5
Q5FVC4	Dnajb12	334.9	365.1	767.9	893.8	408.9	369.6
Q5FVG5	Tpm2	4826.2	4332.2	3053.8	3014	5666	6530.3
Q5FVI6	Atp6v1c1	1925.1	2175.5	3243.8	2757.2	2634.9	2567.6

Q5FVK6	Cc2d1b	313.8	347.5	255.9	281.7	467.3	475.1
Q5FVL2	Emc8	1185.7	1549.3	2456.2	2552	1619.9	1635.6
Q5FVM4	Nono	1921.8	1963.8	1582.1	1820.4	2259.7	2079.5
Q5FVM7	Dnajc16	115.4	144.7	206.5	211.1	148.3	149.9
Q5FVN0	Lpcat3	625.4	764	976	1099.2	984.8	812
Q5FVQ0	Slc39a8	339.6	456	825.1	721.5	1094.6	850.5
Q5FVQ6	MGC108823	476	561.2	853.3	748.3	868.8	993.2
Q5FWT1	Fam98a	1156.8	1051.1	976.9	1071.7	855.5	847
Q5FWU0	Wasf2	835.9	267.1	337.6	362.6	320.3	289.9
Q5FWU2	Nfs1	170.6	200.5	2019.3	1466.2	177.6	162.7
Q5FWY5	Aip	200.1	272.7	574.5	559.4	454.7	395.6
Q5HZA9	Tmem126a	175.1	195.7	244.7	230	223.4	210.7
Q5HZE2	Tmem120a	685.7	442.7	345.6	390.4	369.8	381
Q5HZE4	Mri1	1753	1623.4	1020.6	1178.5	1238.3	1303.1
Q5HZV9	Ppp1r7	98.7	128.4	123.8	127	156.8	146
Q5HZX2	RGD1359201	173.2	215.7	622.1	610.7	202	196.1
Q5HZY0	Ubxn4	523.4	554	431	433	569.1	571.7
Q5HZY2	Sar1b	726.5	1002.6	1235.5	1059.2	910.8	874.1
Q5I0D1	Glod4	4084.6	3926.7	5665	5021.1	4554.4	4361.1
Q5I0D7	Pepd	2659.7	1888.5	2502.2	2313.7	1911.4	1989.2
Q5I0E7	Tmed9	1790.3	2172	2593.3	2584	2064.8	2041.6
Q5I0F0	Drg1	2585.8	2416	1933	2031.6	2085.6	2116.2
Q5I0G4	Gars1	5798.2	6865.5	5528.1	5307.6	6659.5	6536.8
Q5I0H9	Pdia5	2579.6	2852	1727.4	1847.7	2623.1	2591.4
Q5I0K4	Mrps9	186	240.6	495.5	360	234.1	233.2
Q5I0K7	Alg13	271.8	308.7	378.1	431.8	340.6	291.7
Q5I0K8	Mrps7	125.6	122.3	77.4	103	123.4	121.5
Q5I0L3	Yars2	468.7	560.5	388.7	399.4	348.6	418.6
Q5I0L7	Kti12	214.6	236.2	260.7	290.6	237.2	230.7
Q5I0M3	Cfhr1	37.5	34.5	41.7	40.6	49.7	39
Q5I0P2	Gcsh	3413	3367.6	2528.1	2616.4	2059.2	2027.4
Q5I2Z0	Pacsin3	107.6	125.7	155.9	192.4	155.2	118.9
Q5JC29	Eps15	182.5	234.6	346.3	357.4	412.1	349.5
Q5M7A4	Uba5	1801.6	1503.2	1831.3	1898.4	1171.1	1113.8
Q5M7T1	Ciao1	204.3	194.4	218.4	216.3	170.8	162.7
Q5M7T5	Serpinc1	1699.8	2130.5	2242.7	2355.4	3278.1	3469.8
Q5M7T6	Atp6v0d1	1372.3	895	392.3	394.8	739.4	796.2
Q5M7T9	Thnsl2	285.8	290.6	399	452.3	315.3	262.8
Q5M7U6	Actr2	1298.5	1288.9	1091.3	1093	1345.4	1327.8
Q5M7V3	LOC367586	2742.8	2338	7261.5	5821.3	4151.9	3603.9
Q5M7V8	Thrap3	587.7	654.3	836.4	804.7	853.3	764.9
Q5M7W5	Map4	1281	1411.2	2069.8	2118.2	1950	1804.3
Q5M7X1	Copb2	13913	15766.9	9564.1	9332.1	13346	13595.2
Q5M810	Myo1g	128.6	165.3	218.4	207	228.1	218
Q5M818	Mrpl16	62	77.5	135.7	138.4	92.9	74.1
Q5M819	Psph	682.6	456.2	323.2	303.6	381.7	313.6

Q5M823	Nudcd2	725.3	825.3	1209.4	1165.8	728.4	673.5
Q5M860	Arhgdib	3591.4	3623.5	3936	4040.8	6071.6	6100.8
Q5M875	Hsd17b13	17234.2	17087.5	12821.2	14830.8	12110.2	11916.2
Q5M876	Acy3	662.7	791	882.8	1018.7	781.7	753.3
Q5M883	Clic2	235.2	267.3	421.6	378.1	395.6	336.8
Q5M920	Ebna1bp2	174.6	164.7	181.1	317.9	171.7	171.6
Q5M943	Thumpd1	1001.8	1100.5	1096.4	1260.8	1519	1531.8
Q5M963	Cmas	1496.6	1586.5	1944.6	1752.6	1758.6	1666
Q5M9F7	Actr10	569.8	684.5	681.9	861.4	792.3	779.1
Q5M9F8	Scyl1	247.6	424.8	345.4	498.2	303.4	291.6
Q5M9G1	Hexim1	337.3	332.2	204.9	190.7	347.7	324.5
Q5M9H2	Acadvl	3579.4	3365.4	3324.2	3231.9	3437.3	3041.4
Q5M9I5	Uqcrh	336.1	154.2	229.8	177.3	123.2	126.3
Q5PPG2	Lgmn	781.8	780.2	1073	1029.4	1091.3	1160.7
Q5PPG7	Eif2d	1520.2	1657.9	2125.2	2319.2	1622.1	1536.4
Q5PPI1	Srsf9	314.6	252.4	216.5	279.1	395.1	382.4
Q5PPJ6	Lars	5756.4	6032.3	4831.6	4388.5	5465.5	5245.3
Q5PPN5	Tppp3	122.6	167.6	207.8	212.3	286.3	257.4
Q5PQJ6	Pycr3	598	616.1	429.4	460	634.7	621.7
Q5PQK2	Fus	1759.2	1867.8	1169.4	1170.1	2341.4	2912.2
Q5PQK5	Rdx	1726.8	1603.1	1047.4	1084.7	1466.3	1443.4
Q5PQN1	Herc4	351.6	351.2	473.8	428.2	397.8	393.6
Q5PQN7	Lzic	25.9	32.4	52.2	42.7	45.5	40.4
Q5PQP2	Ebag9	42.6	44.5	55.2	57.7	44.6	46.6
Q5PQP7	Gtf3c2	420.2	461.7	756	637	642.1	589.4
Q5PQR0	Raly	2824.5	2993.4	3437.2	3367.5	3545	3413.6
Q5PQX1	Tor1aip1	740.6	795.9	948.5	972.1	1040	1182.7
Q5PQZ9	Ndufc2	1907.1	2109.4	1907.3	2137.9	1577.6	1677.4
Q5QD51	Akap12	1417.8	1455.5	1907.6	1858.8	1603.8	1523
Q5QJC9	Bag5	259.7	324.1	586.2	697.4	425.3	356.2
Q5RJK5	Cbx3	4591.5	3722.5	3067.1	2950.2	3648.8	3930.5
Q5RJK6	Inpp1	442.2	657.3	601.7	609.3	618.7	700.6
Q5RJK8	Acbd6	165.8	222	133.9	164.8	310.2	357.3
Q5RJK9	Polr1c	152.5	186.9	228.2	208.3	224.9	238.9
Q5RJN0	Ndufs7	1443.6	1642.1	1968.2	2002.5	1727.1	1612.5
Q5RJN3	Ccl21	80.7	114.3	95.9	105.6	138.1	108.4
Q5RJR2	Twf1	1293.9	1497	1353	1302.5	1763.7	1791.4
Q5RJR8	Lrrc59	8469.8	9625.6	6594.3	6748.5	7493.2	8048.7
Q5RJR9	Serpinh1	5688.1	6309.8	5826.6	6470.8	12858.7	14247.1
Q5RJS6	Mospd1	339.5	311.2	820.3	579.9	265.4	249.3
Q5RJY4	Dhrs7b	60	67.2	73.2	103.4	62.6	61.4
Q5RK00	Mrpl46	110.4	130.4	113.5	127.4	158.7	124.8
Q5RK08	Nipsnap2	1429	1361.9	1498.5	1508.1	1164.4	1335.8
Q5RK09	Eif3g	6389	6401.1	4674.1	4614.6	4156.1	4214.9
Q5RK10	Rpl13a	14379.8	13522.5	12599.2	13230	9271.7	9541.7
Q5RK23	Abhd1	69.5	75.4	76.9	79.7	76.7	74.3

Q5RK30	Sbds	569.2	712.6	701.5	784.1	889.4	883.8
Q5RKG9	Eif4b	5253.5	3332.1	3655.6	3361.9	2839.7	2731.1
Q5RKH0	Glyr1	485.4	449.1	553	602	578.7	578.3
Q5RKH2	Galk1	76.4	102.8	79.7	109.1	153.3	122.3
Q5RKH6	Os9	1482	1487.8	1349.5	1246.5	1340.3	1340.9
Q5RKH9	Derl1	113.6	22.6	35.7	24.5	15.2	13
Q5RKI0	Wdr1	7800.1	8321.9	7328.6	7790.8	11822.5	12348.7
Q5RKI5	Flii	329.6	371.1	233.5	239.6	683.6	687.5
Q5RKJ4	Fnta	56.5	74.8	65.9	77.5	58.3	65.4
Q5RKJ9	Rab10	1050	917.9	396.4	453.1	494.2	628.7
Q5U1W6	Apool	320.3	345.1	451.4	359	281.1	267.3
Q5U1W8	LOC100911295	233.7	313.9	322.5	286.7	490.9	486.2
Q5U1Y2	Rac2	109.8	92.2	248.8	216.3	137.2	145.3
Q5U1Y3	Fblim1	98.3	125	150.4	197.4	145.1	151.2
Q5U1Z2	Trappc3	679.8	612.1	590.1	498	562.8	615.7
Q5U1Z9	Mtx2	1124.6	1259.4	1832.7	2442.7	1143	1160.8
Q5U211	Snx3	1306.2	1226.9	1543.6	1864.4	1610.4	1653.5
Q5U2N0	Ctps2	322.4	381.2	541.3	550.9	421.3	439
Q5U2N2	Usp14	3168	3609.3	3200.1	2672.5	3545.8	3831.2
Q5U2Q3		605.7	671.7	800.9	843.3	654.6	599.8
Q5U2Q5	Rrm1	678.1	724.7	1676.4	1418.8	920.1	988.7
Q5U2Q7	Etf1	8839	9834.3	6945.9	7362.8	9307.8	8922.4
Q5U2R4	Trmt10c	209.7	213	184.5	157.8	246.2	217.3
Q5U2R7	Mesd	1055.5	1160.7	1122.9	989.2	1258.1	1166.7
Q5U2R8	Mnda	629.6	841.6	1364.4	1358.8	1404.3	1455.8
Q5U2R9	Scfd2	727.1	757.1	1030.7	926.4	783.7	692.5
Q5U2S7	Psmd3	3553.1	3111.9	3479.8	3199.5	3080.9	3006.2
Q5U2T9	Fkbp5	1113.1	1221.8	1656.5	1565.2	1759.4	1714.2
Q5U2U0	Clpx	1854.8	2119.2	2402.4	2104.7	1958.8	1917.2
Q5U2U2	Crkl	369	457.2	503.6	498.8	513.5	497.4
Q5U2U3	Parp3	324.9	307.7	250.9	179.5	419.5	480
Q5U2U7	Rnmt	881.7	979.2	1378.4	1381.9	1208.5	1228.6
Q5U2V1	Fkbp10	801.9	1021.8	1041.2	1028.4	1672.8	1974.2
Q5U2V8	Emc3	297.1	331.5	228.4	203.6	308.5	309.2
Q5U2X6	Ccdc47	2837.4	2580.6	1750.1	1884.6	2122.3	2080.2
Q5U2X8	Acot9	683.5	786.3	865.9	814.9	513.4	497
Q5U2Y6	Tfip11	18.2	27.1	55.8	69.6	38.8	28
Q5U2Z4	Nfkb2	128.9	126.7	155.6	178.9	126.6	141.6
Q5U2Z5	Cmtr1	150.1	178.1	703.6	699.2	248.5	229.6
Q5U300	Uba1	13562.2	14042.6	13483.6	13498	14225.7	14287.2
Q5U302	Ctnna1	7389.2	8103.2	10076.6	9130.4	10153	9951.5
Q5U316	Rab35	1191.7	1203.7	1113.3	1178.7	960.5	946.2
Q5U318	Pea15	540.4	652	602.9	522.3	1156.6	1179.3
Q5U322	Cpe	315.1	357.3	464	652.1	338.8	353.2
Q5U328	Ncl	19457.1	19205.7	11458.4	10802.9	20091.3	21526.7
Q5U355	Itfg1	133.3	175.1	120.6	152.3	198.8	159.8

Q5U362	Anxa4	6328.7	6004.6	4501.3	5196.7	7153.1	7124.7
Q5U3Y7	Tmem97	4408.6	6287.9	3166	3437.5	5836.1	4291.2
Q5U3Y8	Btf3	2312.5	2367.6	1214.7	1108.5	2172.7	2202.2
Q5U3Z3	Isoc2	43.6	69.9	86.1	64.4	65.5	61.7
Q5U3Z7	Shmt2	1278.8	1701.5	1185.2	1248.9	1581.8	1697.8
Q5UAJ5	ATP8	995.6	1243.4	1115.6	1098.6	1186.5	1193.6
Q5UT78	RT1-Db	82	297.8	243.5	412.3	397.1	671.2
Q5VLR5	Erp44	2339.9	2146.4	1377.2	1553.6	1862.4	1793.1
Q5VLR6		773.5	505.6	672.7	789.6	1276.3	809.4
Q5WRG2	Ang	1142.5	1316.6	1346.3	1258.2	1785.9	1859.4
Q5XFV4	Fabp4	14996.4	10809.6	10425.7	9019.8	11145.9	10900.8
Q5XFV9	Rps16	4679.2	4213.3	3141	4359.9	4415.8	3634.9
Q5XFW1	Mbp	148.4	178.8	170.8	171.6	131.7	143.3
Q5XFW4	Mrpl13	189.9	186.5	149.5	303.6	239	240.3
Q5XFW8	Sec13	2541.7	2863.6	2787.9	2745.8	3374.1	3226.1
Q5XFX0	Tagln2	7647.2	6388.2	6221.2	6879.3	9279.6	9936.6
Q5XHY0	Ddx18	467	558.1	661	696.8	613	612
Q5XHY7	Stam2	100.4	108.4	62.3	54.1	86.7	84.5
Q5XHZ0	Trap1	1023.4	1320	1624.4	1619.8	1414.1	1339.4
Q5XI07	Lpp	529.4	598.7	494.9	541.7	887.5	829.2
Q5XI15	Arih1	323.6	164	229.8	218.3	272.5	193.5
Q5XI18	Acbd3	875.4	969.8	415.9	599.6	982.5	905.8
Q5XI19	Fermt2	1821	1684.2	1507.2	1676.7	2269.4	2090.8
Q5XI20	Mturn	159.5	205.1	197.1	172.8	199.6	194.6
Q5XI21	Tom1	328.8	431.2	333	297.2	366.5	341.1
Q5XI22	Acat2	840	974.1	1040.3	932.8	960.7	1079.5
Q5XI28	Raver1	247.6	286.7	472.9	486.3	328.4	311.7
Q5XI29	Cpsf7	444.4	450.2	419.3	443.1	518.3	533.3
Q5XI32	Capzb	2990.2	3080.5	2532.2	3134.7	3071.4	3562.7
Q5XI34	Ppp2r1a	4848.5	4459.9	4944.9	4932.7	5063.2	5326.6
Q5XI36	Adgre5	118.1	132.2	241	221.9	165.1	155.6
Q5XI38	Lcp1	8899.2	8948.7	9624.3	8862.8	14516.5	15924.3
Q5XI41	Tram1	679.9	515	424.1	453.5	355.2	282.2
Q5XI55	Ngly1	83.6	120.7	188.6	187.1	164	130
Q5XI64	Abhd6	450.9	455.7	364.4	372	469	447.3
Q5XI73	Arhgdia	7960.5	8574.9	9055.8	9597.3	11806.4	12115.4
Q5XI77	Anxa11	2655.8	2388.9	2647.8	2800.3	3063.9	2894.5
Q5XI78	Ogdh	2673.2	2941.9	3727.5	3338.3	4133.4	3715.4
Q5XI83	Vps35l	377.1	460.3	514.8	520.3	535.1	541.2
Q5XI85	Amt	831.2	983.6	489.1	559	592.2	542.5
Q5XI86	Ptrh2	313.9	309.7	597.4	521.1	289.2	270.9
Q5XI90	Dynlt3	341.7	346.4	206.9	169.6	317.1	301.4
Q5XI97	Aarsd1	22.2	34.7	39.4	34.4	28.7	28.3
Q5XIA5	Coasy	1534	1432.4	1589.4	1398	1262.6	1218
Q5XIA8	Ghitm	350.9	481.9	323.6	291.8	564	542.5
Q5XIB4	Ufsp2	639.8	872.7	692.6	876.9	1056.1	1016.5

Q5XIC0	Eci2	790.8	917.3	1681	1224.8	995.8	1056.3
Q5XIC1	Gmppa	2231.9	2376.1	2575.7	2526.4	2044.9	1932.1
Q5XIC6	Psmd12	678.8	896.7	1212.4	895.8	702.4	718.5
Q5XID1	Ciapin1	587.3	489	178.1	174.3	562.2	581
Q5XID7	Armcx3	368.5	529.4	647.8	590.8	587.6	570.6
Q5XIE6	Hibch	975.1	864.5	940	723.8	598.2	551.1
Q5XIF3	Ndufs4	1192.7	1425.8	1423.2	1122.2	1215.9	1187.5
Q5XIF6	Tuba4a	3901.3	4625.3	2979.4	3025.2	4211	4628.2
Q5XIG0	Nudt9	478.5	521.8	744.8	724.2	757.3	702.4
Q5XIG4	Ociad1	352.1	242.6	384.1	333.8	253.7	219.5
Q5XIG8	Strap	1034.2	1210.8	1785.8	2103.1	1338.7	1250.8
Q5XIH1	Aspn	1384.7	2182.2	2480.7	2577.9	2698	2488.3
Q5XIH3	Ndufv1	4448.4	4904.8	5757.5	5747.8	3637.9	3662.4
Q5XII2	Gps2	203.1	394.8	628.1	683.1	637.7	836.3
Q5XIJ7	Cab39l	134.3	177.2	115.6	148.1	383	361.2
Q5XIL1	Atp6v1h	400.5	361.3	213.3	339.2	357	382
Q5XIL4	Sorbs3	51.6	64.6	153.4	144.1	81.9	65.5
Q5XIM0	Bcs1l	277	319	716.2	667.2	350.5	321.3
Q5XIM5	Cdv3	2244.5	2286.2	458.2	423.8	1454.7	1611.6
Q5XIM7	Kars1	6884.5	7302.9	6797.3	6061.9	6980.8	6497.2
Q5XIM9	Cct2	3987.7	4097.9	5104.7	5134.4	4471.4	4619.5
Q5XIN6	Letm1	1220.1	1166.8	1406.1	1308.7	887.1	926.5
Q5XIP0	Dnajb4	82.3	83.4	141.5	113.7	101.5	94.1
Q5XIP1	Pelo	778.8	566.2	170.1	152.5	288.1	343.2
Q5XIP2	Pigk	179.5	199	589.8	477.7	293.2	282.9
Q5XIP6	Fen1	533.3	379.6	302.6	466.4	290.1	352.4
Q5XIP9	Tmem43	1046.7	940.7	1083.6	1240.7	1655.8	1427.1
Q5XIT9	Mccc2	10712.5	8891.1	8188.7	8014.1	7228.7	6415.3
Q5XIU9	Pgrmc2	443.6	321	201	299.6	287.7	283.9
Q5XIW0	LOC100362432	214.8	283.3	238.7	326	305.3	282.1
Q5XJW2	Gadd45gip1	525.7	566.6	1151.1	1206.9	489.6	500.4
Q60587	Hadhb	4043.2	4860.2	3502.9	3401.9	5240.1	4870.2
Q62622	Eif4ebp1	138.3	164.3	171.2	133.2	145.8	130
Q62636	Rap1b	2116.8	2119	1608.4	1834.5	2537.8	2827.8
Q62639	Rheb	679.9	675.1	385.2	369.2	613.2	601.6
Q62651	Ech1	807.8	898.7	882.1	906.7	1027.6	1033.6
Q62658	Fkbp1a	967.2	595.6	792.4	709.1	494.6	510
Q62667	Mvp	6670.2	6454.9	7245.5	6963.8	9719.1	9501
Q62703	Rcn2	1546.3	1712.6	2096.9	1818	1873.9	1681.7
Q62733	Tmpo	3126.5	2233.2	2306.7	2228	2361.8	2426.1
Q62745	Cd81	299.2	420.1	309.7	447.3	478.4	487.1
Q62753	Stxbp2	427.4	554.6	774.3	696.4	768.4	753.6
Q62768	Unc13a	64.9	72	676.1	684.1	125.4	71.5
Q62780	Ddx46	1644.8	1832.2	1962.3	1937.3	2287.8	2128.2
Q62785	Pdap1	2811.7	3257.3	3173.1	2868.3	3186.4	3066.6
Q62796	Ralbp1	109.7	155.5	217	212.3	212.2	186.7

Q62819		345.1	419.4	500.8	549.9	342.1	297.4
Q62825	Exoc3	82.9	133.9	223.9	219.9	191.8	179.1
Q62839	Golga2	762.9	763.8	771.6	733.4	630.7	647.9
Q62862	Map2k5	1231.4	1018.5	2356.2	1974.9	1232	1198.1
Q62867	Ggh	3440	2897.1	1884.9	1821	1935.7	1770
Q62868	Rock2	567.7	626.3	385.4	424.8	775.8	825.6
Q62881	Nol3	74.7	101.5	201.4	193	114.8	95.3
Q62902	Lman1	6776.8	7119.7	6635.7	7542.1	5717.7	5468.8
Q62920	Pdlim5	401.3	445.6	291.1	287.9	622.2	614.8
Q62930	C9	1034.9	1310.7	2131.4	1938.9	1756.2	1899.5
Q62952	Dpysl3	1132	966.7	858.1	981.9	1127.5	1249.3
Q62975	Serpina10	472.8	564.1	872.1	828.8	606.8	587.4
Q62991	Scfd1	1592.1	1842.1	1453.4	1534	1570.1	1607.2
Q63009	Prmt1	2229.1	2778.3	3285.2	3098.3	5019.8	4895.6
Q63011		4200.9	4818.2	9094	9536.5	7531	6354.7
Q63014	Akap8	120	135.9	156.6	80.9	172.1	153.4
Q63016	Slc7a5	507.7	617.6	285.4	249.3	345.5	347.8
Q63041	A1m	14626.6	15551.6	48761.4	48441.2	19829.6	19489.9
Q63055	Arfrp1	163.6	179.5	99.3	111.9	176.5	166.7
Q63065	Pdk1	1007.5	1091.7	827.7	851.9	793.7	782
Q63083	Nucb1	470.2	522.6	613.4	544.9	719.3	760.2
Q63135	Cr1l	1338.4	1374	1613	1579.7	1431.8	1418.1
Q63184	Eif2ak2	821.3	1039.6	1080.4	1200.8	1052.1	1064.9
Q63186	Eif2b4	1327.6	1098.9	1418.4	1402.3	901.3	879.3
Q63269	Itpr3	236.7	343.3	589.2	574.5	503.8	419.4
Q63279	Krt19	4615.6	5381.3	6413	5885.7	7018.7	8888.3
Q63318	LOC100911104	114.1	141.6	277.6	217.5	162.4	164.4
Q63355	Myo1c	3113	2736.9	2704.8	2661.4	2627	2616.3
Q63356	Myo1e	735.3	697.6	1191.1	1249.1	890	884.7
Q63357	Myo1d	972.4	1090.7	1055.9	1039.9	1272.2	1196
Q63377	Atp1b3	1249.3	859.3	813.2	840.3	769.5	731.3
Q63396	Sub1	2440	2400.1	2724	2878.1	3053	2843.7
Q63400	Cldn3	189.5	219.6	149.9	121.1	297.4	247.2
Q63413	Ddx39b	1138.6	839.5	1081.3	1126	1056.5	948.4
Q63433	Pkn1	1133.7	1051.1	831.6	830	924.8	970
Q63448	Acox3	70.1	74.6	119.9	202.4	107.8	97.9
Q63450	Camk1	471.6	554.9	195	222.7	553.6	598.9
Q63507	Rpl14	3093.7	2820.9	2029.1	2106.1	2194.4	2119.8
Q63524	Tmed2	669.3	645.5	533.5	575.5	514.6	454.3
Q63530	Pter	876.8	886.6	891.3	988	751	727.9
Q63547	ZG-21	155.3	66.3	178.1	106.4	38	35.4
Q63570	Psmc4	1694	1748.6	1831.1	1947.7	1804.8	1825.3
Q63581		375.7	399.5	566.5	552.3	1702.1	1620.9
Q63584	Tmed10	7125.1	5286.7	5367	5848.9	3535.9	3766.4
Q63598	Pls3	1072.5	1315.1	1402.4	1512.7	2060.1	2047.9
Q63610	Tpm3	4179.3	4004.7	4646.4	4517	5708.5	5891.7

Q63635	Stx6	331.7	464.1	446.8	494.4	429.2	487
Q63639	Aldh1a2	883.7	1024.2	964.3	870.6	1828.5	2049
Q63663	Gbp2	568.4	674	1227.8	1254.6	982.3	1061.6
Q63688	Cyp7b1	1324.6	2052.1	1199.6	1392.2	2769.1	2955.5
Q63691	Cd14	60.1	59.8	71	77.7	92.8	96.6
Q63714	Golgb1	4129.3	4628.2	5641	5188.3	4452.9	4604.4
Q63747		1201	1113.5	891.5	906.5	1171.8	1094.9
Q63768	Crk	955.3	1419.9	1949.2	1926	1878.2	1633
Q63798	Psme2	1118.5	1216.8	1411.3	1236.1	2623	2225.1
Q63942	Rab3d	1012.6	1067	765.2	793.4	969	911.2
Q63965	Sfxn1	196.8	164.7	63.2	47.9	118.3	162.3
Q64057	Aldh7a1	12424.3	14640.7	10717.7	9105.9	10442.1	10798.4
Q641W2	Myg1	579.9	470.9	303.7	440.3	525	544.5
Q641X5	Agpat6	164.4	187.5	153.4	142.9	172.4	181.1
Q641X8	Eif3e	5188.5	6322.1	6137.4	5761.9	5728.9	5642.9
Q641Y0	Ddost	4063.2	2247.1	2229.6	2334.5	1817.8	1801.3
Q641Y2	Ndufs2	2279.1	2351.6	1710.7	1825.8	1599.3	1762.4
Q641Y5	Atg7	205.5	272.7	273.3	292.2	285.7	295.1
Q641Y8	Ddx1	5240.3	5755.8	4747.6	5209.6	5534.2	5548.7
Q641Z2	Ptpn9	82.1	82.8	130.7	158.8	111.4	100.4
Q641Z6	Ehd1	136.9	149.8	185.9	192.8	198.5	223.6
Q64232	Tecr	6640.1	7608	5316.9	5393.5	5582.8	4975.5
Q64240	Ambp	634.9	691.2	857.4	1018.1	961.3	930.6
Q642A6	Vwa1	62.3	66	43.9	59	149	125.7
Q64303	Pak2	2315.6	2446.4	3030.9	2811.3	2773.6	2857.6
Q64350	Eif2b5	575.7	554.7	461.9	419.7	464.8	445.5
Q64361	Lxn	1707	912.6	1648.9	1904.9	922.8	917.7
Q64380	Sardh	3526.3	3744.5	3558.5	3917.4	2838.9	2743.6
Q64428	Hadha	8051.4	8891.9	8208.4	7970	7806.8	7999.6
Q64560	Tpp2	1703.4	1878.9	2010.6	1986.4	1874.2	1830.4
Q64571		430.9	530.9	208.7	219.3	454	468.4
Q64591	Decr1	2993.2	3568.6	3370.4	3593.3	2704.6	2647.9
Q64599	LOC286987	440.1	537.2	6399.7	7501.4	573.8	439.6
Q64715	Map2	268.9	256.6	217.4	209.9	178.4	177.1
Q66H09	Ttc1	107.4	144.6	175.3	216.1	284.2	339.2
Q66H12	Naga	555.1	711.1	940	745.5	1121.3	973
Q66H32	Rangap1	1189.7	1312.5	1195	1270.3	1656.2	1609.1
Q66H39	Abcf3	1853.3	1452.7	1361.4	1317.3	1356.1	1256.8
Q66H59	Npl	437.8	425.9	476.5	591	445.2	374.4
Q66H61	Qars1	6804.5	7316.2	9566.6	8507.5	6983	6695.1
Q66H68	Rbm47	463.1	369.5	757.2	623.6	325.3	317.3
Q66H71	Cpped1	2879	3212.3	3136.2	3078.4	2740.5	2855.3
Q66H79	Trim32	93.6	99.4	158.2	141.7	120.4	113.3
Q66H80	Arcn1	11540.9	12413.1	13141.4	12524.3	10666.6	10463.5
Q66H94	Fkbp9	860.9	999.1	1260.3	1186.2	1356.5	1385
Q66H98	Cavin2	1119.2	1279.3	732	677.7	1497.4	1149.2

Q66HA6	Arl8b	263.2	532.6	355.5	401.6	814.5	726.6
Q66HA7	Cbfb	251.1	388.7	968.2	677.5	581.5	607.6
Q66HA8	Hsph1	3222.8	3483.8	4052.2	4033.5	3818.2	3910.4
Q66HC5	Nup93	1485.8	1691.6	2888.3	2497.3	2405.7	2116.9
Q66HD0	Hsp90b1	16491.2	14110	11883.7	11951	11279.7	11420.1
Q66HD3	Nasp	572.7	662.4	950.6	786.9	874.7	920.4
Q66HF1	Ndufs1	2907.2	2577.2	2010.6	2325	2637.8	2403.3
Q66HF3	Etfdh	3586.3	4171.3	4589.9	5043.2	3870.1	3782.9
Q66HF9	Lrrfip1	1040.8	1001.6	2120.2	1737.1	1453.2	1674.7
Q66HG4	Galm	443.6	448.5	849.9	906.1	545	471
Q66HG5	Tm9sf2	2745.2	3003.1	1810.7	1541.5	2544.3	2852.3
Q66HG6	Ca5b	317	365.7	470.6	427.4	424.5	358.4
Q66HG8	Ik	276.4	336.6	551.2	464.8	391.5	367.9
Q66HG9	Mavs	517.8	588.5	755.8	622.8	486.5	479.9
Q66HM2	Ap2a2	2086.4	2298.7	2497.4	2870.7	3632.8	3401.7
Q66HR2	Mapre1	1339	1318.9	1114	1029.4	1452.5	1493.2
Q66HT1	Aldob	4639.8	6534.5	5928.2	6222.3	6491.9	5263.2
Q66WT9	Picalm	785.3	706.1	821	774.5	897	843.5
Q66X93	Snd1	25584.5	25719.4	23715.4	20870	16486	16160
Q68A21	Purb	1421.2	1553.5	1759.2	1609.7	1472	1464.1
Q68FP1	Gsn	4322.9	3930.2	4089.3	4943.8	5977.1	5687
Q68FP2	Pon3	979.3	1043.3	863.1	1008.3	955.1	902.4
Q68FP9	Cog6	202.9	260.3	369.4	430.4	206.7	205.7
Q68FQ0	Cct5	13807.3	14871.2	14323.4	14164.8	15312	15925.8
Q68FQ7	Rpap3	557.5	567.5	568.3	570.6	711.9	716.8
Q68FQ9	Lancl2	38.7	68.7	122.5	124.1	128.3	95.1
Q68FR6	Eef1g	17971.6	13724	7287.8	8208.1	11097.7	11191.9
Q68FR9	Eef1d	4439.8	3969.4	5692.7	5629.3	3619.5	3663.2
Q68FS0	LOC298795	170.4	204.1	327.5	289.3	331.4	327.8
Q68FS1	Nubp2	398.8	414.5	411.1	397	485	457.8
Q68FS2	Cops4	4026.3	4474.8	4634.8	4478.8	4479.3	4440.9
Q68FS4	Lap3	5192.1	4665.7	2906.4	2931.7	3449.3	3772.5
Q68FS8	Rtca	195.4	193.1	131.4	120.5	222.7	218.7
Q68FS9	Commd10	214.9	221.7	103	120.5	252.1	243.1
Q68FT1	Coq9	3587.1	3938.4	4152.5	3944.4	2691.2	2580.6
Q68FT7	Farsb	5248.5	5142.4	4424	4333.7	4789.6	4782.7
Q68FT8	Serpinf2	193.4	192.4	290.7	276.5	239.6	263
Q68FT9	Scly	1583.7	1770.8	2486.9	2330.9	1564.5	1516
Q68FU2	Psat1	6622.2	8242.1	7221.2	6656.1	5735.2	5792.4
Q68FU3	Etfb	7959	8231.3	8685	8351.9	6114.3	5771.2
Q68FV6	Glmp	117.1	148.5	284.3	304.6	168.7	157.4
Q68FW4	Stx18	574.4	634.2	769.5	1018.5	513.9	548.9
Q68FW7	Tars2	803.1	844	898.8	923.6	708	673.4
Q68FW9	Cops3	1526	1714.6	3954.5	3551.6	1997.8	1806.3
Q68FX0	Idh3B	747.8	644.9	542.5	623.2	533.4	551.1
Q68FX1	Mpi	179.1	237.3	198	261.8	397.2	315.1

Q68FX4	Hcls1	19.8	21	17.9	18.9	48.1	50.6
Q68FX9	Sirt5	81.3	100	75.3	68.4	98	84.5
Q68FY0	Uqcrc1	4940.8	2411.6	2235.6	2133.7	1889.2	1838.7
Q68FY4	Gc	7946.3	7637.9	9197	10325.8	9457.6	9557.2
Q68FZ8	Pccb	10640.5	8864.1	8348.7	7696.4	5847.1	5962.1
Q68G11	Csnk2b	1004.5	1112.3	931.8	988.2	1382.7	1413.1
Q68G16	Ppp5c	1273.7	1204.3	1152.5	1165.8	1221.6	1250.7
Q68G31	Pbld	3502.3	3703.5	4247.3	4004.3	3335	3306.5
Q68G33	Gorasp2	2697.5	2147.3	2354.6	2500.7	1480.7	1555.6
Q68G38	Tor1a	241.1	380.5	225.2	171.2	397.5	439.1
Q68G41	Eci1	2989.6	2911	2352.3	2299.5	2706.8	2631.3
Q6AXM5	Cept1	1397.7	1434.8	1484.8	1635.3	1544.2	1264.6
Q6AXM8	Pon2	459.2	501.6	479.5	520.1	656.7	757.6
Q6AXN4	Ndc1	44.6	54.3	63.4	62.2	61.6	51
Q6AXQ0	Sae1	812.6	859.9	599.5	535	1213.6	1198.8
Q6AXQ5	Pde12	204.2	258.5	556.9	573.3	407	366.7
Q6AXS3	Dek	1157.8	1260.3	2234.2	1912.5	2211.5	1811.9
Q6AXS5	Serbp1	3932	3612.8	1897.5	1745.1	3209.1	2924.8
Q6AXT0	Mrpl37	44.2	71.3	121.5	168.4	101.4	78.7
Q6AXT5	Rab21	794.8	869.3	984.7	1129	1044.4	994.9
Q6AXT8	Sf3a2	203	179.5	148.5	174.3	212.1	208
Q6AXU7	Mif4gd	220.7	259.2	377.4	292.4	329.4	291.9
Q6AXV4	Samm50	1601.1	1179.7	1428.5	1507.5	1316.9	1248.3
Q6AXW2	Tmod3	2280.4	2584.1	2318.4	2291.4	3273	3234.8
Q6AXX6	Prxl2a	2085.3	2298.1	1111	1327	1224.3	1279.6
Q6AXY8	Dhrs1	292.3	263.4	140.5	144.3	337.4	341
Q6AY02	Rbm17	1130.5	1205	1720.8	1692.4	1389.2	1380.6
Q6AY09	Hnrnph2	403.6	290.3	548.9	548.5	292.4	275.3
Q6AY18	Sar1a	1773.5	1945.2	2106.2	1939.5	1655.8	1538.6
Q6AY21	G3bp2	289.2	347.9	249.7	193.8	477.9	442
Q6AY23	Pycr2	1467.4	1740.7	1543.4	1436.7	1447.8	1502.8
Q6AY24	Ubl7	38.8	58.7	58.4	57.3	65.8	53.5
Q6AY25	Tmed3	742	850	386.4	377.3	976	883.3
Q6AY30	Sccpdh	2628.1	3013.4	2673.2	2708.9	2736.7	2682.5
Q6AY55	Dcakd	148.8	208.1	396	398.6	343.8	319.7
Q6AY57	Wipi2	341.4	380.2	547	505.2	486.9	472.9
Q6AY58	Bcap31	4906	4876.4	6836.9	5983.5	4455.7	4166.7
Q6AY65	Arfip2	596.8	832.1	1091.6	1022.2	761.8	728.8
Q6AY86	Vps26a	446.5	466.4	1111	953.7	557	517.8
Q6AY90	Fam192a	94.3	70.4	67.3	71.2	64.7	63.4
Q6AY98	Ube2e2	492.6	564.7	678.7	644.9	793.4	736.8
Q6AYA6	Cybc1	23.5	31.8	26.8	39	29.3	36.6
Q6AYA7	Rfk	695.9	704.8	355.7	393.8	692.2	758.9
Q6AYB2	Sphk2	144.2	161.5	347.2	362.8	217.7	179.3
Q6AYB5	Srp54	6479.4	6503.1	5968.7	6376.8	5742.4	5235.8
Q6AYB8	Blzf1	42	67.3	138.7	117.3	56.7	58.7

Q6AYC4	Capg	135.4	138.5	203	197.6	281.5	272.6
Q6AYC8	Sh2d4a	876.4	1040.3	1647.3	1336.3	1019.5	989.1
Q6AYD3	Pa2g4	10985	11108.5	8699.6	7869.6	9367.8	9496.9
Q6AYD5	Gspt1	4141.1	4481.2	5825.5	5201.4	4138.3	4020.6
Q6AYD9	Nudt19	421.2	404	462.6	503	366.4	401.9
Q6AYE6	Fam98c	312	305.9	300.1	362.2	261.1	239.9
Q6AYF2	Lmcd1	326.7	322.5	629	584.9	398.3	435.7
Q6AYF8	Serpinb9	461.4	493.2	850.3	628.6	730.8	726
Q6AYG3	Prune1	103.9	130.7	95.9	111.9	166.1	142.3
Q6AYG5	Echdc1	2507.8	2758.7	3667.5	3648.2	2651.7	2473.2
Q6AYI1	Ddx5	6577.6	7535.4	8413	8410.3	8430.5	9760.4
Q6AYK3	Isyna1	3839.4	3502.4	4352.6	4449	2811.5	3129.5
Q6AYK5	Lyar	167.7	171.7	287.9	250.3	254.1	206
Q6AYK6	Cacybp	3309.3	3350	3073.5	3143.8	3528.3	3431.7
Q6AYK8	Eif3d	3204.7	4540.7	2889	2922	3919.3	4037.1
Q6AYQ4	Tmem109	1525.5	1717.1	2704.1	2083.2	1860.6	1597.1
Q6AYQ8	Fahd1	784.8	984.3	1164.1	1144.1	834.5	862.5
Q6AYQ9	Ppic	134.9	161.9	155.4	137.2	346.6	422.4
Q6AYS2	Sfxn1	52.9	61.6	57	56.3	54.9	53
Q6AYS3	Ctsa	90	99	176	168	184.6	149
Q6AYS4	Fuca2	94.2	75.7	47.4	62	62.7	58.9
Q6AYS7	Acy1a	2316.9	1206.1	1552.8	1476.1	1227.4	1100.2
Q6AYS8	Hsd17b11	1161.6	1267.6	1559	1522.6	1559.4	1521.4
Q6AYT0	Cryz	144.9	180.8	268.3	264.6	220	197.7
Q6AYT3	Rtcb	4102.9	4322.7	4943.3	4358.9	4362	3843.6
Q6AYU2	Pcbp2	3845.8	4072.3	2443.6	2747.8	3882.7	3720.2
Q6AYU3	Dnajb6	459.1	475.9	628.5	583.3	697.3	664.2
Q6AYY8	Slc33a1	1178.1	1144.5	1006.2	1454.2	887.1	917.1
Q6AYZ1	Tuba1c	489.3	417.7	514.5	501	401.7	448.7
Q6AYZ7	Prpsap1	573.2	788	1399.9	1244.5	954.5	853.7
Q6AZ26	Ctbp1	107.2	146.1	218.4	231	211.3	240
Q6AZ33	Blvra	2831.5	3004.8	3393.5	3869.9	3279.5	3215
Q6AZ44	Acot8	62	80.1	174.9	138.6	127	103.9
Q6AZ50	Atg3	983.7	717.3	884.9	826.6	571.7	533.6
Q6B345	S100a11	115.8	127.7	115.1	117.7	255.6	254.7
Q6DGF4	Zfand6	146.6	169.1	310.6	337.4	212.2	206.6
Q6DGG0	Ppid	2009.8	1889.4	1619.8	1712.1	2080.4	2143.9
Q6DUH4	Smarca2	145.1	197.1	179.7	254.7	251.6	276.1
Q6EV70	Pofut1	59.5	92.2	98.7	121.7	138.1	117.1
Q6EV76	Fut8	405.5	501.5	318.4	335.6	384.2	360.4
Q6GMM8	Slc27a1	100.3	113.6	251.8	212.5	157.7	151.5
Q6GMN2	Baiap2	26.4	41.8	156.8	130.4	78.5	62.3
Q6GQP4	Rab31	507.5	550.7	965	890.8	758.9	761.4
Q6GQY2	Mcfd2	2913	2921	553.5	452.5	2182.8	1748
Q6GT74	Bsg	736.5	648.4	536.5	518.9	547.6	539.9
Q6IE07	Tryx5	225.8	229.2	1468.1	997.8	223.5	222.6

Q6IE52	Mug2	31.5	28.1	237.8	216.3	38.9	32.7
Q6IE67	psma3l	3136.9	3454.5	3539.5	3916.6	3898.5	3895.8
Q6IFU8	Krt17	599.5	886.6	1557.4	1626.2	1150.2	1069.1
Q6IFV9	Krt34	438.4	474.1	776.2	852.1	993.3	535.7
Q6IFW0	Krt33b	558.3	590.6	840.9	902.8	2309.1	608.3
Q6IG12	Krt7	56.2	61.5	78.7	72.8	69	85.6
Q6IMX7	Hspbp1	169.9	244	445	426.8	289.7	280.9
Q6IMY8	Hnrnpu	10500	9755.2	8161.5	8473.9	10385.5	10705.6
Q6IMZ3	Anxa6	25460.1	28012.4	28056.1	27720.9	31267.8	30506.5
Q6IN10	Ece1	153.3	178.1	408.3	372.6	290.1	251.4
Q6IN15	Acsl5	1038.4	1142.4	1441.3	1412.3	1381	1357.1
Q6IN22	Ctsb	1599	1591.7	1581.2	1929	3360.8	3453.9
Q6IRE4	Tsg101	389.3	352.2	569.7	549.9	396.4	382.7
Q6IRJ7	Anxa7	832.2	1189.7	2542	2767.1	2117.3	2186.7
Q6IRK8	Sptan1	26417	26008.6	25790.3	24815.2	29802.1	29812.6
Q6IRK9	Cpq	232.8	228.6	525.8	430.8	270.2	251.5
Q6IUR5	Nenf	97.8	93.6	57.7	67.4	123.2	121.9
Q6IUU3	Qsox1	388.3	411	436.5	340.3	422.5	459.2
Q6J1Y9	Usp19	48.6	72.3	61.8	51.3	58.7	61.7
Q6J2U6	Rnf114	123.9	122.8	132.3	127.6	131.2	128.7
Q6JBI6		1095.5	1148.5	1306	1223.2	1270.9	1195.5
Q6JE36	Ndrg1	2126.5	2437.3	963.2	1059.4	2383.4	2151.6
Q6LDP3		4044.5	3554.1	2982	2955.8	2886.3	3102.6
Q6LDS4		5085.4	5332.9	7035.4	7362.2	5239.2	4785.1
Q6MG08	Abcf1	4302.1	4575.3	5736.2	5686.5	4623.2	4210.6
Q6MG48	Prrc2a	673.2	684	1225	841.5	450.7	478.4
Q6MG51	G4	158.6	151.1	436	519.3	139.2	133.9
Q6MG60	Ddah2	1025.7	1065	934.6	933.8	1251.3	1239.1
Q6MG61	Clic1	1637	1935.4	2288.9	2235.5	3486.1	3706.3
Q6MG73	C2	404.2	410	528	658.6	688.7	735.4
Q6MG78	Stk19	12.8	13.9	21.3	31	15.3	12.5
Q6MG79	C4a	2951.9	3212	6359.9	6659.7	5118.2	5084
Q6MG85	Agpat1	104.5	99.3	163.2	167.6	128.6	98
Q6MGA0	RT1-Ba	309.3	268.3	433.1	434.8	404.1	326.3
Q6MGB4	Slc39a7	3044.2	1813.7	1059.8	896	1160.3	955.9
Q6MGC4	Pfdn6	277	106.8	124.1	142.5	94.2	90.2
Q6NX65	Pdcd10	222.9	255.9	524.2	547.1	252.8	272.7
Q6NYB8	Ifi47	417.7	491.8	903.4	831.4	569.3	578.8
Q6P136	Hyou1	25702.2	23900.1	17613.7	17886.6	17200.2	17737.5
Q6P2A5	Ak3	8388.3	8467.1	8267.2	7706.1	6173.5	5880.9
Q6P3V8	Eif4a1	3202.3	2707.3	2834.6	2611.9	2659.4	2695.3
Q6P3V9	Rpl4	18544	22756.8	15531.9	16347.3	21107.6	20852.8
Q6P4Z6	Lcmt1	309.3	346	437.2	443.3	298	284.1
Q6P4Z9	Cops8	292	261	354.3	303.8	394	378.5
Q6P502	Cct3	5272	5879	6503	5927.5	6941.5	7090.8
Q6P503	Atp6v1d	649.4	727.7	678.9	779.3	948	946.7

Q6P685	Eif2s2	7496.9	7443.8	5748.8	5885.3	6030.7	5859.8
Q6P686	Ostf1	131.7	164.6	191.1	159.4	306.9	281
Q6P6Q7	Stat1	614.5	607.3	541.3	480.3	629.7	679.8
Q6P6R2	Dld	14497.6	15008.8	9411	8053.3	9463.7	9336.9
Q6P6S2	Slc39a11	214.8	222.7	528.5	281.7	238.4	209.9
Q6P6S4	Sil1	97.8	132.8	126.6	139.6	140.6	130.7
Q6P6T4	Eml2	1344.1	1040	1126.1	1064.6	891.5	857.1
Q6P6T5	Ocln	112.9	119.2	82.2	90.9	116.4	96.3
Q6P6T6	Ctsd	3624.2	4565.7	5380.5	6073.4	7132.1	7174.4
Q6P6U2	Psmc3	5519.5	5895.1	6523.6	7053.1	6239	6288.7
Q6P6V0	Gpi	4118.1	5126.8	7464.8	7084.5	7382.9	6815.9
Q6P725	Des	2370.7	1918.8	2773.9	2668.7	2383.5	2540
Q6P734	Serping1	349.3	410.9	942.1	802.6	742	759.5
Q6P742	Plp2	22.2	22.7	12.1	5	38.5	38.5
Q6P757	Eef2k	105	137.8	163.9	188.3	172.8	127
Q6P762	Man2b1	409.5	394.7	667.4	688.8	410	377.2
Q6P769		579.5	812.6	446.6	469	1102.3	1092.6
Q6P777	Mvb12a	244.1	236.9	191.4	182.7	343	323.2
Q6P790	Rpl6	15502.5	14683.2	13396.6	12340	10320.4	10642.8
Q6P791	Lamtor1	162.9	123.6	71	69.4	139.3	119.9
Q6P792	Fhl1	1915.1	2234.8	2239.4	2085.2	2494.1	2583.8
Q6P799	Sars1	5264.6	5498.7	5238.8	5161.1	4815.2	4697.7
Q6P7A7	Rpn1	14678.2	16934.4	18473.2	16620	15246	14541.7
Q6P7A9	Gaa	510.8	497.8	557.8	549.1	474.6	497
Q6P7B0	Wars1	7726.7	7942	7937.9	7933.8	7552.4	7210.7
Q6P7P5	Bzw1	4160.7	3878.5	3138.3	3214.8	3036.5	3181.8
Q6P7P8	Xrcc5	419.8	405	408.8	393.4	347.2	339.7
Q6P7Q4	Glo1	2213.1	2395	1714.6	1688.2	2085.4	2052.7
Q6P7R8	Hsd17b12	454.8	385	246.1	212.9	404.3	459.4
Q6P9U0	Serpinb6a	8792.2	9218.6	7974.3	8053.3	8653.4	8714.8
Q6P9U5	Rpl9	4618.4	5630.4	3435.2	3016	3552.8	3993.6
Q6P9U8	Eif3h	2638.8	3068	4618.3	4653.7	2928.1	2843.5
Q6P9U9	Impdh2	652.1	716	835.7	848.7	890	869
Q6P9V7	Psme1	3276.9	3628.4	3980.8	4807	5800.6	6490.1
Q6P9X1	Clns1a	43.1	40	43.9	44.9	32.2	39.7
Q6P9X4	Ptp4a2	833.4	842.6	801.6	802.6	887.5	897.6
Q6P9Y4	Slc25a4	1265.6	1142.8	1398.5	1474.7	1504.9	1546
Q6PAH0	Apoe	1966.7	2498.9	4064.2	4008.8	3533.7	3828.9
Q6PCT3	Tpd52l2	468.7	534.5	430.5	509.9	514.7	490.9
Q6PCT5	Pqbp1	332.9	378	561.5	467.6	353.9	365.5
Q6PCT9	Psmd6	2920.3	3131.2	2872.1	2777.3	3044.5	3216.8
Q6PCU8	Ndufv3	174.1	173.7	121.8	118.1	106.7	94.2
Q6PDU1	Srsf2	354.6	191.7	351.1	332.2	197.3	191.5
Q6PDU7	Atp5mg	611.7	702.1	621	921.6	907.5	934.8
Q6PDV6	LOC100911847	7235.6	5302.9	6240	6492.5	4401.5	4113.5
Q6PDV7	Rpl10	6473.6	8180.6	5990.9	5652.9	6914.4	7290.1

Q6PDV8	LOC100360057	1924.7	2150.7	2238.1	2293.4	2035.7	1870.7
Q6PDW1	Rps12	13510.3	10305.8	7401.4	6920.7	7269.2	7900.9
Q6PEC1	Tbca	4080.8	4151.6	2518.5	2349.6	3601.4	3815
Q6PST4	Atl1	215.1	261.6	494.9	574.7	320.4	310.9
Q6Q0N1	Cndp2	2605.8	2560.3	3051.8	3089.2	3141.9	3353.5
Q6Q7Y5	Gna13	119.9	117.9	493.7	587.1	137.2	114.4
Q6QI86	Hdhd2	802.2	1115	1119	1099	1117.9	1075.6
Q6RUV5	Rac1	1924.9	1701.4	1872.8	1908.9	1915.9	1866.9
Q6S3A0	Plec	13245.1	13665.5	15317	16614.6	20151	20417.6
Q6T4R6	Rt1da	91.7	93	82.6	109.1	165.5	145.6
Q6TMG5	Ikbkg	257	289.3	262.1	317.3	328.5	340.4
Q6TUD3	Rdh11	1274.4	1584.6	2098.5	2104.5	1400.7	1285.2
Q6TUD4	Yipf3	1398	1388.5	1466.7	1293.4	1161.2	1162.5
Q6TUE3	Tmem106b	150.6	160.9	233.2	237.2	191.4	178.3
Q6TUG0	Dnajb11	5029.2	4815.9	4162.3	4002.9	4287.1	4139.8
Q6TXG7	Shmt1	1531.1	1752.9	1101	1121.8	1739.4	1695.8
Q6TXG9	Sfr1	360.3	384.4	559.9	543.7	409.4	434.8
Q6U6G5	Zc3h15	2009.3	2292.4	1315.2	1202.2	2179.3	2100.8
Q6UPR8	Ermp1	220	249.4	539.3	608.5	267.3	214.7
Q6URK4	Hnrnpa3	339.6	412.4	609.3	507.9	550.8	484.2
Q6VEU8	Ddx24	893.2	1150.5	2046	2629	1815.8	1508.6
Q6VV72	Eif1a	2561.4	2691.3	1470.4	1675.3	2409.7	2487.1
Q6XDA0	Sptb	345.3	466.7	579.1	586.7	455	483.6
Q6XFR6	Gypc	67.6	100.8	92	91.6	93.7	97.3
Q6XLI7	Rbm12	261.4	248.5	156.1	194.2	249.2	269.8
Q704E8	Abcb7	245.3	257.5	377.2	334.6	242.8	218.3
Q712U5	Arpp19	358.8	283	580.7	529.4	217.7	204.4
Q71RR7	Guk1	331.5	370.8	806.4	678.5	352	339.4
Q71SA3	Thbs1	1064	1342.9	4279.1	3720.8	1852.7	1873.1
Q71TY3	Rps27	524.3	614.5	1169.9	855.2	621	569.6
Q71UE8	Nedd8	196.4	216.1	214.9	182.7	240.3	252.3
Q71UF4	Rbbp7	1124.2	1114	1157	1083.7	1473.6	1692.4
Q75N36	Dnajc7	731.1	1115.8	853.8	739.1	1172.9	1257.2
Q75Q41	Tomm22	107.3	121.3	67.3	105.2	151	142.8
Q75WE7	Vwa5a	101.9	123.7	197.1	199.2	182.7	179.4
Q765A7	Pgap1	58.9	65.8	81.9	83.5	65.4	59
Q76IC5	Pgpep1	138.3	129.7	154	167.4	186.1	181.6
Q78P75	Dynll2	3631.2	3776.9	1861.6	2109.5	3119.6	3115.7
Q792H5	Celf2	197.1	168.4	297.6	314.5	300.6	256.7
Q794E4	Hnrnpf	3568.9	3260.1	2843.9	2614.2	3032.4	3320.6
Q794F9	Slc3a2	10832.9	13497.6	12018.3	9852.4	7959.3	7441.4
Q7M0E3	Dstn	8622.5	7969.1	5491	5426.3	9431.5	8909.5
Q7M0F7		384.2	324.4	286.8	250.3	296.9	298.3
Q7M0F9		347.6	309.2	556.9	543.3	229.4	199.9
Q7TMA5	Apob	113.4	152.7	302.1	332.6	204.7	170.8
Q7TMB9	LOC299282	2698.9	2997.4	6812	5904.6	4101	4822.5

Q7TP07		383.9	346.2	264.8	237.6	298.4	307.5
Q7TP15	Mtap	245.2	377.5	395.5	405.2	410.4	397.7
Q7TP38		2771.6	2652	1831.6	1740.1	3276.5	3131.9
Q7TP42	Sec62	4834.6	4291.2	2022.9	2463.2	2886.1	2961
Q7TP48	Apmap	1802.6	1842	1889.7	1947.7	1659.7	1665.3
Q7TP52	Cmbl	2295.8	2382.9	1611.8	1590.2	1919.8	1811.7
Q7TP88	Steap4	484.5	562.7	507.9	645.1	602.2	598.1
Q7TP91	Surf4	380.6	414.4	488.7	467.6	367.8	316.1
Q7TPB1	Cct4	2376.2	1939.9	2800.2	2951.6	2185.6	2103.2
Q7TPI8	Yipf5	29.7	31.7	55.8	50.7	34.6	31.2
Q7TPJ5	Impad1	186.9	238.2	204.9	209.7	264.2	245.5
Q7TPK7		2397.7	2517.2	2177.6	2022.4	2329.4	2032.6
Q7TQ16	Uqcrq	225.8	194.7	345.4	325.2	213.2	188.5
Q7TQ70	Fga	13116.2	14997.5	47871.4	56420.1	23246.8	26906.7
Q7TQ90	Adh4	3295	3190.4	2460.8	2382.6	2600.4	2656.5
Q7TQ94	Nit1	256.9	217.4	76.9	111.9	222	223.1
Q7TQN4	Rela	537.6	609.5	1151.5	1163.2	769.4	722.2
Q7TSU1	Arfgef2	187.2	234.5	537.9	429.2	357.8	372.1
Q7TT49	Cdc42bpb	983.8	1044.7	1076.7	1387.6	1281.4	1214.4
Q80U96	Xpo1	1253	1169.1	1288.4	1414.3	1367.7	1219.6
Q80W89	Ndufa11	450.8	469.2	333	309.5	336.3	320.7
Q80W92	Vac14	79.8	98	207.8	252.1	134.1	130.7
Q80ZA3	Serpinf1	768.5	707.4	640.4	696	1050.5	1145.2
Q80ZF7	Rdh10	217.4	271.7	356.8	368.6	319.5	301.6
Q810U0	Ccdc50	409.4	338	799.1	796.6	390.4	431.1
Q811A3	Plod2	507.8	556.3	1071	1149.7	808.2	1000.3
Q811P6	Tfb1m	115.2	126.1	227.3	365.4	137.1	139.5
Q811S1		756.9	945	1643	1363.2	1088.5	1134.5
Q811S9	Gnl3	413.3	555.6	439.5	481.1	547.9	536.9
Q811X6	Cryl1	323.3	419.4	927.5	952.1	517.4	504.2
Q812C4	Eif4a1	556	271.1	308.1	296.6	212.8	203.5
Q812D3	Ppil3	29.2	35.6	102.3	92.6	37.1	40.6
Q8CF97	Vcpip1	169.5	188.6	275.1	276.9	226.7	215.8
Q8CFD0	Sfxn5	53.9	88.5	69.6	83.3	104	93.6
Q8CFN2	Cdc42	1915.4	2117	2054.5	1784.2	2772.4	2103.5
Q8CG45	Akr7a2	731.6	358.4	251.3	235.4	223	216.4
Q8CGV7	Thtpa	595.7	851.8	903	896.8	814.6	813
Q8CHN6	Sgpl1	1221.1	1240.2	2004.8	2154.2	1337.6	1338.3
Q8CJD3	Zg16	4495.1	3506.5	2238.8	2364.1	2019.7	2093
Q8CJH4	rGM2AP	1232.2	1562.7	1615	1703.7	2634.5	2361.9
Q8K1P7	Smarca4	471.1	532.1	955.6	981.3	793.5	756.8
Q8K1Q0	Nmt1	756	879.8	958.4	895.4	809.9	837.1
Q8K3K9	Gimap4	414.4	323.4	240.3	294	737.4	464.9
Q8K4M9	Osbpl1a	572.5	345.7	333.3	325.4	296	283.9
Q8K584		599.4	320.1	340.6	337.8	245.5	210.4
Q8K5A9	Nradd	200.1	225.5	137.1	163.6	221.1	200.9

Q8R2E7	Fadd	641.5	798.8	447.2	432.6	645	626
Q8R3Z7	Ehd4	2307.2	2096.6	2783.7	2710.5	2659.2	2468.4
Q8R424	Stambp	443.2	373.6	625.1	595	413.5	436.7
Q8R431	Mgll	675.4	497.8	425.7	691.2	469	448.3
Q8R478	Wbp2	122.6	134.1	137.1	146.1	141	138.7
Q8R4A1	Ero1a	528.1	691.1	887.4	826.2	638.5	638.9
Q8SEZ0	Mt-nd5	792.8	654.3	537.2	520.9	441.8	434.6
Q8VH46	Afap1	133.9	144.9	177.6	229.8	199.1	185.3
Q8VHF5	Cs	6326.6	7104.2	5352.3	4946.4	7036.4	6776.7
Q8VHI8	Bnip1	875.5	832.5	464.9	427.8	704.9	753
Q8VHQ7	Sytl4	67.9	76.1	147.6	178.1	114.4	110.3
Q8VHT6	As3mt	1602.8	1884.4	1310.4	1306.7	1717.5	1645.4
Q8VHU4	Elp1	745.4	807.4	1035.5	896.2	944	969.8
Q8VHV8	Selenos	1419.4	1294.5	2030.7	2133.3	1213.8	1236.6
Q8VIB1	G22p1	411.1	416	430.3	444.7	440.3	408.3
Q8VID1	Dhrs4	1299.2	1502.1	1456.6	1259.2	1147.7	1109.9
Q91V33	Khdrbs1	775	998.9	976	1109.1	1186.6	1245.1
Q91VC0	Dctpp1	122.6	125.8	82.6	68.2	83.2	85.6
Q91W30	Akr1b8	911.3	1012	2103.9	1804.9	2191.1	2069.8
Q91Y78	Uchl3	1470	1566.9	2207.6	2179.5	1677.5	1703.9
Q91Y81	Septin2	2736.8	2738.8	2593.3	2871.7	3348.2	3408
Q91ZN1	Coro1a	1839.1	1594.7	1777.8	1997.4	2711.9	2811.4
Q91ZW6	Tmlhe	712.4	663.1	511.6	579.1	546.5	587.6
Q920A6	Scpep1	3552.2	3666.6	4187.8	3670.9	4020.1	3914.3
Q920D2	Dhfr	83.3	98.7	99.8	114.9	101.6	96.1
Q920F5	Mlycd	856.4	939.7	1163.2	1400.4	762.8	717.9
Q920L2	Sdha	8425.2	7923.8	6857.7	7002.4	6305.2	6177.8
Q920P6	Ada	976	1205.1	1617.1	1423.6	1606	1559.5
Q920Q0	Palm	182.9	163.6	204.4	204.2	219.4	199.1
Q923K9	A1cf	538.7	557.3	407.9	475.7	492.5	415.8
Q923M1	Msra	1856.2	2033.1	905.5	929.4	1380.3	1423.1
Q923V4	Fbxo6	289.8	344.7	177.6	154.5	475.8	450.2
Q923V8	Selenof	1934.3	1705	1568.8	1213.9	1773.5	1889.8
Q923W4	Hdgfl3	579.8	716.3	943.3	640.5	933.4	789.6
Q923Z2	Tpm1	2823.8	2658.8	2885.1	2821.8	3278.8	3273.9
Q924M6		4504	4614.4	3694	3489.8	3934.7	3904.9
Q924S5	Lonp1	6939	7850.7	8662.5	12027.9	6910.5	6815.1
Q925G1	Hdgfl2	298.3	322.5	381.8	350.9	489.6	473.5
Q925Q9	Sh3kbp1	560.2	597.5	1245.6	1351.3	762.5	873.6
Q99068	Lrpap1	725	864.6	634.7	625.6	917	1079.6
Q99J82	Ilk	1682.4	2067	2562.2	2515.2	2737.5	2665.6
Q99M63	Smu1	535.9	652.4	893.1	792.8	824.3	725.1
Q99MI5	LOC100912604	1392.7	1147.9	977.1	973.5	1023.1	1018.6
Q99MI7	Uba3	771.6	897.7	917.6	1023.4	1193.3	1223.1
Q99MI8		73.3	65	100.7	127	75.2	60.7
Q99ML5	Pcyox1	1150	1170.4	1332.4	1525.8	1305.7	1293.8

Q99MY2	Nudt4	513.8	581.7	678.2	617.9	698.3	620
Q99MZ8	Lasp1	4498.6	3668.7	4884.5	4067.1	4396.4	4427.1
Q99N27	Snx1	671.4	548.3	855.8	958.4	678.5	645.3
Q99NA5	Idh3a	1486.6	1318.8	1141.2	1008.1	1147	1385
Q99ND9	Rwdd1	334.9	455	229.1	330.4	505.4	444.2
Q99P74	Rab27b	419.5	466.1	253.6	243.9	380.9	336.2
Q99P75	Rab9a	141.9	186.3	129.6	138.2	210.6	206.4
Q99PD6	Tgfb1i1	149.4	138.9	69.6	139.8	262.2	268.4
Q99PJ4		163	158.8	121.8	143.1	230.2	227.5
Q99PV2	Stxbp3	400.6	483.8	1000.5	957.6	686.8	566
Q9EPF2	Mcam	429.1	486.8	647.3	655.8	568.7	505.4
Q9EPH8	Pabpc1	3365.1	3784.9	3608	3727.3	3770	3994.4
Q9EPT7		974.4	1102.3	2703.4	2916.6	1779.1	1830.8
Q9EPV3	Emap2	95	114.6	904.1	723.8	143	125.5
Q9EQ76	Fmo3	101.2	117.6	98	105.8	117.5	120.6
Q9EQH5	Ctbp2	242.1	306.3	307.4	277.5	380.9	387.1
Q9EQP5	Prelp	946	700.3	964.8	851.9	866.9	817.4
Q9EQS0	Taldo1	9689.4	9710.4	10596.4	11049.4	9363.5	9772
Q9EQS4	LOC103691744	9836	8559.5	9079.1	10329.9	7352.4	6725.3
Q9EQT5	Tinagl1	638.1	687.8	364.8	399.4	1053.5	851.6
Q9EQX9	Ube2n	2715.1	2958.2	2302.4	2266.7	3068.6	3012.2
Q9ER24	Atxn10	92.7	102.8	103.2	129.2	131.3	109.7
Q9ER34	Aco2	13835.7	15360.5	13515	13639.4	14558.8	13596.5
Q9ERA7	Msln	296.4	275.9	452.5	419.7	428.9	400.5
Q9ERH3	Wdr7	183.7	185.6	82.4	65.2	167.9	168.7
Q9ERR2	Commd5	139.5	144.9	184.3	174.9	164.4	154.8
Q9ES21	Sacm1l	4736.9	4362.9	4782.4	4698.9	3875.8	3741
Q9ES32	Tfa	1966.1	2389.7	2477.7	2361.2	2166.7	2256.3
Q9ES53	Ufd1	324.7	443.4	1014	950.7	584.5	561.8
Q9ESH1	Psmd4	665.2	591.6	672	735.2	626.7	589
Q9ESN0	Niban1	9490.1	8827.3	7313.2	7643.9	8226.9	8053.7
Q9ESS6	Bcam	418.7	502.2	781.9	1019.7	589.3	574
Q9EST6	Anp32b	1814.9	1898.2	1999.1	2238.1	2472.8	2364.5
Q9JHB5	Tsnax	282.3	360	255.9	263.6	329.7	328.1
Q9JHE5	Slc38a2	211.1	233.8	224.1	188.1	177.6	193.2
Q9JHL4	Dbnl	719.5	1109.1	1718.5	1366.4	1159.6	1032.4
Q9JHU5	Arfip1	620.7	699.7	700.6	758.4	806.1	780.4
Q9JHW0	Psmb7	490.2	738.9	1435.8	1274.7	803.3	713.1
Q9JHW5	Vamp7	133	137.6	131.2	110.1	196.3	158.6
Q9JHX4	Casp8	273.5	290	483.2	463.8	357	344.3
Q9JHZ9	Slc38a3	414.9	372.2	603.4	692.8	314.5	281.8
Q9JI03	Col5a1	164.6	190.4	151.5	155.9	276.1	184.8
Q9JI56	Snap29	558.9	573.3	543.8	458.4	693.8	674.1
Q9JI85	Nucb2	6128.1	4847.3	3641.2	3626.9	4156.1	3691.7
Q9JIL8	Rad50	488.5	549.7	861.8	962.4	637.1	634.7
Q9JIL9	Nbn	94.1	103.1	111.7	132.4	126.7	127.1

Q9JIM0	Mre11	150.9	178.1	355.2	329.8	249.6	219.1
Q9JIY6	Cml6	2721	2103.3	1838.9	1683.1	1216	1223.6
Q9JJ19	Slc9a3r1	4181.3	4192.7	4869.4	4530.1	6088.9	5950.5
Q9JJ31	Cul5	805.6	933	998.4	979.7	1140.1	1068.2
Q9JJ54	Hnrnpd	4861.3	5521	5665	5367.2	7545.1	7508.9
Q9JJP9	Ubqln1	340.2	322.9	364.8	478.5	441.9	471.8
Q9JJW3	Atp5mk	590.6	618.5	381.6	393.4	382.9	403.4
Q9JK11	Rtn4	857.7	889.7	1246.8	1221.8	1369.2	1290.4
Q9JK25	Clip1	770.6	988.6	1176	1072.1	944.1	905.1
Q9JK93		248.9	303.6	271	223.9	397.9	319.7
Q9JKL3	Bax	263.3	320.8	529.2	493.6	442.7	405.9
Q9JLA3	Uggt1	5918.5	6459.5	5578	5847.4	5421.1	5702
Q9JLH7	Cdk5rap3	2754.1	3357.7	2281.3	2331.5	3095.3	3056.8
Q9JLZ1	Glrx3	641.3	756.7	586.6	468.8	836.2	752.9
Q9JMB5	Adrm1	159.1	185.2	233.7	193.6	338.8	250.9
Q9JMJ4	Prpf19	3031.1	2968	4175.6	4213.2	3228	3168.3
Q9QVC8	Fkbp4	941.8	977.5	614.8	578.5	980.3	1000.9
Q9QX67	Dap	76.1	86.1	63.6	60.8	71.4	56
Q9QX69	Lancl1	280.2	386.7	532.9	589.6	360.3	360.2
Q9QX79	Fetub	3296	3218.8	3731.3	3627.9	5435.5	4941.1
Q9QY17	Pacsin2	3840	4852.6	6334.7	6196.2	4583.8	4474.6
Q9QY87	NFIC	313.3	424.1	296.6	217.1	488	449.3
Q9QYL8	Lypla2	2024.1	1983.3	1593.1	1607.3	1683.6	1710.7
Q9QYU2	Tsfm	173.2	151.4	92	117.7	143.6	128.2
Q9QYW0	Aatf	234.4	233.3	376.8	355.3	357.9	308
Q9QYW3	Mob4	388.4	522.6	459.4	461.8	431.6	425.6
Q9QZ86	Nop58	2801.7	3334	5657	5149.2	3995.5	3742.6
Q9QZK8	Dnase2	621.4	712.4	713.2	648.5	642.4	624.3
Q9QZR6	Septin9	778.9	655.7	496.7	493	815.1	975
Q9QZT0	Cuzd1	411.5	448.2	402.2	407.5	278.6	258.6
Q9R037	Wdr44	381.7	403.8	361.4	342.3	409.8	454.7
Q9R063	Prdx5	4335.6	4260.9	5162.8	5338.8	5175.7	5265.4
Q9R0T3	Dnajc3	7077.9	5739.6	5098.7	5151.5	4680.2	4493.3
Q9R0T4	Cdh1	1226.5	1305.3	1282.9	1230.4	1439.4	1381.2
Q9R1J8	P3h1	188.8	229.8	400.1	362.2	297.9	314.4
Q9R1T1	Banf1	413.5	644.8	958.6	836.8	861.4	1014.9
Q9TPL8	RT1.Bbeta	835.5	388.1	419.1	494	794.5	429.2
Q9WTR7	Sec11c	3794.1	4184.5	5358.7	5357.5	3255.4	3440.7
Q9WTT6	Gda	6561	7769.4	7401.4	6969.2	10844.9	11068.2
Q9WTT7	Bzw2	628.2	533.1	549.1	489.8	352.1	351.2
Q9WTV0	Preb	852.7	912.5	791.3	861.2	966	951.2
Q9WU49	Carhsp1	3292	2542.5	1863.2	1770.5	2540.8	2406.7
Q9WU82	Ctnnb1	1761.6	1602.7	1242.4	1075.7	1641.1	1633.4
Q9WUC4	Atox1	192.9	257.3	113.1	130.8	220.3	284
Q9WUC8	Plrg1	647.8	658.7	832.2	694	633.4	611.7
Q9WUF4	Vamp8	805	680.4	488.7	467	645	629.7

Q9WUH9	Fbn2	542.4	608.4	1566.7	1088.2	875	924.9
Q9WV97	Timm9	2919.1	2631.6	2804.8	2869.1	2101.5	2107.7
Q9WVA1	Timm8a	1701.9	1614.3	1117.2	1225.6	1330	1410.4
Q9WVH8	Fbln5	191.2	121	71.4	82.3	119.3	126.8
Q9WVJ6	Tgm2	4618.4	4713.5	5669.1	5738.4	7396.2	7230.4
Q9WVK7	Hadh	1240.6	1088.1	1189.5	1221	983.3	873.2
Q9WVR6	Slc7a8	1689.2	2145.6	2636.6	2397.3	2120.3	1874
Q9WVR7	Ppm1f	1079.6	792.6	440.4	523	822.5	1030.3
Q9WVS2	Osgep	601.3	493.9	777.1	801.6	486.9	517.2
Q9Z0J5	Txnrd2	347.6	406.2	403.3	356.8	391.1	316.7
Q9Z0U8	pRM10	2914.3	3308.7	5474.3	5816.1	4557.5	4502.7
Q9Z0V5	Prdx4	5852.6	4332.5	3925.4	3384.8	3049.1	2870.9
Q9Z144	Lgals2	2488.6	2611.7	1764.3	1943.3	2182.5	2062.7
Q9Z1B2	Gstm5	281.2	282.9	153.8	183.3	260.8	260.1
Q9Z1E1	Flot1	157	192.3	246.1	251.1	317.4	270.1
Q9Z1N1	Fbp2	2119.7	2277.1	1968	2267.5	1683.8	1588.1
Q9Z1P2	Actn1	15273.7	13509.3	15003.2	14272.4	16109	15788.5
Q9Z1W6	Mtdh	1549.3	1548.1	1833.4	1705.5	1309.8	1336.6
Q9Z254	Gipc1	664.1	831.2	1297.6	1107.5	1041.9	1012.5
Q9Z269	Vapb	2130.9	2517.5	2380.2	2437.1	2610.9	2562.7
Q9Z270	Vapa	6937.4	6402.5	4332.9	4732.9	5425.1	5477.3
Q9Z2L0	Vdac1	13853.4	14775.2	9390.2	9011.3	12613.5	12878.4
Q9Z2P5	Ripk3	71.3	82.5	117.2	112.9	167.7	155.8
Q9Z2Q1	Sec31a	8580.2	9136.6	9959.2	8644.3	8986.7	8656.3
Q9Z2S9	Flot2	318.1	380	522.6	505	489.9	485.4
Q9Z2X5	Homer3	92	216.3	264.8	215.7	283.1	271.7
Q9Z2Z8	Dhcr7	517.7	603.4	727.9	626.8	757.8	623.7
Q9Z339	Gsto1	3112.2	3394.2	2162.1	2059.4	2665.3	2864.7
R9PXR4	Tomm70	1757.4	1673.8	1512	1602.3	1805.5	1738.1
R9PXU4	Txnrd1	1394.6	1486.5	2063.4	1807.7	2019.4	1882
V5QSV9		462.8	391.8	331.2	396	1368.9	1384.4
V9H0R3	CCKAR	1060.2	865.2	836.4	803.2	520.5	499
W8BZ34	DDX3Y	429.7	460.3	358.4	347.5	324.7	384.8
X1WI37	Rps4x	14050.1	13162.8	12425.7	11544.2	15170.4	12163.7
Z4YNF4	Acp1	680	624.2	543.2	463.6	668.8	748.8
F1M754	Map4k4	5.8	8.5	0	6	16.6	11.7
Q5U206	Calml3	5	4.3	7.3	0	5	4.9
