# Supplementary material for: Integrative proteomics, phosphoproteomics and acetylation proteomics analyses of acute pancreatitis in rats
Source: Int J Med Sci. 2023 May 11;20(7):888–900. doi: 10.7150/ijms.81658 (PMC10266050; doi:10.7150/ijms.81658)
Supplement: Supplementary file 3 — Supplementary table 2. [file ijmsv20p0888s3.pdf]

Protein	cluster	Description
G3V6P7	6	Myosin, heavy polypeptide 9, non-muscle
F1M853	2	Ribosome-binding protein 1
A0A0G2JUA5	1	AHNAK nucleoprotein
A0A0G2K6S9	6	Myosin-11
E9PTU4	4	Myosin-11
Q3KRF2	3	High density lipoprotein binding protein (Vigilin)
M0R9X8	5	Cytoplasmic dynein 1 heavy chain 1
G3V9M6	6	Fibrillin 1
C0JPT7	6	Filamin A
P63018	4	Heat shock cognate 71 kDa protein
P04785	2	Protein disulfide-isomerase
A0A0G2QC06	2	Signal recognition particle receptor subunit beta
Q10758	6	Keratin, type II cytoskeletal 8
P05197	3	Elongation factor 2
G3V844	2	Alpha-amylase
P63259	6	Actin, cytoplasmic 2
Q6S3A0	6	Plectin
G3V7Q7	1	IQ motif containing GTPase activating protein 1 (Predicted), isoform CRA_b
D4AA52	5	Murinoglobulin-1
P27657	2	Pancreatic triacylglycerol lipase
P12346	4	Serotransferrin
G3V8L3	4	Lamin A, isoform CRA_b
F1MA56	2	Chymotrypsinogen B
P07882	2	Bile salt-activated lipase
Q66X93	3	Staphylococcal nuclease domain-containing protein 1
G3V852	1	RCG55135, isoform CRA_b
A0A0G2JXT8	6	Filamin B
A0A0G2K013	1	Alpha-actinin-4
D3ZAM3	2	Carboxypeptidase B
M0RBF1	5	C3-beta-c
G3V6S0	4	Spectrin beta chain
P00731	3	Carboxypeptidase A1
P34058	4	Heat shock protein HSP 90-beta
P19223	2	Carboxypeptidase B
F1LP05	5	ATP synthase subunit alpha
P10719	3	ATP synthase subunit beta, mitochondrial
P63269	6	Actin, gamma-enteric smooth muscle
P63039	2	60 kDa heat shock protein, mitochondrial
G3V6T7	2	Protein disulfide-isomerase A4
P28037	2	Cytosolic 10-formyltetrahydrofolate dehydrogenase
M0R757	2	Elongation factor 1-alpha
Q63041	5	Alpha-1-macroglobulin
P54316	2	Inactive pancreatic lipase-related protein 1
P04797	6	Glyceraldehyde-3-phosphate dehydrogenase
A0A0G2K0D8	5	10-formyltetrahydrofolate dehydrogenase

Q03626	5	Murinoglobulin-1
D3ZTP0	2	10-formyltetrahydrofolate dehydrogenase
P06685	3	Sodium/potassium-transporting ATPase subunit alpha-1
G3V826	4	Transketolase
Q64571	4	Carboxylic ester hydrolase (Fragment)
Q6P3V9	4	60S ribosomal protein L4
A0A068F1Y2	5	Beta-actin (Fragment)
Q5XIF6	4	Tubulin alpha-4A chain
P10860	2	Glutamate dehydrogenase 1, mitochondrial
F1LRI5	5	GCN1 activator of EIF2AK4
P16617	4	Phosphoglycerate kinase 1
P62260	4	14-3-3 protein epsilon
Q6P136	2	Hyou1 protein
Q5U328	4	Nucleolin
G3V976	2	Carboxypeptidase A2
M0R660	6	Glyceraldehyde-3-phosphate dehydrogenase
Q7TQ70	5	Fibrinogen alpha chain
P17764	2	Acetyl-CoA acetyltransferase, mitochondrial
P04636	3	Malate dehydrogenase, mitochondrial
A0A096P6L8	1	Fibronectin
Q5M7X1	2	Coatomer subunit beta
P24368	2	Peptidyl-prolyl cis-trans isomerase B
Q5BJZ3	2	Proton-translocating NAD(P)(+) transhydrogenase
P51647	2	Retinal dehydrogenase 1
Q1JU68	3	Eukaryotic translation initiation factor 3 subunit A
P11980	4	Pyruvate kinase PKM
B1H216	4	Globin c3
Q6IE52	5	Murinoglobulin-2
P63245	4	Receptor of activated protein C kinase 1
M0R5J4	1	2-phospho-D-glycerate hydro-lyase
A0A0G2JSI1	4	4-trimethylaminobutyraldehyde dehydrogenase
Q5U302	1	Catenin (Cadherin associated protein), alpha 1
P07150	6	Annexin A1
A0A0G2K401	2	Propanoyl-CoA:carbon dioxide ligase subunit alpha
P21531	2	60S ribosomal protein L3
P08426	2	Cationic trypsin-3
Q63610	6	Tropomyosin alpha-3 chain
G3V836	6	Clusterin
P06866	6	Haptoglobin
P11884	3	Aldehyde dehydrogenase, mitochondrial
G3V7J0	2	Aldehyde dehydrogenase family 6, subfamily A1, isoform CRA_b
P09495	6	Tropomyosin alpha-4 chain
P19945	2	60S acidic ribosomal protein P0
Q5FVG5	4	Similar to tropomyosin 1, embryonic fibroblast-rat, isoform CRA_c
Q6IMY8	4	Heterogeneous nuclear ribonucleoprotein U
P09895	5	60S ribosomal protein L5

Q6P6R2	2	Dihydrolipoyl dehydrogenase, mitochondrial
P35565	2	Calnexin
P50399	4	Rab GDP dissociation inhibitor beta
Q5RJR9	6	Collagen-binding protein
G3V9N0	2	Polyadenylate-binding protein
P10111	4	Peptidyl-prolyl cis-trans isomerase A
Q62667	6	Major vault protein
P20059	1	Hemopexin
D3ZAF5	5	Periostin
Q5XI38	6	Lymphocyte cytosolic protein 1
Q66H80	3	Coatomer subunit delta
Q6AYD3	2	Proliferation-associated protein 2G4
A0A097BW25	6	Periostin
B2RYG2	2	Phosphoenolpyruvate carboxykinase (GTP) (Fragment)
Q5RKI0	6	WD repeat-containing protein 1
D3ZU22	6	60S ribosomal protein L7a
A0A0G2K3Z9	4	Peroxiredoxin-1
P56574	2	Isocitrate dehydrogenase [NADP], mitochondrial
P14480	5	Fibrinogen beta chain
P10758	6	Lithostathine
A0A0G2JSQ4	4	Tropomyosin 1, alpha, isoform CRA_p
P69897	4	Tubulin beta-5 chain
A0A0G2JSZ5	3	Protein disulfide-isomerase A6
Q6P790	3	60S ribosomal protein L6 (Fragment)
Q68FS4	2	Cytosol aminopeptidase
P31977	4	Ezrin
P05065	6	Fructose-bisphosphate aldolase A
P05545	6	Serine protease inhibitor A3K
P68255	4	14-3-3 protein theta
G3V7C6	4	Tubulin beta chain
Q6P502	6	T-complex protein 1 subunit gamma
A0A0G2JTL5	3	Pyruvate carboxylase
A0A0G2K1F3	3	Coatomer subunit gamma
A0A096MK30	6	Moesin
Q07936	6	Annexin A2
M0R735	2	Heterogeneous nuclear ribonucleoprotein Q
P02680	1	Fibrinogen gamma chain
P62909	3	40S ribosomal protein S3
P62907	2	60S ribosomal protein L10a
Q7TMB9	5	Ab1-021
Q68FY4	1	Gc-globulin
D3ZFG3	2	Chymotrypsin-like elastase 3B
P45592	6	Cofilin-1
Q68FR6	2	Elongation factor 1-gamma
O35854	3	Branched-chain-amino-acid aminotransferase, mitochondrial
P63102	4	14-3-3 protein zeta/delta

A0A0G2K1C0	6	Actin-like protein 3
P04639	5	Apolipoprotein A-I
F1LP30	3	Methylcrotonoyl-CoA carboxylase subunit alpha, mitochondrial
P00762	3	Anionic trypsin-1
Q68FZ8	3	Propionyl coenzyme A carboxylase, beta polypeptide
Q5D059	1	Heterogeneous nuclear ribonucleoprotein K
Q6P6T6	6	Cathepsin D
P36953	5	Afamin
A0A096MIX2	5	RNA helicase
F1LQ48	4	Heterogeneous nuclear ribonucleoprotein L
P49242	3	40S ribosomal protein S3a
P62755	5	40S ribosomal protein S6
Q63011	1	Zero beta-globin (Fragment)
D3ZSA9	3	Nodal modulator 1
F1LNF0	1	Myosin heavy chain 14
Q9ESN0	2	Protein Niban 1
Q499V7	3	Succinate-CoA ligase subunit beta (Fragment)
E9PSQ1	2	Alpha-amylase
Q01177	1	Plasminogen
P13635	1	Ceruloplasmin
P85834	2	Elongation factor Tu, mitochondrial
P02454	1	Collagen alpha-1(I) chain
G3V9K0	3	Cysteinyl-tRNA synthetase
Q62902	3	Protein ERGIC-53
F1M614	5	Laminin subunit alpha 2
A0A0G2JWK7	6	Transgelin
D3ZUL3	4	Collagen type VI alpha 1 chain
P50475	2	Alanine--tRNA ligase, cytoplasmic
Q7M0E3	4	Destrin
P07943	4	Aldo-keto reductase family 1 member B1
A0A0G2JVH4	2	MICOS complex subunit MIC60
P05426	2	60S ribosomal protein L7
P62243	3	40S ribosomal protein S8
A2VCW9	2	Alpha-aminoadipic semialdehyde synthase, mitochondrial
P47942	1	Dihydropyrimidinase-related protein 2
P85108	4	Tubulin beta-2A chain
P49088	2	Asparagine synthetase [glutamine-hydrolyzing]
Q4G075	2	Leukocyte elastase inhibitor A
P61983	4	14-3-3 protein gamma
Q9Z2L0	2	Voltage-dependent anion-selective channel protein 1
P13601	5	Aldehyde dehydrogenase, cytosolic 1
A0A140TAF0	6	RCG62531, isoform CRA_g
P02651	1	Apolipoprotein A-IV
A0A0G2K9V6	3	Threonyl-tRNA synthetase
P14141	2	Carbonic anhydrase 3
Q5M875	2	17-beta-hydroxysteroid dehydrogenase 13

W8BZ34	2	RNA helicase
A0A0G2K6U1	5	Vesicle-fusing ATPase
D3ZCT7	3	Protein transport protein SEC23
P12788	6	Trypsin-4
A0A0G2K719	2	RNA helicase
Q5PPJ6	2	Leucyl-tRNA synthetase
F1LPV0	4	Asparagine--tRNA ligase
A0A0G2K2Q2	2	Glycine C-acetyltransferase
F1LT35	2	Similar to 60S ribosomal protein L23a
P54318	2	Pancreatic lipase-related protein 2
Q4G061	2	Eukaryotic translation initiation factor 3 subunit B
Q924M6	2	Apoptosis-inducing factor
P25113	4	Phosphoglycerate mutase 1
Q66HA8	1	Heat shock protein 105 kDa
P19804	5	Nucleoside diphosphate kinase B
P35213	4	14-3-3 protein beta/alpha
Q9WTT6	6	Guanine deaminase
Q6TUG0	2	DnaJ homolog subfamily B member 11
A0A0G2KAP1	2	Endoplasmic reticulum oxidoreductase 1 beta
D3ZD23	2	ATP-binding cassette subfamily E member 1
Q5EBC0	1	Inter alpha-trypsin inhibitor, heavy chain 4
Q920L2	2	Succinate dehydrogenase [ubiquinone] flavoprotein subunit, mitochondrial
Q3KRC3	3	SRP receptor subunit alpha
G3V8G5	6	Golgi apparatus protein 1
P25235	2	Dolichyl-diphosphooligosaccharide--protein glycosyltransferase subunit 2
P41562	4	Isocitrate dehydrogenase [NADP] cytoplasmic
P85125	4	Caveolae-associated protein 1
Q68FU3	3	Electron transfer flavoprotein subunit beta
P30349	4	Leukotriene A-4 hydrolase
Q5PQK5	2	Radixin
P63029	6	Translationally-controlled tumor protein
P23358	2	60S ribosomal protein L12
M0RAQ6	6	Hexokinase
D4A817	5	Histone H2B
Q9WVJ6	6	Protein-glutamine gamma-glutamyltransferase 2
G3V712	1	Keratin complex 2, basic, gene 7, isoform CRA_a
P04642	6	L-lactate dehydrogenase A chain
G3V9N8	5	AP complex subunit beta
E9PTV9	5	Glyceraldehyde-3-phosphate dehydrogenase
B4F758	1	High mobility group protein 1
Q5U2Q7	4	Eukaryotic peptide chain release factor subunit 1
P07314	3	Glutathione hydrolase 1 proenzyme
B6DYQ7	2	GST class-pi
P61314	2	60S ribosomal protein L15
P0C5H9	3	Mesencephalic astrocyte-derived neurotrophic factor
Q4KM74	2	Vesicle-trafficking protein SEC22b

Q9QY17	5	Protein kinase C and casein kinase substrate in neurons 2 protein
Q6P6V0	1	Glucose-6-phosphate isomerase
F1MA98	1	Nucleoprotein TPR
P61621	4	Protein transport protein Sec61 subunit alpha isoform 1
D4ACV3	5	Histone H2A
F1LW74	5	IQ motif-containing GTPase-activating protein 2
Q08163	6	Adenylyl cyclase-associated protein 1
Q6P685	2	Eukaryotic translation initiation factor 2 subunit beta
O88941	3	Mannosyl-oligosaccharide glucosidase
P08932	6	T-kininogen 2
P81795	5	Eukaryotic translation initiation factor 2 subunit 3, X-linked
P14668	1	Annexin A5
P00774	2	Chymotrypsin-like elastase family member 2A
B1WC34	2	Glucosidase 2 subunit beta
P62282	2	40S ribosomal protein S11
P62963	6	Profilin-1
G3V7L6	5	26S proteasome AAA-ATPase subunit RPT1
D4A9D8	2	Oxysterol-binding protein
Q9R063	1	Peroxiredoxin-5, mitochondrial
P14408	3	Fumarate hydratase, mitochondrial
B5DF80	3	Polyadenylate-binding protein
Q4QQV4	5	Histidine--tRNA ligase, cytoplasmic
Q6AYU2	4	Pcbp2 protein
Q6P2A5	3	GTP:AMP phosphotransferase AK3, mitochondrial
Q9JJ54	6	Heterogeneous nuclear ribonucleoprotein D0
P97852	6	Peroxisomal multifunctional enzyme type 2
Q5RK10	3	60S ribosomal protein L13a
Q3ZB97	4	AP complex subunit beta
Q5BKA1	4	Methionine aminopeptidase 2
B0BMS8	6	Myl9 protein
P14669	1	Annexin A3
Q5U362	4	Annexin
A0A0G2JZ73	6	Alpha-1-antiproteinase
Q6MG08	5	ATP-binding cassette sub-family F member 1
D3ZZT9	1	Collagen type XIV alpha 1 chain
D4A1J4	5	3-hydroxybutyrate dehydrogenase type 2
P05712	2	Ras-related protein Rab-2A
B2GV33	5	Amine oxidase (Fragment)
D3ZMS1	5	Splicing factor 3b, subunit 2
Q5XFX0	6	Transgelin-2
Q6PDV6	3	40S ribosomal protein S14
P62919	3	60S ribosomal protein L8
F1LNH3	4	Collagen type VI alpha 2 chain
P63326	3	40S ribosomal protein S10
D3ZU13	2	Eukaryotic translation initiation factor 4 gamma, 1
P97675	1	Ectonucleotide pyrophosphatase/phosphodiesterase family member 3

P12007	2	Isovaleryl-CoA dehydrogenase, mitochondrial
Q6LDS4	5	Superoxide dismutase [Cu-Zn]
A0A0G2K654	6	H1.2 linker histone, cluster member
Q6MG79	1	C4a anaphylatoxin
P81155	2	Voltage-dependent anion-selective channel protein 2
Q5RK09	2	Eukaryotic translation initiation factor 3 subunit G
Q5XIM9	5	T-complex protein 1 subunit beta
Q4FZT9	5	26S proteasome non-ATPase regulatory subunit 2
O55096	1	Dipeptidyl peptidase 3
B5DEN5	2	Elongation factor 1-beta
P54313	1	Guanine nucleotide-binding protein G(I)/G(S)/G(T) subunit beta-2
P24155	5	Thimet oligopeptidase
P31044	4	Phosphatidylethanolamine-binding protein 1
A0A0G2QC11	6	Keratin 86
P32551	2	Cytochrome b-c1 complex subunit 2, mitochondrial
Q64380	3	Sarcosine dehydrogenase, mitochondrial
Q6AY09	5	Heterogeneous nuclear ribonucleoprotein H2
D4A8U7	5	Dynactin subunit 1
Q63584	3	Transmembrane emp24 domain-containing protein 10
Q9ES21	3	Phosphatidylinositol-3-phosphatase SAC1
F1M983	1	Complement factor H
G3V6H2	5	Pre-mRNA processing factor 8, isoform CRA_a
P70584	2	Short/branched chain specific acyl-CoA dehydrogenase, mitochondrial
A1A5L2	6	Pgm1 protein (Fragment)
Q6PDV7	4	60S ribosomal protein L10
P09527	4	Ras-related protein Rab-7a
G3V6L9	2	Peptidylprolyl isomerase
P26772	5	10 kDa heat shock protein, mitochondrial
A0A0G2JVG4	3	Peroxisomal trans-2-enoyl-CoA reductase
Q9Z270	2	Vesicle-associated membrane protein-associated protein A
Q8VHF5	4	Citrate synthase, mitochondrial
A0A0G2K051	1	Early endosome antigen 1
B5DES0	1	Small nuclear ribonucleoprotein Sm D2
Q6AY58	5	B-cell receptor-associated protein
F1M062	2	La ribonucleoprotein 1, translational regulator
Q63581	6	Uncharacterized protein
A7VJC2	6	Heterogeneous nuclear ribonucleoproteins A2/B1
P97532	3	3-mercaptopyruvate sulfurtransferase
F7EL36	6	Acidic leucine-rich nuclear phosphoprotein 32 family member A
D4ABT8	1	Heterogeneous nuclear ribonucleoprotein U-like 2
P62828	4	GTP-binding nuclear protein Ran
O08651	3	D-3-phosphoglycerate dehydrogenase
Q66HM2	6	AP-2 complex subunit alpha
Q5XI78	1	2-oxoglutarate dehydrogenase, mitochondrial
A0A0G2JTV2	6	Non-muscle caldesmon
A0A0A0MY25	3	Ribonuclease pancreatic beta-type

P62278	4	40S ribosomal protein S13
Q32PZ7	2	Signal recognition particle subunit SRP72 (Fragment)
D3ZFA8	4	40S ribosomal protein S17
P24049	3	60S ribosomal protein L17
Q6AYK8	4	Eukaryotic translation initiation factor 3 subunit D
P97690	1	Structural maintenance of chromosomes protein 3
O35987	4	NSFL1 cofactor p47
Q7TP38	4	Amine oxidase
P80067	2	Dipeptidyl peptidase 1
Q5XI73	6	Rho GDP-dissociation inhibitor 1
Q68FP1	6	Gelsolin
F1LRS8	5	CD2-associated protein
F1M6V1	4	Heterochromatin protein 1-binding protein 3
Q9QZ86	5	Nucleolar protein 58
P51635	4	Aldo-keto reductase family 1 member A1
P0DMW0	6	Heat shock 70 kDa protein 1A
B5DEP6	5	PCI domain-containing protein (Fragment)
Q68FR9	5	Elongation factor 1-delta
Q5M7V3	5	LOC367586 protein
Q9Z0U8	1	Nucleic acid binding factor pRM10
G3V7V5	2	Peptidylprolyl isomerase
B5DFA0	3	Villin-1
P54921	2	Alpha-soluble NSF attachment protein
A0A0G2JSV2	4	Carbonyl reductase [NADPH] 1-like
Q5XIH3	3	NADH dehydrogenase [ubiquinone] flavoprotein 1, mitochondrial
A0A0G2K8P3	4	E3 ubiquitin-protein ligase
D4A9L9	2	Endoplasmic reticulum protein 27
D4A8A0	4	Aspartate carbamoyltransferase
B2RYQ8	2	Large subunit ribosomal protein L36a, isoform CRA_a
P62718	2	60S ribosomal protein L18a
Q3ZAV2	4	Y-box-binding protein 1
P18420	5	Proteasome subunit alpha type-1
P11762	4	Galectin-1
G3V6H0	5	RCG48149, isoform CRA_b
A0A0H2UI38	2	40S ribosomal protein S19
Q62636	4	Ras-related protein Rap-1b
Q9QX79	6	Fetuin-B
F1M9X2	3	Pancreatic secretory granule membrane major glycoprotein GP2
P19112	5	Fructose-1,6-bisphosphatase 1
Q7TQ90	2	Alcohol dehydrogenase 5
Q9JJ19	6	Na(+)/H(+) exchange regulatory cofactor NHE-RF1
Q63507	2	60S ribosomal protein L14
D3ZHA0	1	Filamin-C
A0A0G2KAN1	6	Collagen alpha-2(I) chain
D3ZPN7	3	60S ribosomal protein L21
A0A0G2JU82	5	Microtubule-actin cross-linking factor 1

P08503	5	Medium-chain specific acyl-CoA dehydrogenase, mitochondrial
A0A0G2JSY2	3	Calpain inhibitor
P09456	6	cAMP-dependent protein kinase type I-alpha regulatory subunit
P19944	2	60S acidic ribosomal protein P1
Q6IFU8	5	Keratin, type I cytoskeletal 17
G3V928	1	Prolow-density lipoprotein receptor-related protein 1
A0A0G2K905	6	Arp2/3 complex 34 kDa subunit
Q78P75	2	Dynein light chain 2, cytoplasmic
A0A0G2JSI5	2	Chymotrypsin-like elastase family member 1
A0A0H2UHE1	3	Succinate--CoA ligase [ADP/GDP-forming] subunit alpha, mitochondrial
Q4KM71	6	Splicing factor proline/glutamine rich (Polypyrimidine tract binding protein associate
D4A6A2	4	Heterogeneous nuclear ribonucleoprotein A3-like
D3ZM69	1	Erythrocyte membrane protein band 4.1-like 2
Q5M860	6	Rho GDP dissociation inhibitor beta
P04055	2	Phospholipase A2
Q07984	2	Translocon-associated protein subunit delta
P41123	2	60S ribosomal protein L13
A0A0F7RQJ6	2	D-dopachrome tautomerase
P60123	5	RuvB-like 1
P04256	6	Heterogeneous nuclear ribonucleoprotein A1
P07335	6	Creatine kinase B-type
P54311	1	Guanine nucleotide-binding protein G(I)/G(S)/G(T) subunit beta-1
P13832	6	Myosin regulatory light chain RLC-A
G3V624	1	Coronin
D3ZD11	2	Microsomal signal peptidase 25 kDa subunit
P15429	3	Beta-enolase
Q6MG61	6	Chloride intracellular channel protein 1
P16446	4	Phosphatidylinositol transfer protein alpha isoform
Q7TPB1	5	T-complex protein 1 subunit delta
Q5U2X6	2	PAT complex subunit CCDC47
Q5U3Y8	4	Transcription factor BTF3
Q68G31	5	Phenazine biosynthesis-like domain-containing protein
B1H249	5	Glucosamine 6-phosphate N-acetyltransferase
O70351	3	3-hydroxyacyl-CoA dehydrogenase type-2
A0A0G2JSG6	3	Adenylate kinase 2, mitochondrial
B5DEL9	2	40S ribosomal protein S7
F1LM66	5	116 kDa U5 small nuclear ribonucleoprotein component
Q4QRB8	6	Argininosuccinate lyase
Q4QQS4	1	RuvB-like helicase
E0A3N4	6	Serpina3n-like protein
Q4KM73	3	UMP-CMP kinase
F1MA18	4	Y-box-binding protein 3
P50503	5	Hsc70-interacting protein
Q8R3Z7	1	EH-domain-containing 4
Q4KLI7	6	Splicing factor 3a, subunit 3
A0A0G2JUE4	5	Golgin subfamily A member 4

Q6PAH0	1	Apolipoprotein E
B5DFK6	2	AP-3 complex subunit delta
B5DFC9	6	Nidogen-2
D4A4K4	5	Vacuolar protein sorting 13 homolog C
Q60587	4	Trifunctional enzyme subunit beta, mitochondrial
P85973	6	Purine nucleoside phosphorylase
Q45QN0	1	Guanine nucleotide binding protein alpha inhibiting 2
P49432	3	Pyruvate dehydrogenase E1 component subunit beta, mitochondrial
P39052	1	Dynamin-2
Q3B8Q2	4	Eukaryotic initiation factor 4A-III
Q5RKG9	3	Eukaryotic translation initiation factor 4B
Q6AYS7	3	Aminoacylase-1A
M0R961	5	Far upstream element-binding protein 2
O35077	4	Glycerol-3-phosphate dehydrogenase [NAD(+)], cytoplasmic
G3V9W6	5	Aldehyde dehydrogenase
B2RZ74	6	U1 small nuclear ribonucleoprotein 70 kDa
Q3B8Q1	1	Nucleolar RNA helicase 2
F1LM33	1	Leucine-rich PPR motif-containing protein, mitochondrial
Q5VLR5	2	BWK4
F1LQS6	6	Xanthine dehydrogenase
Q6AYD5	5	Eukaryotic peptide chain release factor GTP-binding subunit ERF3B-like
F1LND7	2	Farnesyl pyrophosphate synthase
Q07009	5	Calpain-2 catalytic subunit
G3V8A5	4	Vacuolar protein sorting-associated protein 35
Q5QD51	5	A-kinase anchor protein 12
P62836	4	Ras-related protein Rap-1A
A0A0G2KB63	2	Prohibitin
Q3MHS7	3	GDP-D-mannose dehydratase
D3ZD31	1	Mannose receptor, C type 1
P13084	2	Nucleophosmin
P10888	2	Cytochrome c oxidase subunit 4 isoform 1, mitochondrial
E9PT66	5	Splicing factor 3b, subunit 3
D3ZNA3	5	60S ribosomal protein L7a
Q5XIC1	3	Mannose-1-phosphate guanyltransferase alpha
Q5FWT1	3	Protein FAM98A
Q6AXS5	2	Plasminogen activator inhibitor 1 RNA-binding protein
D3ZSY4	6	Eosinophil peroxidase
O55211	2	40S ribosomal protein S2
Q6P9V7	6	Proteasome (Prosome, macropain) activator subunit 1
Q6AXW2	6	RCG25684, isoform CRA_a
D4AEC0	6	Histone H2A
Q02874	1	Core histone macro-H2A.1
A0A0U1RRP9	1	C3/C5 convertase
G3V7I0	2	Peroxiredoxin 3
P13221	3	Aspartate aminotransferase, cytoplasmic
Q91ZN1	6	Coronin-1A

Q4V8I6	5	60S ribosomal protein L11
B4F7C9	2	Dolichyl-diphosphooligosaccharide--protein glycotransferase
P13255	2	Glycine N-methyltransferase
Q62867	2	Gamma-glutamyl hydrolase
A0A0G2K1Z9	5	Histocompatibility 13 (Predicted), isoform CRA_d
Q9JMJ4	5	Pre-mRNA-processing factor 19
A0A0G2K8H0	5	Caprin-1
Q63639	6	Retinal dehydrogenase 2
Q9WU82	4	Catenin beta-1
P32232	3	Cystathionine beta-synthase
Q6AYG5	5	Ethylmalonyl-CoA decarboxylase
F7FLB2	1	Pgm2 protein
D4A8T3	2	Coatomer subunit zeta
Q63598	6	Plastin-3
Q5I0H9	4	Protein disulfide-isomerase A5
P20761	6	Ig gamma-2B chain C region
F7EPE0	6	Prosaposin
Q9EQX9	4	Ubiquitin-conjugating enzyme E2 N
D3ZD09	3	Cytochrome c oxidase subunit
P14604	2	Enoyl-CoA hydratase, mitochondrial
P31399	2	ATP synthase subunit d, mitochondrial
Q5I0G4	4	Glycine--tRNA ligase
P19234	3	NADH dehydrogenase [ubiquinone] flavoprotein 2, mitochondrial
R9PXU4	1	Thioredoxin-disulfide reductase
D3ZBN0	1	Histone H1.5
D3ZL10	5	Collagen type VI alpha 6 chain
P0DP31	6	Calmodulin-3
D4A5I9	5	Unconventional myosin-6
Q641Y0	2	Dolichyl-diphosphooligosaccharide--protein glycosyltransferase 48 kDa subunit
P34064	1	Proteasome subunit alpha type-5
P20788	2	Cytochrome b-c1 complex subunit Rieske, mitochondrial
A0JN30	3	Canopy 2 homolog (Zebrafish)
P07895	5	Superoxide dismutase [Mn], mitochondrial
Q569B4	5	Ighg protein
P20762	5	Ig gamma-2C chain C region
Q3B8R4	6	Igh-6 protein
P06302	4	Prothymosin alpha
Q6P7P5	2	Basic leucine zipper and W2 domain-containing protein 1
F1LMW7	4	Myristoylated alanine-rich C-kinase substrate
Q9JLH7	4	CDK5 regulatory subunit-associated protein 3
Q6IN22	6	Cathepsin B
A0A0G2K0V8	1	Nuclear distribution protein C homolog
Q812C4	3	Translation initiation factor 4A, isoform 1 (Fragment)
P21670	1	Proteasome subunit alpha type-4
P42676	5	Neurolysin, mitochondrial
Q63396	1	Activated RNA polymerase II transcriptional coactivator p15

Q91Y78	5	Ubiquitin carboxyl-terminal hydrolase isozyme L3
D4ABD7	5	Thyroid hormone receptor interactor 11
D3ZD97	5	RNA helicase
Q4V8H8	4	EH domain-containing protein 2
G3V6W6	5	Proteasome 26S subunit, ATPase 6
G3V6S3	4	Calumenin
Q6Q0N1	1	Cytosolic non-specific dipeptidase
Q6TXG7	4	Serine hydroxymethyltransferase
B2RZ24	2	Succinate-CoA ligase subunit beta (Fragment)
D4A830	3	Inorganic diphosphatase
P22734	1	Catechol O-methyltransferase
D3ZH41	1	Cytoskeleton-associated protein 4
D3ZE15	2	Complex I-B16.6
G3V843	6	Prothrombin
P85968	1	6-phosphogluconate dehydrogenase, decarboxylating
Q4KLP0	2	Probable 2-oxoglutarate dehydrogenase E1 component DHKTD1, mitochondrial
P63331	1	Serine/threonine-protein phosphatase 2A catalytic subunit alpha isoform
F1LPM3	6	Sorbin and SH3 domain-containing protein 2
D3ZUV3	3	Eukaryotic translation initiation factor 2A
Q5I0F0	2	Developmentally-regulated GTP-binding protein 1
A0A1B0GWQ7	6	Component 3 of promoter of RISC
G3V8B6	5	26S proteasome non-ATPase regulatory subunit 1
Q923M1	2	Mitochondrial peptide methionine sulfoxide reductase
O08629	3	Transcription intermediary factor 1-beta
Q6VV72	4	Eukaryotic translation initiation factor 1A
Q6U6G5	4	Zinc finger CCCH domain-containing protein 15
D3ZEI0	2	60S ribosomal protein L28
Q4KLJ1	4	RCG61762, isoform CRA_a
A1L1J8	2	RAB5B, member RAS oncogene family
P62898	2	Cytochrome c, somatic
D3ZXI0	2	Pyrroline-5-carboxylate reductase
P63170	4	Dynein light chain 1, cytoplasmic
Q6XDA0	5	Spectrin beta chain
A0A0G2K8B7	5	RNA helicase
P16391	1	RT1 class I histocompatibility antigen, AA alpha chain
P62845	2	40S ribosomal protein S15
A0A0G2JZM2	1	Protein transport protein SEC23
D4A6P3	5	Shootin-1
P62856	2	40S ribosomal protein S26
B3DM93	3	Programmed cell death protein 4
P25304	1	Agrin
A0A0G2K261	5	Isoleucyl-tRNA synthetase
P63329	3	Serine/threonine-protein phosphatase 2B catalytic subunit alpha isoform
P12075	2	Cytochrome c oxidase subunit 5B, mitochondrial
G3V667	5	Integrin subunit alpha 6
F1M7S4	2	Mast cell carboxypeptidase A

D3ZWL6	4	Adenosylhomocysteinase-like 2
F1LVV4	6	Regulator of chromosome condensation 2
G3V6B0	3	Pyridoxal-dependent decarboxylase domain-containing 1
F1MAN8	5	Laminin subunit alpha 5
D4AD70	2	60S ribosomal protein L38
A0A096MJ39	6	Glycoprotein IIIb
P62198	4	26S proteasome regulatory subunit 8
A0A0G2K1S6	4	Malic enzyme
G3V9Z6	4	Septin
Q9Z1W6	3	Protein LYRIC
Q9WU49	2	Calcium-regulated heat stable protein 1
B0BNB2	2	Density-regulated protein
A1A5L1	4	Bleomycin hydrolase
B2GV15	2	Dihydrolipoamide acetyltransferase component of pyruvate dehydrogenase comple
D3ZUD8	4	Transmembrane 9 superfamily member
Q63009	6	Protein arginine N-methyltransferase 1
Q5PQX1	6	Torsin-1A-interacting protein 1
A0A0G2K2P5	4	Tight junction protein ZO-1
P18886	3	Carnitine O-palmitoyltransferase 2, mitochondrial
P36972	6	Adenine phosphoribosyltransferase
B2RZD5	3	RCG41580, isoform CRA_a
P04157	6	Receptor-type tyrosine-protein phosphatase C
G3V8V1	1	Granulin, isoform CRA_c
P62494	6	Ras-related protein Rab-11A
D3ZZR9	2	Peptidylprolyl isomerase
Q641Y2	2	NADH dehydrogenase [ubiquinone] iron-sulfur protein 2, mitochondrial
G3V6C4	6	UDP-glucose 6-dehydrogenase
Q5I0P2	3	Glycine cleavage system H protein, mitochondrial
Q6AXS3	1	Protein DEK
Q6AXX6	2	Peroxiredoxin-like 2A
Q9WTR7	5	Signal peptidase complex catalytic subunit SEC11C
D3ZPV8	3	Gamma-glutamylcyclotransferase
P62332	6	ADP-ribosylation factor 6
P62138	1	Serine/threonine-protein phosphatase PP1-alpha catalytic subunit
D3ZFF4	3	Lysocardiolipin acyltransferase 1
D3ZE72	5	Methionine aminopeptidase
Q6AYK3	3	Inositol-3-phosphate synthase 1
B5DEY8	6	Sorting nexin
D3ZE21	5	Transmembrane emp24 protein transport domain-containing 11
P52631	6	Signal transducer and activator of transcription 3
M0R6L9	6	Ferritin
B0BN63	4	Activator of Hsp90 ATPase activity 1
Q6IFW0	6	RCG32608
O54975	4	Xaa-Pro aminopeptidase 1
P07154	6	Procathepsin L
Q5M7W5	1	Microtubule-associated protein 4

Q8VHT6	4	Arsenite methyltransferase
P12749	2	60S ribosomal protein L26
D3ZE09	5	Talin 2
B2RZD4	2	60S ribosomal protein L34
Q505J8	3	Phenylalanine--tRNA ligase alpha subunit
F1LXP8	5	Rho GTPase activating protein 18 (Predicted), isoform CRA_a
A0A0G2JYI0	5	LPS-responsive beige-like anchor protein
P97519	3	Hydroxymethylglutaryl-CoA lyase, mitochondrial
B2GV99	6	Myl6 protein
O35112	5	CD166 antigen
A0A0G2K8Z9	5	Kinesin family member 13B
Q9JK11	1	Reticulon-4
M0R5F8	4	High mobility group nucleosome-binding domain-containing protein 5
Q4QQW3	5	Hydroxyacid-oxoacid transhydrogenase, mitochondrial
M0RAD5	3	ATP-dependent Clp protease proteolytic subunit
F1LMV6	4	Desmoplakin
A0A0G2K0Q7	1	Myosin light chain kinase, smooth muscle
Q5U2R8	1	Interferon activated gene 204
Q6P9U8	5	Eukaryotic translation initiation factor 3 subunit H
P11232	4	Thioredoxin
P15651	4	Short-chain specific acyl-CoA dehydrogenase, mitochondrial
A0A0G2K542	1	UTP--glucose-1-phosphate uridylyltransferase
D3ZGE2	1	Myeloperoxidase
Q5PPG7	5	Eukaryotic translation initiation factor 2D
B2RZD6	3	NDUFA4, mitochondrial complex-associated
D3ZBT9	6	Protein phosphatase 6, regulatory subunit 3
F7F2F3	5	Heat shock 70 kDa protein 4L
O35824	4	DnaJ homolog subfamily A member 2
F1M111	5	Myosin VC
P11915	6	Sterol carrier protein 2
O88656	1	Actin-related protein 2/3 complex subunit 1B
G3V9J1	3	Uncharacterized protein
Q4KMA2	4	UV excision repair protein RAD23 homolog B
Q4KLF8	4	Actin-related protein 2/3 complex subunit 5
A0A0G2JZQ1	2	RNA helicase
A0A0G2K695	1	Myoferlin
P24329	3	Thiosulfate sulfurtransferase
Q3KRE2	4	Methyltransferase like 7A, isoform CRA_b
Q63798	6	Proteasome activator complex subunit 2
Q5M7V8	1	Thyroid hormone receptor-associated protein 3
P11497	3	Acetyl-CoA carboxylase 1
M0RCH0	2	Eukaryotic translation initiation factor 3 subunit I
D4A8F2	6	Ras suppressor protein 1
Q64591	3	2,4-dienoyl-CoA reductase [(3E)-enoyl-CoA-producing], mitochondrial
P08461	5	Dihydrolipoyllysine-residue acetyltransferase component of pyruvate dehydrogenas mitochondrial

Q91Y81	6	Septin-2
Q32PX6	1	Ras homolog family member G
A0A0G2JSS9	6	Atlastin-3
A0A0G2JW94	4	Clathrin interactor 1
B2RYT5	3	Cox7a2l protein
Q6AYS8	1	Estradiol 17-beta-dehydrogenase 11
A0A6F8P9J1	1	Lysosomal associated membrane protein 1
O35162	2	Heat shock 70 kDa protein 13
P63088	6	Serine/threonine-protein phosphatase PP1-gamma catalytic subunit
Q68FS0	1	Similar to 14-3-3 protein sigma (Fragment)
D3ZIF5	5	Adaptor-related protein complex 3 subunit beta 1
Q5XIG8	5	Serine-threonine kinase receptor-associated protein
F1LR10	5	LIM domain and actin-binding protein 1
G3V8T4	6	DNA damage-binding protein 1
D3ZF97	2	Endoplasmic reticulum lectin 1
P07323	5	Gamma-enolase
Q6P9Y4	1	ADP/ATP translocase
Q6IFV9	1	Keratin 34
P52944	1	PDZ and LIM domain protein 1
Q71SA3	5	Thrombospondin 1
Q68G41	2	Dodecenoyl-Coenzyme A delta isomerase (3,2 trans-enoyl-Coenzyme A isomerase)
Q8CHN6	5	Sphingosine-1-phosphate lyase 1
Q4QQV6	1	Lymphocyte specific 1, isoform CRA_a
D4A3K5	6	Histone H1.1
D4A3I4	4	Transcription factor BTF3
F1LY19	5	RCG38820
Q6P7Q4	2	Lactoylglutathione lyase
P18437	2	Non-histone chromosomal protein HMG-17
F1M6Q3	1	Collagen type IV alpha 2 chain
B0BNA5	6	Coactosin-like protein
G3V811	6	Protein-glutamine gamma-glutamyltransferase
P43278	4	Histone H1.0
Q68FT9	5	Selenocysteine lyase
Q6DGG0	4	Peptidyl-prolyl cis-trans isomerase D
P22791	6	Hydroxymethylglutaryl-CoA synthase, mitochondrial
D3ZN95	5	Host cell factor C1
Q5XIN6	3	Mitochondrial proton/calcium exchanger protein
B0BNN3	5	Carbonic anhydrase 1
B2RZ78	5	Vacuolar protein sorting-associated protein 29
B0BNE6	2	Complex I-23kD
P62959	2	Histidine triad nucleotide-binding protein 1
Q5I0E7	5	Transmembrane emp24 domain-containing protein 9
Q6AY02	5	Splicing factor 45
D3Z900	3	Mitochondrial amidoxime reducing component 2
A0A0G2K896	1	Similar to RIKEN cDNA 1300017J02
Q5RJN0	5	Complex I-20kD

A0A0G2JW29	5	alpha-1,2-Mannosidase
D3ZFK5	5	Microsomal signal peptidase 12 kDa subunit
Q5U1Y2	5	Rac family small GTPase 2
Q5XI19	6	Fermitin family homolog 2 (Drosophila)
Q4V8E2	5	26S proteasome regulatory subunit RPN11
Q5XI18	4	Acyl-Coenzyme A binding domain containing 3
D3ZU56	1	Sister chromatid cohesion protein PDS5 homolog B
Q3B8N9	3	Biphenyl hydrolase-like
G3V818	1	Alpha-parvin
Q6PEC1	4	Tubulin-specific chaperone A
A0A096MKE0	5	Aspartate-beta-hydroxylase
F1LPD0	3	Collagen alpha-1(XV) chain-like
B2GUZ5	1	F-actin-capping protein subunit alpha-1
P10868	3	Guanidinoacetate N-methyltransferase
D3ZBS2	1	Inter-alpha-trypsin inhibitor heavy chain H3
P27605	2	Hypoxanthine-guanine phosphoribosyltransferase
Q7TT49	1	Serine/threonine-protein kinase MRCK beta
Q5I0D7	3	Xaa-Pro dipeptidase
P19139	1	Casein kinase II subunit alpha
Q5M7T5	6	Antithrombin-III
G3V644	2	Complex I-9kD
A0A0G2JW28	6	Gamma-adducin
A0A0H2UHZ2	6	Nucleosome assembly protein 1-like 4
D4A4T9	4	Cysteine and histidine-rich domain-containing protein 1
Q3T1K5	5	F-actin-capping protein subunit alpha-2
A0A0G2K022	6	Reticulocalbin-3
D4A1Q0	2	60S ribosomal protein L36
A0A5P8DHK3	1	Nesprin 2 long isoform
Q63186	3	Translation initiation factor eIF-2B subunit delta
P04041	1	Glutathione peroxidase 1
Q68FW9	5	COP9 signalosome complex subunit 3
Q5M7A4	3	Ubiquitin-like modifier-activating enzyme 5
B0BMW4	3	GNAS complex locus
Q6AXQ0	4	SUMO-activating enzyme subunit 1
Q6TUD3	5	LRRGT00111
Q8CJH4	6	GM2 activator protein
D3Z9U2	6	CD163 antigen (Predicted)
G3V8D5	2	6-phosphogluconolactonase
Q6P6T4	3	Echinoderm microtubule-associated protein-like 2
B2RYB8	1	Integrin beta
A0A0H2UHV6	5	Calcineurin subunit B type 1
A0A0G2KAN5	5	Enhancer of rudimentary homolog
D4A133	6	H(+)-transporting two-sector ATPase
G3V617	1	Mitogen-activated protein kinase 14
G3V918	1	Trifunctional purine biosynthetic protein adenosine-3
D4A8H8	1	Cytoplasmic FMR1-interacting protein

P05370	6	Glucose-6-phosphate 1-dehydrogenase
Q6IN15	1	Acyl-CoA synthetase long-chain family member 5
Q66H32	6	RAN GTPase-activating protein 1
A0A0G2K248	5	S-adenosylmethionine synthase
Q6JE36	4	Protein NDRG1
Q5RJK5	2	Chromobox 3
F1M265	1	Uncharacterized protein
A0A0G2K648	4	WD repeat-containing protein 61
P11240	3	Cytochrome c oxidase subunit 5A, mitochondrial
P29266	3	3-hydroxyisobutyrate dehydrogenase, mitochondrial
A0A0G2K135	5	Complement factor I
A2VCW2	4	Caveolin (Fragment)
P63322	4	Ras-related protein Ral-A
O35397	3	Caspase-6
G3V6P8	6	Guanine nucleotide-binding protein subunit gamma
A0A0G2JSQ7	5	Kallikrein 1
D4AEP0	5	Adenylosuccinate synthetase isozyme 2
B2GV92	6	Ptges3 protein
Q99P74	2	Ras-related protein Rab-27B
Q68FQ7	6	RNA polymerase II-associated protein 3
P36860	4	Ras-related protein Ral-B
Q09167	4	Serine/arginine-rich splicing factor 5
A0A0G2K2Y3	6	Aminopeptidase
Q5U211	1	Sorting nexin-3
A0A0G2K038	2	Oligoribonuclease, mitochondrial
Q7TP52	2	Carboxymethylenebutenolidase homolog
F1M635	5	Protein LBH
P25093	5	Fumarylacetoacetase
P13668	6	Stathmin
Q5BJN3	1	Tia1 cytotoxic granule-associated RNA-binding protein-like 1
M0R7E6	6	Arsenite-resistance protein 2
Q63065	2	[Pyruvate dehydrogenase (acetyl-transferring)] kinase isozyme 1, mitochondrial
Q8VHU4	1	Elongator complex protein 1
A0A0G2K2F6	2	40S ribosomal protein S15a
A0A0G2JZ75	5	Plasmalemma vesicle-associated protein
A0A0G2JY69	5	Peroxin-19
B1WC32	4	SUMO-activating enzyme subunit 2
P61212	4	ADP-ribosylation factor-like protein 1
Q7TP42	2	Translocation protein SEC62
F1LSD3	5	Integrin beta
Q68G33	3	Golgi reassembly-stacking protein 2
Q9Z2S9	1	Flotillin-2
D3ZVS2	3	L-2-hydroxyglutarate dehydrogenase
Q9Z1N1	3	Fructose-1,6-bisphosphatase isozyme 2
Q04970	4	GTPase NRas
D3ZTX0	5	Transmembrane emp24 domain-containing protein 7

D3ZP13	2	Sulfhydryl oxidase
F1LN42	5	Tensin 1
Q32Q06	5	AP-1 complex subunit mu-1
G3V8L1	1	PYD and CARD domain-containing
P62870	4	Elongin-B
Q64350	2	Translation initiation factor eIF-2B subunit epsilon
F1M0U5	5	NBAS subunit of NRZ tethering complex
P63036	6	DnaJ homolog subfamily A member 1
A0A0G2K3V7	4	DNA topoisomerase I
P52925	6	High mobility group protein B2
Q5FVQ6	1	Interferon-gamma-inducible GTPase Ifgga3 protein
P32198	6	Carnitine O-palmitoyltransferase 1, liver isoform
Q66HR2	4	Microtubule-associated protein RP/EB family member 1
A0A0G2K075	3	Signal sequence receptor subunit alpha
D4A994	5	ER membrane protein complex subunit 1
C5NTX8	5	Tensin3
A0A0H2UHZ6	1	60 kDa poly(U)-binding-splicing factor
A0A0G2K1L8	1	Brain acid soluble protein 1
B2RYS2	2	Cytochrome b-c1 complex subunit 7
A0A140UHY3	4	RNA-binding protein EWS-like
B2GV72	2	Carbonyl reductase 3
D3ZD89	5	N(alpha)-acetyltransferase 15, NatA auxiliary subunit
G3V8C4	6	Chloride intracellular channel protein
Q5U1W8	6	High-mobility group nucleosome binding domain 1
D3ZCS3	4	Poly(rC)-binding protein 4
A0A0G2K0I3	1	Nicotinamide phosphoribosyltransferase
D3ZN76	5	Protein transport protein sec16
Q4V8B0	2	Oxidation resistance protein 1
D4A4X4	4	Cingulin
Q5HZE4	2	Methylthioribose-1-phosphate isomerase
D4A617	1	Ectonucleoside triphosphate diphosphohydrolase 1
Q9Z144	2	Galectin-2
P62076	3	Mitochondrial import inner membrane translocase subunit Tim13
F1M957	6	von Willebrand factor
Q9WVA1	2	Mitochondrial import inner membrane translocase subunit Tim8 A
A0A0G2K1Q7	5	Ankyrin-3
G3V827	5	Cysteine conjugate-beta lyase 1, isoform CRA_a
Q68G11	6	Casein kinase II subunit beta
Q63524	3	Transmembrane emp24 domain-containing protein 2
A0A0A0MXW1	3	2-oxoisovalerate dehydrogenase subunit beta, mitochondrial
P62890	2	60S ribosomal protein L30
Q99N27	5	Sorting nexin-1
A0A0G2K7X7	6	Complement C7
A0A0G2K161	5	Band 4.1
P11505	5	Plasma membrane calcium-transporting ATPase 1
P04961	1	Proliferating cell nuclear antigen

Q66HC5	1	Nuclear pore complex protein Nup93
Q9JIY6	3	Probable N-acetyltransferase CML6
Q5RKJ9	2	RAB10, member RAS oncogene family
D4A746	4	GDP-mannose pyrophosphorylase B
Q6Q7Y5	5	Guanine nucleotide-binding protein subunit alpha-13
B1WC84	6	Canopy 4 homolog (Zebrafish)
P00173	4	Cytochrome b5
P16975	1	SPARC
Q5U316	3	Ras-related protein Rab-35
D3ZUX7	2	Acyl-CoA synthetase family member 3
A9CME3	5	Complement component 4 binding protein, alpha
Q6MG60	4	N(G),N(G)-dimethylarginine dimethylaminohydrolase 2
D4A9U6	3	E3 ubiquitin-protein ligase
V5QSV9	6	Galectin
Q9EST6	6	Acidic leucine-rich nuclear phosphoprotein 32 family member B
P15205	1	Microtubule-associated protein 1B
Q6AYZ7	5	Phosphoribosyl pyrophosphate synthetase-associated protein 1
B5DF91	6	ELAV-like protein 1
P54001	1	Prolyl 4-hydroxylase subunit alpha-1
E9PTK9	1	Ankyrin repeat and KH domain-containing 1
B2RZD1	2	Protein transport protein Sec61 subunit beta
D4AAB5	5	Peptidase M20 domain-containing protein 2
Q63083	6	Nucleobindin-1
F1LRI7	4	AP2-associated protein kinase 1
D3ZF39	1	UDP-N-acetylglucosamine pyrophosphorylase 1
A0A0G2JU07	2	Ubiquitin-conjugating enzyme E2 variant 2
P28042	3	Single-stranded DNA-binding protein, mitochondrial
Q5U2R9	5	Sec1 family domain-containing 2
Q66HG5	4	Transmembrane 9 superfamily member 2
A0A0G2K7M2	4	UV excision repair protein RAD23
Q6P762	5	Alpha-mannosidase
D3ZLC1	6	Lamin B2
Q6P769	4	Uncharacterized protein
F1LQT0	5	DnaJ homolog subfamily C member 25
P27139	4	Carbonic anhydrase 2
D4AAE9	2	CDGSH iron sulfur domain 2
P08082	4	Clathrin light chain B
Q5U3Z7	4	Serine hydroxymethyltransferase
O70593	5	Small glutamine-rich tetratricopeptide repeat-containing protein alpha
Q63663	1	Guanylate-binding protein 1
B1WC16	5	BCL2-associated transcription factor 1, isoform CRA_a
F1M0X6	5	Mago homolog B, exon junction complex subunit
D4A8G7	5	SNW domain-containing protein 1
F1LPC7	5	Hepatoma-derived growth factor
A0A0H2UHP9	5	RCG39700, isoform CRA_d
D4A2D7	5	Importin 4

Q5U2V1	6	Peptidylprolyl isomerase
D3ZW55	6	Inosine triphosphate pyrophosphatase
Q811X6	5	Lambda-crystallin homolog
Q9Z339	2	Glutathione S-transferase omega-1
Q80ZA3	6	Alpha-2 antiplasmin
M0R9L3	1	Sorting nexin
Q62930	1	Complement component C9
Q91W30	1	Aldose reductase-like protein
O08984	5	Delta(14)-sterol reductase LBR
D3ZUB0	1	Reticulocalbin 1
B2RYM3	1	Inter-alpha trypsin inhibitor, heavy chain 1
A0A0G2K0L0	5	3-phosphoadenosine-5-phosphosulfate synthase
Q561R9	4	Endoribonuclease LACTB2
G3V681	6	DNA helicase
A0A0G2KAZ7	6	Heterogeneous nuclear ribonucleoprotein D-like
P52759	4	2-iminobutanoate/2-iminopropanoate deaminase
F5CC78	5	Dystrophin Dp71e
F1SW39	1	PC4 and SFRS1 interacting protein 1
D4A5A6	1	DNA-directed RNA polymerase subunit
Q63942	2	GTP-binding protein Rab-3D
Q63356	5	Unconventional myosin-Ie
Q6P9U9	1	Inosine-5-monophosphate dehydrogenase
Q62868	4	Rho-associated protein kinase 2
P35231	6	Regenerating islet-derived protein 3-alpha
A0A0G2K776	6	Prostacyclin synthase
Q9EPV3	5	Endothelial monocyte-activating polypeptide II (Fragment)
M0RC99	5	Ras-related protein Rab-5A
B0K008	2	Eukaryotic translation initiation factor 1
D4A5K6	3	CAAX prenyl protease
Q9WVK7	3	Hydroxyacyl-coenzyme A dehydrogenase, mitochondrial
P80385	5	5-AMP-activated protein kinase subunit gamma-1
D3ZVU4	5	Ribokinase
Q4KLZ6	5	Triokinase/FMN cyclase
Q5M823	5	NudC domain-containing protein 2
M0R5N4	5	Prefoldin subunit 4
D3ZZW1	4	Dedicator of cyto-kinesis 1
O35964	1	Endophilin-A2
Q62639	4	GTP-binding protein Rheb
A0A0G2JUY4	5	Ig-like domain-containing protein
Q3B7D0	5	Oxygen-dependent coproporphyrinogen-III oxidase, mitochondrial
Q4FZT2	3	Protein phosphatase methylesterase 1
P42930	6	Heat shock protein beta-1
Q7M0F9	5	Aldehyde dehydrogenase (NAD), cytosolic (Fragments)
G3V709	5	Nicotinate phosphoribosyltransferase
P25286	5	V-type proton ATPase 116 kDa subunit a1
G3V8L7	1	Integrin alpha M

Q5M7U6	4	Actin-related protein 2
Q8VHV8	5	Selenoprotein S
B2RZ08	2	RGD1563325 protein
Q9JHB5	4	Translin-associated protein X
Q02356	3	AMP deaminase 2
Q5U2U7	1	mRNA cap guanine-N7 methyltransferase
B5DF65	3	Biliverdin reductase B
P17955	6	Nuclear pore glycoprotein p62
P15684	5	Aminopeptidase N
D4A4J0	1	FACT complex subunit
F1LWE6	4	Musashi RNA-binding protein 2
D3ZXP7	2	Actin-related protein 2/3 complex subunit 1A (Fragment)
Q9QVC8	4	Peptidyl-prolyl cis-trans isomerase FKBP4
D4A7J8	1	PRP4 pre-mRNA processing factor 4 homolog (Yeast)
P85845	5	Fascin
A0A0G2K2P4	5	Cytochrome P450, family 2, subfamily t, polypeptide 1
Q5WRG2	6	Angiogenin
Q5RJR2	6	Twinfilin-1
A0A0G2K7T6	1	Nuclear pore complex protein Nup155
Q6AZ26	1	C-terminal binding protein 1
D4AA63	5	Ubiquilin 2
F8WFT7	4	Anion exchange protein
Q63965	2	Sideroflexin-1
D3ZUX5	3	Coiled-coil-helix-coiled-coil-helix domain containing 3 (Predicted), isoform CRA_a
Q9WV97	3	Mitochondrial import inner membrane translocase subunit Tim9
Q7TQN4	5	Nuclear factor kappaB subunit p65
F7EZ89	5	TBC1 domain family, member 15
P70562	5	Class A basic helix-loop-helix protein 15
F7EVX2	3	Translation initiation factor eIF-2B subunit beta
G3V8Q1	3	Coatomer subunit epsilon
Q5M7T9	5	Threonine synthase-like 2
P08753	1	Guanine nucleotide-binding protein G(i) subunit alpha-3
D3ZCL3	4	U1 small nuclear ribonucleoprotein C
D3ZNQ6	5	Ubiquitin-conjugating enzyme E2M
P22062	4	Protein-L-isoaspartate(D-aspartate) O-methyltransferase
Q09326	5	Alpha-1,6-mannosyl-glycoprotein 2-beta-N-acetylglucosaminyltransferase
P28492	5	Glutaminase liver isoform, mitochondrial
A0A0G2KAM4	1	Fascin
A0A0G2QC53	1	Epidermal growth factor receptor pathway substrate 15-like 1
A0A0G2K1N3	5	Hydroxymethylbilane synthase
P63074	2	Eukaryotic translation initiation factor 4E
Q4FZU0	4	Acid phosphatase 6, lysophosphatidic
P04166	3	Cytochrome b5 type B
P21571	5	ATP synthase-coupling factor 6, mitochondrial
Q5U2T9	1	Peptidylprolyl isomerase
P43138	4	DNA-(apurinic or apyrimidinic site) endonuclease

D3ZS58	3	NADH dehydrogenase [ubiquinone] 1 alpha subcomplex subunit 2
D3ZD73	4	RNA helicase
A0A0G2K2L1	4	Podocalyxin
D3ZX42	1	G protein-coupled receptor 21 (Predicted), isoform CRA_a
P70582	5	Nuclear pore complex protein Nup54
Q66HA6	6	ADP-ribosylation factor-like protein 8B
Q6PDU1	3	Serine/arginine-rich splicing factor 2
P70541	5	Translation initiation factor eIF-2B subunit gamma
D3ZXH7	4	Aly/REF export factor
P62859	4	40S ribosomal protein S28
P18421	4	Proteasome subunit beta type-1
Q5U2Q3	5	Ester hydrolase C11orf54 homolog
Q6IRJ7	1	Annexin
A0A140TAD1	2	ADP-sugar pyrophosphatase
P70565	5	Junction plakoglobin
Q925Q9	5	SH3 domain-containing kinase-binding protein 1
Q9EQH5	6	C-terminal-binding protein 2
D4A3T3	4	Chromobox 1
D4A6X4	6	Acylphosphatase
B0K030	1	DnaJ (Hsp40) homolog, subfamily B, member 1 (Predicted), isoform CRA_b
Q5RKI5	6	FLII, actin-remodeling protein
D3ZMX6	5	Syntrophin, beta 2
D3ZVR9	6	Phosphoglucomutase 5
I6L9G6	4	TAR DNA-binding protein 43
F1LNM0	5	Disks large homolog 1
Q03346	5	Mitochondrial-processing peptidase subunit beta
B2GV14	4	Taxilin alpha
D3ZF26	6	Tankyrase 1-binding protein 1
P02764	6	Alpha-1-acid glycoprotein
P25977	1	Nucleolar transcription factor 1
F1LVT5	5	RUN domain-containing 1
D3ZN37	1	Rho-associated protein kinase
D3ZJ92	1	Pre-mRNA processing factor 40 homolog A (Yeast) (Predicted)
D3ZGW2	1	AP-1 complex subunit gamma
Q64599	5	Hemiferrin
Q63269	1	Inositol 1,4,5-trisphosphate receptor type 3
D3ZN27	5	DnaJ heat shock protein family (Hsp40) member C13
P61959	4	Small ubiquitin-related modifier 2
P20767	1	Ig lambda-2 chain C region
D3ZPI4	5	GTPase activating RANGAP domain-like 4 (Predicted), isoform CRA_a
D4ACW1	5	NOP2 nucleolar protein
A0A0G2K9Q1	5	Golgi phosphoprotein 3-like
Q5BJN1	3	START domain containing 10, isoform CRA_b
F1LM09	5	Ubiquitin carboxyl-terminal hydrolase 7
Q5BJP3	2	Ubiquitin-fold modifier 1
D3Z898	6	Deoxynucleoside triphosphate triphosphohydrolase SAMHD1

D3ZT90	2	Glutaryl-CoA dehydrogenase
D4A631	5	Brefeldin A-inhibited guanine nucleotide-exchange protein 1
D3ZRX9	6	Calponin (Fragment)
A0A0G2K0I1	5	Spermatogenesis-associated 5
G3V824	5	Insulin-like growth factor 2 receptor
O35828	6	Coronin-7
Q4QQV8	5	Charged multivesicular body protein 5
Q4KLL4	5	Transmembrane 9 superfamily member 4
Q9QYL8	2	Acyl-protein thioesterase 2
D3ZD83	4	Major facilitator superfamily domain-containing 10
B2RZB6	4	U6 snRNA-associated Sm-like protein LSm8
Q6P686	6	Osteoclast-stimulating factor 1
Q6GQP4	1	Ras-related protein Rab-31
A0A0G2JY08	1	Unconventional myosin-XVIIIa
Q6AYQ9	6	Peptidyl-prolyl cis-trans isomerase
D3Z8L7	3	Ras-related protein R-Ras
Q4KM87	1	Actin-like 6A
D3ZZN3	5	Acetyl-coenzyme A synthetase
F8WFK6	5	Glutathione peroxidase
G3V7P1	4	Syntaxin-12
Q8VHI8	2	Vesicle transport protein SEC20
B2GV01	6	Metastasis-associated 1 family, member 2
B1WBS4	6	Similar to Vacuolar protein sorting 26 homolog (VPS26 protein homolog), isoform CR
P97612	1	Fatty-acid amide hydrolase 1
Q497C3	5	Methyltransferase-like 26
D4A9Q3	3	40S ribosomal protein S23
B2RYU7	4	Cbx5 protein
O54921	6	Exocyst complex component 2
A0A0G2K751	4	DnaJ homolog subfamily C member 8
Q6P734	1	Plasma protease C1 inhibitor
P20069	5	Mitochondrial-processing peptidase subunit alpha
Q5BJU0	3	RAS-related 2
G3V7G0	4	Dynein light intermediate chain
B2GUZ9	3	Fam49b protein
F1LPB4	5	A-kinase-anchoring protein 9
B2GV54	5	Neutral cholesterol ester hydrolase 1
Q6P4Z9	6	COP9 signalosome complex subunit 8
E9PU07	6	Eosinophil-associated, ribonuclease A family, member 1
A0A0G2K1U8	2	Chymotrypsin-C
P63086	1	Mitogen-activated protein kinase 1
P31430	3	Dipeptidase 1
P32821	6	Trypsin V-A
D3ZLK9	3	ATP-dependent (S)-NAD(P)H-hydrate dehydratase
D3ZQI1	6	Glutathione peroxidase
G3V7K5	6	NPC intracellular cholesterol transporter 1
P27867	3	Sorbitol dehydrogenase

Q4QR75	5	Exosome complex component RRP45
Q6AZ50	3	Ubiquitin-like-conjugating enzyme ATG3
O70257	5	Syntaxin-7
A0A1B0GWS5	1	Complement C5 (Fragment)
D3ZKQ4	5	RAB, member RAS oncogene family-like 6
P29419	2	ATP synthase subunit e, mitochondrial
A0A0G2K613	1	Uncharacterized protein
Q4KM69	6	COP9 (Constitutive photomorphogenic) homolog, subunit 5 (Arabidopsis thaliana)
Q5XIP9	6	Transmembrane protein 43
A0A0G2K3I9	4	Dual specificity protein phosphatase
G3V982	6	Engulfment and cell motility 2, ced-12 homolog (C. elegans), isoform CRA_b
M0R6H1	3	Similar to threonine aldolase 1
Q9EPF2	5	Cell surface glycoprotein MUC18
B5DFI1	5	Conserved oligomeric Golgi complex subunit 1 (Fragment)
Q9WUH9	5	Fibrillin-2
Q5BMA6	6	Leukemia-associated Rho guanine nucleotide exchange factor
Q5M943	6	THUMP domain containing 1
B3IYD2	2	Ubiquitin-fold modifier-conjugating enzyme 1
F7F0B1	4	Zinc finger CCCH-type antiviral protein 1
Q4V8C2	5	Centromere/kinetochore protein zw10 homolog
Q9QZT0	3	CUB and zona pellucida-like domain-containing protein 1
Q568Z6	5	IST1 homolog
D3ZAX5	6	Calcium homeostasis endoplasmic reticulum protein
D4A7L4	2	Complex I-ESSS
H9KVF6	1	Non-specific serine/threonine protein kinase
D3ZUF9	4	Pitrilysin metalloproteinase 1
F1LVF5	5	Coiled-coil domain-containing 149
D4A465	6	Late endosomal/lysosomal adaptor, MAPK and MTOR activator 2
Q62658	3	Peptidyl-prolyl cis-trans isomerase FKBP1A
Q10743	5	Disintegrin and metalloproteinase domain-containing protein 10
Q4QQS6	2	Dolichyl-phosphate beta-glucosyltransferase
Q5XIM5	2	Protein CDV3 homolog
M0RCH8	4	Ribosomal L1 domain-containing protein 1-like
P69682	5	Adaptin ear-binding coat-associated protein 1
F1LMQ3	5	26S proteasome non-ATPase regulatory subunit 8
G3V7L8	6	ATPase, H+ transporting, V1 subunit E isoform 1, isoform CRA_a
P10818	2	Cytochrome c oxidase subunit 6A1, mitochondrial
D4A9K3	5	D-aminoacyl-tRNA deacylase
Q66H98	4	Caveolae-associated protein 2
Q5U2N0	5	CTP synthase 2
D3ZP96	6	DNA replication licensing factor MCM2
P61480	5	Ribosome biogenesis protein WDR12
A0A0G2JYN4	3	ERGIC and golgi 2
A0A140TAH1	3	Hepatocyte growth factor-regulated tyrosine kinase substrate
Q63688	6	25-hydroxycholesterol 7-alpha-hydroxylase (Fragment)
Q75N36	6	Cytoplasmic CAR retention protein

Q8CF97	5	Deubiquitinating protein VCPIP1
P23711	6	Heme oxygenase 2
F1M820	5	Sorbin and SH3 domain-containing protein 1
Q5FVC4	5	DnaJ (Hsp40) homolog, subfamily B, member 12
Q5XI07	6	Lipoma-preferred partner homolog
P19643	3	Amine oxidase [flavin-containing] B
G3V8G2	5	26S proteasome non-ATPase regulatory subunit 5
D3ZL86	5	HEAT repeat-containing protein 1
O89046	4	Coronin-1B
D3ZUT9	6	Integrator complex subunit 3
D3ZTB5	6	S100 calcium-binding protein A13
A0A0G2K160	5	AT hook-containing transcription factor 1
Q63747	4	Small nuclear ribonucleoprotein-associated protein
D3Z9I1	2	Cytochrome c oxidase assembly factor 3
Q66HD3	1	Nuclear autoantigenic sperm protein
A0A0G2K0T8	2	Family with sequence similarity 25, member A
Q6PDU7	6	ATP synthase subunit g, mitochondrial
P36506	4	Dual specificity mitogen-activated protein kinase kinase 2
O55159	6	Epithelial cell adhesion molecule
B0BMZ1	2	Family with sequence similarity 241 member B
D3ZJH9	6	Malic enzyme
P97621	5	Polyubiquitin (Fragment)
F8WG67	6	Acyl-CoA thioesterase 7, isoform CRA_a
B4F7A5	1	CD99 molecule (Xg blood group)
D4A9L2	5	Serine/arginine-rich splicing factor 1
Q5JC29	1	Epidermal growth factor receptor pathway substrate 15 isoform B
D3ZY71	5	RCG21156
B2GVB9	6	Fermitin family member 3
A0A096MJP9	6	DNA damage-inducible 1 homolog 2
A0A023IM54	6	TAP binding protein
P07151	6	Beta-2-microglobulin
Q00981	4	Ubiquitin carboxyl-terminal hydrolase isozyme L1
P17046	4	Lysosome-associated membrane glycoprotein 2
G3V8P4	5	Protein-tyrosine-phosphatase
B5DEQ4	4	RCG27500, isoform CRA_c
D3ZUM4	6	Beta-galactosidase
G3V8P5	5	Similar to cDNA sequence BC017158
P01015	1	Angiotensinogen
Q64240	1	Protein AMBP
D4A1V7	6	MOB kinase activator 1B
Q66HG4	5	Galactose mutarotase
B0K025	2	Oligosaccharyltransferase complex subunit OSTC
Q9WUF4	2	Vesicle-associated membrane protein 8
M0R623	5	Nucleolar GTP-binding protein 1
P10252	4	CD48 antigen
Q5FVK6	6	Coiled-coil and C2 domain-containing protein 1B

P07340	4	Sodium/potassium-transporting ATPase subunit beta-1
G3V816	5	Nucleoside diphosphate kinase
Q6IMX7	5	Hsp70-binding protein 1
A0A0G2JZ53	5	Baculoviral IAP repeat-containing 6
Q9JJP9	1	Ubiquilin-1
D3Z9P1	4	3-ketodihydrosphingosine reductase
P00388	4	NADPH--cytochrome P450 reductase
B3DM95	4	Parathymosin
A0A0G2K7X3	4	Nuclear ubiquitous casein and cyclin-dependent kinase substrate 1
Q712U5	5	cAMP-regulated phosphoprotein 19
Q3KRC5	4	tRNA-dihydrouridine(47) synthase [NAD(P)(+)]-like
B2RZ33	2	Cytoplasmic protein
P37397	6	Calponin-3
B2RYS0	2	Cox7a2 protein
Q63691	6	Monocyte differentiation antigen CD14
D3ZI16	3	COP9 signalosome complex subunit 6
Q6AYQ8	5	Acylpyruvase FAHD1, mitochondrial
F1LT10	1	Afadin
A0A0G2JXP1	4	Glycogenin-1
Q99PV2	5	Syntaxin binding protein 3, isoform CRA_a
F1M1H0	5	2-deoxy-D-ribose 5-phosphate aldolase
D3ZUL8	6	TRAMP-like complex RNA-binding factor ZCCHC8
Q5HZY0	4	UBX domain-containing protein 4
D3Z9K4	6	Pleckstrin homology domain-containing O2
H6X320	5	Pentaxin
Q4VBH2	4	tRNA nucleotidyl transferase 1
Q56A27	4	Nuclear cap-binding protein subunit 1
Q6MGA0	1	MHC class II antigen
B2GV06	2	Succinyl-CoA:3-ketoacid coenzyme A transferase 1, mitochondrial
Q62920	4	PDZ and LIM domain protein 5
F1LNI5	6	Protein phosphatase 1G
Q5M7T6	2	V-type proton ATPase subunit
E9PTN4	5	SRSF protein kinase 1
Q99068	4	Alpha-2-macroglobulin receptor-associated protein
Q00238	1	Intercellular adhesion molecule 1
D3ZF86	4	ARFGEF family member 3
B2RYD7	3	Dolichyl-diphosphooligosaccharide--protein glycotransferase
G3V9Q4	6	Serine/threonine kinase 38
Q8K1P7	1	Transcription activator BRG1
F1LZ05	6	Poly [ADP-ribose] polymerase
D4A7U1	1	Zyxin
Q5XIE6	3	3-hydroxyisobutyryl-CoA hydrolase, mitochondrial
D4ADF5	6	Programmed cell death 5
Q63016	2	Large neutral amino acids transporter small subunit 1
P45479	1	Palmitoyl-protein thioesterase 1
Q4KLI0	1	SWI/SNF-related matrix-associated actin-dependent regulator of chromatin subfami

Q6AYU3	1	DnaJ homolog subfamily B member 6
Q920F5	5	Malonyl-CoA decarboxylase, mitochondrial
P37996	4	ADP-ribosylation factor-like protein 3
Q99MI7	6	NEDD8-activating enzyme E1 catalytic subunit
M0RA08	5	Perilipin
Q56R17	6	Importin subunit alpha
P63312	6	Thymosin beta-10
Q9ES53	5	Ubiquitin recognition factor in ER-associated degradation protein 1
F7FJQ3	1	Epididymal secretory protein E1
Q07014	5	Tyrosine-protein kinase Lyn
Q4KM64	4	Protein jagunal homolog 1
P27881	1	Hexokinase-2
A0A0G2KAI8	5	Developmentally-regulated GTP-binding protein 2
Q03555	6	Gephyrin
Q09030	3	Trefoil factor 2
F1LQX8	5	Inositol 1,4,5-trisphosphate receptor type 1
O54772	5	SWI/SNF-related matrix-associated actin-dependent regulator of chromatin subfami
D3ZXF9	1	Mitochondrial ribosomal protein L12
P97878	5	Exocyst complex component 5
Q6AYF8	1	RCG43931
P10687	5	1-phosphatidylinositol 4,5-bisphosphate phosphodiesterase beta-1
Q1PBJ1	1	Lactadherin
F1LMA7	5	C-type mannose receptor 2
B2GV57	5	Cysteinyl-tRNA synthetase
A0A0G2JY82	5	Secretory carrier-associated membrane protein
A0A0G2K714	4	Endophilin-B1
V9H0R3	3	Cholecystokinin-2 receptor (Fragment)
D3ZGY2	5	Ubiquitinyl hydrolase 1
A0A0G2K1A1	5	Uncharacterized protein LOC500855
G3V6U9	5	Actin-histidine N-methyltransferase
B1PLB1	4	CD34 antigen (Predicted)
A0JN17	6	V-Ki-ras2 Kirsten rat sarcoma viral oncogene homolog
A0A0G2K1W1	1	RAB11 family-interacting protein 5
A0A0G2JYU6	6	Formin-like 1
B1WC67	1	RCG29001
A0A0G2K950	1	3-phosphoadenosine-5-phosphosulfate synthase
Q5PQJ6	4	Pyrroline-5-carboxylate reductase 3
Q6NYB8	5	Ifi47 protein
D3ZSV1	4	Hypothetical LOC287541 (Predicted), isoform CRA_c
A0A0G2K3L8	1	WD repeat and FYVE domain-containing 1
G3V7Z8	4	Poly(A) binding protein, nuclear 1, isoform CRA_a
B5DEJ5	5	Eefsec protein
B5DEZ4	6	Tcerg1 protein
A0A0G2K110	5	Eukaryotic translation initiation factor 6
P06238	5	Alpha-2-macroglobulin
Q02589	5	[Protein ADP-ribosylarginine] hydrolase

F1M1R8	5	Lymphoid-restricted membrane protein
Q5XFW1	3	Myelin basic protein
Q5U2X8	3	Acyl-CoA thioesterase 9
A0A3B0J380	1	Complement C1q subcomponent subunit A
D3ZLT1	2	Complex I-B18
Q6MG73	6	C3/C5 convertase
Q792H5	1	CUGBP Elav-like family member 2
F1M5V2	1	GLI pathogenesis-related 2
Q63450	4	Calcium/calmodulin-dependent protein kinase type 1
G3V9M1	4	RNA helicase
Q6AY98	6	Ube2e2 protein (Fragment)
D3ZBM3	5	Ferrochelatase
D4A9W3	2	D-glutamate cyclase
D3Z8X6	6	E3 ubiquitin-protein ligase
A0A0A0MXY5	6	ATP-dependent 6-phosphofructokinase
D3Z881	1	TBC1 domain family, member 4
Q4QQS8	1	Nuclear pore complex protein Nup85
Q3S4A4	5	ADP-ribosylation factor GTPase activating protein 1 heart isoform
D3ZRV0	5	DCN1-like protein
G3V8E4	5	Similar to D7Wsu128e protein
B5DFM8	6	Pre-mRNA-splicing factor SPF27
O70377	6	Synaptosomal-associated protein 23
M0R5W4	5	Mevalonate kinase
Q5PPN5	6	Tubulin polymerization-promoting protein family member 3
D4ACW0	6	RNA-binding motif protein 6
P09656	2	Serine protease inhibitor Kazal-type 1-like
B1WBQ0	6	CDC5 cell division cycle 5-like (S. pombe)
Q6AYK5	1	Cell growth-regulating nucleolar protein
A0A0G2JY73	5	Eukaryotic translation initiation factor 4 gamma, 3
P28648	5	CD63 antigen
P25086	6	Interleukin-1 receptor antagonist protein
D3ZFP4	6	DNA helicase
P62775	6	Myotrophin
D4AD75	5	Dpy-19-like C-mannosyltransferase 1
D3ZTW9	4	Endonuclease G-like 1 (Predicted), isoform CRA_d
D4A5X1	5	Stromal interaction molecule 2
B0BNM9	6	Glycolipid transfer protein
G3V829	5	Far upstream element-binding protein 3
D3ZQ74	6	Procollagen-lysine 5-dioxygenase
F1LMP9	5	Disabled homolog 2
D4A510	5	SWI/SNF-related, matrix-associated, actin-dependent regulator of chromatin, subfar
P61515	2	Putative 60S ribosomal protein L37a
A0A0G2JWP1	5	Cleavage stimulation factor subunit 2
B5DER4	5	Mitochondrial ribosomal protein L1
O55171	6	Acyl-coenzyme A thioesterase 2, mitochondrial
G3V6N2	5	Transmembrane emp24 protein transport domain containing 4 (Predicted), isoform

Q7TP07	2	Da1-12
G3V7J2	1	Interferon-inducible double-stranded RNA-dependent protein kinase activator A
G3V8N0	2	Sideroflexin 2
Q80W89	2	NADH dehydrogenase [ubiquinone] 1 alpha subcomplex subunit 11
Q496Z0	5	Elongator complex protein 2
D3ZUY8	1	AP-2 complex subunit alpha
Q8R2E7	2	FAS-associated death domain protein
D3ZE59	5	Transmembrane protein 115
G3V985	5	Similar to SCO cytochrome oxidase deficient homolog 1 (Yeast) (Predicted)
Q7TP91	5	Surfeit locus protein 1
Q5XIA8	4	Growth hormone-inducible transmembrane protein
Q7TPJ5	6	Ac2-190
D4A031	1	RNA helicase
B1H267	2	Sorting nexin-5
A0A140UHX0	6	Protein kinase C delta type
Q562B3	2	Nckap1 protein (Fragment)
D4AE49	5	Mtr4 exosome RNA helicase
Q7TMA5	5	Apolipoprotein B-100
P36201	5	Cysteine-rich protein 2
D3ZLX3	3	MOB kinase activator 2
Q6MG48	5	Protein PRRC2A
Q6B345	6	Protein S100-A11
M0R7A6	5	Intersectin 2
Q3B8R6	1	Alpha-2-glycoprotein 1, zinc
D4A772	5	Dystrobrevin
Q4KM77	2	Etoposide-induced protein 2.4 homolog
Q9JHX4	5	Caspase-8
P69736	2	Endothelial differentiation-related factor 1
B2RYQ2	4	Serine/threonine-protein phosphatase 2A activator
Q2A121	5	Alpha-ketoglutarate-dependent dioxygenase FTO
F1M2M6	5	NTF2 domain-containing protein
F1LXA0	2	NADH dehydrogenase [ubiquinone] 1 alpha subcomplex subunit 12
P97615	4	Thioredoxin, mitochondrial
M0R3V4	2	Myeloid-derived growth factor
Q5BJX1	4	39S ribosomal protein L41, mitochondrial
D3ZDZ1	1	Amyloid beta (A4) protein-binding, family B, member 1 interacting protein
G3V648	4	1-acylglycerol-3-phosphate O-acyltransferase 3
Q5XI90	4	Dynein light chain Tctex-type 3
Q62975	5	Protein Z-dependent protease inhibitor
Q498R7	2	CXXC motif containing zinc binding protein
Q52KJ9	6	Thioredoxin domain containing 1
F1M609	5	Acyl-coenzyme A oxidase
M0RCH5	4	Glucosamine-6-phosphate isomerase
D4ABX6	2	Multimerin 2
P70569	5	Unconventional myosin-Vb
A0A0G2K3V4	1	O-GlcNAc transferase subunit p110

D4AE06	4	Peptidylprolyl isomerase
Q6AYC4	6	Macrophage-capping protein
P31211	5	Corticosteroid-binding globulin
D4ABK7	5	Heterogeneous nuclear ribonucleoprotein H3 (2H9) (Predicted), isoform CRA_c
Q5U2Q5	5	Ribonucleoside-diphosphate reductase
Q5XI29	6	Cleavage and polyadenylation specificity factor subunit 7
A0A096MKE9	5	DNA ligase
Q6AY21	4	G3BP stress granule assembly factor 2
Q4V8H2	6	SEC3-like 1 (S. cerevisiae)
D3ZYL4	5	39S ribosomal protein L50, mitochondrial
Q6AY25	4	Transmembrane emp24 domain-containing protein 3
A0A0G2K9T1	4	E3 ubiquitin-protein ligase
O35263	5	Platelet-activating factor acetylhydrolase IB subunit alpha1
Q5XIG0	1	ADP-ribose pyrophosphatase, mitochondrial
B5DFI0	6	RNA cytidine acetyltransferase
M0R776	4	Mitochondrial ribosomal protein S36
Q6AYY8	3	Acetyl-coenzyme A transporter 1
P13599	1	IgG receptor FcRn large subunit p51
D4A2H2	1	Serine palmitoyltransferase 1
P60522	5	Gamma-aminobutyric acid receptor-associated protein-like 2
Q56A18	5	SWI/SNF-related matrix-associated actin-dependent regulator of chromatin subfami
G3V6C9	5	Non-specific serine/threonine protein kinase
D4A2H4	1	DENN domain-containing 3
Q6UPR8	5	Endoplasmic reticulum metallopeptidase 1
Q99P75	4	Ras-related protein Rab-9A
P58200	5	Vesicle transport through interaction with t-SNAREs homolog 1B
A0A0G2K9D6	5	SWI/SNF-related, matrix-associated, actin-dependent regulator of chromatin, subfar
D3ZQI0	1	Polymerase (RNA) II (DNA directed) polypeptide J (Predicted)
D3ZU48	5	Uncharacterized protein
O55158	5	Tetraspanin
Q6IRE4	5	Tumor susceptibility gene 101 protein
P37285	4	Kinesin light chain 1
P97829	4	Leukocyte surface antigen CD47
A0A0G2K6I4	4	ENAH, actin regulator
A0A0G2K8K3	1	Heparin cofactor 2
P11951	2	Cytochrome c oxidase subunit 6C-2
Q5XID1	4	Anamorsin
B0BNI6	2	Similar to solute carrier family 25, member 35, isoform CRA_a
M0RDW3	5	N-acetyltransferase 8 (GCN5-related) family member 4
A0A0G2JSH9	4	Peroxiredoxin-2
Q4KM38	6	FUS interacting protein (Serine-arginine rich) 1
Q9JHZ9	5	Sodium-coupled neutral amino acid transporter 3
A0A0G2K9S4	1	Unconventional myosin-Va
F1LPG9	4	WASH complex subunit 2C
B5DER3	5	Hypertrophic agonist responsive protein B64, isoform CRA_b
D3ZFJ3	6	SH3 domain-binding protein 1

F1M3X3	5	Ig-like domain-containing protein
Q66HF9	1	Leucine-rich repeat flightless-interacting protein 1
Q5FVL2	5	ER membrane protein complex subunit 8
Q4G005	4	General transcription and DNA repair factor IIH helicase subunit XPB
Q68FT1	3	Ubiquinone biosynthesis protein COQ9, mitochondrial
A0A0G2K0V3	4	3(2),5-bisphosphate nucleotidase 1
D3ZWS0	5	Scribble planar cell polarity protein
Q7TSU1	1	Brefeldin A-inhibited guanine nucleotide-exchange protein 2
F1M9C9	5	Histidine--tRNA ligase
Q9Z1B2	4	Glutathione S-transferase Mu 5
F1LRP7	4	Protein argonaute-2
F1LQP9	4	Transportin 1
F1LQM9	5	Exportin 7
A0A0H2UHL6	4	Pro-cathepsin H
G3V6K1	5	Transcobalamin 2, isoform CRA_a
D3ZVU7	6	Histone deacetylase 1
D3ZAY8	1	Pinin
B2RYD0	5	Ube2g2 protein
F1M7S0	4	Membrane-associated guanylate kinase inverted 3
Q68FS8	4	RNA 3-terminal phosphate cyclase
Q6P7R8	4	Very-long-chain 3-oxoacyl-CoA reductase
Q5U2V8	4	ER membrane protein complex subunit 3
D3ZU83	2	ERGIC and golgi 3
D4AB01	2	Histidine triad nucleotide binding protein 2 (Predicted), isoform CRA_a
F1M4W7	5	Cleavage stimulation factor subunit 3
A0A0G2JU01	1	Rho guanine nucleotide exchange factor 1
D3ZP47	5	14 kDa phosphohistidine phosphatase
Q704E8	5	Iron-sulfur clusters transporter ABCB7, mitochondrial
D4ACL2	3	Tetratricopeptide repeat protein 38
D3ZF54	4	Anoctamin
Q498N3	4	Dctn4 protein
P49301	1	C-type lectin domain family 10 member A
F1LZJ4	5	Putative hydroxypyruvate isomerase
D3ZC82	5	Nuclear FMR1-interacting protein 2
D3ZIN7	2	Mitochondrial ribosomal protein S23
Q6IRK9	5	Carboxypeptidase Q
P32089	4	Tricarboxylate transport protein, mitochondrial
F1LT09	5	WD repeat domain 33
D3ZQL1	2	ER membrane protein complex subunit 7
A0A140TAF7	1	Protein O-glucosyltransferase 1
A0A0G2K6R2	6	HSR domain-containing protein
A0A0G2JX72	2	Muscleblind-like-splicing regulator 1
Q811A3	1	Procollagen-lysine,2-oxoglutarate 5-dioxygenase 2
F1LV89	5	Rap1 GTPase-activating protein
Q6TUE3	5	LRRGT00101
Q5PQP2	5	Receptor-binding cancer antigen expressed on SiSo cells

A0A1W2Q5Z6	1	Tax1-binding protein 3
A0A0G2JW52	1	Protein arginine N-methyltransferase 3
B2RYW3	3	Complex I-B22
D4ACC2	6	KN motif and ankyrin repeat domain-containing protein 2
A0A0G2JX30	1	ATPase family, AAA domain-containing 2B
B5DEH0	5	LIM domain-containing protein 1
A2RUW1	3	Toll-interacting protein
O08658	4	Nuclear pore complex protein Nup88
O35274	4	Neurabin-2
F1LMV9	1	Coronin
Q8R424	5	STAM-binding protein
Q6GQY2	2	Multiple coagulation factor deficiency 2
D3ZH12	4	Nitric oxide synthase-interacting protein
A0A0H2UHD9	3	Nicalin
Q5HZV9	6	Protein phosphatase 1 regulatory subunit 7
Q5XI85	2	Aminomethyltransferase
D3ZQB6	2	Cat eye syndrome chromosome region, candidate 5 homolog (Human) (Predicted)
D4AEH3	4	Proteasome (Prosome, macropain) 26S subunit, non-ATPase, 7 (Predicted)
P04916	5	Retinol-binding protein 4
D4A8G5	1	Transforming growth factor-beta-induced protein ig-h3
O08719	4	Ena/VASP-like protein
Q562B9	5	Polynucleotide phosphorylase 1 (Fragment)
Q68FW7	3	Threonine--tRNA ligase, mitochondrial
Q6NX65	5	Programmed cell death protein 10
D3ZK96	5	Poly(A) polymerase
Q66H09	6	Tetratricopeptide repeat domain 1
E9PST0	5	Septin
G3V920	4	RCG61879
Q68FV6	5	Glycosylated lysosomal membrane protein
B2GV55	6	Ubiquitin-conjugating enzyme E2 Q1
D3ZC89	4	Uncharacterized protein
Q08013	2	Translocon-associated protein subunit gamma
F1LQ00	6	Collagen type V alpha 2 chain
Q5PQP7	1	General transcription factor IIIC, polypeptide 2, beta
Q4G069	4	Regulator of microtubule dynamics protein 1
Q5QJC9	5	BAG family molecular chaperone regulator 5
M0R5Q3	5	RAN-binding protein 3
D3ZYM5	6	MTSS I-BAR domain-containing 1
A0A0G2JUM8	5	Cytochrome c oxidase assembly factor 6
O55004	1	Ribonuclease 4
Q5EBA7	5	HGF activator
A0A0G2K3S6	5	RNA binding motif protein 10, isoform CRA_a
O35094	5	Mitochondrial import inner membrane translocase subunit TIM44
P35433	2	Amidophosphoribosyltransferase
Q66H94	1	Peptidyl-prolyl cis-trans isomerase FKBP9
D3ZXJ5	5	Elongation factor-like GTPase 1

D4A9P7	6	BolA family member 2
Q6AYS3	1	Carboxypeptidase
B1H230	2	Dual-specificity mitogen-activated protein kinase kinase 3-like
P11275	2	Calcium/calmodulin-dependent protein kinase type II subunit alpha
O35358	4	Guanine nucleotide binding protein gamma 10 subunit (Fragment)
B1H275	5	BUD23, rRNA methyltransferase and ribosome maturation factor
A0A0G2JWF2	5	Ig-like domain-containing protein
M0R565	5	WD repeat domain 82
Q6AYB2	5	RCG53912, isoform CRA_a
Q5M883	1	Chloride intracellular channel protein 2
Q08290	5	Calponin-1
P62815	4	V-type proton ATPase subunit B, brain isoform
A0A0G2JZ79	6	NAD-dependent protein deacetylase sirtuin-1
D3ZWD6	1	Complement C8 alpha chain
Q642A6	6	von Willebrand factor A domain-containing protein 1
Q66H79	5	Tripartite motif protein 32
A0A0G2JY07	1	DNA helicase
D3ZZR5	1	Small nuclear ribonucleoprotein polypeptide A
Q925G1	6	Hepatoma-derived growth factor-related protein 2
Q6MG85	5	1-acyl-sn-glycerol-3-phosphate acyltransferase
A0A0H2UHE5	5	Protein-serine/threonine phosphatase
D4A8H5	5	Protein phosphatase 4, regulatory subunit 2
Q6GMM8	5	Long-chain fatty acid transport protein 1
F1M7P1	5	DDB1- and CUL4-associated factor 13
Q4KLH4	6	Paraspeckle component 1
Q6PST4	5	Atlastin-1
Q5BJQ6	6	Cleavage stimulation factor subunit 1
Q62819	5	Aromatic L-amino acid decarboxylase
A0A0G2K9P5	3	Conserved oligomeric Golgi complex subunit 5
B1H241	5	Protein Ric-8A
Q569C9	5	Golgi phosphoprotein 3
P25409	3	Alanine aminotransferase 1
F2Z3T4	2	Muscleblind-like protein 2
F1LMH5	1	Protein quaking
B2GV73	6	Actin-related protein 2/3 complex subunit 3
G3V6M8	1	Nucleoporin 37
Q5XI55	1	Peptide-N(4)-(N-acetyl-beta-glucosaminyl)asparagine amidase
F1LRC2	6	Allograft inflammatory factor 1
O08700	5	Vacuolar protein sorting-associated protein 45
Q5M9G1	4	Protein HEXIM1
D3ZZQ4	5	Adipogenesis-associated, Mth938 domain-containing
A4GW50	5	RCG29601
G3V643	3	Trefoil factor 2
P42854	2	Regenerating islet-derived protein 3-gamma
M0R959	5	Corrinoid adenosyltransferase
Q63055	4	ADP-ribosylation factor-related protein 1

Q1PS21	6	DNA replication licensing factor MCM7
F1M951	1	Tyrosine-protein phosphatase non-receptor type 23
Q8K584	3	Arginase
Q9R1J8	1	Prolyl 3-hydroxylase 1
Q9JI56	4	SNAP-29 protein
Q5XIM0	5	BCS1-like protein
P62501	6	TSC22 domain family protein 1
P23347	5	Anion exchange protein 2
B1H227	6	LOC682908 protein
Q9QYW0	1	Protein AATF
D3ZUP5	5	BRICK1 subunit of SCAR/WAVE actin nucleating complex
A0A0G2K8A9	2	HTH La-type RNA-binding domain-containing protein
G3V7Q4	1	Tyrosine-protein phosphatase non-receptor type 12
D4A4S5	6	Folate receptor 2 (Fetal) (Predicted), isoform CRA_a
Q6P7P8	3	X-ray repair cross-complementing protein 5
B4F778	6	Replication factor C (Activator 1) 4 (Predicted), isoform CRA_a
A0A0G2JW85	6	Ras GTPase-activating protein 3
D4A7X1	6	Mitochondrial ribosomal protein S16
D4A7F2	5	Myc-binding protein
D3ZI07	4	Kinesin-like protein
A1A5Q1	6	Poly [ADP-ribose] polymerase
A0A4X0W8E9	6	Active breakpoint cluster region-related protein
A0A0G2K059	6	Calcium uniporter protein
Q6AY65	5	Arfaptin-2
Q5XI64	4	Monoacylglycerol lipase ABHD6
P84100	4	60S ribosomal protein L19
Q6AY86	5	Vacuolar protein sorting-associated protein 26A
Q6AXY8	4	Dehydrogenase/reductase (SDR family) member 1
G3V8H5	1	I-kappa-B kinase
Q19LA7	4	Vesicle-associated membrane protein 2 (Fragment)
Q9JIL8	5	DNA repair protein RAD50
A0A140TAE1	4	Acyl-CoA 6-desaturase
M0R5H1	1	Enhancer trap locus 4
A0A0G2JX56	5	DnaJ (Hsp40) homolog, subfamily C, member 5, isoform CRA_a
Q5XI36	5	CD97 molecule
M0R3Z8	5	RCG28930, isoform CRA_b
P09655	5	Serine protease inhibitor Kazal-type 1
Q08602	2	Geranylgeranyl transferase type-2 subunit alpha
Q6P777	4	Multivesicular body subunit 12A
D3ZLM5	3	NHL repeat containing 2 (Predicted)
B2GV08	1	AP complex subunit sigma
M0RD20	6	Calcium-activated neutral proteinase small subunit
Q9WVH8	2	Fibulin-5
Q68FT8	1	RCG33981, isoform CRA_a
F7ES73	6	Negative regulator of ubiquitin-like proteins 1
D3ZHM7	5	Deoxynucleotidyltransferase, terminal,-interacting protein 2

Q9Z2P5	6	Receptor-interacting serine/threonine-protein kinase 3
A1L108	6	Actin-related protein 2/3 complex subunit 5-like protein
D4A6D9	5	HCLS1-binding protein 3
Q6P791	4	Ragulator complex protein LAMTOR1
D3ZZR3	6	Cathepsin S
A0A0G2K528	1	Syntaxin 16
Q6P4Z6	5	Leucine carboxyl methyltransferase 1
D3ZNI3	5	Programmed cell death 11
Q5FWU2	5	Cysteine desulfurase, mitochondrial (Fragment)
A0A0H2UHZ4	2	Zinc finger Ran-binding domain-containing protein 2
D4A1Q9	4	Tubulin tyrosine ligase-like 12
Q5BJZ6	1	Carnosine N-methyltransferase
D3ZAR1	6	Low density lipoprotein receptor adapter protein 1
Q8VH46	1	Actin filament-associated protein 1
Q9JKL3	1	Apoptosis regulator BAX
D3ZPP2	4	ADP-ribosylation factor-like GTPase 8A
D4A259	6	Polymerase (RNA) II (DNA directed) polypeptide D (Predicted)
D3ZTJ0	5	Protein XRP2
G3V6Y7	4	Caspase activity and apoptosis inhibitor 1
D3Z9D2	5	FYVE and coiled-coil domain autophagy adaptor 1
Q62862	5	Dual specificity mitogen-activated protein kinase kinase 5
Q5XIP2	5	GPI-anchor transamidase
D3ZD19	6	Extracellular link domain-containing 1 (Predicted)
Q7TQ16	5	Cytochrome b-c1 complex subunit 8
A0A0H2UHP0	6	CCCTC-binding factor, isoform CRA_a
Q32PZ3	6	Protein unc-45 homolog A
Q5U2U3	4	Poly [ADP-ribose] polymerase
D3ZUU6	6	C-type lectin domain family 3, member B
Q99PJ4	6	Diphosphomevalonate decarboxylase (Fragment)
Q1EG89	6	Paxillin
Q6AYT0	5	Quinone oxidoreductase
Q499T2	6	Gamma-interferon-inducible lysosomal thiol reductase
O88764	6	Death-associated protein kinase 3
D3ZBR0	5	Keratin-associated protein 3-2
Q6IN10	5	Endothelin-converting enzyme 1
P29117	6	Peptidyl-prolyl cis-trans isomerase F, mitochondrial
M0R3M8	5	Ribosomal RNA-processing 12 homolog
D4AE65	5	Ribosomal RNA-processing 7 homolog A
D4ABC4	6	Protein phosphatase 4, regulatory subunit 3A
Q02974	4	Ketohexokinase
Q7TQ94	4	Deaminated glutathione amidase
P62634	5	Cellular nucleic acid-binding protein
B0BN90	1	rRNA adenine N(6)-methyltransferase
Q06C60	1	BolA family member 1
P00697	1	Lysozyme C-1
Q63400	4	Claudin-3

F7F3Z1	5	Lectin, mannose-binding 2-like
M0R7W3	6	XIAP-associated factor 1
Q6AYA7	4	Riboflavin kinase
F1MAA3	6	Serine/threonine-protein phosphatase 2A 56 kDa regulatory subunit
D3ZZM3	5	Component of oligomeric Golgi complex 4
A0A0G2K0U8	1	tRNA pseudouridine(55) synthase
P19132	5	Ferritin heavy chain
P23640	4	Ras-related protein Rab-27A
A0A0G2JVP1	5	VRK serine/threonine kinase 2
Q08849	1	Syntaxin-3
A0A0G2K781	6	PPFIA-binding protein 1
B5DEP0	2	Dguok protein
Q02293	5	Protein farnesyltransferase subunit beta
M0R5B6	5	Exosome component 1
Q3T1G7	2	Conserved oligomeric Golgi complex subunit 7
D4A1J3	5	Paralemmin 3
F7ENH8	5	Histone deacetylase 2
F1M1D5	5	Tubulin-specific chaperone D
A0A0G2KBC4	1	Receptor protein-tyrosine kinase
Q5M818	5	39S ribosomal protein L16, mitochondrial
A0A096MKE5	4	Zinc finger protein 326 (Fragment)
Q8VHQ7	5	Synaptotagmin-like protein 4
D3Z9M1	5	U6 small nuclear RNA (adenine-(43)-N(6))-methyltransferase
G3V7R1	5	Nuclear pore complex protein Nup50
D3ZG78	5	Zinc finger ZZ-type and EF-hand domain-containing 1
B2RYF8	4	Cnpy3 protein
A0A0G2K5A0	1	Ribosomal RNA-processing protein 43
Q5XIP1	2	Protein pelota homolog
M0R7G4	3	MICOS complex subunit
M0R9Y3	1	Nucleoporin 43
Q5U318	6	Astrocytic phosphoprotein PEA-15
G3V969	5	Tcfcp2 protein
Q66H68	5	RNA-binding protein 47
B3DM88	1	Ehbp1l1 protein
Q3B7D6	5	F-spondin
P54645	1	5-AMP-activated protein kinase catalytic subunit alpha-1
D4A3V3	5	Poly (ADP-ribose) polymerase family, member 12
Q6MGC4	3	H2-K region expressed gene 2, rat orthologue
Q4QR73	5	DnaJ (Hsp40) homolog, subfamily A, member 4
D3Z955	4	Phosphoglucomutase 2-like 1
D3ZIF0	1	Zinc finger protein 512
Q6AY57	1	WD repeat domain phosphoinositide-interacting protein 2
Q505J9	6	Outer mitochondrial transmembrane helix translocase
D3ZJX5	3	Mitochondrial import inner membrane translocase subunit TIM50
Q9QY87	4	CCAAT-box-binding transcription factor (Fragment)
P49793	1	Nuclear pore complex protein Nup98-Nup96

A0A0A0MY43	4	Activating signal cointegrator 1 complex subunit 3
Q32KJ5	1	N-acetylglucosamine-6-sulfatase
E9PSP1	1	Phospholipid transfer protein
P01322	4	Insulin-1
B0K020	4	CDGSH iron-sulfur domain-containing protein 1
P21708	5	Mitogen-activated protein kinase 3
Q9JIL9	1	Nibrin
A0A0A0MXV4	5	Angiotensin-converting enzyme (Fragment)
J7P1Z1	6	Interferon-gamma-inducible GTPase Ifggd2 protein
Q68FQ9	1	LanC lantibiotic synthetase component C-like 2 (Bacterial)
Q4QQU5	2	Protein YIPF6
A0A0H2UHA3	5	Volume-regulated anion channel subunit LRRC8D
Q7TPI8	5	Ac2-256
Q6AYB8	5	Golgin-45
Q499T3	1	Sirpa protein
Q6PCU8	2	NADH dehydrogenase [ubiquinone] flavoprotein 3, mitochondrial
D3ZYQ9	5	E3 ubiquitin protein ligase
Q6AYE6	3	Uncharacterized protein RGD1303117
B5DF51	5	ER membrane protein complex subunit 5
A0A1L1WKE8	5	Calcium modulating ligand (Fragment)
A0A0G2JV46	2	Protein FAM107B
G3V8Y5	5	DNA-directed RNA polymerase subunit beta
Q6TXG9	5	Swi5-dependent recombination DNA repair protein 1 homolog
D3ZNS8	5	Achalasia, adrenocortical insufficiency, alacrimia (Allgrove, triple-A) (Predicted), isof
G3V6L8	4	RCG61894, isoform CRA_a
A0A0G2K1T0	5	Glutaminase
D3ZWR1	2	5, 3-nucleotidase, cytosolic
A0A0G2JYG5	6	Unconventional myosin-IXb
Q4V885	1	Collectin-12
O54861	5	Sortilin
Q62881	5	Nucleolar protein 3
B1WBY2	1	FAD synthase
B4F759	4	PHD finger protein 5A
F1M790	5	Prostaglandin F2 receptor negative regulator
G3V8E2	6	Striatin-interacting protein 1
B2RYT7	2	Haloacid dehalogenase-like hydrolase domain-containing protein 3
P19836	5	Choline-phosphate cytidylyltransferase A
Q8SEZ0	2	NADH-ubiquinone oxidoreductase chain 5
B1WBW0	5	U3 small nucleolar ribonucleoprotein protein MPP10
A0A387KC71	4	Aldo-keto reductase family 1, member C15
Q6P6T5	2	Occludin
D4A511	2	Signal recognition particle 9 kDa protein
B0BMY7	6	Protein tyrosine kinase 9-like (A6-related protein) (Predicted), isoform CRA_b
D3ZXT2	1	Stromal antigen 2
D3ZCA0	4	Pyridoxal phosphate homeostasis protein
D3ZF45	6	La ribonucleoprotein 4B

D3ZGL1	1	Rho GTPase-activating protein 25
P09034	1	Argininosuccinate synthase
Q6T4R6	6	MHC class II antigen
A0A0G2JV16	6	Dedicator of cytokinesis 11
D3ZGF1	5	CD44 antigen
Q9ERR2	5	COMM domain-containing protein 5
B5DEP4	3	28S ribosomal protein L42, mitochondrial
Q810U0	5	Coiled-coil domain-containing protein 50
A9UMW0	5	Ubiquitin-like protein 5
D3ZWF5	5	Transcription and mRNA export factor ENY2
B2GUV2	1	Vacuolar protein sorting-associated protein 52 homolog
D3ZJ01	5	RAB11-binding and LisH domain, coiled-coil and HEAT repeat-containing
G3V631	5	RAB guanine nucleotide exchange factor (GEF) 1 (Predicted), isoform CRA_a
Q5PPI1	6	Serine/arginine-rich splicing factor 9
D4AB70	5	alpha-1,2-Mannosidase
Q5XIJ7	6	Calcium-binding protein 39-like
D3ZAS9	5	DDRGK domain-containing protein 1
M0RDM7	5	GPI ethanolamine phosphate transferase 2
G3V6K3	3	Exosome component 7
D3ZB65	1	BRCA2 and CDKN1A-interacting protein
E9PT04	5	VPS39 subunit of HOPS complex
A0A023IMI6	1	Proteasome subunit beta
P05982	5	NAD(P)H dehydrogenase [quinone] 1
Q5XI86	5	Aminoacyl-tRNA hydrolase
Q4G067	5	Mitochondrial ribosomal protein L44
Q496Z9	5	TRMT1-like protein
F1M471	1	EPM2A-interacting protein 1
A0A0G2JYE0	2	Ataxin 2-like
B1VKB4	1	Synaptopodin
D3ZT01	2	Component of oligomeric Golgi complex 2
D4A962	5	Heterogeneous nuclear ribonucleoprotein U-like 1
D4A7A8	6	Rab7b, member RAS oncogene family
Q2VC85	4	GCS light chain (Fragment)
P16970	1	ATP-binding cassette sub-family D member 3
D3ZAI6	1	5-nucleotidase domain-containing 3
P23606	1	Protein-glutamine gamma-glutamyltransferase K
D3ZL21	1	Non-specific serine/threonine protein kinase
D3ZM03	5	Vacuolar ATPase assembly integral membrane protein VMA21
D3Z9E1	6	Elastin microfibril interfacer 1
Q01579	5	Glutathione S-transferase theta-1
Q99PD6	6	Transforming growth factor beta-1-induced transcript 1 protein
D4A648	1	Non-specific serine/threonine protein kinase
Q6DGF4	5	AN1-type zinc finger protein 6
Q6P742	4	Proteolipid protein 2
D3ZHG8	5	ADP-ribosylation factor-binding protein GGA3
B2RZB7	4	Small nuclear ribonucleoprotein Sm D1

G3V6Y9	1	Peptidyl-prolyl cis-trans isomerase G
Q5HZX2	5	RGD1359201 protein (Fragment)
M0RBT5	5	SDS3 homolog, SIN3A corepressor complex component
Q9ERH3	4	WD repeat-containing protein 7
D3ZE63	2	Uncharacterized protein
A0A0G2JYT1	6	ELKS/Rab6-interacting/CAST family member 1
F1LYQ8	6	FERM, ARHGEF and pleckstrin domain-containing protein 1
G3V6H4	2	Acetylcholine receptor subunit epsilon
Q9JHW0	5	Proteasome subunit beta type-7
P10686	6	1-phosphatidylinositol 4,5-bisphosphate phosphodiesterase gamma-1
Q76IC5	6	Pyroglutamyl-peptidase 1
P04218	5	OX-2 membrane glycoprotein
F1M8K7	5	Dol-P-Man:Man(5)GlcNAc(2)-PP-Dol alpha-1,3-mannosyltransferase
Q5XFW4	1	Mitochondrial ribosomal protein L13
D3ZQ25	1	Fibulin-1
Q923V4	4	F-box only protein 6
G3V9P7	5	Huntingtin
D3ZIY3	4	YTH N(6)-methyladenosine RNA-binding protein 3
Q5BK08	1	Cd209b protein (Fragment)
Q71RR7	5	Guanylate kinase
Q4V897	6	Coiled-coil domain-containing protein 90B, mitochondrial
A0A0G2JZ83	5	ArfGAP with GTPase domain, ankyrin repeat and PH domain 3
Q562A2	5	Zinc finger RNA-binding protein
A0A0G2K8T0	6	Ceramidase
D3ZFY0	4	Selenide, water dikinase
Q68FX4	6	Hematopoietic cell-specific LYN substrate 1
B0BN31	5	FGFR1OP N-terminal like
A0A0G2QC24	1	Leucine rich repeat containing 40
Q156J1	5	Bcl-2-interacting death suppressor
P97584	1	Prostaglandin reductase 1
D3ZF21	5	GPRIN family member 3
D3ZC63	5	Cytidine/uridine monophosphate kinase 2
A0A0H2UHL3	1	Adipocyte enhancer-binding protein 1
Q63448	5	Peroxisomal acyl-coenzyme A oxidase 3
G3V8C6	4	Steroid receptor RNA activator 1
D4ADI4	4	Kinesin-like protein
D3ZEW3	5	TRPM8 channel-associated factor 2
A0JPN6	5	Mediator of RNA polymerase II transcription subunit 22
Q64715	3	Microtubule-associated protein
P29418	3	ATP synthase subunit epsilon, mitochondrial
M0R7I0	1	Importin subunit alpha
A0A0G2KA71	5	Nectin cell adhesion molecule 2
B2RZ38	1	Ras-related GTP-binding protein
D4A5S6	2	ALG1, chitobiosyldiphosphodolichol beta-mannosyltransferase
Q9EPT7	5	Prothrombinase FGL2
D4A533	5	Transmembrane anterior posterior transformation 1

Q3SWT4	1	Protein IWS1 homolog
D3ZUL4	5	B-cell CLL/lymphoma 7C (Predicted), isoform CRA_a
Q6IUR5	4	Neudesin
A0A0G2JYJ1	4	Growth factor receptor-bound protein 10
D3ZXY2	5	PDZ domain containing 8 (Predicted)
A0A0G2K0Y2	4	Insulin-like growth factor 2 mRNA-binding protein 3
D3ZH44	5	Tyrosine-protein kinase
D3ZY96	6	Neutrophilic granule protein
D3ZDJ4	4	Unc-93 homolog B1 (C. elegans)
F1M7L9	6	Uncharacterized protein
D4A264	1	Zinc-binding alcohol dehydrogenase, domain-containing 2
P14272	5	Plasma kallikrein
A0A0G2QC21	5	Rho guanine nucleotide exchange factor 7
Q6AXQ5	5	2,5-phosphodiesterase 12
D3ZIM7	6	Family with sequence similarity 177, member A1
D3ZTF6	5	Phosphatidylinositol-4-phosphate 3-kinase
P49186	5	Mitogen-activated protein kinase 9
D3ZAP7	5	DNA helicase
P62078	5	Mitochondrial import inner membrane translocase subunit Tim8 B
F1LS93	5	SH3-domain-binding protein 2
Q5U2Z5	5	Cap-specific mRNA (nucleoside-2-O-)-methyltransferase 1
Q5BJT0	6	Arginine and glutamate-rich protein 1
A0A0G2JTI7	2	Pre-mRNA-splicing factor 3
B2LYI9	4	Tenascin C
B5DEZ8	1	Plexin domain containing 2 (Predicted)
G3V9C9	1	Similar to D11Bwg0280e protein, isoform CRA_c
Q641Z6	1	EH domain-containing protein 1
Q5RJK8	6	Acyl-CoA-binding domain-containing protein 6
Q75WE7	1	von Willebrand factor A domain-containing protein 5A
B4F775	5	Golgi associated PDZ and coiled-coil motif containing (Predicted)
A0A0G2K101	1	Leucine zipper protein 1
Q5FWY5	1	AH receptor-interacting protein
Q5XHY7	2	Signal transducing adapter molecule 2
D4ACM9	5	Microfibrillar-associated protein 1A
D3ZZ38	5	Sorting nexin
P52590	1	Nuclear pore complex protein Nup107
A0A0G2K5Y1	5	Spermatogenesis-defective protein 39 homolog
Q5XI28	5	Ribonucleoprotein PTB-binding 1
Q6GMN2	5	Brain-specific angiogenesis inhibitor 1-associated protein 2
B5DF45	5	TNF receptor-associated factor 6
F1LTD7	5	DENN domain-containing 4C
B2RYJ7	2	ARP1 actin-related protein 1 homolog B (Yeast)
A0A0G2JX25	5	GMP reductase
D4A915	5	Anoctamin
D3ZPI8	6	Complement C8 gamma chain
Q9WVS2	5	Probable tRNA N6-adenosine threonylcarbamoyltransferase

P97886	5	UDP-glucuronosyltransferase
B2RZ98	1	Ncf4 protein
Q5XIL4	5	Sorbin and SH3 domain containing 3, isoform CRA_b
D4AC45	4	1-acyl-sn-glycerol-3-phosphate acyltransferase
G3V8U3	5	D4, zinc and double PHD fingers family 2 (Predicted)
D3ZJS3	4	RCG43475
B2RYW7	5	Signal recognition particle 14 kDa protein
Q6AYF2	5	LIM and cysteine-rich domains 1
A0A0G2K4T3	1	Arf-GAP with coiled-coil, ANK repeat and PH domain-containing protein 2
D3ZUJ5	5	dTMP kinase
Q9EQT5	4	Tubulointerstitial nephritis antigen-like
P53610	5	Geranylgeranyl transferase type-1 subunit beta
F1M9C7	4	CTD small phosphatase-like protein 2
Q68G38	4	Torsin-1A
D3ZTV7	1	ASH2-like histone lysine methyltransferase complex subunit
D3ZVM5	4	Heat shock protein family A (Hsp70) member 12B
P01681	1	Ig kappa chain V region S211
F1LRM0	5	S-phase cyclin A-associated protein in the ER
B2RYN1	5	Protein-ribulosamine 3-kinase
G3V8S0	1	Serine peptidase inhibitor, Kunitz type, 2
A0A0G2JXG7	1	Capping protein regulator and myosin 1 linker 1
D4AEG7	6	TBC1 domain family, member 13
Q5FVQ0	1	Metal cation symporter ZIP8
Q02353	6	Bifunctional heparan sulfate N-deacetylase/N-sulfotransferase 1
D4ADD7	4	Glutaredoxin 5
B5DEF3	4	Gamma-glutamyl carboxylase
D3ZV96	4	Uncharacterized protein
G3V684	5	Mediator of RNA polymerase II transcription subunit 15
B2RZ72	5	Actin-related protein 2/3 complex subunit 4
B5DF55	5	RCG55706
A0A0G2K9B4	5	39S ribosomal protein L15, mitochondrial
G3V7P6	1	Nudix (Nucleoside diphosphate linked moiety X)-type motif 16 (Predicted), isoform C
D3ZRE7	5	SWAP complex protein (Predicted), isoform CRA_b
D4A9M6	5	A kinase (PRKA) anchor protein 1, isoform CRA_b
P63255	4	Cysteine-rich protein 1
D3ZY40	5	PCF11 cleavage and polyadenylation factor subunit
A0A0G2JZF6	5	NADH:ubiquinone oxidoreductase complex assembly factor 2
D3ZAF7	5	TBC1 domain family, member 2B
Q80W92	5	Protein VAC14 homolog
A0A068FP44	6	Coactivator-associated arginine methyltransferase 1
F1LRB8	3	S-adenosylmethionine synthase
M0R8U1	5	Dynein axonemal heavy chain 5 (Fragment)
Q5XJW2	5	Growth arrest and DNA damage-inducible proteins-interacting protein 1
M0RA79	1	Ig-like domain-containing protein
A0A0G2KAJ7	5	Collagen alpha-1(XII) chain
P36876	5	Serine/threonine-protein phosphatase 2A 55 kDa regulatory subunit B alpha isoform

B5DEN9	2	Vacuolar protein sorting-associated protein 28 homolog
D3ZV75	1	Major facilitator superfamily domain-containing 1
Q5M7T1	3	Probable cytosolic iron-sulfur protein assembly protein CIAO1
D4A4Z0	5	Coiled-coil domain containing 12 (Predicted), isoform CRA_a
D4AA54	5	Pleckstrin homology, MyTH4 and FERM domain-containing H1
B1H282	1	Collagen beta(1-O)galactosyltransferase 1
Q4V7E8	5	Leucine-rich repeat flightless-interacting protein 2
D4A4Y0	5	Exosome component 4
Q6AY55	1	Dephospho-CoA kinase domain-containing protein
B2RYW8	5	MICOS complex subunit Mic10
A0A0G2K0W0	5	Geranylgeranyl pyrophosphate synthase
Q5FVM7	5	DnaJ homolog subfamily C member 16
E9PTE1	4	SON DNA and RNA-binding protein
M0RA15	5	Uncharacterized protein
Q9JJW3	2	ATP synthase membrane subunit K, mitochondrial
P30121	1	Metalloproteinase inhibitor 2
Q91VC0	2	dCTP pyrophosphatase 1
A0A0G2K568	5	Ketimine reductase mu-crystallin
F7F5J1	4	Transcription factor EB
Q68FS9	4	COMM domain containing 10, isoform CRA_a
D3ZJA9	6	Solute carrier family 27 (Fatty acid transporter), member 3 (Predicted)
D4ADZ9	5	Pseudouridine synthase 7
P31722	1	Complement C1q subcomponent subunit C
M0R5M5	3	JRK-like
P55062	2	Bax inhibitor 1
A0A0G2JTX5	4	Dipeptidyl peptidase 4
F1LP46	5	ATP-dependent RNA helicase SUPV3L1, mitochondrial
F1LVK5	4	RRM domain-containing protein
P04638	5	Apolipoprotein A-II
Q812D3	5	Peptidyl-prolyl cis-trans isomerase-like 3
D4ADT3	1	WAPL cohesin release factor
D4A0H4	5	Cullin 2
Q62825	1	Exocyst complex component 3
D3ZEH2	3	FAD-dependent oxidoreductase domain containing 1
Q3SWS8	4	mRNA export factor
A0A140TAC3	6	Epsin-1
A0A0G2JU79	5	RCC1 and BTB domain-containing protein 1
Q5U2Y6	5	Tuftelin-interacting protein 11
P08592	5	Amyloid-beta A4 protein
D4A9B0	6	S1 RNA-binding domain 1
Q62768	5	Protein unc-13 homolog A
M0R3S1	4	Mitochondrial ribosomal protein S21-like
Q9ERA7	1	Mesothelin
A0JN29	5	Endoplasmic reticulum junction formation protein lunapark
Q4V8K2	6	Beta-catenin-like protein 1
A0A096MKG2	5	Nucleolar protein 6

Q9JIM0	5	Double-strand break repair protein MRE11
O08769	1	Cyclin-dependent kinase inhibitor 1B
A0A0H2UI27	5	Septin
Q4KM35	6	Proteasome subunit beta type-10
M0RC47	5	Phosphatidylinositol 3-kinase regulatory subunit alpha
Q5BJQ2	1	Ubiquitin carboxyl-terminal hydrolase MINDY-1
P02625	4	Parvalbumin alpha
O54748	5	Serine/threonine-protein kinase 3
Q6MG51	5	Uncharacterized protein C6orf47 homolog
A0A0H2UI10	6	Protein O-GlcNAcase
D4AAV4	5	Rho GTPase-activating protein 17
D7NIW0	6	HECT-type E3 ubiquitin transferase
B4F7E8	1	Protein Niban 2
A0A0G2JSR9	5	45 kDa calcium-binding protein
G3V9K8	5	Developmentally regulated RNA-binding protein 1
O88794	2	Pyridoxine-5-phosphate oxidase
P70500	2	CDP-diacylglycerol--inositol 3-phosphatidyltransferase
A0A0G2K1M5	4	Divergent protein kinase domain 2A
A0A0G2K7N8	1	Bromodomain PHD finger transcription factor
Q765A7	5	GPI inositol-deacylase
F1LZF2	6	Formin-binding protein 1
D3ZFB2	4	LUC7-like 3 pre-mRNA-splicing factor
D4A3I5	4	Family with sequence similarity 160, member B1
G3V8F7	5	Golgi associated, gamma adaptin ear containing, ARF binding protein 2, isoform CRA
F1M277	6	Leucine-rich repeat-containing 25
Q562C6	5	Leucine zipper transcription factor-like protein 1
D3ZCT5	5	Phosphatase domain-containing, paladin 1
P27274	3	CD59 glycoprotein
B3DMA0	4	Tumor protein p53-inducible protein 11
P05942	6	Protein S100-A4
M0RAI4	5	Glutamyl-tRNA synthetase
D3ZDB9	4	NmrA-like family domain-containing protein 1
A0A0G2JZH9	4	DExH-box helicase 57
