# Supplementary material for: Integrative proteomics, phosphoproteomics and acetylation proteomics analyses of acute pancreatitis in rats
Source: Int J Med Sci. 2023 May 11;20(7):888–900. doi: 10.7150/ijms.81658 (PMC10266050; doi:10.7150/ijms.81658)
Supplement: Supplementary file 4 — Supplementary table 3. [file ijmsv20p0888s4.pdf]

proten	cluster
P15205	3
Q5M7V8	5
F1LN42	2
D3ZXN9	5
Q6MG48	2
Q3ZB99	3
F1M062	4
Q5M7W5	1
M0R3M4	5
D3ZRK0	2
G3V7U2	3
Q3KRF2	4
Q66HD0	2
A0A0G2K2P5	5
A0A0G2JYG5	3
D3ZU13	2
G3V836	1
C0JPT7	5
F1LMW7	5
P31000	5
G3V8L3	4
A0A0G2K089	4
F1LXQ7	4
G3V976	2
A0A0G2JVL3	6
E9PST5	5
Q64715	5
M0R7Z0	2
F1M124	2
Q6TRW4	3
A0A0G2K2B4	5
A0A0G2K2J9	6
P63039	4
Q6AYT4	6
P54316	5
F1LP05	2
P17764	2
Q5XI28	5
D3ZI11	5
P35565	2
F1LW91	5
D3ZZZ9	4
A8C4G9	3
P70478	4
Q9Z1W6	2

Q62622	5
A0A0G2K3N1	5
D3ZM33	2
E9PT23	3
Q3SWT4	1
Q2THW7	4
P19945	5
O35854	4
A0A0G2K8Z9	4
D3ZIE1	2
A0A0G2K1W1	1
Q62785	2
Q3ZAV2	6
P30427	3
A0A0G2JZI2	5
D4A4X4	4
F1M7S0	4
A0A0G2JYE0	5
F1LN75	6
P46462	2
B5DFB6	4
P27321	5
A0A0G2K5C8	5
P07153	2
P24368	4
D4A9D8	4
F1SW39	4
F1M3G7	5
F1LWX5	5
Q5M875	4
P60711	3
M0R965	5
F1LU97	3
A0A0G2K613	3
A0A0G2K911	5
A0A0G2K5M6	3
Q63625	3
O08561	5
F1MA56	3
A0A0G2JZY3	5
G3V6P7	2
A0A140TAA3	5
Q9WU74	3
D3ZIE4	3
B2RZ37	4
Q4G061	2

M0R567	3
A0A0G2K0X1	5
G3V662	4
A0A0G2JYI0	5
A0A0G2K0A8	2
F1M589	1
P13668	2
Q7TQ70	2
A0A1W2Q676	4
D3ZLC3	4
D0UFD0	2
D3ZAY8	5
D3ZVD8	6
M0R3Z8	4
Q9EQS4	2
F1LP26	5
P08426	5
A0A0G2JUK2	3
O08629	4
F1M6T3	4
Q6TQE1	5
D4ACN4	5
Q62901	4
A0A0G2K694	5
D4A5I4	1
D3ZU55	1
A0A0G2JZ69	4
G3V7Q4	3
Q9WVE9	1
A0A0G2JU82	3
G3V852	3
Q8K1P7	2
A7VJC2	6
P85125	3
D4A3G2	1
A0A0G2JXG7	1
P82995	1
P54258	2
D4A0D9	5
D3ZJG8	1
F1M2D4	5
A0A0G2K5I9	5
D3ZP96	1
F1M9C3	6
P62890	2
A7BFV9	2

Q4V8H8	3
P15791	6
P63245	5
D4ACW1	2
D4AE06	3
D3ZF86	4
F1M1X9	5
A0A0G2K6S9	2
D4A9H6	5
D3ZQQ2	5
P62982	2
O35314	5
D4AB71	5
A0A0G2K7N9	2
D4A997	1
Q68FR2	3
Q5BJY9	5
B3DM93	4
P10111	6
Q64303	4
F1LSM0	2
Q5XXR3	1
A0A0G2JTD1	5
Q6AYU2	3
Q9QZ86	1
D3ZKD3	5
A0A0G2JYV0	5
A0A0G2JX74	4
Q6AY30	2
A0A0G2JTB7	6
A0A0G2JYT1	4
D3ZS88	3
M0R750	4
G3V826	4
Q6JE36	4
Q9JI66	1
D4A2D3	2
P12749	4
F1LQ66	2
F1M1R8	2
Q4KLN7	4
D4A8A0	3
A3E0T0	4
Q4V8B0	3
D3ZVF2	3
M0RBD9	3

F1LZ05	1
Q5RKH3	5
F1LN59	4
D3ZKQ4	5
O35775	4
F1LNM0	4
D3ZML2	4
P04157	3
D3ZMG0	2
V9H0R3	4
O08873	3
G3V8R5	6
A0A0G2K2R4	2
A0A0G2K0Q7	3
Q7TP36	6
F1M981	2
D3ZQ89	5
Q9Z2L0	5
D3ZFY7	5
A0A0G2JZF2	3
D4A7U1	1
Q66HF9	2
O88989	2
A0A0G2K7I4	3
B2RYG2	4
Q9WVR6	6
Q4V8F0	3
B2RYD7	2
F1LYG2	2
Q9WU70	2
D3ZWC6	6
A0A0X1KG81	4
F1M265	2
A0A0G2JTZ2	2
A0A0K0WYG5	5
Q1EG89	5
P84586	3
D3ZMR2	4
Q7TSU1	5
Q3KR97	5
Q9JHY8	1
D3ZN95	5
D3ZBN0	1
P51647	4
P19836	5
P70562	4

F6Q5G6	1
Q6AYD3	4
B1WC33	2
B2RZ74	4
A0A0G2K719	6
F1LU18	3
Q08163	3
M0RD54	6
F1LPA4	5
D3ZV15	3
Q6P6T5	6
P53987	4
F1LSM8	6
Q8VHF0	6
Q32PZ5	5
A0A0G2K0M8	5
P14668	3
Q5JCS6	2
Q5U1W8	2
A1A5N2	2
D3ZPQ4	3
A0A0G2K920	4
F1M9D0	4
D3ZHM7	5
Q5XI01	2
G3V8I4	2
F1LNT3	2
F1LM19	5
D4A631	5
P25113	1
D4A4F4	1
F1LM99	2
Q4KM77	4
Q6AY02	4
F1LVX2	2
D3ZAF7	2
P97827	1
Q6P685	2
D3ZYL0	5
G3V6N1	3
D3ZQL6	5
P86411	5
B5DF98	3
Q62910	3
P60868	6
Q8K585	2

D4A652	3
Q62952	5
A0A0H2UHZ2	5
D3Z8R4	6
Q68FX7	4
Q6PDV8	6
A0A1W2Q6Q2	5
F1LYX9	4
D4A777	2
Q5XI38	1
A0A0G2JX30	5
O70185	5
A0A096MKC0	4
F1M0K6	6
F1M024	4
D3ZY28	1
D3ZQW8	3
A0A0G2K382	4
D3ZAF9	3
G3V7X2	3
A0A0G2K4G0	4
P41123	4
Q2KMK7	1
A0A0G2JTI5	3
P61354	2
A0A0G2K5T1	3
D4ACF1	2
M0RAK0	5
A0A0G2JUE4	6
D3ZLW4	2
P40615	2
Q794F9	6
D3ZVK3	3
G3V9L1	2
A0A0G2JYU6	1
D3ZUL8	5
F1MAQ8	5
B1WC06	3
A0A0G2JT35	3
D3ZHZ3	1
F1LVA9	4
P31044	3
G3V7J2	5
Q5HZB6	5
D3ZC56	1
A0A0H2UHG0	2

G3V7F5	6
Q5BJN2	6
B5DFK6	2
D3ZD11	2
B2GV74	6
P61983	5
Q9R1Q2	5
Q5BJK8	3
Q63507	3
D3Z9Z0	6
A0A0G2JXT8	1
F1M9G6	6
G3V7I2	3
P81155	3
C0IXW6	4
A0A0G2K3G9	2
D4A224	4
P42930	4
Q5RJT0	4
Q9R066	5
F1LRV4	3
Q6AXY7	5
A0A140UHX0	1
D3ZBC7	2
P47875	5
B5DF62	5
A0A0G2K7P2	2
A0A0G2JUJ9	4
F1M1D2	5
A0A0G2JZ87	5
M0RAP6	3
F1M6T6	2
B2GV18	6
Q5U2Y6	3
Q5PQR0	2
Q5XIS9	6
G3V8E2	3
Q6PDV6	2
A0A0G2K2B7	6
D4A0U9	1
Q6AYB6	4
Q62947	6
P23606	2
Q2PS20	3
P70705	3
A0A0G2QC02	4

D4A9L9	2
M0R7B4	2
B5DF74	4
F1LT49	2
A0A0G2JXD9	3
F7ENH8	1
O54880	4
O35346	4
D4ABD7	4
D3ZSR2	2
D3ZDJ2	2
A0A1K0FUA6	2
G3V7Q0	5
D4A3V4	5
F1LQJ5	3
Q5M7U8	6
Q3MID3	6
D3ZEX7	3
O08651	4
D3ZYJ5	1
B2RYJ6	3
A0A0H2UHL9	6
G3V6C9	4
A0A0G2JSR7	2
Q5FVJ3	6
P14056	4
G3V6T1	2
F1LQS1	6
Q4V7A6	2
O08769	4
F1LUD3	3
D4ACK7	2
D3ZDU2	4
D3ZJ32	6
Q5XI73	5
G3V7G0	2
A0A0H2UH99	2
P24049	5
D3ZGL6	5
Q02874	3
Q99M64	4
A0A0G2K6T0	5
D4A454	5
R9PXR4	4
A0A023IM54	5
Q6MG49	5

A0A0G2QC53	4
F1LSG8	3
Q9JHU5	6
A0A0G2JZI9	2
Q5FVM4	6
R9PXZ7	3
F2Z3T9	5
A0A0G2JYQ4	1
A0A0G2K6T9	6
Q3MUI1	4
Q6AY58	2
D3ZCP0	2
D3ZAS8	5
Q63083	4
D3ZAP7	2
A0A0G2K3S6	5
B1WBZ1	2
P80386	5
D3ZEA0	4
A0A0G2K8H1	3
A0A0G2JWF7	5
Q5PQX1	4
D4ABP4	2
Q66HE8	6
A0A0G2KAX2	4
D4A563	5
A0A0G2JZ13	3
D3ZF21	4
D3ZIC4	2
B0BNL5	6
A0A0H2UHC3	2
Q6GMM8	2
A0A0G2KAN1	1
D3ZYR1	6
A0A0G2K033	6
P32232	2
Q00969	4
A0A0G2K3A0	5
D3ZCG3	2
D4A8H5	5
D4AE99	1
D3ZCX6	5
A0A0G2JW85	1
A0A0G2JX56	3
Q1JU68	2
D4AA13	4

D4A3E1	1
M0RD40	6
A0A0G2K7D5	2
Q5XIF6	3
Q63553	4
A0A0G2KB61	5
D3ZF70	5
Q4KM38	3
F1M840	3
D3ZLE2	6
Q5U2M8	1
P97839	3
Q5XIG4	2
A0A0G2K2E8	2
P14270	6
D3ZMS1	2
A0A0G2JT06	4
Q4G075	6
A0A0G2K0W5	6
F1LWM1	1
P48675	3
D4AE69	4
B1WC47	5
D3ZBV9	5
Q9Z158	4
A0A096MKE9	4
A0A0G2JUF8	3
D4AAV0	4
A0A0G2JW94	4
Q6AYK4	4
D3ZSI8	3
Q62806	4
D3ZM69	4
Q6AYS6	5
O08623	3
D4ACM9	1
Q9JK25	4
Q1RP74	3
Q6GMN2	2
Q9ES53	2
D3ZU00	4
Q6I7S1	1
A0A0G2JYJ1	5
F1MAA3	3
A0A0G2K960	3
A9UK05	4

D3ZFJ3	1
F1LT94	5
A1L1J6	5
D4A8G7	2
Q63433	5
A0A0G2K1P3	3
Q5BJP5	4
A0A0G2JZ04	1
B1H241	1
Q4KLM7	2
A0A0G2K8N9	4
A0A096MKE2	5
B2RYA7	3
Q9WVR8	3
A1EC67	4
Q0D2L6	3
M0R805	5
Q3B8Q2	2
D4AC95	1
F1M951	1
Q4KLY3	6
A0A0G2JZX5	5
Q5PPP1	2
D3ZBT9	5
D3ZJ92	1
A0A0G2JSV0	5
Q66HS9	6
Q9ESH1	4
C7C5T1	5
M0R9L0	4
Q5M939	1
D3ZTL3	1
A0A0G2KB24	4
P16443	1
Q3T921	1
A0A0G2K9K0	3
D3ZQM2	3
D3ZUC1	5
D4A503	4
D3ZTF6	6
P08010	5
D4A0W7	4
F1LRT4	2
D4A978	2
Q9R063	1
B5DEH3	3

P26284	2
Q9JHY1	6
D4A3K5	3
Q01714	4
B0BND5	2
D7NIW0	5
A0A1W2Q629	2
M0R9N8	3
Q3ZU82	4
M0R7E6	3
Q5XID7	4
D3ZYQ8	1
Q3KRD1	1
B5DEP6	4
P0C0R5	3
B2GV05	3
D3Z8K5	6
F1LR36	2
Q5M860	1
Q5XI97	5
A0A0G2K1U1	4
D4A417	5
A0A0F7R5I4	4
Q5M7X1	5
P35280	2
F1LPJ7	4
D3ZC15	5
D4A421	5
Q4KLG9	4
Q9EQG6	2
D3Z8I4	1
Q4V8B4	3
Q5XID1	4
P0C548	4
D4A9D6	4
D3ZCG2	6
B1H237	2
A0A0G2K5Y1	4
D3Z8S0	4
D4A731	2
Q5TKR9	6
P11030	2
Q99N27	5
D4A8G6	3
F1LR29	3
A0A0G2KAK9	3

B2GV94	6
B2GUU0	3
D3ZI68	5
D4A433	2
M0R439	6
D3ZYY8	2
D3ZMJ7	2
Q587K3	5
Q66H61	1
P52759	4
Q6J4I0	5
F1M3B3	5
Q9ET45	2
D4ACA6	4
F1MAA1	3
D4ACF5	5
A0A0H2UHQ5	1
G3V9S3	1
E9PT49	2
D3ZWX4	3
A0A0G2JXN8	5
D3ZNK1	6
Q5BK26	6
F1LV79	3
A0A1B0GWZ0	4
Q9Z1Z9	2
P22909	3
A1EC95	3
B3DMA0	2
Q62826	4
D3ZFK5	2
A0A0G2K7S6	5
D3ZHL6	6
Q66LH7	5
G3V7X3	1
Q8K3W5	5
D3ZEI6	6
Q8VBW4	4
D4A0W1	5
P97541	2
Q9Z1X1	1
A0A0G2JZL9	6
D3ZYM3	4
D3ZG03	5
D3ZZJ7	4
F1M4I4	3

Q5XI06	4
F1LSC3	2
A0JPN0	5
D4AEG0	6
F1LW74	2
D3ZM54	4
B0BNM4	1
A0A0G2JT78	2
D3ZJR6	5
D3ZDT1	3
D3ZFY8	1
P84100	4
D3ZHK9	5
Q5U2X0	2
Q3MIE4	4
F1LVX1	2
F1LUG5	4
B1WBW0	6
Q535K8	5
F1LT09	2
D3ZBL6	3
A0A0G2K535	5
D3ZVE0	5
F1MA82	1
A0A0G2JVW3	3
A0A0G2JSY8	2
O08589	2
Q3SWU4	5
Q63942	2
Q64315	2
P53565	2
Q920G0	2
F1LW29	3
Q68FV2	2
F1LNL3	3
Q3KRF3	5
D3ZHH8	3
F1M775	2
Q5U3Y8	4
D3ZET9	4
F1LY38	3
A0JPL9	4
A0A0G2K0K8	6
Q5U401	3
D3ZKI5	2
A0A0G2K1B4	5

B0BNB2	3
F1M542	6
B3SVE5	2
A0A0G2K9N2	1
M0R464	5
Q9WUD9	3
Q56B11	5
D4A1W1	3
P09655	6
B5DF93	5
A0A0G2K2Q2	6
Q3KRD5	1
A0A0G2K737	3
P55213	4
O35303	2
B2RYP4	4
F1MAQ4	6
Q5PQP7	6
O35550	6
F1M7L9	4
A0A0G2K1Z9	6
P63326	3
B1WBW4	5
P22734	1
B5DEZ8	3
D4A2W9	1
F1LVL6	6
Q53UA7	5
D4AB33	4
P50545	2
D3ZA78	1
Q75T81	4
D3ZUL5	4
Q5XIT1	4
P0C8E4	2
O55207	3
P97706	1
D3ZSR7	3
B2RZ47	4
F1LPS8	4
Q5BK07	4
D3Z8L5	4
A0A0G2K0M5	5
F1M989	4
A0A096MJA0	3
D3ZJW0	2

A0A0G2K6W6	3
B1WC02	3
A0A0G2K007	4
D4A2N1	5
A0A0G2K1B5	4
D3ZHT3	2
Q0KL00	1
A0A0G2JVB3	2
Q5YLM1	4
Q5PQM2	2
A0A096MJ27	2
D3ZYM5	2
P97756	4
M0RAP5	4
A0A0G2JX62	2
D3ZJ01	2
Q641W3	2
M0R617	3
A0A1W2Q689	3
Q5M7W6	5
B5DFE2	1
A0A0G2KA92	4
D3ZZT9	3
F1LZS6	3
D4A772	6
D3ZHL1	5
Q5FVR4	3
P0CF24	4
A0A0G2JWJ7	2
D3ZUC2	1
Q6AYC8	5
A0A0G2JZ92	3
A0A0G2K7Q9	5
Q63623	3
D3ZXM2	1
Q4G017	1
Q76KC6	4
A0A0G2K9N0	5
Q3T1J5	3
A0A0G2K0Y9	4
D4A753	4
D3Z8Z2	4
A0A0G2JU83	4
D3ZW33	5
D3ZU40	4
Q6AYC4	3

Q4KLN6	3
A0A0G2QC22	1
Q5PQS6	4
Q68FY0	6
B5DEJ5	4
O55197	1
A0A0G2K0Q5	1
D4A8C6	3
F1M8U2	6
P12785	2
H9N1L4	5
D4A1Y0	2
F1LRE5	6
G3V656	4
Q4V7C1	3
D3ZAB6	3
P16391	2
P02767	3
F1LSW6	5
D3ZGQ8	4
G3V9Y1	5
D3ZTC4	1
F1LMK2	5
P52631	1
P63004	4
A0A0G2K3K7	5
A0A3G1T2C5	5
Q7TPH2	2
D4A914	2
Q9QY02	4
E9PSU1	3
D3ZAT7	4
A0A0G2JUH4	3
F1LPD3	5
A0A0G2JYY3	3
D4A2B0	4
A0A0G2JU75	4
A0A0G2K6J1	3
Q66HG9	5
Q6AYD6	3
Q6AYQ0	4
A0A0G2JWF0	5
Q63768	6
A0A1P0PBZ6	3
M0R7U6	3
Q4V7C8	2

Q3B7U0	3
Q9JIK3	2
F1MAF2	4
D3ZM82	1
D4AB75	3
F1LSQ0	2
Q4FZU8	5
D4AD05	3
F1M547	1
Q68FU2	3
Q6RFZ7	3
F1M0A6	5
D4AAG9	3
B1WBX7	2
F1LZX5	2
D3ZX52	3
D4AAN8	1
D3ZMK9	1
B2GV15	2
D3ZTZ2	6
F1LNL2	3
A0A0G2K6E0	1
D4A1U1	5
Q501R9	5
B5DF64	5
D4A3D8	4
A0A0G2KA12	5
B4F7F2	3
F1LMR4	1
A0A0G2JX01	5
D4A9L2	5
B2RYP7	4
P49301	1
F1LXU0	5
O35786	3
F1LXA9	5
F1LRC6	2
O88521	6
Q00972	6
O35413	4
M0RDY6	1
D3ZTY1	2
D4A3V7	6
O70141	1
D3ZBY5	4
B2RYL3	1

A0A096MJ54	1
D4ACP5	3
D3ZTB5	1
D4A4Z9	2
Q5FVC5	3
P07861	4
Q2IBD4	3
Q4V8J7	5
P0C2N5	3
F1LWN1	2
D3Z941	6
F1M277	1
Q6AYU3	6
O08619	3
D4A4N9	4
Q9JLY8	1
B5DFG8	5
A0A096MKG5	4
F1M0V0	5
Q6AXX4	4
A0A0G2K0Z9	3
D3ZXZ1	4
M0RDJ7	5
F1M8T4	5
Q5RKJ2	5
B2RYH1	6
Q5PQJ8	1
P0C5Y8	4
B5DFL5	5
F1LZQ6	4
Q63632	1
Q6P777	2
Q5M9F3	4
D3ZIH4	4
D3ZSG1	3
M0R9X4	5
Q6AYL9	3
B5DF86	1
Q5EB62	4
G3V6B2	5
A0A0G2K016	3
D3ZU63	4
Q8CIV0	2
D3ZUQ8	2
D3ZZH6	5
Q5M836	6

Q3KRC3	4
Q499R0	5
D4AB03	1
Q5FVL6	2
D3Z8X7	3
D4A9P0	3
F1LVD8	2
A0A0G2K5U3	5
D4A729	5
D3G8R8	1
A0A0G2K0X8	3
Q5I0H5	3
Q99ND8	2
D4A5F1	4
Q498D5	4
P38438	5
B2GV96	4
A0A0G2K426	4
Q03062	1
D3ZHG4	1
Q2KJ09	4
B1H268	2
D3Z8B9	6
Q6P756	4
Q5XIU5	6
Q6AXS9	5
A1A5S2	5
F1M0C4	6
Q66HA4	4
Q923Z2	3
Q6AYX5	4
Q3KRE3	3
O08618	6
D3ZMY7	3
A0A140UHW6	3
Q5I0E3	3
D3Z837	6
Q569C0	6
Q9QWM1	4
D4A9A3	1
R9PXW1	4
D4A6E8	2
Q5XIR9	2
Q5EBC7	6
D3ZT47	3
F1M865	4

F1LYC7	4
B0BN89	5
Q5HZE9	3
F7FMK8	2
Q99MD2	5
O88450	5
G3V9Y6	2
G3V6X1	3
D3ZAR1	4
D3ZN60	3
D3ZJW3	5
D4ACB8	4
G3V9N1	2
Q66HC2	4
F1M6K4	1
A0A0G2K3D7	3
Q7TQ84	5
A0A0G2JTF0	4
P15257	6
Q6IE50	3
Q5BJU9	1
D3ZSU6	3
Q5U2Z5	4
O88777	2
D3ZYD7	3
F1M3W5	3
Q6EV76	3
Q6P686	3
Q810G8	3
D3ZPW6	4
D3ZYM4	4
M0R970	1
Q5FWU0	2
A0A0G2JWM2	3
D3ZZM3	6
A0A059NZR0	3
D3ZSX8	1
B2RYA6	1
Q5U2P2	6
D3ZQU7	6
A0A0G2KB11	2
Q45QJ4	6
Q9JHX4	1
D3ZJT6	5
A0A0G2K365	1
Q5RJS0	3

D3ZPP3	5
D3ZEY4	5
Q5FVM6	6
D3ZAI0	4
D3ZGZ9	2
P11505	6
Q76KC5	6
Q5XI72	6
O70617	4
D4A748	3
D3ZVD1	3
O35264	5
P19103	5
F1LZV1	4
P36972	1
P62142	4
Q6AXQ4	5
D4A533	5
E9PSK7	5
P49445	3
P97536	1
G3V7Q7	5
D3ZR69	1
Q9R0Q2	1
Q4V8C2	1
B2GV98	5
F1M5G8	4
D3ZVB3	5
B1WBS4	6
Q5XI44	3
D4A9B0	1
D3ZJX1	4
Q7M6Z3	1
Q63569	2
A0A0G2JTF5	3
Q5U2U8	2
D4A8N7	4
Q6AYG5	6
G3V6B7	3
D3ZQ30	3
A0A0G2JSV6	2
A0A0G2JWH3	3
A0A0G2K3S4	1
D3ZY01	1
G3V781	3
Q4G055	5

A0A0G2QC45	5
D3ZSV6	6
B5DF45	3
F1M324	1
Q66H43	3
D4A105	5
F1M8B7	3
Q5U2N1	4
B2RYH6	5
D3ZPX0	3
D4ADU2	2
D3ZL57	4
A0A0G2K062	1
A0A0G2JSH0	3
F1LYA6	1
D4A4W6	3
Q3KR53	1
Q5U2Z4	1
Q9EQR2	3
M0R6F6	4
Q99NI4	5
Q66HR2	6
F1M6E5	3
D3ZG10	6
Q6AYB0	4
D3ZIG8	5
G3V617	6
F1LU78	1
P54748	6
Q62771	4
Q6MGB6	2
M0R7I0	3
D4ADC7	5
D3ZZB2	2
A0A0G2K6Z1	6
Q4V888	3
F1M388	5
M0R6Z9	2
Q499Q2	6
Q5VJ70	5
F1M8W4	6
F1LXV0	6
B5DFE5	2
A0A0G2JW28	5
F1LSV8	4
P47196	3

O35274	4
D3ZN85	4
A0A0G2JWP1	3
O89037	1
D3XAM6	4
B3STT9	3
A0A0G2JSV2	2
Q9EQH5	4
A0A0G2KB97	4
B2RYN0	4
B2GV52	5
A0A0H2UHW4	3
Q9EST6	5
F8WG67	4
A0A0G2K4L3	5
Q9ET61	3
Q6AY66	1
G3V631	2
F1MAJ8	4
A0A0G2K0V8	1
P41499	2
P63088	3
D3ZLQ9	6
Q9Z2G8	3
G3V9S9	5
F1M8D5	5
O88794	3
D4A2W7	2
Q7TP58	5
D3ZZ25	5
A0A0G2K0K6	2
Q62885	3
D4ACE7	3
D3ZNG0	3
D4A3I3	5
D4AC07	1
D3ZVJ3	1
A1L108	5
M0RAC2	5
Q8CJ52	6
D3ZV40	6
D4A8M4	2
Q68G11	5
O55164	3
Q6AYZ7	4
B2GV12	5

G3V8M9	4
P11915	1
D3ZRG3	3
M0R8E0	6
P62775	3
F1LPI7	5
M0R4T9	6
Q7TST9	1
Q6PAH0	2
Q3B7U4	5
B2RYJ2	3
Q4G034	2
D3ZG88	6
B2RYJ1	1
D4A827	5
A0A0G2K4P5	1
B2BL37	2
G3V8P7	1
D3ZA22	2
Q91XP6	5
Q63713	3
B4F759	4
D3ZBK4	2
E9PU42	1
D3ZYT8	4
Q01728	1
Q5U2S7	5
D3ZK96	5
P13941	3
G3V9Z6	2
D4A962	4
B4F7A5	1
D4A2T7	3
A0A0G2JVW0	5
P0DL28	4
Q6JHU9	5
D3ZDC7	5
Q5XIX3	3
D3ZLS5	1
Q3L7M0	6
P81718	5
A2RRU4	1
F1LWB9	2
A0A0G2JSR0	2
A0A0G2JT30	3
Q5PPG7	3

A0A0G2K9L2	6
A0A128E118	3
D3ZZQ4	5
T2CB11	2
Q4FZR1	6
D4ADS9	5
Q4G045	5
Q3KR73	5
B5D5N9	4
P97887	3
P41232	4
A0A0G2K9R6	4
D3ZGM7	3
E9PTG5	6
Q63562	1
M0R469	5
Q80Z39	1
D3ZH41	4
A0A0G2K9Q1	5
F1M7L6	1
D3ZHG8	2
F7F5J1	3
Q498S6	2
F1LVA8	3
D4A240	4
A0A140TAD1	2
M0RAC7	4
Q6P751	2
A0A0G2K1P8	5
B0BMS8	4
D3ZT26	4
D4ABZ7	3
G3V9W6	3
Q5BJP0	1
Q5RJK5	3
F1LQX8	4
Q31274	1
D4A020	5
D3ZV96	6
O54748	3
A0A0G2JV54	4
D3ZBQ5	2
A0A0G2K2U0	5
A0A096MJ09	2
Q05BA4	4
M0R851	3

Z4YNI2	4
F1LR42	5
P40329	2
E9PU29	3
B5DF63	2
D4A5H7	5
Q5PPN2	1
D4A981	6
D3ZBN3	2
P48769	5
Q6AY70	4
D3ZH92	5
G3V6N3	2
F1LML0	5
D3ZPZ7	4
A0A0U1RRU7	5
D3ZAW6	3
Q6IE67	2
Q62760	4
Q9R064	6
A0A0G2K4W6	6
P34064	2
D4ADM2	3
A0A0G2K6H5	4
B5DFG9	6
A0A4X0W8E9	1
P55260	6
F1LY19	5
B5DFM1	5
D3ZCZ3	5
Q9Z1T4	4
Q5FVG8	6
Q6P2A5	6
D3ZE09	5
A0A0G2JVE9	3
D4ACN6	4
F1LRH8	1
I6L9G6	4
F1LM33	3
F1LWK7	2
A0A0G2KAJ5	4
F1M4U0	5
A0A0G2JTK4	3
P22509	4
F1M037	1
P21818	5

B1WC90	1
A0A096MK24	2
A0A0G2K4Y6	1
D4A1W5	2
F7IXA2	2
D3ZJ86	3
F1M3L7	2
A0A0G2JT65	5
D3Z8M3	3
F1LW07	5
D3ZUB0	5
Q6AZ33	2
D4ADF6	3
G3V6Y7	5
P55770	3
A0A0G2K952	2
Q5EB77	4
Q32PX7	5
A0A0G2JSH6	1
B1WC37	6
D3ZC89	5
D3ZLA6	1
Q9Z2P4	1
D3ZVC4	5
D3ZEM6	1
D4AE31	5
Q7TQ19	1
Q5U2P9	6
F1M3D2	5
Q25C79	1
D4ADE7	3
B1H266	3
A0A0H2UI14	3
A0A0G2K2P6	2
G3V8Y7	5
A0A0G2JV78	5
Q9R1U5	3
Q6IMX7	1
D3ZLD0	5
G3V6L8	5
Q91ZW1	5
B0LT89	4
P16617	3
D3ZTR5	5
Q4V8C4	2
D3ZSC1	2

Q4KLN4	5
B2GV33	4
F1LNX7	5
A0A0G2K3M5	4
B5DEI3	3
B0K014	2
Q923K9	4
A0A0G2JT07	2
Q68FP5	4
Q8CGS4	3
P08483	2
D4ADZ9	5
G3V8J0	5
F1LUU6	6
F1LPQ4	3
Q7TP39	5
D3ZV81	5
Q2I6B0	1
D3ZN16	2
Q9R050	3
D4AD85	6
Q9JHZ9	4
D3ZWZ8	1
D3ZWA1	3
F1M5M9	2
D3ZE59	3
Q5BJV3	5
G3V727	3
Q5I0G4	2
D3ZZA8	4
D4A1K4	6
Q4V8G6	6
D4A193	3
Q6TUH4	5
Q4V8I7	2
Q498T9	1
D3ZA02	4
I7FZ15	5
O55173	5
A0A0G2K429	2
F1M656	3
A0A0G2K8Z6	4
A0A0G2K8E6	4
A0A0G2KA68	6
D3ZS50	4
P35284	6

Q6AYY8	4
Q9JJP9	2
Q7TP93	3
D3ZMQ3	4
Q7TT49	6
D3ZTA3	3
B5DEY0	2
P41156	5
P97544	2
Q80U96	1
D3ZP89	5
Q9JHL4	1
G3V8P4	5
A0A0G2K781	3
D3ZLQ8	3
D3ZBE8	2
P97531	4
Q62896	6
F1LWT0	2
P02401	5
Q4QQV2	4
P48961	5
D3Z9T4	2
A0A0H2UH97	1
Q04666	4
Q3MJK5	1
A0A0G2K808	4
Q68FS9	1
Q2I6B1	5
F1LVA5	4
P69682	4
Q91ZQ0	5
P47820	4
P12369	5
A0A0H2UHS9	4
Q66H19	3
Q80ZG2	1
F1LTF8	3
A0A0G2K4R5	4
Q5XIH0	5
Q9ET58	1
A0A096MK35	2
A0A0G2K219	1
A0A0G2K4K3	1
F1LR71	6
D3ZQI6	5

B4F7F1	5
Q5FVP5	1
P55053	2
E9PSW6	1
G3V8J4	1
A0A096MK18	4
Q63259	5
Q4QQW1	6
D3Z9J7	2
M0R608	3
D4ABY4	6
A0A096MJZ0	4
A0A0H2UHK0	6
Q6IE70	2
Q6DGF9	2
D4A544	3
Q4FZS6	4
P11345	4
A1A5L2	1
A0A0G2KA14	2
D3ZLR4	6
B2GV14	1
A0A0G2KAW5	5
P59722	5
P12368	4
Q5XIN1	3
A0JPQ7	6
Q5M9H0	1
B8PS70	3
Q5XI25	4
F1LMN3	1
Q5M7A4	6
D3ZJK8	3
P19112	6
D3ZM07	6
D4A346	3
D3ZUL1	2
E9PT74	6
A0A0G2K1U9	3
Q6AY65	6
Q5XI29	6
B1WBZ7	2
A0A096MKA6	4
D4A0R1	1
P52590	4
Q64542	1

F1LRI5	6
P70553	4
G3V6T9	3
P28494	2
Q75Q41	3
D3ZK26	1
Q64725	3
A0A0G2K9S4	3
M0RBY8	3
D4ABP3	5
Q924T8	3
D3ZK73	4
D4A6T9	3
Q4Z8P1	3
G3V7X5	4
B1H216	3
A0A0H2UHX1	4
F7EZF5	5
Q63639	3
D3ZGL1	2
Q4V7C0	2
B2GV01	2
G3V7W0	1
B5DFA0	2
G3V6F5	2
B2RZA4	4
D4AEG3	1
G3V619	4
A0A0G2K9T0	1
Q4KLK2	6
Q6TXG4	4
O54921	3
Q5XIL2	6
Q91Y81	5
D4A0E2	4
A0A0G2JXT6	5
M0R963	5
Q9WVA1	2
D4ABX6	4
Q4V8J4	5
Q5XIM5	6
A0A0G2K9S1	5
B5DEZ0	4
D4A3K3	1
Q5I0D2	5
Q5I0K2	3

Q4KLI1	3
P21708	2
Q80W87	2
M0R9X8	4
F1LST1	1
D3Z955	4
A0A0G2K2V4	2
P24528	6
D3ZWL6	2
F1M4J0	4
F7FH17	1
G3V8U3	2
D4A4T0	5
D4A778	5
Q5I6B8	3
B5DEJ9	3
D3ZQJ0	3
Q6AZ53	3
A0JPP8	5
Q9Z0G8	5
A0A0G2K6Q5	6
M0RCV5	1
Q64617	3
Q0PMD2	1
P49743	1
B1WBQ0	5
E9PTX9	6
G3V6K4	2
P26376	3
Q9QYP1	3
Q66H15	5
A0A0G2JTM7	5
D3ZKY5	2
Q5D1N7	3
D3Z8D3	5
D3Z994	2
