# Supplementary material for: Integrative proteomics, phosphoproteomics and acetylation proteomics analyses of acute pancreatitis in rats
Source: Int J Med Sci. 2023 May 11;20(7):888–900. doi: 10.7150/ijms.81658 (PMC10266050; doi:10.7150/ijms.81658)
Supplement: Supplementary file 5 — Supplementary table 4. [file ijmsv20p0888s5.pdf]

Protein	cluster
F1M853	6
Q3KRF2	1
F1M779	3
Q66HD0	6
P06761	1
P04785	6
Q6P3V9	6
Q6IMZ3	2
P56574	6
A0A0G2JZI2	6
P10860	6
A0A0G2JZ69	5
P46462	4
P28037	6
P21531	6
Q10758	3
P62630	2
A0A0H2UHM5	6
P49242	1
Q66X93	3
P54316	1
B0K031	6
G3V9M6	6
P04636	4
D3ZTP0	1
Q6P6R2	6
Q9ER34	6
G3V7J0	5
Q6P136	1
P18418	6
P51647	6
Q07936	3
G3V6T7	2
X1WI37	6
Q6P7A7	4
Q64428	2
A0A0G2K401	3
P13803	5
P35565	6
P09895	1
G3V836	3
P54318	1
G3V852	3
B2GV15	6
P31000	2

Q9Z2L0	6
P06685	6
P23514	3
Q5RK10	6
G3V6S0	6
Q68FR6	5
F1LST1	3
A0A0G2JTL5	3
Q7TQ70	3
P11507	5
P34058	2
P82995	2
A0A0G2JZ73	3
P62282	6
Q8VHF5	2
Q4G061	1
Q66HF3	5
P29314	2
Q66H80	6
P00731	6
Q641Y8	1
Q5M875	6
D3ZD23	1
Q5M7X1	1
Q5I0G4	6
A0A0G2K2Q2	5
Q5XIH7	1
P62243	6
Q5BJY9	2
A2VCW9	1
F1MA56	2
P67779	4
Q5U328	2
P19945	1
A0A0H2UHE1	1
Q9Z0V5	1
Q4G075	1
Q5D059	2
G3V7U4	2
P85834	1
A0A0G2K3Z9	6
Q01129	6
P31399	4
Q5XIT9	6
P00774	1
B5DEL9	1

Q6P9U5	2
O70351	6
F1LNF1	1
P14408	6
P15999	5
Q9JLA3	2
F1LRJ9	5
P24049	1
G3V9K0	5
P41777	6
G3V9S9	1
P38983	6
P14668	3
Q63507	6
P62260	1
P29315	1
P24329	1
Q6PDW1	1
B0BMT9	1
P0C5H9	6
P62278	2
P25235	6
Q9WVJ6	1
P04642	2
Q68FS4	1
P04762	1
Q6P2A5	1
Q6PDV7	6
B0BN81	6
Q6P502	4
P14141	6
Q9JKB7	2
P50475	5
Q06647	5
P10111	2
Q4QQV4	1
D3ZQN7	3
D3ZCT7	1
Q6T487	2
P12007	6
D4A1Z2	6
B5DFC8	6
B2RZ37	6
Q66HF1	6
Q04462	2
Q68FQ0	4

Q499V7	4
D3ZIE9	6
P50399	3
P26772	6
P10888	1
P83732	5
B2RYI2	2
P45592	2
P32551	3
P14480	4
F1LT35	6
Q60587	1
O08557	6
M0RDK9	5
Q5XIH3	6
Q3KRC3	1
F1MA98	2
Q00438	2
F1M062	6
B2RZ24	6
Q641X8	5
P04646	1
P81155	2
P06866	3
Q3MHS9	3
Q63355	3
Q62667	3
P08426	6
P61314	1
P62828	2
Q3KR86	6
Q4G079	6
P18420	1
O35244	6
P51886	2
P70584	6
P29266	6
P07632	6
P62832	2
Q6P6V0	3
P25113	1
B4F768	2
P11030	4
P04256	6
G3V7G9	6
F1LY19	1

D3ZFG3	6
Q923M1	1
Q499N5	6
P13255	1
P62268	6
Q9Z0J5	1
Q5BK63	1
P02401	2
O88656	2
Q794E4	1
D3ZSA9	1
P00173	2
G3V7V5	5
A0A0G2QC06	6
Q5XI38	2
P14942	4
Q5XFX0	2
B5DEN5	6
F1LNH3	2
Q6P6T6	3
D3ZU13	5
Q5VLR5	4
Q642E2	1
A0A0G2JSR7	6
O35775	6
Q4KM73	6
P30349	1
Q794F9	3
Q63584	1
Q6JHU9	2
P68255	2
D4A781	5
F1LU71	6
M0RAK2	4
B0BN52	2
Q3MIE4	2
P62329	3
Q6P747	1
P62902	1
P47853	3
B2RZD4	6
P63269	2
Q6AYG5	2
F1LQI1	5
D3ZUF9	2
B2GV99	2

A0A0G2JSH9	6
D3ZZT9	1
Q5XIH1	6
P84092	2
P13221	1
Q6P3V8	1
Q5U3Z7	3
P50503	5
Q6AYB5	4
Q9QZ86	3
D3ZAF5	2
A0A0G2JW90	6
F1LM33	4
B5DFA0	6
Q6TXG7	6
A0A0G2K248	2
G3V8B6	4
Q68FY0	1
Q6P9V7	6
P62959	6
F1LP82	4
F2Z3Q8	1
P62982	6
P24155	2
P62963	2
P42123	2
P09495	3
G3V7I0	6
P27139	2
D4A3K5	2
P61589	1
Q4KMA2	3
P48004	3
P10758	3
A0JPJ7	4
A0A096MK30	3
G3V7T6	2
A0A0G2JVD6	3
G3V9Y1	5
D3Z900	3
P07314	6
P21913	6
Q9JI85	1
P29418	6
A0A0G2KAP1	1
A0A0G2JVG4	5

Q641Y2	1
B6DYQ9	6
F7EV94	1
Q6P7R8	5
F1LRV4	4
A0A0G2JUC7	6
Q63610	5
Q5M963	2
B1WC61	4
Q6AY58	2
Q5RK08	1
B0BNG3	2
Q924S5	5
P84100	6
Q4KLN7	4
P06302	3
Q6MG61	3
Q5XI32	5
A0A0G2JTG7	4
P0DMW0	4
P02767	6
P50398	1
P25886	6
Q6PAH0	3
F1LQS6	3
P24473	5
Q6AY18	5
A0A140UHY3	2
P62856	1
Q7TP42	1
B5DF65	1
Q62636	2
P07335	3
D3ZZN3	2
Q5XI85	1
P05370	2
D3ZZR9	1
Q9JJ54	5
Q920L2	6
B2GV92	3
Q5XIC6	5
D3ZD09	1
D3ZZH2	2
P04055	1
Q561S0	5
Q6PDV8	1

Q498U4	6
P18445	6
P69897	3
G3V7Y3	6
A0A0G2JZF0	1
D3ZVQ0	6
D4A206	2
D4AC36	1
D3ZD97	2
B0K020	6
B2RYN0	2
P00762	6
A0A0G2K1Q1	1
Q9QX79	2
Q91Y81	2
B2RYN6	1
A0A0G2K7R1	2
D3ZUX7	1
O35077	1
G3V6C4	4
P23457	1
A0A0G2JSU3	3
D4AE49	3
P29419	6
D4A8T3	6
B0K008	1
Q01205	1
P10760	2
A0A0G2JZH8	5
G3V8A7	1
P29117	3
Q3MHS7	6
D3ZFJ6	6
P39052	2
P27657	1
Q6TUG0	1
D3ZF12	6
Q5U2N2	2
A0A0H2UHP9	1
B0BNM1	5
Q9ESH1	1
D3ZN76	1
D3ZPV8	4
P97519	4
A0A0G2K3C1	3
Q6AY30	5

P12075	2
F7EL36	2
Q5U2X6	1
D4A746	5
G3V7L6	1
P09656	3
P52555	3
Q5BJP3	5
Q9QVC8	3
Q5U2S7	4
Q6P6U2	6
Q3B8N9	1
B2GV73	3
D3ZZZ9	3
D3ZG43	5
P20788	1
A0A0H2UHE4	3
D4AEH3	4
D4AB01	1
P14669	1
D3ZNA3	6
Q5XHZ0	1
D3ZE72	5
A0A0G2K1M2	1
Q63524	1
Q5BKA1	1
D3ZNQ6	2
D4A9T6	1
E9PST5	3
Q5PQR0	2
Q9EQP5	3
M0R9Q1	2
F1LSP2	1
Q6B345	3
P12368	1
G3V827	1
O08619	2
Q91ZN1	3
F1LRB8	4
P17136	2
D3ZQM0	4
B1WC67	2
F1LPC7	1
D3ZF39	3
A0A0G2JSS9	6
Q99MZ8	1

Q9WUC4	1
Q6AXS5	1
Q641Z6	5
M0R3V4	6
Q5I0E7	6
Q6AYK6	1
D4A1Y5	4
A0A0G2K1Q7	1
Q5PQZ9	1
F1M024	5
O35264	2
P62859	1
Q32PX2	1
G3V661	4
Q5EBD4	4
Q704E8	3
Q62651	2
P28492	1
D4ACV3	2
P20761	4
P63312	3
A0A0G2K0V8	5
Q5XIN6	5
P62275	6
D3ZTX0	1
D3ZZ99	2
A0A0G2K8H0	5
Q3ZB97	1
F1LN30	4
O35567	6
G3V7L8	5
Q6AYQ8	1
B2GUZ3	1
M0RBI3	6
Q62780	3
G3V6S3	4
M0R9L0	2
P63255	2
Q5U2R9	1
P11240	5
P63329	1
Q5M943	6
D4A7U1	3
Q6P792	2
D3ZRC3	2
B2GUY6	1

B5DFI1	5
F1LND7	1
B2RYX1	1
D3ZVS2	1
F1M7S4	5
Q9R1T1	3
P70580	1
A0A0G2K1N3	5
D4A500	3
Q7TQ16	1
G3V8Q1	6
Q9Z269	1
F1LN92	6
P97584	5
F2Z3T7	1
D4A3V2	3
Q5FWU2	5
B4F764	2
D3ZAS8	1
A4L9P7	2
Q5I0P2	1
P62142	1
Q5XI07	1
M0R7B4	4
D3ZNJ5	2
P15865	4
F1LNL2	3
P30835	6
D3ZGU2	4
O70185	1
Q3KRE2	6
F1LNP1	2
D4A0Y4	2
D3ZCZ9	2
B5DEQ4	5
D4A197	6
A0A0G2JVG3	2
Q5I2Z0	1
A0A0G2KB11	1
A0A096MKG5	1
A0A0G2K2L8	1
Q6RUV5	2
B1H248	1
A0A0A0MY43	4
Q9Z2G8	2
Q7TP78	1

B0BNB4	2
M0RAD5	1
Q4KM66	5
F2Z3T4	1
B5DEP9	3
G3V7Q6	2
A0A0G2K7Q6	1
Q3S4A4	5
P40307	1
Q68FP2	5
Q3KRE0	2
B0BMY8	4
D3ZE63	2
D3ZXF9	1
Q5BJR5	6
A0A0G2K1A1	1
P27321	2
A0A096MKF8	4
P32089	2
P40112	3
O35824	1
D3Z7Z4	1
Q8SEZ0	1
Q5PQK5	1
D3ZQV0	1
Q9Z0W7	3
D3ZLF6	3
Q6AYJ1	5
P24051	6
A0A0G2K3V2	1
A0A0G2K654	2
Q5XIA5	6
Q6P6T0	1
Q8CHN6	6
D4AEB4	1
Q66HG6	1
A8C4G9	2
D3ZD73	5
P13668	3
D3ZBG6	5
A0A0G2JWV2	3
Q9ERU2	1
O88350	2
Q04931	6
Q5U1W8	6
G3V6L8	2

D4AAG8	5
M0R4L7	3
B2RYT5	1
G3V9C7	2
A2VCW2	1
A0A0G2K350	3
G3V6S8	3
A8IRI3	3
F1MAD2	3
Q5M9I5	1
Q6QI86	4
D4A4J0	2
B1H240	2
Q99J82	2
A0A0G2JYW3	3
Q68FW7	1
O88794	6
B0K017	1
G3V7C6	3
D3Z952	1
O70437	4
Q78P75	2
D4AE03	3
Q66HG5	3
Q63011	3
B2RYM5	6
O08658	2
P05964	3
A0A0G2K8B7	1
B5DF51	5
A0A0G2JX25	5
P07151	3
A0A0G2K2X3	3
Q5M876	1
Q63223	3
M0RA08	3
G3V7G0	2
D4A666	2
P09655	1
B0BNE1	3
A0A0G2JUE4	4
B2RYS0	1
Q6AY23	3
Q75WE7	2
G3V843	3
D4A8U7	1

Q9EST6	4
Q02356	2
A0A0H2UHZ4	1
P12346	2
Q9ET64	6
F1LWX5	2
Q7M0A0	2
A0A0G2K0W0	5
D4A0U3	6
Q66HT2	2
Q6P7P8	2
D3ZZQ6	3
D4A6A2	3
M0R6T1	3
D3ZUD8	1
D3ZBP3	1
Q6P7R7	6
B2GV72	1
Q64361	2
G3V6H0	5
A0A0G2KAI8	6
Q32PZ7	6
F1M9C9	2
D3ZGR7	3
P62634	1
D3ZN95	2
Q5I0L3	1
B5DF89	3
Q9WU49	6
Q5M7V8	2
B2RZ74	3
A0A0G2JV54	1
Q5RKH8	2
Q6MGB4	4
F1LPB3	4
G3V946	4
Q62839	4
A0A0G2KA82	6
Q64303	2
D3ZD80	4
A0A0G2QC34	6
A0A0G2K2W2	1
M0R4T9	6
P97564	1
D4A1R8	1
Q4KLP0	3

Q4R1A4	6
D3ZIT4	6
Q6AYQ4	1
A0A0G2K3H2	3
F1M2K3	4
P53812	4
Q76MV3	6
Q64602	4
Q6AYJ7	4
A0A0G2K5I9	2
A0A0G2KAX2	2
D4A8C6	2
D3ZMQ0	2
Q9EPJ3	3
F1LRV9	1
Q5U312	4
Q3B7T6	1
F1M4G6	4
A0A0G2JV35	2
D3ZLY9	3
D3ZYQ8	1
B2RYN1	3
A0A0G2JT35	1
E9PTN1	3
P19223	1
Q63287	3
Q3KR73	5
G3V9U2	6
Q4V8F5	2
Q03114	1
D3ZUW8	4
P05942	3
B5DFB2	3
Q9JKC9	4
D4ADG2	1
B5DEQ0	1
P0C606	2
O08559	1
D4A817	3
Q9JM77	1
P62193	1
D3ZNH4	3
B1WBY2	4
A0A0G2JWF7	3
D3ZZP2	3
B2RYJ4	1

D4A7L6	3
Q5XI18	1
G3V9W4	6
D4A4A9	6
Q6P686	4
Q5FVL2	6
F1LVG9	3
A0A0G2K761	2
D4AEL0	1
Q5XIB5	3
Q6AY57	6
Q6PDU2	1
D3ZFP6	1
D3ZIF0	2
D4A538	1
Q9Z1L0	4
A0A0G2K3W1	5
Q5RK30	4
Q5RKH0	2
Q6TXG9	1
Q52KS1	3
Q4KM87	2
A0A0G2K906	2
Q6MGC4	1
Q9R0T4	1
D3ZSW3	3
D3ZHE7	4
Q5XI39	4
Q6IG09	3
B0BNJ4	6
A0A1B0GWT1	3
A0A0G2K5M6	6
D4A4L4	3
D3ZGQ8	2
F1M1X9	2
Q7M076	3
F1LZ43	1
D4A411	6
Q5U2Y3	2
Q91ZW6	3
Q5XI63	3
Q5M878	4
A0A0G2JYA7	5
D3ZXA6	2
G3V9A4	3
Q5XIF4	4

Q9ESZ0	3
B5DFE9	3
P63018	2
F1LSH0	2
F2Z3T8	3
D3ZYK9	1
D3ZS58	6
Q5M949	6
Q6AXR6	5
Q5EB92	1
M0RAD8	1
A0A0G2JU43	6
Q566Q8	1
B5DFN4	2
Q5XI15	1
A0A096MK48	5
D4A930	2
B2GVB9	2
M0R8V7	6
A0A0H2UHG7	6
D3ZWM5	3
D3ZBD0	3
P60868	6
D3ZXP3	1
D3ZHD7	4
Q91XT0	4
Q66H19	2
Q5I0K4	6
D4A586	2
P28648	2
F1LY38	2
B0BNB2	2
A0A0H2UHH3	1
D3ZBP2	4
Q66HA7	3
Q6TQE1	6
